# Correction of cognitive deficits in mouse models of Down syndrome by a pharmacological inhibitor of DYRK1A

**DOI:** 10.1242/dmm.035634

**Published:** 2018-09-27

**Authors:** Thu Lan Nguyen, Arnaud Duchon, Antigoni Manousopoulou, Nadège Loaëc, Benoît Villiers, Guillaume Pani, Meltem Karatas, Anna E. Mechling, Laura-Adela Harsan, Emmanuelle Limanton, Jean-Pierre Bazureau, François Carreaux, Spiros D. Garbis, Laurent Meijer, Yann Herault

**Affiliations:** 1 Institut de Génétique et de Biologie Moléculaire et Cellulaire, Department of Translational Medicine and Neurogenetics, 67400 Illkirch, France; 2 Centre National de la Recherche Scientifique, UMR7104, 67400 Illkirch, France; 3 Institut National de la Santé et de la Recherche Médicale, U964, 67400 Illkirch, France; 4 Université de Strasbourg, 67400 Illkirch, France; 5 ManRos Therapeutics, Perharidy Research Center, 29680 Roscoff, Bretagne, France; 6 Faculty of Medicine/Cancer Sciences & Clinical and Experimental Medicine, University of Southampton, Center for Proteomic Research, Life Sciences Building 85, Highfield, Southampton SO17 1BJ, UK; 7 Laboratory of Engineering, Informatics and Imaging (ICube), Integrative multimodal imaging in healthcare (IMIS), UMR 7357, and University Hospital Strasbourg, Department of Biophysics and Nuclear Medicine, University of Strasbourg, 67400 Illkirch, France; 8 Department of Radiology, Medical Physics, Medical Center – University of Freiburg, Breisacher Strasse 60a, 79106 Freiburg, Germany; 9 Université de Rennes 1, ISCR (Institut des sciences chimiques de Rennes)-UMR, 6226, 35000 Rennes, France

**Keywords:** DYRK1A, Kinase inhibitor, Leucettine, Down syndrome, Synapsin

## Abstract

Growing evidence supports the implication of DYRK1A in the development of cognitive deficits seen in Down syndrome (DS) and Alzheimer's disease (AD). We here demonstrate that pharmacological inhibition of brain DYRK1A is able to correct recognition memory deficits in three DS mouse models with increasing genetic complexity [Tg(*Dyrk1a*), Ts65Dn, Dp1Yey], all expressing an extra copy of *Dyrk1a*. Overexpressed DYRK1A accumulates in the cytoplasm and at the synapse. Treatment of the three DS models with the pharmacological DYRK1A inhibitor leucettine L41 leads to normalization of DYRK1A activity and corrects the novel object cognitive impairment observed in these models. Brain functional magnetic resonance imaging reveals that this cognitive improvement is paralleled by functional connectivity remodelling of core brain areas involved in learning/memory processes. The impact of *Dyrk1a* trisomy and L41 treatment on brain phosphoproteins was investigated by a quantitative phosphoproteomics method, revealing the implication of synaptic (synapsin 1) and cytoskeletal components involved in synaptic response and axonal organization. These results encourage the development of DYRK1A inhibitors as drug candidates to treat cognitive deficits associated with DS and AD.

## Abstract (precis)

**Summary:** Normalization of DYRK1A kinase activity can be achieved in three different mouse models of Down syndrome with pharmacological inhibitors of DYRK1A, leading to correction of recognition memory deficits.

## INTRODUCTION

Down syndrome (DS) results from the trisomy of human chromosome 21 (HSA21). It is still the most frequent intellectual disability, affecting 1 newborn per 700 births. Among the most common DS features are hypotonia, dysmorphic features and intellectual disability (Sureshbabu et al., 2011; Morris et al., 1982). Although children with DS show good socialization skills – encompassing social relations, friendship and leisure activities – they exhibit difficulties in communication abilities, i.e. the daily use of receptive, expressive and written language (Marchal et al., 2016). They experience troubles in daily life skills, such as self-caring, eating, toileting, dressing, behaving safely, and conceptualizing time and money. Improving the intellectual quotient of DS people would allow them to achieve more independence, increase their vigilance and globally improve their quality of life.

Among candidate genes explaining intellectual disabilities in DS people, the dual specificity tyrosine-phosphorylation-regulated kinase 1A, *DYRK1A*, is located in the DS chromosome 21 critical region (Walte et al., 2013; Duchon and Herault, 2016). It encodes a serine/threonine kinase which has numerous substrates. Two nuclear localization signals confer nuclear activity to this kinase (Alvarez et al., 2007), through interactions with transcription factors including GLI1 (Mao et al., 2002), RNA POL II (Di Vona et al., 2015) or splicing factors like cyclin L2 (Graaf et al., 2004). In the cytoplasm, DYRK1A phosphorylates cytoskeletal substrates such as β-tubulin, MAP1A or MAP1B (Ori-McKenney et al., 2016; Murakami et al., 2012; Scales et al., 2009). DYRK1A plays a role in cell cycle regulation by phosphorylating the cyclin-dependent kinase (CDK) inhibitor KIP1 (also known as CDKN1B) in cultured hippocampal neurons and in embryonic mouse brain (Soppa et al., 2014) and LIN52 *in vitro* (Litovchick et al., 2011). Through its ‘priming’ activity for glycogen synthase kinase 3β (GSK-3β)-dependent phosphorylation, DYRK1A regulates the nuclear/cytoplasmic localization of the NFAT transcription factors (Arron et al., 2006). At the synaptic level, DYRK1A binds to N-methyl-D-aspartate receptor subunit 2A (GLUN2A; also known as GRIN2A) and synaptojanin 1 (SYNJ1) (Chen et al., 2014; Grau et al., 2014) and phosphorylates amphyphysin 1 (Murakami et al., 2012) and GLUN2A (Grau et al., 2014). These are examples of different biological brain functions controlled by DYRK1A which are probably dysregulated when DYRK1A is overexpressed in DS, leading to cognitive impairments.

Several mouse models overexpressing DYRK1A have been described. The first one, Tg(CEPHY152F7)12Hgc, carries a single copy of a yeast artificial chromosome (YAC) containing a 570 kb fragment of human DNA encompassing *TTC3*, *DYRK1A* and *KCNJ6*. This model shows no strong defect in spatial learning and memory, but displays less crossing of the site where the platform was during the probe test in the Morris water maze (MWM) task (Smith et al., 1997). Another model, Tg(MT1A-*Dyrk1a*)#Xest (#=9 or 33), was produced by expressing the *Dyrk1a* rat complementary DNA (cDNA) under the control of the metallothionein 1a exogenous promoter (Altafaj et al., 2001). These mice demonstrated impairments in neuromotor development and hyperactivity evaluated in treadmill performance and rotarod tests (Martínez de Lagrán et al., 2004). They also display defects in visuospatial learning and memory in the MWM task (Martínez de Lagrán et al., 2004; Pons-Espinal et al., 2013), as well as in recognition memory revealed in the novel object recognition (NOR) task (de la Torre et al., 2014). A third model, Tg(*DYRK1A*)36Wjs, was generated using a bacterial artificial chromosome (BAC) containing the human *DYRK1A* gene. *DYRK1A* triplication leads to alterations in synaptic transmission with an increase in long-term potentiation (LTP) and a decrease in long-term depression (LTD). The transgenic mice are also deficient in the MWM task, suggesting spatial learning and memorization disabilities (Ahn et al., 2006). Although the human YAC and BAC transgenic mice exhibit features similar to those seen in DS patients, they carry an extra copy of human/rat *DYRK1A* gene, which could lead to biased phenotypes, as optimal expression and functionality of the human/rat protein cannot be ensured in a mouse background. Therefore, a BAC transgenic model with the entire *Dyrk1a* murine gene, Tg(*Dyrk1a*)189N3Yah [hereafter referred to as Tg(*Dyrk1a*)], was created (Guedj et al., 2012). This model shows alterations in short-term memory in the Y-maze task, and in spatial memory in the MWM task (Souchet et al., 2014). Deficits in cortical synaptic plasticity were also observed (Thomazeau et al., 2014). Comparable impairments were seen in the Ts(17^16^)65Dn model (hereafter referred to as Ts65Dn), a mouse model trisomic for almost 13.4 Mb, homologous to HSA21 and containing *DYRK1A* (Reeves et al., 1995). Spatial memory, especially reversal learning reflecting cognitive flexibility, was altered in the Water T-maze test and in the reversal version of the MWM (Olmos-Serrano et al., 2016). Although the Ts65Dn model has been widely used to study DS features, it carries a triplication of genes located in a subcentromeric region of mouse chromosome 17 (MMU17) which are not syntenic to any HSA21 genes (Duchon et al., 2011). A complete DS model, Dp1Yey, was thus produced, which is trisomic for 22.9 Mb, spanning the entire HSA21 region on MMU16 (Li et al., 2007). Dp1Yey mice are less well performing than control mice in the MWM task and display context-associated learning deficits in the fear conditioning test (Yu et al., 2010).

Reducing DYRK1A overdosage leads to correction of several DS traits, demonstrating the major implication of this kinase in DS. Indeed, normalization of DYRK1A expression attenuates spatial learning as well as associative memory defects, and rescues LTP in the Ts65Dn model (García-Cerro et al., 2014; Altafaj et al., 2013). In addition, reversal to two DYRK1A copies in Dp1Yey mice enhances working and associative learning performance assessed in the T-maze and contextual fear-conditioning tests, respectively (Jiang et al., 2015). Furthermore, epigallocatechin gallate (EGCG), a natural polyphenol found in coffee, cocoa and green tea, reported to inhibit DYRK1A, restores intellectual capacities of trisomic mice (Guedj et al., 2009; de la Torre et al., 2014). EGCG has undergone a phase 2 clinical trial (de la Torre et al., 2016). However, EGCG also interacts with the cannabinoid receptor 1 (CNR1) (Korte et al., 2010). This receptor modulates the release of neurotransmitters in various brain areas, such as the prefrontal cortex and hippocampus, thereby controlling memory, cognition processes and mood. Interaction of EGCG with CNR1 might thus affect memory, cognition and pain perception, leading to psychiatric disorders (Freund et al., 2003; Wilson and Nicoll, 2002), compromising its therapeutic use. Furthermore, DYRK1A is less sensitive to EGCG [half-maximal inhibitory concentration (IC_50_), 0.33 µM] than vimentin (IC_50_, 0.003 µM) and the laminin receptor (IC_50_, 0.04 µM) (Khan et al., 2006; Yang et al., 2009). Cognitive restoration in trisomic mice by EGCG might thus be due to inhibition of targets other than DYRK1A. Consequently, alternative pharmacological inhibitors have started to emerge (Kim et al., 2016; Nakano-Kobayashi et al., 2017; Nguyen et al., 2017). Nevertheless, all available results clearly demonstrate the implication of DYRK1A in DS intellectual deficiencies and the beneficial effects of its inhibition on the correction of cognitive deficits.

DYRK1A has become a major screening target for the development of selective and potent pharmacological inhibitors (Smith et al., 2012; Stotani et al., 2016; Nguyen et al., 2017). We here investigated the effects of a relatively selective DYRK1A inhibitor, leucettine L41 (hereafter referred to as L41) in three different trisomic mouse models with increasing genetic complexity: Tg(*Dyrk1a*), Ts65Dn and Dp1Yey. Leucettines are derived from the marine sponge alkaloid Leucettamine B (Debdab et al., 2011; Tahtouh et al., 2012). The chemically synthesized L41 displays a high selectivity for DYRK1A but also DYRK1B, DYRK2 and some Cdc2-like kinases (CLKs) (Fig. 1). It acts by competing with ATP binding to the kinase catalytic site. We here establish a proof of concept that pharmacological inhibition of brain DYRK1A is able to correct NOR cognitive impairment in three DS models with increasing genetic complexity. We show, via brain functional magnetic resonance imaging (fMRI) in Dp1Yey, the most complete mouse model of DS, that such cognitive improvement is paralleled by significant functional connectivity remodelling of core brain areas involved in learning and memory processes. Furthermore, phosphoproteomic analyses in the Tg(*Dyrk1a*) model unravelled brain DYRK1A targets for which phosphorylation increases with DYRK1A overdosage and decreases following L41 treatment. These novel substrates, such as synapsin 1 (SYN1), also found in the phosphoproteomic analyses of Ts65Dn, the most used DS model, bring new insight into the role of DYRK1A, and allow us to propose some dysregulated biological processes related to axonal organization and synaptic response which are responsible for cognitive deficits associated with DS.

## RESULTS

### Leucettines restore cognitive function, assessed in the NOR test, through kinase inhibition in three DS mouse models overexpressing DYRK1A

To investigate the importance of DYRK1A in cognitive deficits shown by transgenic mouse models of DS, we used a series of low molecular weight pharmacological inhibitors, collectively known as leucettines (Debdab et al., 2011; Tahtouh et al., 2012; T. Tahtouh, unpublished). We selected the well-characterized leucettine L41 as an archetype of this inhibitor family (Fig. 1) and L43, a closely related analogue which displays little kinase inhibitory action. Because both compounds were found to inhibit CNR1 (T. Tahtouh, unpublished), we also used L99, a DYRK1A inhibitor lacking activity on CNR1 (Fig. 1). To ensure its brain bioavailability, L41 was dosed following acute intraperitoneal (i.p.) injection in Tg(*Dyrk1a*) and wild-type (wt) mice. Plasma half-life was ∼45 min, and the inhibitor reached a maximum brain concentration at 20 min, and was eliminated 2 h later. No differences in L41 pharmacokinetics or biodistribution were observed between transgenic and wt mice (Fig. S1).

**Fig. 1.**
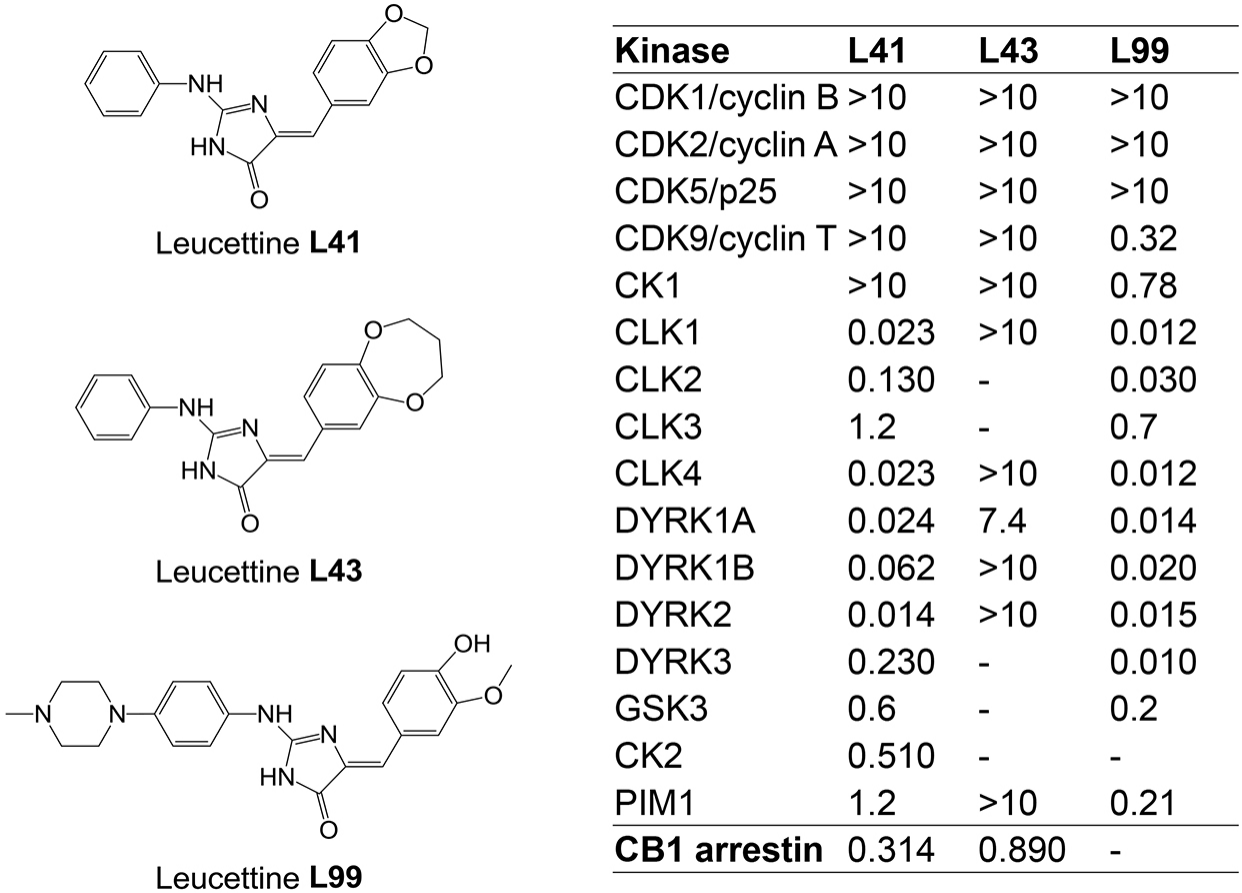
**Chemical structure and selectivity of the leucettines used in this study.** Selectivity of leucettines L41, L43 and L99 was assessed *in vitro* on 16 recombinant kinases, and in a cellular CB1 annexin assay. Dose-response curves provided IC_50_ values (reported in µM). –, no inhibition at 10 µM.

We used three mouse models of DS: Tg(*Dyrk1a*), which expresses a single additional copy of DYRK1A (Guedj et al., 2012); and Ts65Dn (Reeves et al., 1995) and Dp1Yey (Li et al., 2007), which carry MMU16 segments encompassing *Dyrk1a*, with 89 and 101 genes homologous to HSA21, respectively (Gupta et al., 2016).

Using the NOR test, we first evaluated the effects on Tg(*Dyrk1a*) animals following daily i.p. treatment with L41 (20 mg/kg) for 5, 12 or 19 days (Fig. 2A). As expected, untreated wt mice discriminated the novel over the familiar object. L41 treatment for 5, 12 or 19 days had no effect on the performance of wt animals. Untreated Tg(*Dyrk1a*) mice were unable to discriminate the novel over the familiar object (de la Torre et al., 2014) (Fig. 2A). In contrast, L41-treated Tg(*Dyrk1a*) mice preferentially explored the novel object, thus reverting to the behaviour of wt animals. This recovery was fully observed following 19 days of treatment, but was consistently or only marginally seen following 12 and 5 days of treatment, respectively (Fig. 2A). In other words, a minimum of 12 days of daily L41 treatment was necessary for full recovery in the NOR test.

**Fig. 2.**
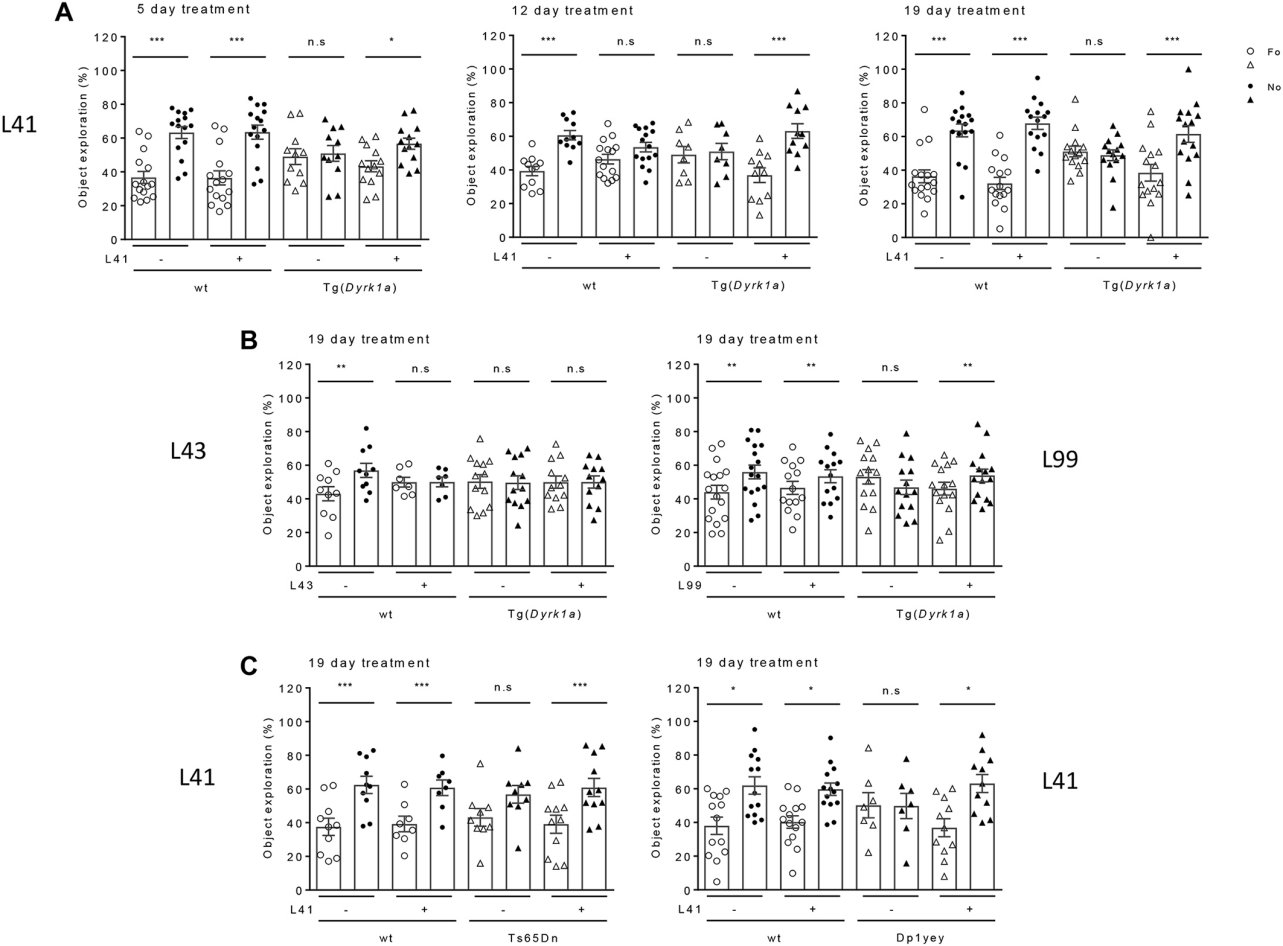
**DYRK1A-specific inhibitors rescue NOR deficits induced in Tg(*Dyrk1a*), Ts65Dn and Dp1yey trisomic mice.** (A) Duration of treatment. NOR test results for Tg(*Dyrk1a*) mice treated with L41 or vehicle for 5, 12 or 19 days. Percentage object exploration by sniffing was determined for each object after a 24 h retention delay (familiar object, open symbol; novel object, filled symbol). NOR results of three Tg(*Dyrk1a*) cohorts treated with L41 for 5 (left), 12 (centre) or 19 (right) days. Tg(*Dyrk1a*) and Ts65Dn treated animals spent more time exploring the novel object compared with control mice, showing a rescue of their recognition memory. Left: 5 days treatment induced a NOR rescue in Tg(*Dyrk1a*) animals [wt: *n*=15, *P*<0.001; treated wt: *n*=15, *P*<0.001; untreated Tg(*Dyrk1a*): *n*=11, *P*=0.8; treated Tg(*Dyrk1a*): *n*=13, *P*=0.02]. Centre: a more consistent rescue was obtained after 12 days of L41 treatment [not treated wt: *n*=12, *P*<0.001; treated wt: *n*=15, *P*=0.11; untreated Tg(*Dyrk1a*): *n*=8, *P*=0.76; treated Tg(*Dyrk1a*): *n*=11, *P*<0.001]. Right: rescue obtained after 19 days of L41 treatment: the exploration was significantly different for wt (*n*=15, *P*<0.001), treated wt (*n*=15, *P*<0.001) and treated Tg(*Dyrk1a*) (*n*=15b, *P*<0.001) mice, but not for nontreated transgenic mice (*n*=13, *P*=0.64). (B) Treatment with L43 (left) and L99 (right). L99 treatment induced a cognitive rescue in the Tg(*Dyrk1a*) mice, whereas L43 treatment had no effect. L99 (right): [wt: *n*=10, *P*=0.02; treated wt: *n*=12, *P*=0.003; Tg(*Dyrk1a*): *n*=12, *P*=0.01; treated Tg(*Dyrk1a*): *n*=9, *P*<0.001]. L43 (left): [wt: *n*=12, *P*=0.008; treated wt: *n*=7, *P*=0.9; Tg(*Dyrk1a*): *n*=13, *P*=0.91; treated Tg(*Dyrk1a*): *n*=12, *P*=0.71]. (C) Ts65Dn and Dp1yey models. Left: in the Ts65Dn study, a significant statistical difference was observed for untreated wt (*n*=10, *P*<0.001), treated wt (*n*=8, *P*=0.009) and treated Ts65Dn (*n*=11, *P*=0.002), but not for untreated Ts65Dn animals (*n*=9, *P*=0.08). Right: L41 also normalizes the recognition memory of Dp1Yey mice (wt: *n*=13, *P*=0.04; treated wt: *n*=14, *P*=0.02; Dp1Yey: *n*=7, *P*=0.98; treated Dp1Yey: *n*=11, *P*=0.03). Data are represented as mean±s.e.m. with individual points per animal. Statistical analysis was performed with the two-way ANOVA test, Tukey post hoc. n.s., not significant. *P<0.05, **P<0.01, ***P<0.001.

These experiments were repeated (daily i.p. treatment for 19 days) with the kinase-inactive/CNR1-active L43 and the kinase-active/CNR1-inactive L99 leucettines (Fig. 2B). Results clearly showed the beneficial behavioural effects of L99 (Fig. 2B, right) and the lack of effects of L43 (Fig. 2B, left), demonstrating that the rescuing activity of leucettines derives from kinase inhibition rather than CNR1 antagonism.

We next ran the same experiments in Ts65Dn and Dp1Yey animals (Fig. 2C). Daily i.p. treatment with L41 for 19 days led to rescue in the NOR test. Intriguingly, L41 treatment had no restoring effect on working memory (Fig. S2A), nor on place memory in Tg(*Dyrk1a*) mice (Fig. S2B), as assessed in the Y-maze and place object location paradigms, respectively.

### L41 treatment has a global effect on brain functional connectivity measured by resting state fMRI

To noninvasively investigate whether DYRK1A kinase activity alters the brain functional connectivity (FC) and to reveal possible circuitry-based mechanisms underlying cognitive improvements induced by L41, we performed brain resting state fMRI (rsfMRI) experiments in vehicle or L41-treated Dp1Yey and wt mice. The brain connectivity patterns associated with default mode network (DMN) – the main functional circuitry describing the brain's intrinsic activity at rest (Raichle, 2015) – were mapped comparatively for each experimental group (Fig. 3A-a,b,B-a,b) via seed-based analysis. The seed used for generating DMN was the retrosplenial cortex (RSP), considered as the mouse DMN core area. DMN configuration obtained for the wt vehicle-treated group (Fig. 3A-a) served as a control pattern and encompassed the midline cortical areas [RSP, posterior parietal association areas (PTLp), temporal association areas, visual areas] as well as the rostral and medial anterior cingulate cortex (ACA) and hippocampal formation (HF) as previously described in mice (Sforazzini et al., 2014; Stafford et al., 2014). This DMN-like configuration was only minimally impacted by L41 treatment in wt animals (Fig. 3A-c,d), by decreasing the RSP connectivity with limited hippocampal (HF) areas.

**Fig. 3.**
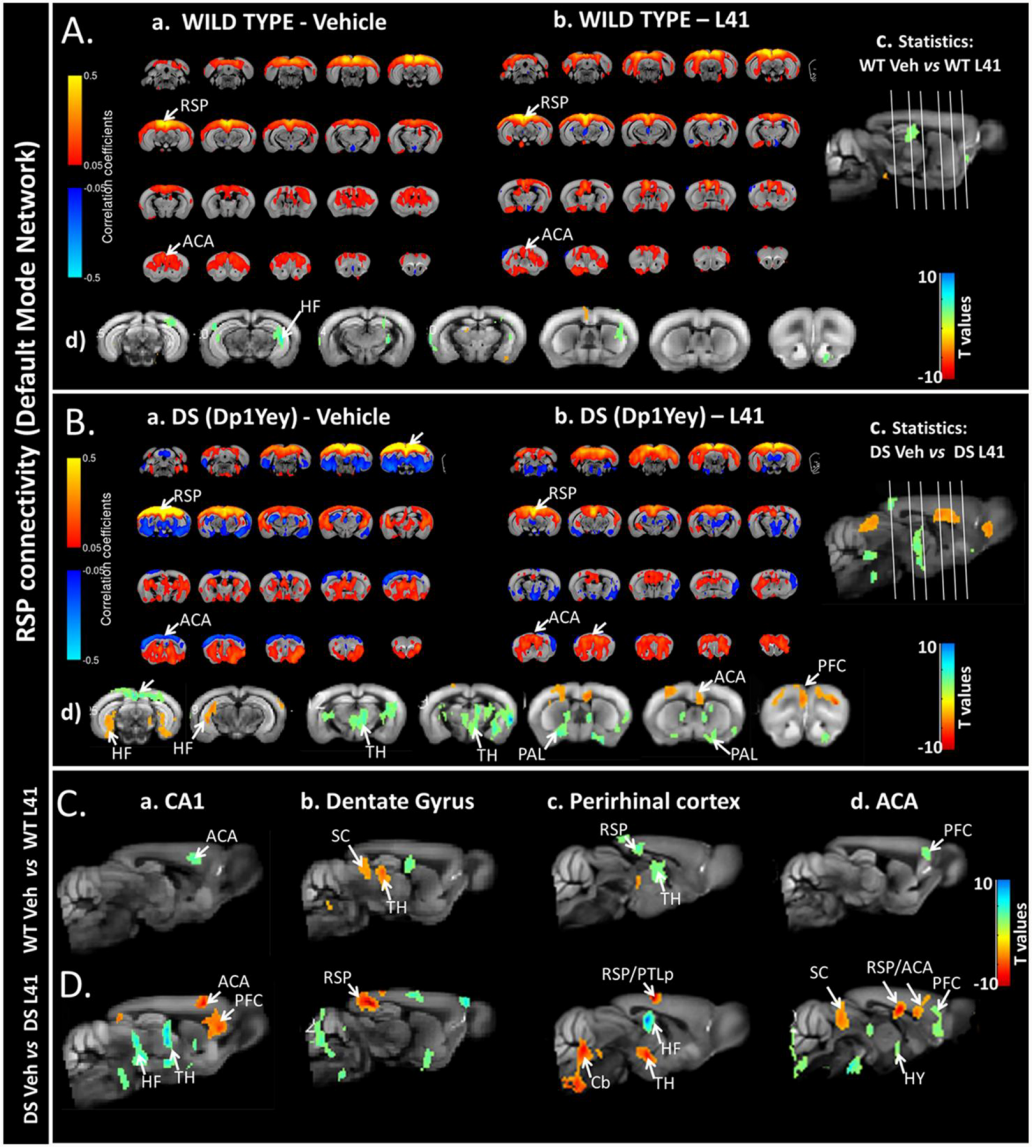
**Influence of L41 on mouse brain functional connectivity (FC) patterns mapped via rsfMRI.** (A,B) Default mode network (DMN) pattern in wt (a) and Dp1Yey (b) animals, mapped using the RSP cortex (core hub of DMN) as a seed region. A-a shows the typical DMN-like pattern observed in mice, spatially covering the middle rostrocaudal cortical axis of wt animals treated with vehicle, connecting the RSP and ACA. As shown in B-b, L41 treatment in wt animals slightly modifies the DMN patterns compared with wt-vehicle (see also statistics in A-c, sagittal view and A-d, coronal view; two-tailed Student's *t*-test, *P*<0.01). Red-orange scale quantifies the areas in which L41 treatment results in increased FC of wt-L41 compared with wt-vehicle. Blue-green scale indicates areas with decreased RSP connectivity after L41 treatment, compared with vehicle-treated wt mice. B-a demonstrates strongly altered DMN in vehicle-treated Dp1Yey compared with wt vehicle-treated animals (A-a, wt-vehicle), highlighting the pathological connectivity features of the mutant animals. As shown in B-b, L41 treatment in mutant Dp1Yey animals (DP16-L41) strongly modifies the DMN, restoring the positive correlations (red) of the RSP with the frontal brain areas (arrows, B-b). Voxel-wise statistics shown in B-c and B-d indicate, in red-orange, the areas in which L41 treatment results in increased FC of the DP16-L41 group compared with the DP16-vehicle group. Blue-green scale indicates areas with decreased RSP connectivity after L41 treatment, compared with the vehicle-treated group mutant mice. In A-a,b and B-a,b, red indicates the positively correlated areas (0.1 to 0.5 correlation coefficients); blue indicates negatively correlated areas (–0.1 to –0.5 correlation coefficients). (C,D) FC patterns in wt (C) and Dp1Yey (D) animals after L41 treatment: C-a, CA1 FC; C-b, dentate gyrus FC; C-c, perirhinal cortex FC; C-d, ACA FC (two-tailed Student's *t*-test, *P*<0.01). Red-orange shows the brain areas in which L41 treatment results in increased FC; blue-green indicates areas with decreased connectivity after L41 treatment, compared with vehicle-treated mice. ACA, rostral and medial anterior cingulate cortex; Cb, cerebellum; HF, hippocampal formation; HY, hypothalamus; PAL, pallidum; PFC, prefrontal cortex; PTLp, posterior parietal association areas; RSP, retrosplenial cortex; SC, superior colliculus; TH, thalamus.

In Dp1Yey mice, trisomy strongly influenced DMN architecture (Fig. 3B-a) by altering its pattern along midline cortical areas, highlighting the pathological features of Dp1Yey brain, as compared with wt brains (Fig. 3A-a). Notably, Dp1Yey mice show reversed connectivity features of RSP (the core area of DMN) towards the rostrofrontal cortical regions, including ACA [Fig. 3A-a versus B-a; switch from positive correlations (red/yellow scale) to negative correlations (blue scale)]. Intergroup statistics (vehicle-treated wt versus vehicle-treated Dp1Yey; Fig. S3) revealed diminished RSP-ACA connectivity in Dp1Yey animals compared with controls (Fig. S3A), while strengthening the local connectivity around the RSP seed (Fig. S3B). Concurrently, the RSP of Dp1Yey vehicle animals showed increased connectivity to limbic areas of basal forebrain [i.e. pallidum (PAL)] when compared with that of the wt vehicle group (Fig. S3B).

L41 treatment of Dp1Yey mice rescued this altered DMN pattern (Fig. 3B-b), prominently acting to significantly increase the FC of the RSP with the ACA, prefrontal cortex (PFC) and ventral HF (group statistics in Fig. 3B-d, orange/red) and to reduce FC with subcortical regions including the thalamus (TH) and PAL (Fig. 3B-d, green/blue).

To further reveal FC signatures of L41 action in Dp1Yey mice we evaluated the connectivity, across the whole brain, for several key brain areas involved in learning and memory [hippocampal CA1 and dentate gyrus (DG) areas, perirhinal cortex (PERI) and ACA]. Group statistical analysis of FC maps highlighted overall restricted effects of L41 on brain FC of wt animals (Fig. 3C-a-d) but robust L41-dependent brain FC modifications in the DS model (Fig. 3D-a-d). Acting at the hippocampal level, L41 treatment triggered robust changes in CA1 and DG connectivity in Dp1Yey mice (Fig. 3D-a,b). The CA1 strengthened its FC with the PFC and ACA (Fig. 3D-a, orange/red) and decreased its functional communication with the ventral HF (subiculum) and thalamic nuclei (Fig. 3D-a, green/blue). The strongest L41-triggered DG connectivity modifications were identified along the DG-RSP functional pathway in Dp1Yey mice (Fig. 3D-b). A divergent and limited effect of decreased CA1-ACA connectivity was measured in wt mice, after L41 treatment (Fig. 3C-a, green/blue), and the DG altered its connectivity towards the TH and superior colliculus (SC) in wt animals.

Furthermore, L41 treatment triggered remodelling of functional cross-talk between the PERI and the HF, RSP and PTLp in Dp1Yey animals (Fig. 3D-c), while acting primarily on PERI-TH connectivity in wt animals (Fig. 3C-c). Group statistics additionally revealed a selective impact of L41 on ACA connectivity in Dp1Yey mice (Fig. 3D-d), significantly modifying its patterns towards the PFC (decrease), RSP (increase), SC (increase) and hypothalamic (HY) areas (decrease). Meanwhile, L41 induced limited effects in wt mice, by decreasing ACA-PFC connectivity (Fig. 3C-d). Overall, these results indicate the potential of L41 to act at a circuitry level, modifying the global brain FC in Dp1Yey mice, which are strongly susceptible to its effects.

### Increased DYRK1A expression and catalytic activity in DS models: leucettines normalize DYRK1A activity

To validate the Tg(*Dyrk1a*) and Ts65Dn models in terms of DYRK1A expression and function, we first verified the expression levels of *Dyrk1a* mRNA (Fig. 4A) and DYRK1A protein (Fig. 4B) in brains derived from control or L41-treated animals (19 days, daily i.p.). Total mRNAs were extracted from brains and *Dyrk1a*, *Gsk-3b* and *Rplp0* mRNAs were quantified by quantitative polymerase chain reaction (qPCR) with specific primers. Results showed the expected ∼1.5-fold increase in *Dyrk1a* mRNA levels (normalized with respect to *Gsk-3b* and *Rplp0*) in both transgenic models compared with their wt littermates. L41 treatment for 19 days did not modify *Dyrk1a* mRNA levels (Fig. 4A). DYRK1A protein levels were also increased in transgenic mice models compared with control wt animals, as shown by western blotting (WB) of total brain proteins, whereas GSK-3α/β and β-actin levels remained at identical levels in transgenic and wt mice brains (Fig. 4B). L41 treatment had no effect on the expression of DYRK1A and GSK-3α/β. We next measured DYRK1A catalytic activities from transgenic and wt brain protein extracts (Fig. 4C). After 19 days of L41 or vehicle treatment, GSK-3α/β activity remained identical in the brains of transgenic and wt mice (data not shown), and was thus used to normalize the DYRK1A kinase activity. As expected, DYRK1A activity was elevated by ∼1.5- to 1.8-fold in transgenic brains compared with wt brains (Fig. 4C). L41 treatment did not reduce DYRK1A activity in wt mice brains, but reduced DYRK1A activity by ∼30% in the brains of Tg(*Dyrk1a*) and Ts65Dn animals, essentially down to the level of control counterparts. DYRK1A kinase activity was thus normalized by L41 treatment (Fig. 4C). In other words, although basal DYRK1A activity in trisomic and disomic mice brains was insensitive to L41, only excess DYRK1A activity in trisomic mice brains appeared to be sensitive to L41. To verify that all brain DYRK1A activity can, in principle, be inhibited by L41, DYRK1A was extracted and immunopurified from the brains of untreated wt and both transgenic animals. DYRK1A kinase activities were assayed *in vitro* in the presence of increasing concentrations of L41. Results showed that the DYRK1A of wt and transgenic animal brains can be almost fully inhibited *in vitro* with essentially identical dose-response curves (Fig. 4D).

**Fig. 4.**
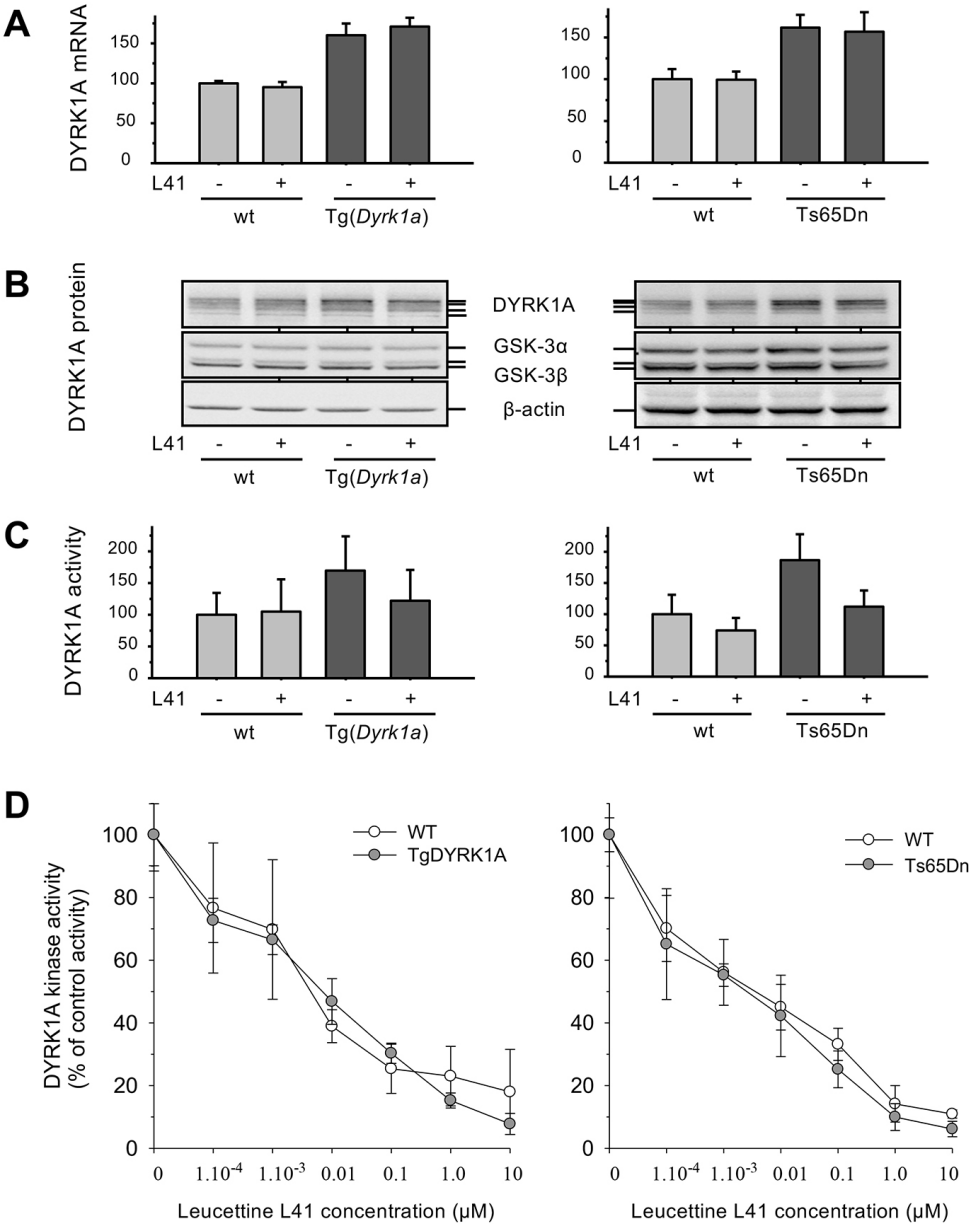
***Dyrk1a* mRNA and DYRK1A protein expression, and catalytic activity in Tg(*Dyrk1a*) and corresponding wt mice brains, and in Ts65Dn and corresponding wt mice brains.** (A) mRNA expression. Total RNA was extracted, purified and reverse transcribed into cDNA. mRNA expression of *Dyrk1a*, *Gsk-3b* and reference *Rplp0* was quantified by qPCR from the amplification of cDNA with specific primers (one primer annealing to an exon-exon junction). Results are presented as mean±s.e. of four to six measurements and are shown relative to *Rplp0* expression, normalized to wt *Gsk-3b* expression. (B) Protein expression. Total proteins were extracted, resolved by SDS-PAGE and analysed by WB using antibodies directed against DYRK1A, GSK-3α/β and actin (loading control). (C) DYRK1A catalytic activity. DYRK1A was purified from brain extracts by immunoprecipitation and GSK-3α/β was purified by affinity chromatography on axin-agarose beads. Activities of the purified kinases were assayed in triplicate in a radioactive kinase assay using specific peptide substrates, and are reported after normalization with wt GSK-3α/β activities (mean±s.e.). (D) *In vitro* DYRK1A kinase activity. The catalytic activity of DYRK1A immunoprecipitated from the brains of Tg(*Dyrk1a*) and Ts65Dn mice and their respective controls was assayed in the presence of a range of L41 concentrations.

DYRK1A activity was measured following immunoprecipitation (and normalization on the basis of GSK-3α/β activity measured in the same samples) from brain extracts of wt and Tg(*Dyrk1a*) animals treated daily for 5, 12 or 19 days (Fig. 5A-C) with L41, or for 19 days with kinase-inactive L43 (Fig. 5D). As expected, DYRK1A activity was increased in Tg(*Dyrk1a*) versus wt brains. Tg(*Dyrk1a*) brain DYRK1A activity was normalized after treatment with L41 for 12 and 19 days, but not after 5 days of L41 treatment, nor after 19 days of L43 treatment. These results correlate with L41-induced DYRK1A activity normalization (Fig. 5) and cognitive rescue (Fig. 2).

**Fig. 5.**
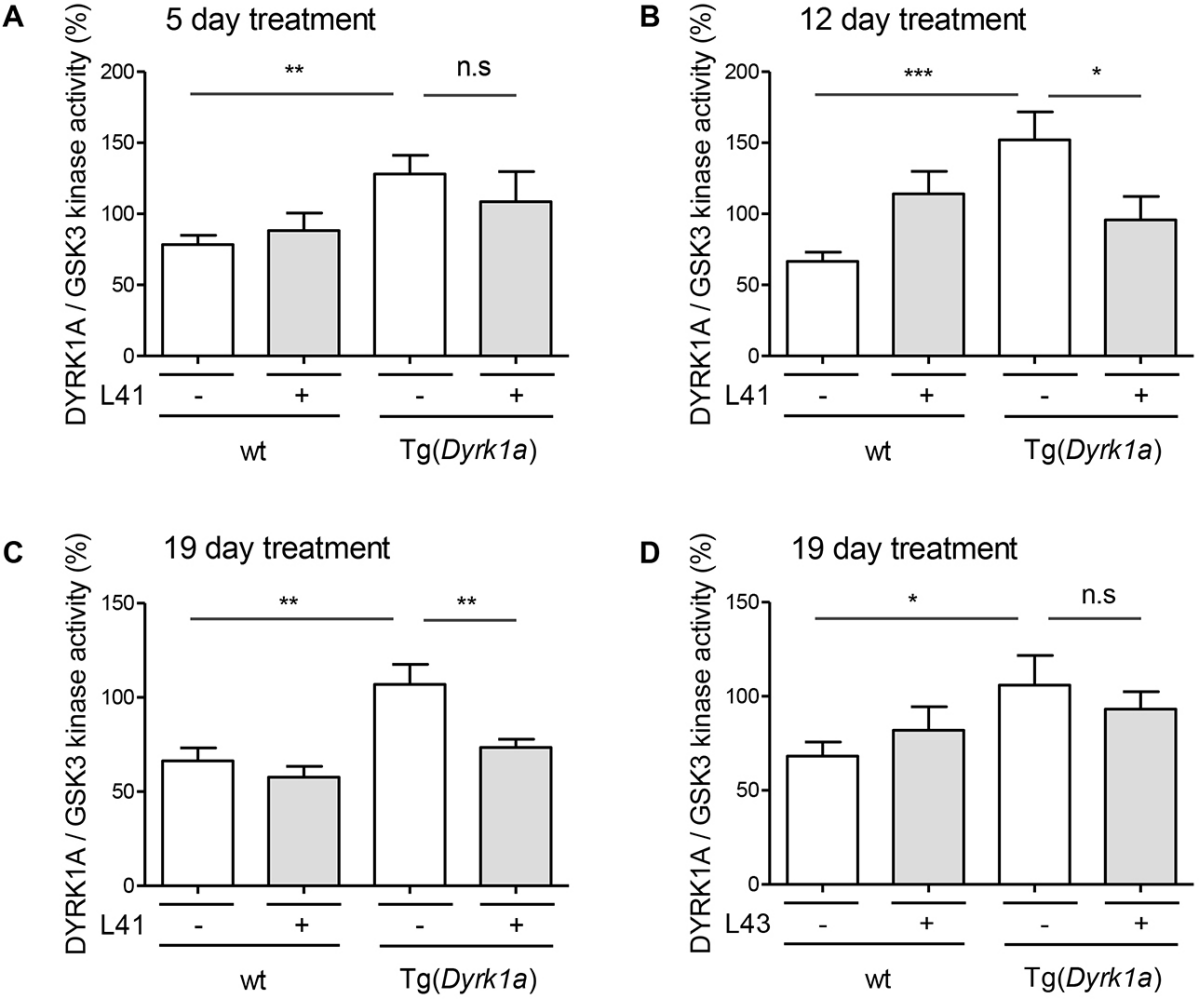
**Effects** **of** **L41 treatment duration and treatment with L43.** (A-D) Wt and Tg(*Dyrk1a*) mice were treated with L41 or vehicle for 5 (A), 12 (B) or 19 (C) days or L43 or vehicle for 19 days (D). Brains were recovered and extracted, and then DYRK1A and GSK-3α/β were immunopurified and affinity purified, respectively, and assayed for their catalytic activities. DYRK1A kinase activity was normalized with GSK-3α/β activities in each extract (mean±s.e.). DYRK1A inhibition in Tg(*Dyrk1a*) mice brains was not significant after 5 days of L41 treatment (*P*=0.42), but was increasingly significant after 12 (*P*=0.04) and 19 (*P*=0.01) days of L41 treatment. 19 days treatment with kinase-inactive L43 did not reduce DYRK1A activity in Tg(*Dyrk1a*) mice (*P*=0.5). n.s., not significant. *P<0.05, **P<0.01, ***P<0.001.

In all previous experiments, brains were collected 1 h after the last leucettine treatment. We wondered about the persistence of the effects of L41 after the last injection (Fig. 6). Tg(*Dyrk1a*) and wt animals were treated with L41/vehicle daily for 19 days. NOR tests were run and brains collected 24 h or 48 h after the last L41 treatment. DYRK1A catalytic activity was dosed in Tg(*Dyrk1a*) and wt mice brains. As expected, wt brain DYRK1A activity was insensitive to L41 treatment. Tg(*Dyrk1a*) brain DYRK1A activity was increased compared with control wt brain DYRK1A activity (Fig. 6A,C), and normalized to wt levels 24 h after the last L41 treatment (Fig. 6A). In contrast, L41 had no more effects 48 h after the last treatment (Fig. 6C). In terms of restoration of cognitive abilities, the NOR tests revealed that Tg(*Dyrk1a*) deficits were still corrected 24 h, but not 48 h, after the last L41 treatment (Fig. 6B,D). Because L41 is essentially undetectable in brain extracts 2 h after the acute i.p. injection, it might be protected from degradation once bound to DYRK1A or it could have been metabolized to an unidentified, stable active inhibitor.

**Fig. 6.**
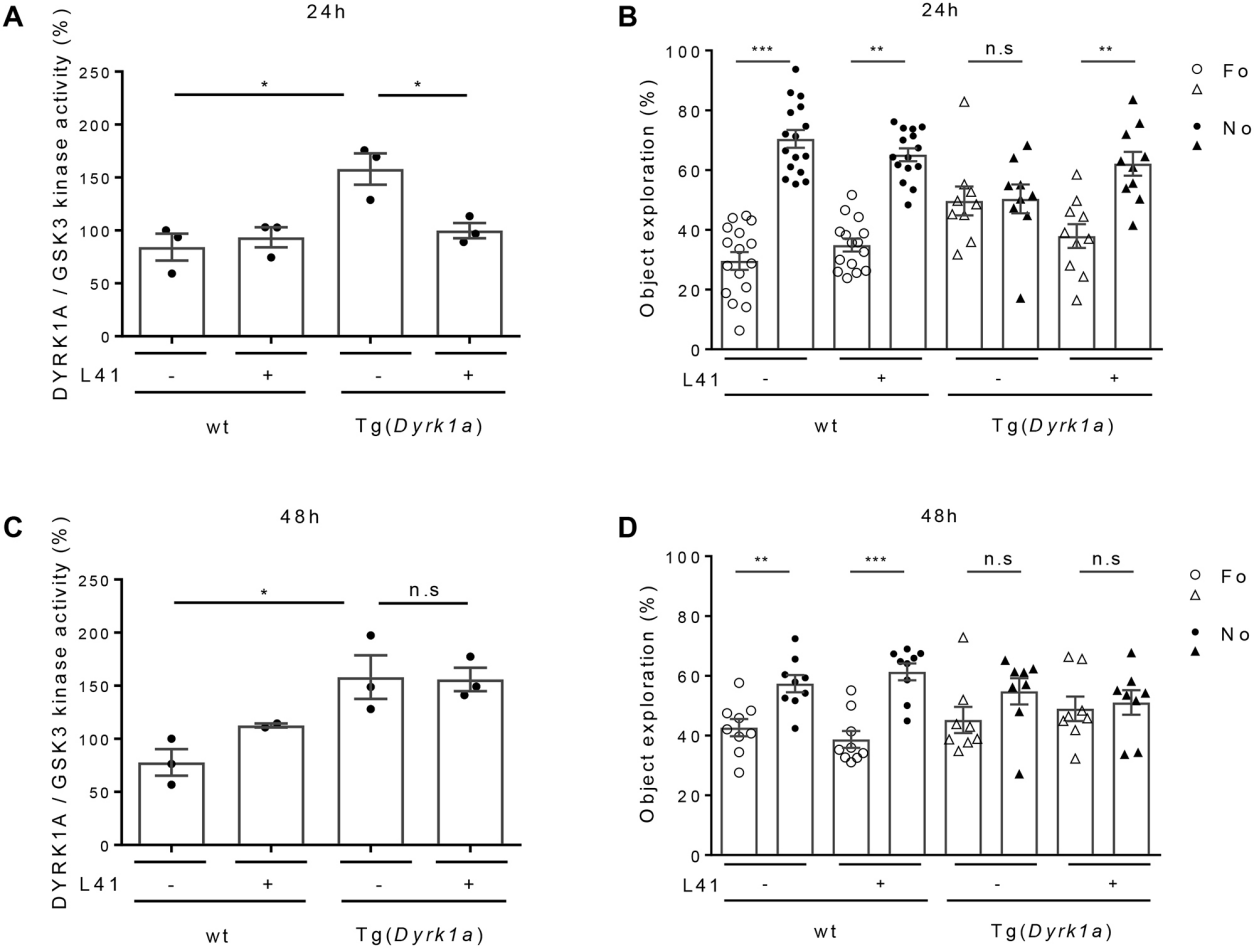
**Persistence of the L41 inhibitory effect on DYRK1A activity and rescue of NOR deficit.** (A,C) DYRK1A and GSK-3α/β activities were measured after purification from the brains of wt (*n*=3), L41-treated wt (*n*=3), Tg(*Dyrk1a*) (*n*=3) and L41-treated Tg(*Dyrk1a*) (*n*=3) animals, 24 h (A) or 48 h (C) following the end of a 19 day L41 treatment. After 24 h (*P*=0.02), but not at 48 h (*P*=0.93), the DYRK1A catalytic activity of the treated Tg(*Dyrk1a*) mice brains was normalized compared with that of nontreated animals. (B,D) NOR tests were performed 24 h (B) or 48 h (D) after the last day of the 19 day L41 treatment. Although the rescuing effect was detectable 24 h after the last L41 treatment (*P*=0.01), no rescue was seen after a 48 h delay (*P*=0.72). n.s., not significant. *P<0.05, **P<0.01, ***P<0.001.

### Overexpressed DYRK1A accumulates in cytoplasm and synapse: differential subcellular L41 distribution

We next investigated the subcellular distribution of DYRK1A in the brains of Tg(*Dyrk1a*) and wt animals (Fig. 7A,B). Brains were collected and cells dissociated and fractionated using two methods. The first allowed the separation of a cytosol+synaptosomes fraction from a nuclear fraction (Fig. 7A). The second separated a cytosol+nuclei fraction from a synaptosomal fraction (Fig. 7B). The purity of each fraction was evaluated by WB with specific markers: postsynaptic density protein 95 (PSD95; also known as DLG4) (cytosol+synaptosomes), histone H2B (nuclei), cyclin L1 (cytosol+nuclei), SYN1 and AMPA-selective glutamate receptor 1 (GLUR1; also known as GRIA1) (synaptosomal fraction) (Fig. 7A,B, top). DYRK1A expression levels were assessed following sodium dodecyl sulfate–polyacrylamide gel electrophoresis (SDS-PAGE) of the different cellular fractions, followed by WB, and normalization to the levels of β-actin (Fig. 7A,B, bottom). DYRK1A was detected in all fractions in both genotypes, but its expression was significantly higher (∼1.5-fold), in the cytosol and synaptosomes of Tg(*Dyrk1a*) brains compared with those of wt brains. No differences in nuclear DYRK1A expression were seen between transgenic and wt animals. Brain DYRK1A overdosage in Tg(*Dyrk1a*) animals thus occurs in the cytosol and synaptosomes, but not in the nuclei. We are currently exploring the reasons for this differential distribution of excess DYRK1A.

**Fig. 7.**
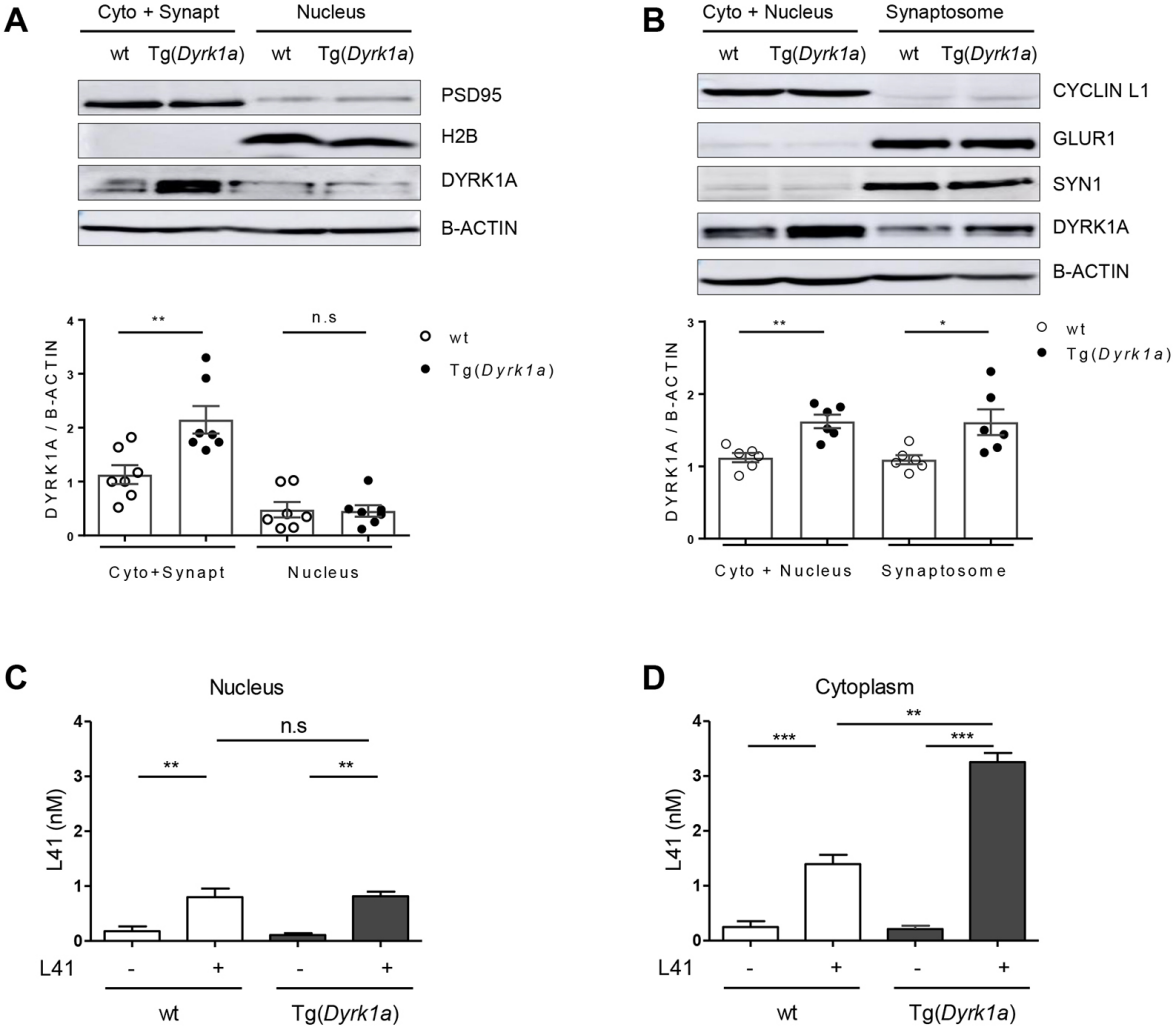
**DYRK1A and L41 subcellular localization.** (A,B) Wt or Tg(*Dyrk1a*) brains were fractionated by two methods and the expression of DYRKA was estimated by WB following SDS-PAGE. Reference subcompartment-specific proteins were detected by WB. (A) DYRK1A expression in cytoplasm+synaptosomes and in nuclear fractions [wt, *n*=6; Tg(*Dyrk1a*), *n*=6]. DYRK1A overexpression is observed in the cytoplasm+synaptosomes fraction (*P*=0.006), but not in the nuclear fraction (*P*=0.9). WB of specific markers validates the purity of fractions: PSD95 (95 kDa, cytoplasmic+synaptosomal marker), H2B (17 kDa, nuclear marker), β-actin (42 kDa, housekeeping protein). (B) DYRK1A expression in cytoplasm+nuclei and in synaptosomal fractions [wt, *n*=7; Tg(*Dyrk1a*), *n*=7]. DYRK1A was overexpressed in both cytoplasmic+nuclear (*P*=0.001) and synaptosomal (*P*=0.02) fractions. Fractionation was confirmed by WB of specific compartment markers: cyclin L1 (55 kDa, cytoplasmic+nuclear marker), GLUR1 (100 kDa, postsynaptic marker), SYN1 (74 kDa, presynaptic marker), β-actin (42 kDa, housekeeping protein). (C,D) L41 subcellular levels. L41 was more highly detected in the brain nuclear (C) and cytoplasmic (D) compartments in L41-treated wt (*n*=5) and Tg(*Dyrk1a*) (*n*=2) mice compared with nontreated wt (*n*=5) and nontreated Tg(*Dyrk1a*) (*n*=5) mice. L41 distribution was not significantly different between the brain nuclear fractions of treated wt and Tg mice. In contrast, the L41 level was increased in the cytoplasm of treated Tg mice brain compared with the cytoplasm of control wt mice brain (*P*=0.002). n.s., not significant. *P<0.05, **P<0.01, ***P<0.001.

We next measured L41 levels in nuclear and cytoplasmic fractions prepared from the brains of Tg(*Dyrk1a*) and wt animals which had been i.p. injected daily for 19 days with L41 (20 mg/kg) or vehicle (Fig. 7C,D). At the end of the treatments, brains were recovered and processed for L41 extraction and quantification by isobaric stable isotope chemical labelling, offline hydrophilic interaction chromatography (HILIC), followed by ultra-high precision liquid chromatography with electrospray ionization mass spectrometry (LC–MS). Results show essentially undetectable L41 in vehicle-treated animals, identical L41 levels in the brain nuclear fractions of Tg(*Dyrk1a*) and wt animals (Fig. 7C), and a significantly increased L41 level in the cytoplasmic fraction of Tg(*Dyrk1a*) brains compared with the cytoplasmic fraction of wt animals' brains (Fig. 7D). Thus, DYRK1A overexpression in the transgenic animals' brains appears to be limited to the cytoplasmic fraction, corresponding to the subcellular distribution of overexpressed DYRK1A (Fig. 7A,B). Accordingly, more L41 is detected in the cytoplasmic fraction from transgenic animals compared with their control littermates.

### Phosphoproteomic effects of DYRK1A trisomy and L41 treatment reveal key synaptic and cytoskeletal components

To explore the mechanisms underlying the correcting effects of L41 on NOR cognitive deficits of transgenic models, we analysed the phosphoproteome of proteins isolated from the hippocampus, cortex and cerebellum of both Tg(*Dyrk1a*) and Ts65Dn models, along with their respective wt counterparts, and following treatment with vehicle or L41 (20 mg/kg, daily i.p. injection for 19 days) (Fig. 8). All tissue samples were processed for phosphoproteomics analysis based on the enrichment and separation of proteotypic phosphopeptides with HILIC (see Materials and Methods). In Tg(*Dyrk1a*) and Ts65Dn mice, the hippocampus, cortex and cerebellum yielded 1384, 1523 and 2004 peptides, respectively, corresponding to 886, 948 and 1229 proteins (Table 1; Tables S1-S13).

**Fig. 8.**
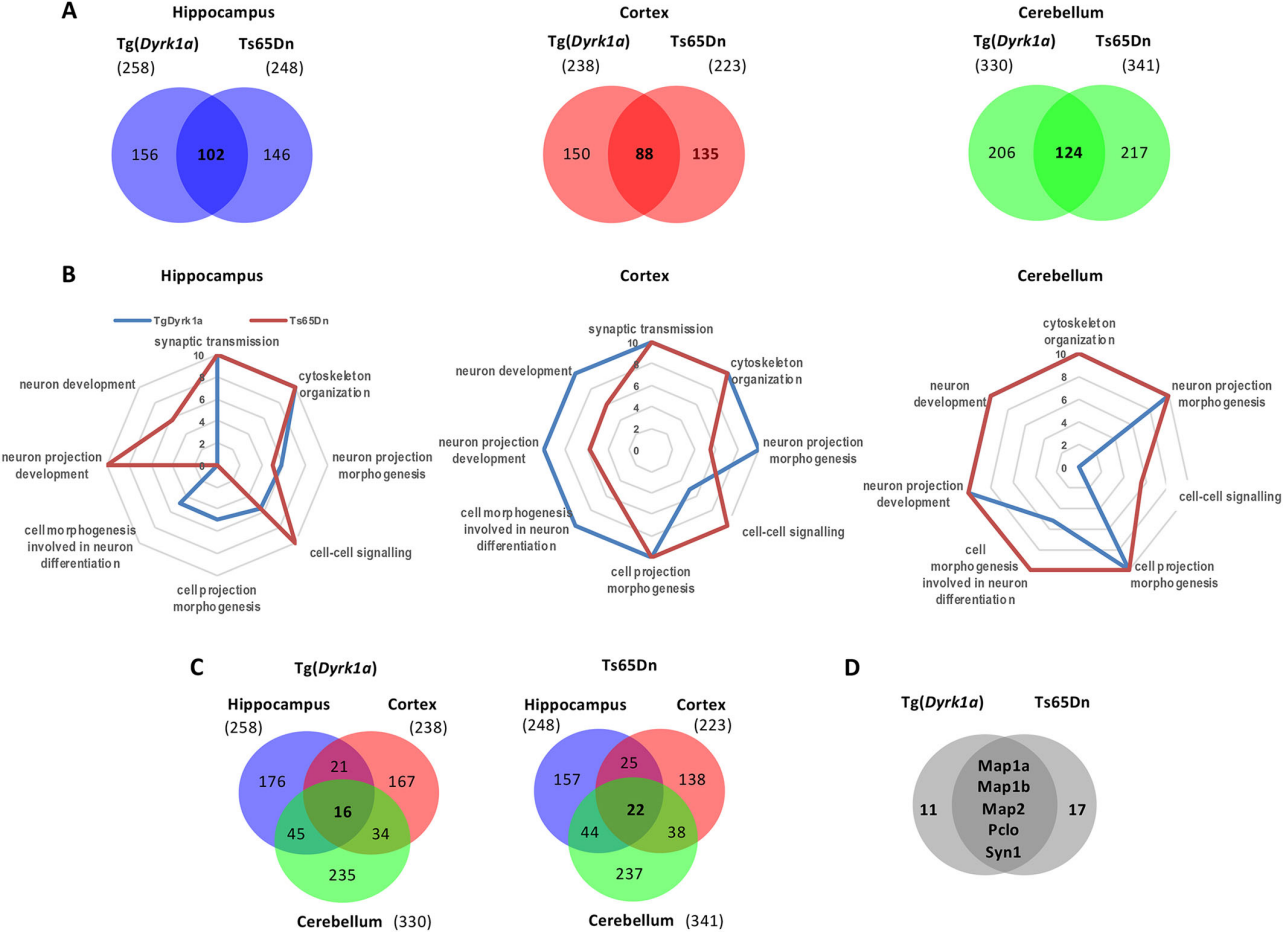
**Phosphoproteomic analysis of Tg(*Dyrk1a*) and Ts65Dn mice brains following exposure to L41.** Phosphoproteins, in each brain substructure, that are both up- or downregulated by trisomy and, respectively, down- and upregulated by L41 treatment were selected for analysis. (A) Venn diagrams comparing the two transgenic models versus wt and L41 treatment, at tissue level. 102, 88 and 124 modified phosphoproteins were common to the two models in the hippocampus, cortex and cerebellum, respectively. Numbers in parentheses indicate dual modulated phosphoproteins in each model and each tissue. (B) Biological processes enrichment deregulated by the phosphoproteins which are modulated one way in both Tg(*Dyrk1a*) and Ts65Dn mice and affected by L41 treatment in the opposite way. Represented here are those common to both models and to the three brain tissues. DAVID and ToppCluster analyses were performed in the hippocampus, cortex and cerebellum separately. Enriched classification is determined by the –log(*P*-value). Synaptic transmission, common to the hippocampus and cortex, and cytoskeleton organization, common to the three brain regions, are the processes most modified by trisomy and sensitive to L41. (C) Venn diagrams illustrate the number of dually modulated phosphoproteins in each model and in each tissue, and the numbers shared by different brain areas. 16 and 22 proteins were shared by all three tissues in Tg(*Dyrk1a*) and Ts65Dn mice, respectively. (D) Venn diagram comparison of these 16 and 22 phosphoproteins revealed that five are shared by both models.

**Table 1.**
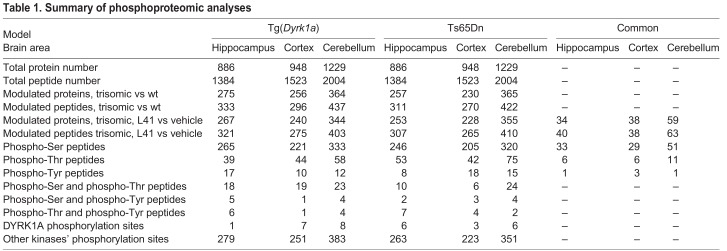
**Summary of phosphoproteomic** **analyses**

Among the peptides/proteins detected in this study, only 30% of the proteins and 20% of the peptides were significantly up- or downregulated in trisomic versus wt animals. Most peptides (80%) were phosphorylated on serine residues, whereas phosphorylation on threonine (15%) or tyrosine residues (5%) was less frequent. Few peptides (less than 5%) were phosphorylated on two amino acids. Very few phosphopeptides displayed the consensus DYRK1A phosphorylation sequence [R-P-x(1,3)-S/T-P] and most phosphopeptides were predicted to be phosphorylated by kinases from the CMGC (MAPK or GSK-3 protein) or AGC [MTOR or PKG (also known as PRKG1)] groups (data obtained with the PhosphoRS algorithm within the Proteome Discoverer software tool, version 1.4).

We selected the phosphopeptides displaying a trisomy-associated modulation (up- or downregulation) which was reverted by L41 treatment (down- or upregulation) (Fig. 8). These analyses were first run in each brain tissue and in each of the two models and their wt controls. We thus focused on proteins displaying an L41-reversible, trisomy-associated phosphorylation modulation. Based on these two criteria, 258 and 248 phosphoproteins were selected from Tg(*Dyrk1a*) and Ts65Dn hippocampus (Fig. 8A), respectively. Similarly, the Tg(*Dyrk1a*) and Ts65Dn cortex showed 238 and 223 dysregulated phosphoproteins, respectively (Fig. 8A). We found that 330 and 341 phosphoproteins in Tg(*Dyrk1a*) and Ts65Dn cerebellum, respectively, were altered by trisomy and L41 treatment (Fig. 8A). Among these phosphoproteins, 102, 88 and 124 were common to both transgenic models in the hippocampus, cortex and cerebellum, respectively (Tables S1-S12). These shared phosphoproteins were selected for DAVID cluster analysis (Tables S10-S12), which unravelled enrichment in synaptic, cytoskeletal and learning pathways (Fig. 8B; Fig. S4). ToppCluster analysis of the modulated phosphoproteins in each model and each brain region confirmed enrichment in synaptic transmission common to both models in the hippocampus and cortex, while cytoskeleton organization was enriched in both models for all three brain regions (Fig. 8B; Tables S11-S13).

We also compared, in each model, the phosphoproteins subsets of all three brain areas (Fig. 8C). In Tg(*Dyrk1a*), only 16 phosphoproteins were commonly modulated in the three brain substructures (Fig. 8C, left), while only 22 responded to these criteria in Ts65Dn (Fig. 8C, centre). Among these 16 and 22 phosphoproteins shared by the three brain regions, only five were common to both DS models (Fig. 8D): the microtubule-associated proteins MAP1A, MAP1B and MAP2, and presynaptic components piccolo (PCLO) and SYN1. All phosphosites modulated by both trisomy and L41 treatment, for each of the five proteins, are schematized in Fig. S5. They illustrate the complexity of the phosphoproteomics consequences of a single gene trisomy [Tg(*Dyrk1a*)] or a partial chromosome 16 trisomy (Ts65Dn) and the complexity resulting from the treatment with a single pharmacological agent. Among these five proteins, we looked for the residues with increased phosphorylation when DYRK1A was overexpressed, and with reduced phosphorylation when DYRK1A was inhibited by L41, and also matching the consensus DYRK1A phosphorylation sequence (Himpel et al., 2000). Based on these criteria, serine 551 of SYN1 was selected for further study.

### DYRK1A interacts with SYN1 and other proteins implicated in synaptic functions

To investigate potential interactions between DYRK1A and SYN1, co-immunoprecipitation (co-IP) experiments were carried out with adult mouse brain lysates (Fig. 9A) using antibodies directed against SYN1 or DYRK1A (negative control, GAPDH). As expected, DYRK1A and SYN1 were found in their respective immunoprecipitates (IPs). SYN1 was detected in DYRK1A IPs and DYRK1A was detected in SYN1 IPs (Fig. 9A), suggesting that these proteins form a direct or indirect complex in brain extracts. Calmodulin-dependent kinase 2A (CAMK2A) was present in SYN1 IPs, as expected from previous results (Llinás et al., 1985; Benfenati et al., 1992) and from its role in presynaptic vesicle pool release (Cesca et al., 2010). CAMK2A was also detected in DYRK1A IPs, suggesting the possibility of a DYRK1A/SYN1/CAMK2A complex, although separate DYRK1A/CAMK2A and SYN1/CAMK2A complexes are possible.

**Fig. 9.**
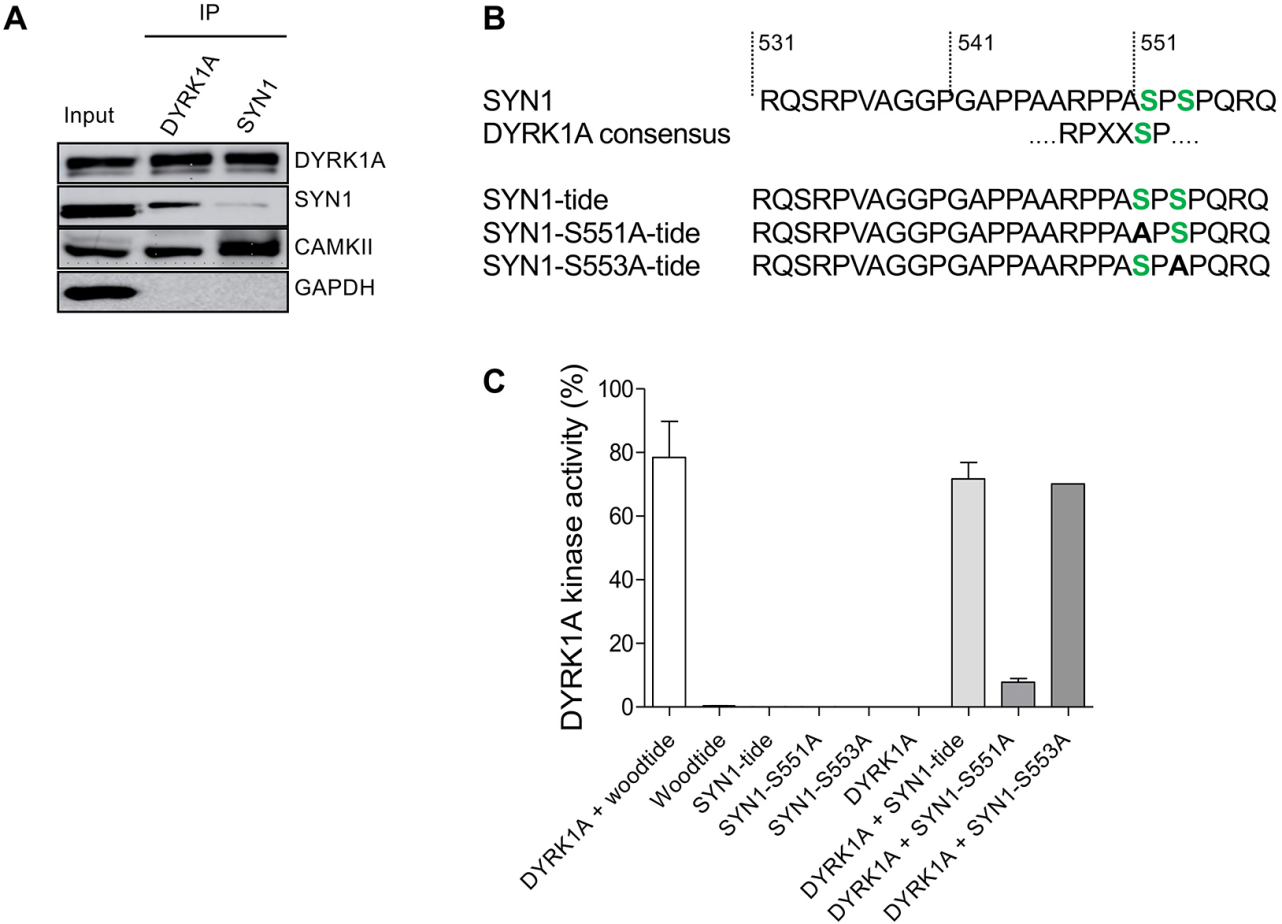
**Direct interaction of DYRK1A and SYN1, phosphorylation of SYN1 by DYRK1A.** (A) DYRK1A and SYN1 were immunoblotted following immunoprecipitation from wt mice brain extracts. DYRK1A or SYN1 present in the starting material (Input) were recovered in the IPs. SYN1 (74 kDa) was present in the DYRK1A IP and DYRK1A (85 kDa) was detected in the SYN1 IP, suggesting that these two proteins interact directly. Positive control of the SYN1 IP was performed using an anti-CAMKII antibody. As expected, CAMKII (50 kDa) was present in the SYN1 IP. DYRK1A IP also brought down CAMKII, suggesting complexes between SYN1, CAMKII and DYRK1A. (B) Sequence of SYN1 in the vicinity of Ser551 matches with the consensus DYRK1A phosphorylation site. Based on this sequence, three peptides were synthesized and used as potential substrates: SYN1, SYN1-S551A and SYN1-S553A. (C) Kinase activity of recombinant DYRK1A towards the three different SYN1peptides. SYN1 and SYN1-S553A peptides were phosphorylated at the same level as Woodtide by recombinant DYRK1A (71.7%±5.2%, 70.1% and 78.4%±11.4%, respectively). No significant catalytic activity was measured with the SYN1-S551A peptide (7.9%±1.2%).

To see whether DYRK1A directly phosphorylates SYN1, we ran *in vitro* kinase assays using recombinant DYRK1A and various SYN1-derived peptides, including Ser551, as potential substrates, or Woodtide as a reference substrate (Fig. 9B,C). Recombinant DYRK1A displayed similar activity towards SYN1-tide or SYN1-S553A-tide compared with Woodtide. In contrast, no significant phosphorylation could be measured with the SYN1-S551A peptide. This confirms that DYRK1A is able to phosphorylate SYN1 on its S551 residue, but not on the nearby Ser553 site. The Ser551 site matches with the consensus DYRK1A phosphorylation site (Fig. 9B).

## DISCUSSION

### Rescue of cognitive deficits by pharmacological inhibition of excess DYRK1A

In this study, we show that trisomy is associated with an increase in DYRK1A expression and catalytic activity, and that a class of synthetic DYRK1A inhibitors, the leucettines, exemplified by L41, is able to cross the blood brain barrier and selectively inhibit the excess DYRK1A linked to trisomy. Why only this fraction of overexpressed DYRK1A is inhibited, and most native, basal DYRK1A is not, remains a mystery. This effect could be linked to the accumulation of excess DYRK1A and L41 in specific cellular compartments and not in others, as shown in Fig. 7. Intriguingly, a similar sensitivity to inhibition of excess DYRK1A compared with ‘normal’ DYRK1A was observed with EGCG. This finding is encouraging in terms of potential therapeutic implications, as complete inhibition of DYRK1A is not desired. Intracellular DYRK1A has been described in both nuclear and cytoplasm compartments (Martí et al., 2003). Our results indicate that it is also present in synaptosomes, which might have consequences on the regulation of synaptic vesicles trafficking (see below).

We here demonstrate the rescuing effect of synthetic DYRK1A inhibitors, leucettines L41 and L99, on deficient recognition memory of three different trisomic mouse models with increasing genetic complexity, Tg(*Dyrk1a*), Ts65Dn and Dp1Yey. These beneficial behavioural effects directly correlate with inhibition of excess DYRK1A activity. There is also a strong coincidence with the duration of the drug treatment (Figs 2A and 5), the potency of the leucettine analogues (Figs 2B and 5) and the duration of the drug-free period following the last injection (Fig. 6). Finally, behavioural correcting benefits detected in the NOR test (Figs 2 and 6) correlate with remodelling of brain functional connectivity detected by fMRI (Fig. 3). However, we observed that working and spatial memories impaired in the Tg(*Dyrk1a*) mice were insensitive to L41 treatment, as assessed in the Y-maze and place object location tasks, respectively. This indicates a specific action of DYRK1A inhibition on molecular pathways specifically related to recognition memory. Our findings further strengthen the essential role of DYRK1A in intellectual phenotypes associated with DS. Leucettine derivatives should thus be investigated further as drug candidates to improve cognitive functions of DS patients.

### L41 treatment in DYRK1A-overexpressing mice triggers remodelling of brain FC pathways

Brain rsfMRI in Dp1Yey mice revealed global resilience of functional cerebral circuitry after L41 administration. Notably, L41 corrected the abnormal DMN patterns found in Dp1Yey mice, but also acted on connectivity of key brain areas associated with cognitive and memory processing (PFC, ACA, PERI, HF). DMN (Raichle, 2015) – previously described as a highly active circuitry during rest – and preserved across species (Stafford et al., 2014), was shown to be vulnerable to various neuropathological conditions (Hawellek et al., 2011; Raichle, 2015; Zhou et al., 2017), including DS (Anderson et al., 2013; Pujol et al., 2015). The core area of this network in mice is the RSP (associated with the posterior cingulate/precuneus cortex in humans) (Hübner et al., 2017; Sforazzini et al., 2014). Our analysis unravelled increased local connectivity around the RSP in DS mice, but strongly reduced long-range communication with frontocortical brain regions (ACA, PFC; Fig. S3), when compared with wt animals. This short-range stronger connectivity is not limited to the RSP, but represented a common feature for other investigated brain regions (ACA, PERI, HF) of Dp1Yey mice. Such a pattern of increased local, short-range brain communication was described as a cardinal feature of FC in DS patients (Anderson et al., 2013; Pujol et al., 2015; Vega et al., 2015). Indeed, DS human brains are characterized by simplified network structure, organized by local connectivity (Anderson et al., 2013; Pujol et al., 2015; Vega et al., 2015) and impaired efficiency to integrate information from distant connections.

Dp1Yey mouse brains additionally displayed features of higher negative functional correlations as compared with the wt vehicle group and, more obviously, a reversed correlation pattern (switch from positive to negative correlations) between the RSP and frontal cortical areas in the DS model. This feature, attenuated or corrected following L41 treatment, could eventually be discussed in the context of L41 regulation of inhibition/excitation ratios, imbalanced in DYRK1A-overexpressing mice (Souchet et al., 2014). Indeed, increased number and signal intensity from neurons expressing GAD67 (also known as GAD1), an enzyme that synthesizes GABA, indicating inhibition pathway alterations, was quantified in three different DS models (Souchet et al., 2014), including Dp1Yey. Pharmacological correction of inhibition/excitation was achieved in the Tg(*Dyrk1a*) DS mouse model (Souchet et al., 2015) by EGCG treatment. We can speculate on a similar effect of L41 on inhibition/excitation balance, and subsequent modulation of brain connectivity. Nevertheless, the brain synchrony modifications after L41 inhibition of excess DYRK1A activity in DS models might potentially reflect other molecular mechanisms and interactions at the synaptic and cytoskeletal level, as shown here, and subsequently underpin correction of cognitive and memory deficits of DS mice. Importantly, L41 had only limited effects on FC in wt animals, whereas in the Dp1Yey model it largely impacted the connectivity features, on distributed action sites, that coincide with alterations reported for brain anatomy in DS models, most notably, frontal and prefrontal cortical areas (ACA/PFA), the HF, PAL and TH. Volumetric MRI in DS mouse models, showed a general trend for smaller frontal lobes, hippocampal and cerebellar regions, but larger thalamic and hypothalamic areas (Powell et al., 2016; Roubertoux et al., 2017). Diffusion MRI also identified potential microstructural alterations in the above-mentioned areas and also the striatum (including the PAL) (Nie et al., 2015). Our rsfMRI study advances the current knowledge on the brain functional communication in DS mouse models, revealing targeted and effective action of L41 on brain circuitry, consistent with the profile of cognitive and novel object recognition memory improvements.

### DYRK1A and SYN1

Phosphoproteomic analyses using ultra-high precision LC–MS analysis unravelled several clusters of neuronal phosphorylated proteins directly controlled by DYRK1A or clusters indirectly modulated in the trisomic condition and sensitive to L41 treatment. Five phosphoproteins were shared by Tg(*Dyrk1a*) and Ts65Dn mice and were present in three brain substructures (hippocampus, cortex, cerebellum) (Fig. 8). Furthermore, these phosphoproteins showed significant modulation in their phosphorylation levels in trisomic versus disomic animals and these modulations were sensitive, in the opposite direction, to L41 treatment. A few key pathways, including controlling synaptic vesicle (SV) transport, calcineurin NFAT signalling and cytoskeleton organization, were found to be directly affected by DYRK1A, or as a consequence of its kinase activity (Fig. S4), while others might represent indirect effects of the overdosage. Nevertheless, the immune response was found to be affected, correlating with several studies linking DYRK1A to inflammation. We here focused on SYN1 as it was the only protein that revealed a serine residue corresponding to the DYRK1A phosphorylation consensus sequence. SYN1 Ser551 was hyperphosphorylated following DYRK1A overexpression and dephosphorylated following L41 treatment. Representative annotated ultra-high resolution product ion spectrum of proteotypic peptide qSRPVAGGPGAPPAARPPAsPSPqR encoding the phosphorylated residue Ser551 is shown in Fig. S6.

Co-IP experiments showed that DYRK1A interacts, either directly or indirectly, with SYN1 (Fig. 9). SYN1 has been described to be involved in the reserve SV pool maintenance at the presynaptic bouton by tethering SVs to the actin cytoskeleton (Hilfiker et al., 2005; Benfenati et al., 1991). Phosphorylation of SYN1 by CAMKII leads to the release of SVs and allows them to move close to the active zone (Llinás et al., 1991). Neurotransmitter release at the active zone is thus strongly dependent on SYN1 phosphorylation. We showed that SYN1 was phosphorylated by DYRK1A on its S551 residue *in vitro* and *in vivo*, thus highlighting a novel role of DYRK1A in SYN1-dependent presynaptic vesicle trafficking. Besides its physiological role in synaptic plasticity regulation, SYN1 has been associated with epilepsy (Garcia et al., 2004; Fassio et al., 2011). Mutations in the phosphorylation domains of SYN1 essential for vesicle recycling control have been related to epilepsy (Fassio et al., 2011). In addition, mental retardation, autosomal dominant 7 (MRD7) patients with *Dyrk1a* haploinsufficiency display epilepsy seizures (Courcet et al., 2012; Møller et al., 2008; Oegema et al., 2010; Valetto et al., 2012; Yamamoto et al., 2011). Our results suggest that epileptic seizures observed in MRD7 patients could be induced by defects in SYN1 regulation.

### DYRK1A, microtubule-binding proteins, PCLO and other synaptic targets

The other four proteins found in our phosphoproteomics study will be the object of another study but are briefly reviewed here. The detection of MAP1A, MAP1B and MAP2, all previously reported as DYRK1A substrates (Murakami et al., 2012; Scales et al., 2009), validated the power of our analysis and confirmed the role of DYRK1A in dendrite morphogenesis and microtubule regulation (Ori-McKenney et al., 2016). The last phosphoprotein, PCLO, is a cytoskeletal matrix protein associated with the presynaptic active zone (Cases-Langhoff et al., 1996), which acts as a scaffolding protein implicated in SV endocytosis and exocytosis (Garner et al., 2000; Fenster et al., 2003). The lack of PCLO in the human brain leads to a dramatic neuronal loss associated with pontocerebellar hypoplasia type III (Ahmed et al., 2015). Moreover, PCLO knockdown in cultured hippocampal neurons increases SYN1 dispersion out of the presynaptic terminal and SV exocytosis (Leal-Ortiz et al., 2008). It has been shown that PCLO modulates neurotransmitter release by regulating F-actin assembly (Waites et al., 2011). It clearly appears that PCLO acts upstream of SYN1 and regulates its role in vesicle recycling.

Taken together, our findings reveal SYN1 as a new direct substrate of DYRK1A, suggesting a novel role of this kinase in the regulation of SV release at the presynaptic terminal. Moreover, the relatively safe and selective DYRK1A inhibitors, the leucettines, successfully correct recognition memory deficits associated with DS in three different mice models. Although the DYRK1A-dependent biological process which is rescued by these drugs still needs to be elucidated, leucettines and their analogues represent promising therapeutic drugs to enhance cognitive functions in DS patients.

### DYRK1A, DS and AD

There is strong support for the involvement of DYRK1A in cognitive deficits associated with Alzheimer's disease (AD): (1) *DYRK1A* mRNA (Kimura et al., 2007) and DYRK1A protein (Ferrer et al., 2005) levels are increased in postmortem human AD brains compared with healthy brains; (2) calpain 1-induced cleavage of DYRK1A is observed in AD brains and associated with increased activity (Jin et al., 2015); (3) DYRK1A phosphorylates key AD players, such as amyloid precursor protein (Ryoo et al., 2008), presenilin 1 (Ryu et al., 2010), Tau (also known as MAPT) (Woods et al., 2001; Ryoo et al., 2007; Azorsa et al., 2010; Coutadeur et al., 2015; Jin et al., 2015), septin (Sitz et al., 2008) and neprylysin (Kawakubo et al., 2017); (4) DYRK1A regulates splicing of Tau mRNA (Shi et al., 2008; Wegiel et al., 2011; Yin et al., 2012, 2017; Jin et al., 2015); (5) DYRK1A inhibition corrects cognitive defects in 3xTG-AD (Branca et al., 2017), APP/PS1 (B. Souchet, unpublished) and Aβ25-35 peptide-injected wt mice (Naert et al., 2015), three widely used mice models of AD. These facts provide additional incentive to investigate the regulation and substrates of brain DYRK1A and to develop potent and selective DYRK1A inhibitors to treat cognitive deficits observed in different indications. DS patients display early symptoms of AD and show a high frequency of dementia at later age (Ballard et al., 2016). The triplication of APP located on the HSA21 is thought to contribute to amyloid plaques and neurofibrillary tangles, two causative factors in AD, that accumulate early in 30- to 40-year-old DS people (Head, et al., 2012a). These factors, associated with neuroinflammation and oxidative damage also diagnosed in both AD and DS individuals, lead to precocious dementia observed from age 30 to 39 (Head et al., 2012b). Studying DS will have an impact on the understanding of AD and, reciprocally, DYRK1A is clearly a common factor between the two diseases.

## MATERIALS AND METHODS

### Animal models, treatment and behaviour assessment

Tg(*Dyrk1a*) mutant mice and Dp1Yey models were maintained on the C57BL/6J genetic background. Ts(17^16^)65Dn trisomic mice were obtained from The Jackson Laboratory and kept on the C57BL/6J×C3B, a congenic sighted line for the BALB/c allele at the *Pde6b* locus (Hoelter et al., 2008). The local ethics committee, Com'Eth (no. 17), approved all mouse experimental procedures, under the accreditation number APAFIS #5331 and #3473, with Y.H. as the principal investigator.

Behavioural studies were conducted in 12- to 20-week-old male animals. All assessments were scored blind to genotype and treatment, as recommended by the ARRIVE guidelines (Karp et al., 2015; Kilkenny et al., 2010). Leucettine L41 was prepared at 40 mg/ml in dimethyl sulfoxide (DMSO), aliquoted and stored below −20°C. The final formulation was prepared just prior to use as a 2 mg/ml solution diluted in PEG300/water (50/45), to reach a final DMSO/PEG300/water 5/50/45 (v/v/v) mix. Treated animals received a daily dose (5, 12 or 19 days) of this formulation by i.p. injection of 20 mg/kg/day. Nontreated animals received the same formulation without L41.

The NOR task is based on the innate tendency of rodents to differentially explore a novel object over a familiar one (Ennaceur and Delacour, 1988). Day 1 was a habituation session. Mice freely explored the apparatus, a white circular arena (53 cm diameter) placed in a dimly lit testing room (40 lux). On day 2, the acquisition phase, mice were free to explore two identical objects for 10 min. Mice were then returned to their home cage for a 24 h retention interval. To test their memory, on day 3, one familiar object (already explored during the acquisition phase) and one novel object were placed in the apparatus and mice were free to explore the two objects for a 10 min period. Between trials and subjects, the different objects were cleaned with 50° ethanol to reduce olfactory cues. To avoid a preference for one of the objects, the new object was different for different animal groups and counterbalanced between genotype and treatment as well as for location of novel and familiar objects (left or right). Object exploration was manually scored and defined as the orientation of the nose to the object at a distance <1 cm. For the retention phase, the percent of time spent exploring familiar versus novel objects was calculated to assess memory performance.

### rsfMRI

rsfMRI was performed on 26 animals separated into four groups: wt, vehicle treated; wt, L41 treated; Dp1Yey, vehicle treated; Dp1Yey, L41 treated. rsfMRI was carried under medetomidine sedation during scanning [subcutaneous bolus injection, 0.3 mg/kg in 100 µl 0.9% NaCl solution right before the scan followed by continuous subcutaneous infusion of medetomidine (0.6 mg/kg, 200 µl/h)]. Physiological parameters were continuously monitored. rsfMRI data were collected using a 7 T small bore animal scanner and a mouse head adapted cryocoil (Biospec 70/20 and MRI CryoProbe, Bruker, Germany). The whole brain was examined [24 slices; 150×150×700 μm^3^ spatial resolution) using single shot gradient echo EPI (echo time/repetition time=10 ms/1700 ms)] and 200 volumes were recorded. The preprocessing included motion correction, data co-registration with Allen Mouse Brain Atlas (mouse.brain-map.org), detrending, band pass filtering (0.01-0.1 Hz) and regression of ventricular signal. For seed-based correlation analysis, the functional connectivity of several brain areas was mapped: RSP to map the default mode network, CA1, DG, PERI and anterior cingulate area (ACC). Correlation coefficients were then computed (two-tailed Student's *t*-test, *P*<0.001) between the seed region and the averaged BOLD signal time series of the remaining whole brain for each group and were converted and mapped to z-values using Fisher's r-to-z transformation.

### DYRK1A and GSK-3β protein levels

Brains were obtained from mice and snap-frozen until further use. Then tissues were weighed, homogenized and sonicated in 1 ml lysis buffer (60 mM β-glycerophosphate, 15 mM p-nitrophenylphosphate, 25 mM Mops pH 7.2, 15 mM EGTA, 15 mM MgCl_2_, 2 mM dithiothreitol, 1 mM sodium orthovanadate, 1 mM sodium fluoride, 1 mM phenylphosphate disodium and protease inhibitor cocktail) per g of material. Homogenates were centrifuged for 15 min at 17,000 ***g*** and 4°C. The supernatant was recovered and assayed for protein content (Bio-Rad, France). The proteins were separated by 10% NuPAGE pre-cast Bis-Tris polyacrylamide mini gel electrophoresis (Invitrogen, France) with MOPS-SDS running buffer. Proteins were transferred to 0.45-µm nitrocellulose filters (Schleicher and Schuell, Germany). They were blocked with 5% low-fat milk in Tris-buffered vehicle/Tween 20, and incubated overnight at 4°C with antibodies. Anti-DYRK1A (H00001859-M01; 1:1000) and anti-GSK-3α/β (KAM-ST002E; 1:1000) were obtained from Interchim (France) and Stressgen (France), respectively. Appropriate secondary antibodies conjugated to horseradish peroxidase (Bio-Rad) were added to visualize the proteins using the enhanced chemiluminescence reaction (ECL, Amersham, France).

### Protein kinase assays

Protein kinase assays to measure the catalytic activity of DYRK1A in the brains of the animals treated with or without drugs were performed as follows: frozen half brains were homogenized in lysis buffer (1.2 ml/half brain) using Precellys^®^ homogenizer tubes. After centrifugation at 2800 ***g*** for 2×15 s, 1 mg brain extract was incubated with 2 µg DYRK1A (H00001859 M01, Interchim) or GSK3-β (MBS8508391, Emelca Bioscience, France) antibodies at 4°C for 1 h under gentle rotation. Then, 20 µl protein G agarose beads (Thermo Fisher Scientific, France), previously washed three times with bead buffer (50 mM Tris pH 7.4, 5 mM NaF, 250 mM NaCl, 5 mM EDTA, 5 mM EGTA, 0.1% Nonidet P-40 and protease inhibitor cocktail from Roche, France), were added to the mix and gently rotated at 4°C for 30 min. After a 1 min spin at 10,000 ***g*** and removal of the supernatant, the pelleted immune complexes were washed three times with bead buffer, and a last time with Buffer C (60 mM β-glycerophosphate, 30 mM p-nitrophenolphosphate, 25 mM Mops pH 7.2, 5 mM EGTA, 15 mM MgCl_2_, 2 mM dithiothreitol, 0.1 mM sodium orthovanadate, 1 mM phenylphosphate, protease inhibitor cocktail). DYRK1A or GSK-3 immobilized on beads were assayed in buffer C as described in the Supplementary Materials and Methods with Woodtide (KKISGRLSPIMTEQ) (1.5 µg/assay) or GSK3-tide (YRRAAVPPSPSLSRHSSPHQpSED-EEE, where pS stands for phosphorylated serine) as substrates.

Protein kinase assays to evaluate SYN1 phosphorylation by DYRK1A were performed with 50 ng recombinant DYRK1A protein (PV3785, Thermo Fisher Scientific) and 0.98 mM Woodtide, and three peptides derived from the SYN1 putative DYRK1A phosphorylation site (Fig. 9C). Kinase activity was then measured as described in the Supplementary Materials and Methods.

The selectivity of the three leucettines used in this study was evaluated in a panel of 16 recombinant protein kinases assayed as described in the Supplementary Materials and Methods.

### Subcellular fractionation

Nuclear, cytosolic and synaptosomal subcellular fractionation of brain tissue was performed with the Syn-PER™ and ProteoExtract^®^ Tissue Dissociation Buffer Kit and Subcellular Proteome Extraction Kit following the instructions of the manufacturer. Fractions were analysed by SDS-PAGE and WB with specific antibodies.

### Phosphoproteomics results analysis

Gene ontology enrichment analyses of phosphoproteins that are modulated (up- or downregulated) in Tg(*Dyrk1a*) or Ts65Dn mice versus wt and also modulated in the opposite manner (down- or upregulated) by the L41 treatment, were conducted using ToppCluster (Bonferroni correction, *P*-value cutoff 0.05). Only biological processes common to the three brain regions and both models are presented (complete biological processes are listed in Tables S11-S13).

DYRK1A substrates and their respective phosphorylation sites were identified in the phosphoproteome based on the DYRK1A phosphorylation consensus sequence R-P-x(1,3)-S/T-P (Himpel et al., 2000). Protein-protein interactions of each substrate were generated with STRING web server application. Biological process enrichments of each cluster were assessed by using ToppCluster web server application. Phospho-network was mapped with the Cytoscape tool. See Supplementary Materials and Methods for details.

### Immunoprecipitation and immunoblotting

All immunoprecipitations were performed on fresh half brains of 3-month-old wt male mice. Brains were dissected and lysed in 1.2 ml RIPA lysis buffer (Santa-Cruz Biotechnology, France) using Precellys^®^ homogenizer tubes. After centrifugation at 2800 ***g*** for 2×15 s, 1 ml brain extract was incubated with 2 µg of antibody of interest at 4°C for 1 h under gentle rotation. An aliquot of the remaining supernatant was kept for further immunoblotting as homogenate control. Then, 20 µl protein G agarose beads, previously washed three times with bead buffer, were added to the mix and gently rotated at 4°C for 30 min. After a 1 min spin at 10,000 ***g*** and removal of the supernatant, the pelleted immune complexes were washed three times with bead buffer before WB analysis with appropriate antibodies directed against DYRK1A (H00001859 M01, Interchim; 1:1000), PSD95 (ab18258, Abcam, France; 1:1000), SYN1 (ab64581, Abcam; 1:1000), CAMK2A (PA5-14315, Thermo Fisher Scientific; 1:1000) and GAPDH (MA5-15738, Thermo Fisher Scientific; 1:3000). Immunoblots were revealed with Clarity Western ECL Substrate (Bio-Rad).

## Supplementary Material

Supplementary information
