# Supplementary material for: Correction of cognitive deficits in mouse models of Down syndrome by a pharmacological inhibitor of DYRK1A
Source: Dis Model Mech. 2018 Sep 27;11(9):dmm035634. doi: 10.1242/dmm.035634 (PMC6176987; doi:10.1242/dmm.035634)
Supplement: Supplementary information [file dmm-11-035634-s1.pdf]

SUPPLEMENTARY INFORMATION

Correction of cognitive deficits in mouse models of Down syndrome by pharmacological inhibitor of DYRK1A

Thu Lan **NGUYEN**, Arnaud **DUCHON**, Antigoni **MANOUSOPOULOU**, Nadège **LOAËC**, Benoît **VILLIERS**, Guillaume **PANI**, Meltem **KARATAS**, Anna E **MECHLING**, Laura-Adela **HARSAN**, Emmanuelle **LIMANTON**, Jean-Pierre **BAZUREAU**, François **CARREAUX**, Spiros D. **GARBIS**, Laurent **MEIJER** and Yann **HERAULT**

Material and Methods

Synthesis of Leucettines L41, L43 and L99.

Leucettine L41 was prepared as previously described (Debdab et al., 2011)

Leucettine L43 ((5*Z*)-5-(3,4-Dihydro-2*H*-1,5-benzodioxepin-7-ylmethylidene)-2-phenylamino-3,5-dihydro-4*H*-imidazol-4-one). Prepared from 4 equivalents of aniline using the Monowave® 300 Anton-Paar apparatus (reaction time = 30 min., temperature = 140°C, P = 200 Watt) in 52% yield as yellow powder. Mp > 260°C. ^{1}H NMR (300 MHz, DMSO-d_6): δ = 2.08-2.16 (m, 2H, CH_2), 4.17 (t, 4H, J = 5.4 Hz, OCH_2), 6.43 (s, 1H, =CH), 6.97 (d, 1H, J = 8.1 Hz, Ar), 7.07 (dd, 1H, J = 7.2, 7.2 Hz, Ar), 7.37 (dd, 2H, J = 8.4, 7.2 Hz, Ar), 7.61 (dd, 1H, J = 8.1, 0.9 Hz, Ar), 7.78 (d, 2H, J = 8.4 Hz, Ar), 7.85 (d, 1H, J = 0.9 Hz, Ar), 9.79 (br s, 1H, NH), 10.65 (br s, 1H, NH). ^{13}C NMR (75 MHz, DMSO-d_6): δ = 31.4, 70.4, 114.1, 114.2, 119.4, 121.0, 121.5, 122.9, 123.2, 125.9, 128.9, 130.9, 138.8, 150.7, 151.0, 154.8, 170.5. HRMS, *m/z* = 336.1347 found (calculated for $C_{19}H_{18}N_3O_3$, $[M+H]^+$ requires 336.1347). Anal. Calcd for $C_{19}H_{17}N_3O_3$: C, 68.05; H, 5.11; N, 12.53. Found: C, 67.88; H, 4.88; N, 12.51.

Leucettine L99 ((Z)-5-(4-hydroxy-3-methoxybenzylidene)-2-((4-(4-methylpiperazin-1-yl)phenyl)amino)-3,5-dihydro-4H-imidazol-4-one). Prepared from 1,1 equivalents of aniline using the Monowave® 300 Anton-Paar apparatus (reaction time = 30 min., temperature = 140°C, Pmax = 200 Watt) in 23% yield as green-yellow powder. Mp > 260°C. ^{1}H NMR (300 MHz, DMSO-d_6)
^{1}H NMR (300 MHz, DMSO-d_6) δ 2.22 (s, 3H, CH_3), 2.46 (t, J = 5.2 Hz, 4H), 3.09 (t, J = 4.9 Hz, 4H), 3.86 (s, 3H, CH_3), 6.39 (s, 1H), 6.78 (d, J = 8.1 Hz, 1H), 6.95 (m, 2H), 7.18 (dd, J = 8.6, 1.8 Hz, 1H), 7.68 (d, J = 8.6 Hz, 2H), 8.22 (d, J = 1.8 Hz, 1H), 9.47 (br s, 1H, NH), 10.51 (br s, 1H, NH). ^{13}C NMR (300 MHz, DMSO-d_6) δ 46.22, 49.05, 55.10, 55.63, 113.41, 115.33, 115.76, 116.16, 120.81, 125.04, 127.74, 131.35, 138.72, 147.42, 147.90, 154.26, 170.81. HRMS, *m/z* = 408.2037 found (calculated for $C_{22}H_{26}N_5O_3$, $[M+H]^+$ requires 408.20356). Anal. Calcd for $C_{22}H_{25}N_5O_3$: C, 64.85; H, 6.18; N, 17.19. Found: C, 64.03; H, 6.02; N, 17.01.

Detailed rsfMRI analysis

All animal experiments were performed in accordance with the guidelines and ethics on animal experimentation established by the German and French laws (ethical allowance 35_9185.81/G-13/15 from Regierungspräsidium Freiburg). Animal preparation, data acquisition and analysis were performed as described previously (Mechling et al., 2016). In brief, resting state magnetic resonance imaging (rsfMRI) was performed on 26 animals separated in 4 groups: wild type saline treated (Wild type – Saline); Wild Type – L41 treated mice; DP16 – Saline treated and DP16 – L41 treated. RsfMRI was carried our under medetomidine sedation (sc bolus injection, 0.3 mg per kg body weight in 100 µL 0.9% NaCl-solution right before the scan followed by continuous sc infusion of medetomidine (0.6 mg per kg body weight in 200 µL per hour) during scanning). The physiological parameters including body temperature, respiration rate, blood oxygen saturation and heart rate were continuously monitored to ensure stable and comparable imaging conditions for all animals. RsfMRI data was collected using single shot gradient echo EPI (TE/TR = 10 ms/1700 ms) at a 7 T small bore animal scanner (Biospec 70/20, Bruker, Germany) with a mouse head adapted cryocoil (MRI CryoProbe, Bruker, Germany). Following a shimming protocol, the whole mouse brain (excluding the cerebellum) was covered using

Disease Models & Mechanisms • Supplementary information

24 axial slices (0.7 mm slice thickness), with a field of view of 1.92 × 1.2 cm2 and an acquisition matrix of 128 × 80. This resulted in a planar spatial resolution of 150 × 150 µm2. 200 volumes were recorded with slice acquisition in interlaced fashion for each run. The preprocessing pipeline including coregistration with Allen mouse brain atlas (AMBA), creation of brain mask and smoothing with a Gaussian kernel of FWHM 0.4 x 0.4x 1 mm^3 was performed using MATLAB and SPM8 (http://www.fil.ion.ucl.ac.uk/spm/).

Seed-based correlation analysis: To evaluate the alterations of functional networks, several brain areas were selected as the regions of interest (ROIs). As all the data was normalized onto AMBA, each ROI was extracted using this atlas and used as seed region to perform FC analysis of the pre-processed rsfMRI data. Correlation coefficients were then computed (two-tailed t-test, $p < 0.001$) between the seed region and the averaged time series of the remaining whole brain for each group and were converted to z values using Fisher's r-to-z transformation. Statistical evaluation of the impact of L41 treatment on the FC was performed via group two-tailed t-test ($p < 0.01$), comparing wild type L41 treated animals versus saline treated wild type animals (WT L41 vs WT Sal) as well as DP16 - L41 treated animals versus Saline treated DP16 animals (DP16 L41 vs DP16 Sal). Included ROIs were: RSP: restrosplenial cortex to map the default mode network; CA1; Dentate gyrus; Perirhinal cortex and Anterior Cingulate area (ACC).

Assay of recombinant protein kinases

Kinase activities for each enzyme were assayed in Buffer A (10 mM $MgCl_2$, 1 mM EGTA, 1 mM dithiothreitol, 25 mM Tris-HCl pH 7.5, 50 µg heparin/mL) or Buffer B (50 mM $MgCl_2$, 90 mM NaCl, 30 mM Tris-HCl pH 7.4), with their corresponding substrates, in the presence of 15 µM ATP in a final volume of 30 µL. After 30 min incubation at 30°C, the reaction was stopped by harvesting, using a FilterMate harvester (Packard), onto P81 phosphocellulose papers (GE Healthcare) which were washed in 1% phosphoric acid. 20 µL of scintillation fluid were added and the incorporated radioactivity measured in a Packard counter. Blank values were subtracted and activities calculated as pmoles of phosphate incorporated during the 30 min incubation. Controls were performed with appropriate dilutions of dimethylsulfoxide (DMSO). Kinase activities were expressed in % of maximal activity, i.e. in the absence of inhibitors. IC_{50} values were obtained from the dose-response curves.

CDK1/cyclin B (M phase starfish oocytes, native, affinity purified), *CDK2/cyclin E* (human, recombinant, from A. Echalier), *CDK5/p25* (human, recombinant, expressed in *E. coli*) were assayed in Buffer A (supplemented extemporaneously with 0.15 mg BSA/mL, except for CDK2) with 25 µg of histone H1. *CDK9/cyclinT* (human, recombinant, expressed in insect cells) were assayed as described for CDK1/cyclin B, but using 8.07 µg of CDK7/9-Tide (YSPTSPSYSPTSPSYSPTSPSKKKK). *DYRK1A, 1B, 2, 3* (human, recombinant, expressed in *E. coli* as GST (glutathione S-transferase) fusion proteins), and *CLK1, 2, 3, 4* (mouse, recombinant, expressed in *E. coli* as GST fusion proteins) were assayed in Buffer A (supplemented extemporaneously with 0.15 mg bovine serum albumin (BSA)/mL) with 1 µg of RS peptide (GRSRSRSRSRSR) as a substrate. *GSK-3α/β* (glycogen synthase kinase -3, porcine brain, native, affinity purified on axin-sepharose beads), was assayed using a GSK-3 specific substrate (GS-1: YRRAAVPPSPSLSRHSSPHQpSEDEEE where pS stands for phosphorylated serine). *Pim1* (human, recombinant, expressed in *E. coli*) were assayed as described for DYRK1A with 25 µg of histone H1 as a substrate. *Casein kinase 2 (CK2)* (human, recombinant, obtained from C. Cochet) was assayed in Buffer B, with 7.79 µg of peptide substrate 29 (RRREDEESDDEE).

CNR1 receptor antagonism assay

The DiscoveRx PathHunter β-arrestin assay monitors the activation of a GPCR in a homogenous, non-imaging assay format using the Enzyme Fragment Complementation (EFC) technology with β-galactosidase (β-Gal) as the functional reporter. The enzyme is split into two inactive complementary portions (EA for Enzyme Acceptor and ED for Enzyme Donor) expressed as fusion proteins in the cell. EA is fused to β-Arrestin and ED is fused to the GPCR of interest, in this case the cannabinoid receptor CNR1. Dose-response curves were carried out on CNR1, in the presence of its agonist CP55940, for the three Leucettines L41, L43, L99 and IC_{50} values were determined. For antagonist assays, data was normalized to the maximal and minimal response observed in the presence of EC80 ligand and vehicle. An EC80 of 32 nM CP55940 was used. The effects of Leucettines are reported as % inhibition of fully activated CNR1 receptor by CP55940.

Disease Models & Mechanisms • Supplementary information

DYRK1A and GSK-3β mRNA levels

Total mRNAs were isolated from mice Tg(*Dyrk1a*) and Ts65Dn brain tissues using the RNeasy Plus kit (Qiagen, Hilden, Germany) according to the manufacturer's protocol**.** Total RNA (727ng) was reverse transcribed by the SuperScript III first strand synthesis kit (#18080-051 Invitrogen) in accordance with the manufacturer's instructions. mRNA expression profiles were analysed by real-time quantitative PCR using SsoFast EvaGreen Supermix (#172-5204 Bio-Rad) in a CFX96™ Touch real-time PCR detection system (Bio-Rad). Primers used for the detection of *Gsk-3β*, *Dyrk1a*, genes and housekeeping gene *Rplp0* are listed in Table (Eurofins, Ebersberg, Germany). The complete reactions were subjected to the following program of thermal cycling: 1 cycle of 30 seconds at 95°C; 50 cycles of 5 seconds at 95°C and 5 seconds at 59°C. A melting curve was run after the PCR cycles, followed by a cooling step. Each sample was run in simplicate in each experiment. Expression level of *Dyrk1a* is normalized to the expression level of *Gsk-3β* and *Tbp*.

Gene	Forward primer	Reverse primer
Gsk3β	5'-agtggtgagaagaaagatgaggtc-3'	5'-gagtgtctgcttggctcgac-3'
Dyrk1a	5'-tggggcagaggatataccagt-3'	5'-gtcgatagcaaggtcataaggca-3'
Rplp0	5'-cattgaaatcctgagtgatgtgcag-3'	5'-ggatgaccagcccaaaggag-3'

Phosphoproteomics, methods and analysis of results

In-solution Proteolysis and iTRAQ labeling. Specimens were vortexed in 0.5 M triethylammonium bicarbonate (TEAB), 0.05% sodium dodecyl sulfate (SDS) and pulsed probe sonicated (Misonix, Farmingdale, NY, USA). The resulting lysates were then centrifuged (16,000 g, 10 min, 4°C) and supernatants were measured for protein content using infrared spectrometry (Direct Detect, Millipore, Merck KGaA, Darmstadt, Germany). An equivalent of 100 µg total protein from each specimen was subjected to reduction, alkylation, trypsin proteolysis and isobaric tags for relative and absolute quantitation (iTRAQ) labeling per supplier's specifications (ABSciex, San Hose, CA, USA). The following iTRAQ specimen labeling assignment was used: 113 ≅ Ts65 T control; 114 ≅ Ts65 T 41; 115 ≅ Ts65Dn control; 116 ≅ Ts65Dn 41; 117 ≅ Tg(*Dyrk1a*) T control; 118 ≅ Tg(*Dyrk1a*) T 41; 119 ≅ Tg(*Dyrk1a*) control, and 121 ≅ Tg(*Dyrk1a*) 41. The eight iTRAQ labeled specimens were pooled and lyophilized to dryness.

Hydrophilic interaction chromatography (HILIC) Peptide Fractionation. The pooled specimen was reconstituted in 100 µL solution of 70% acetonitrile / 30% H_2O with 10 mM formic acid and subjected to high-performance HILIC separation (150-mm × 2.1 mm ID, 2.6 µm particle, Core-shell, Kinetex, Phenomenex, Torrance, CA, USA) at a flow rate of 200 µL/min and column temperature at 35°C. The multi-step gradient separation scheme was as follows : 10 min isocratic at 100% mobile phase A (70% acetonitrile / 30% H_2O with 10mM formic acid), linear gradient for 80 min to 40% mobile phase B (100% H_2O, 10 mM formic acid), second linear gradient for 30 min to 95% mobile phase B, isocratic for 10 min at 95% mobile phase B and linear gradient for 10 min gradient to 5% mobile phase B. Fractions were collected in a peak dependent manner as recorded at 218 nm signal response. A total of 50 fractions were collected, lyophilized to dryness, and stored at -20 °C until LC-MS analysis.

LC-MS analysis. The LC-MS experiments were performed on the Dionex Ultimate 3000 UHPLC system coupled with the high resolution nanospray LTQ-FT-Orbitrap Elite mass spectrometer (Thermo Scientific, Bremmen, Germany). Each peptide fraction was reconstituted in 30 µL loading solution (2% acetonitrile, 0.1% formic acid) and a 10 µL volume was loaded on the Acclaim PepMap 100, 100 µm ID × 2 cm C18, 5 µm particle, 100 Å pore) trapping column with the µ PickUp Injection mode. The loading pump flow rate was 5 µL/min and samples were loaded on the trapping column for 8 min. The analytical peptide separation was performed with low pH C_{18} Reverse Phase capillary ultra performance liquid chromatography (Acclaim PepMap, 75 µm ID × 50 cm, 2 µm particle, 100 Å pore, San Hose, CA, USA) at a flow rate of 300 n /min and column temperature at 50°C retrofitted. The multi-step gradient elution scheme was as follows : Isocratic at 100% mobile phase A (2% acetonitrile, 0.1% formic acid) ; linear gradient for 120 min to 85% mobile phase B, (100% acetonitrile, 0.1% formic acid), Isocratic at 85% mobile phase B for 5 min, and 100% mobile phase A, in 10 min. Nanospray ionization of eluted peptides was performed with metal coated silica

Disease Models & Mechanisms • Supplementary information

emitters (FS360-20-10-D-20-C7, New Objective, Woburn, MA, USA). Gaseous phase transition of the peptides was performed with positive ion electrospray ionization applying a voltage of 2.5 kV. Top 7 multiply charged precursors within 350 -1900 m/z and intensity threshold 500 counts were selected with FT mass resolution of 120,000 and isolated for both CID and HCD fragmentation within mass windows of 2 and 1.2 Da respectively. Normalized collision energy was set at 35 for the CID spectra and 40 for the HCD spectra that were acquired with FT resolution of 15,000 within a m/z range of 100-1900. Already targeted precursors were dynamically excluded for further isolation and activation for 30 sec with 10 ppm mass tolerance.

Protein Identification and Quantification. All tandem mass spectra were submitted to Sequest search engine implemented on the Proteome Discoverer software version 1.4 for peptide and protein identifications. All spectra were searched against an updated UniProt Fasta file containing 20,200 human reviewed entries. The Sequest node for the HCD spectra included the following parameters: Precursor Mass Tolerance 10 ppm, Fragment Mass Tolerance 20 mmu, Dynamic Modifications were Oxidation of M (+15.995 Da), Deamidation of N, Q (+0.984 Da), Phosphorylation of S (+79.966 Da) and Static Modifications were iTRAQ8plex at any N-Terminus, K, Y (+304.205 Da) and Methylthio at C (+45.988 Da). The Fragment Mass Tolerance for the CID spectra was 0.5 Da while search for phosphorylation was not included. The level of confidence for peptide identifications was estimated using the Percolator algorithm with decoy database searching. Strict FDR was set to 0.01, relaxed FDR was set to 0.05 and validation was based on q-Value. The Reporter Ion Quantifier node included a custom iTRAQ 8plex (Thermo Scientific Instruments) Quantification Method, integration window tolerance of 10 ppm with Most Confident Centroid. Protein ratios were normalized to protein median and phosphorylation localization probability was estimated with the phosphoRS node. Only uniquely occurring peptides were used for protein relative quantification and peptides with missing iTRAQ channels were excluded.

Phosphoproteomics analysis results. A total of about 70 tryptic peptide fractions were analyzed from each brain region. Only phosphoproteins identified at ≥99% confidence (peptide FDR ≤1%, q <0.01 based on decoy search) were accounted for. The cortex, cerebellum, and hippocampus regions reproducibly profiled a total of 1,199, 1,507, and 996 unique phosphoproteins, respectively. The mass spectrometry proteomics data have been deposited to the ProteomeXchange Consortium (http://proteomecentral.proteomexchange.org) via the PRIDE partner repository with the data set identifier PXD000XXX (Griss et al., 2013; Hermjakob & Apweiler, 2006; Csordas et al., 2013).

References

Csordas A, Wang R, Ríos D, Reisinger F, Foster JM, Slotta DJ, Vizcaíno JA, Hermjakob H, 2013. From Peptidome to PRIDE: public proteomics data migration at a large scale. *Proteomics* 13, 1692-1695.

Debdab, M., Carreaux, F., Renault, S., Soundararajan, M., Fedorov, O., Filippakopoulos, P., Lozach, O., Babault, L., Tahtouh, T., Baratte, B., Ogawa, Y., Hagiwara, M., Eisenreich, A., Rauch, U., Knapp, S., Meijer, L. and Bazureau, J.P., 2011. Leucettines, a class of potent inhibitors of cdc2-like kinases and dual specificity, tyrosine phosphorylation regulated kinases derived from the marine sponge leucettamine B. Modulation of alternative pre-RNA splicing. *J. Med. Chem.* 54, 4172-4186.

Griss J, Foster JM, Hermjakob H, Vizcaíno JA, 2013. PRIDE Cluster: building a consensus of proteomics data. *Nat Methods* 10, 95-96.

Hermjakob H, Apweiler R, 2006. The Proteomics Identifications Database (PRIDE) and the ProteomExchange Consortium: making proteomics data accessible. *Expert Rev Proteomics*. 3, 1-3.

Mechling AE, Arefin T, Lee HL, Bienert T, Reisert M, Ben Hamida S, Darcq E, Ehrlich A, Gaveriaux-Ruff C, Parent MJ, Rosa-Neto P, Hennig J, von Elverfeldt D, Kieffer BL, Harsan LA, 2016. Deletion of the mu opioid receptor gene in mice reshapes the reward-aversion connectome. *Proc Natl Acad Sci USA* 113, 11603-11608.

Supplementary Figures

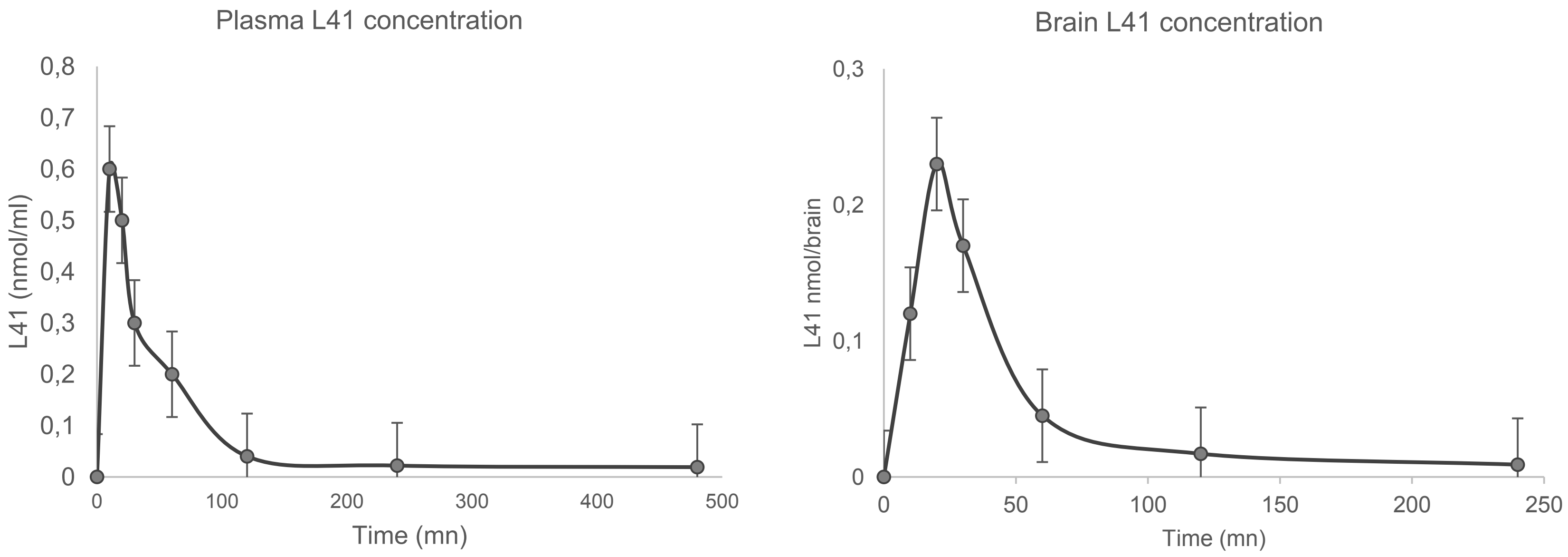

Suppl. Figure S1. Pharmacokinetics and brain biodistribution of L41 following acute intra-peritoneal injection. L41 concentration in the plasma and brain of Tg(*Dyrk1a*) and wt mice with a zoom on the first 8 hours.

Disease Models & Mechanisms • Supplementary information

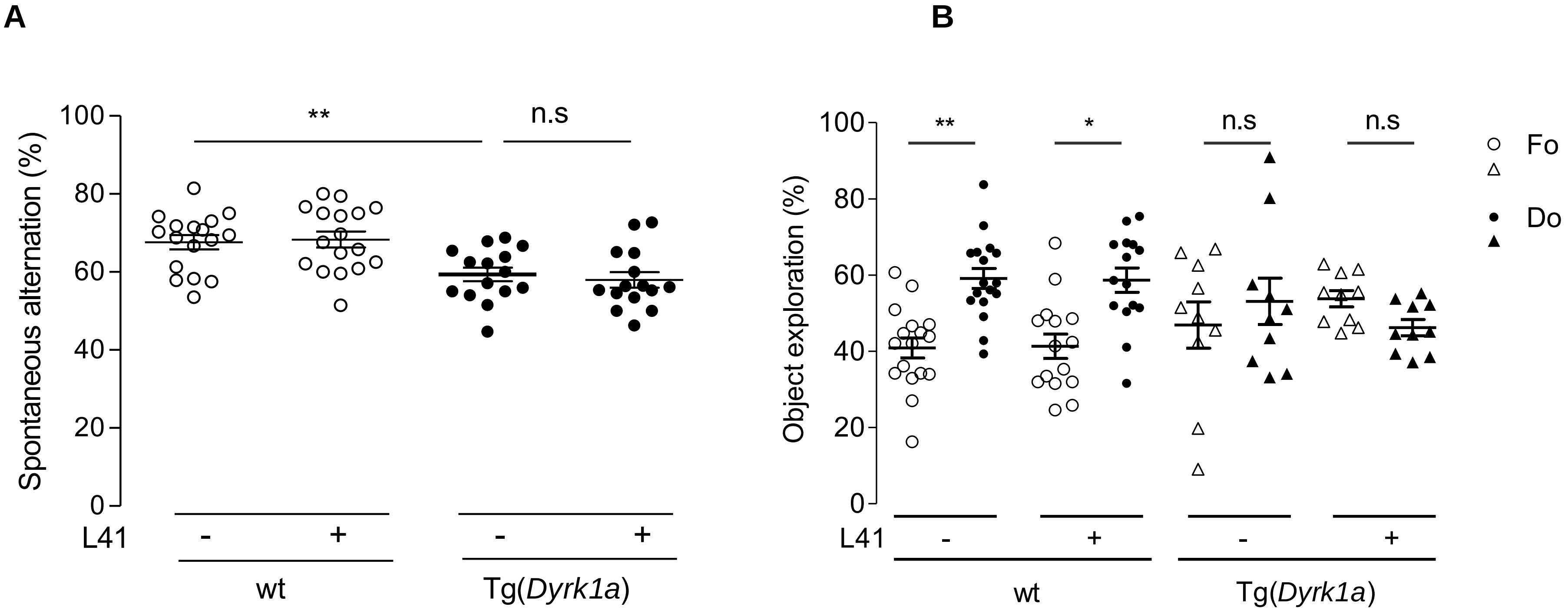

Suppl. Figure S2. L41 treatment has no effect on the working and spatial memory of Tg(*Dyrk1a*) mice. A. Y-maze results of a Tg(*Dyrk1a*) and control mice cohort treated or not with L41 (wt NT n=17; wt T n=17; Tg NT n=15; Tg T n=15). The percentage of spontaneous alternations is significantly decreased in the transgenic mice compared to controls treated or not with the L41 (p=0.003 and p=0.002 respectively). L41 treatment does not normalize the working memory deficit in Tg(*Dyrk1a*) mice (p=0.59). **B.** POR results of a cohort of Tg(*Dyrk1a*) and wt mice treated or not with L41. L41 treatment was unable to rescue the spatial memory impairment of the transgenic mice (wt NT n=17, 40% Fo and 60% Mo, p=0.003; wt T n=15, 41% Fo and 59% Mo, p=0.02; Tg NT n=10, 47% Fo and 53% Mo, p=0.62; Tg T n=10, 54% Fo and 46% Mo, p=0.11). Data are represented as mean ± s.e.m. with individual points per animal. Statistical analysis was performed with the student test. n.s., not significant; * p<0.05; ** p<0.01; *** p<0.001.

Disease Models & Mechanisms • Supplementary information

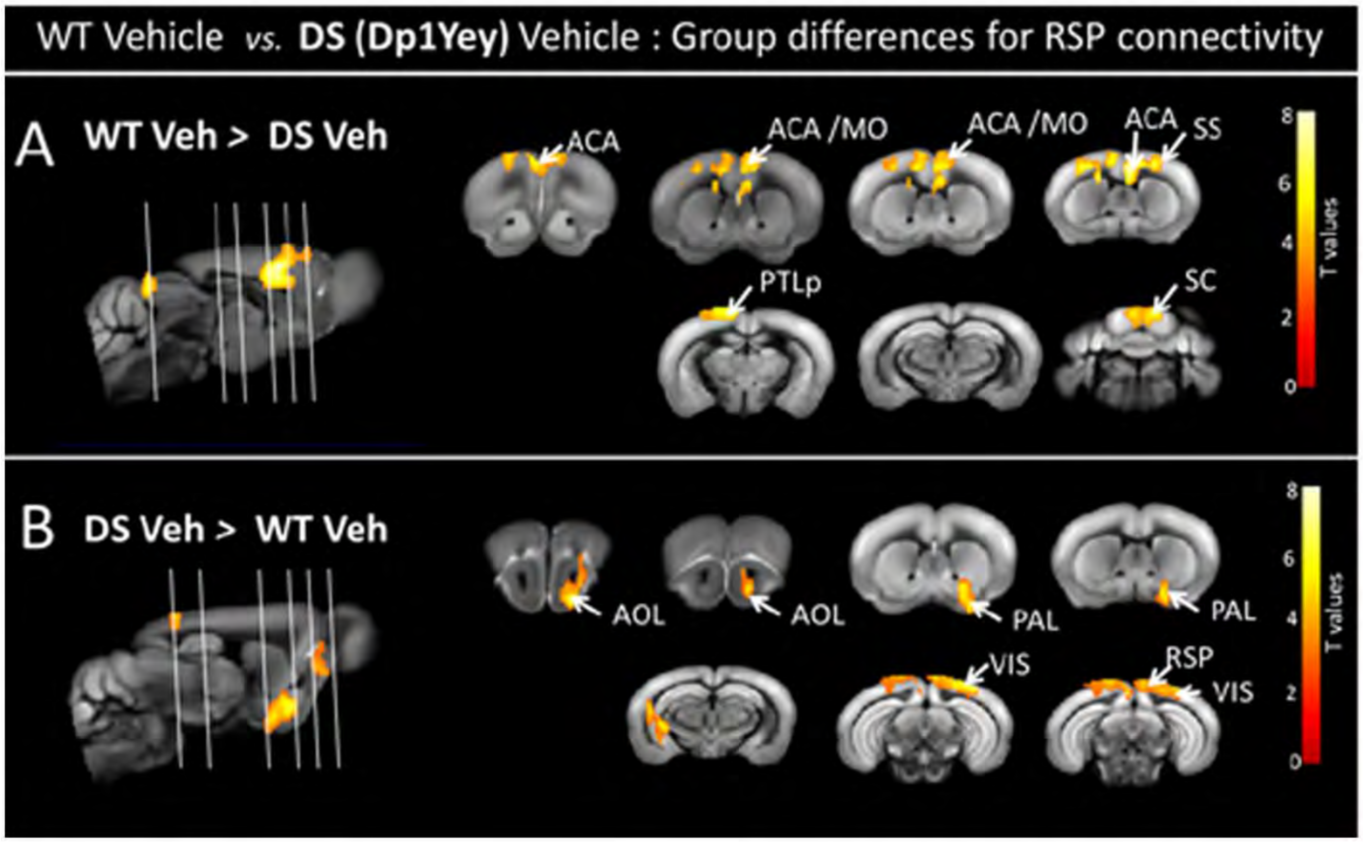

Suppl. Figure S3. Group statistical differences in RSP connectivity (default mode network) between wt vehicule treated mice and Dp1Yey, DS mouse model.

(A) Wt Vehicule animals display stronger long-range connectivity between RSP and fronto-cortical areas, including ACA, but also with parietal temporal areas and superior colliculus (WT Sal > DS Sal).
(B) Dp1Yey mice show increased short-range local connectivity around RSP seed areas, but also increased pattern of connectivity with ventral striatum (PAL), limbic area (DS Sal > WT Sal).

Disease Models & Mechanisms • Supplementary information

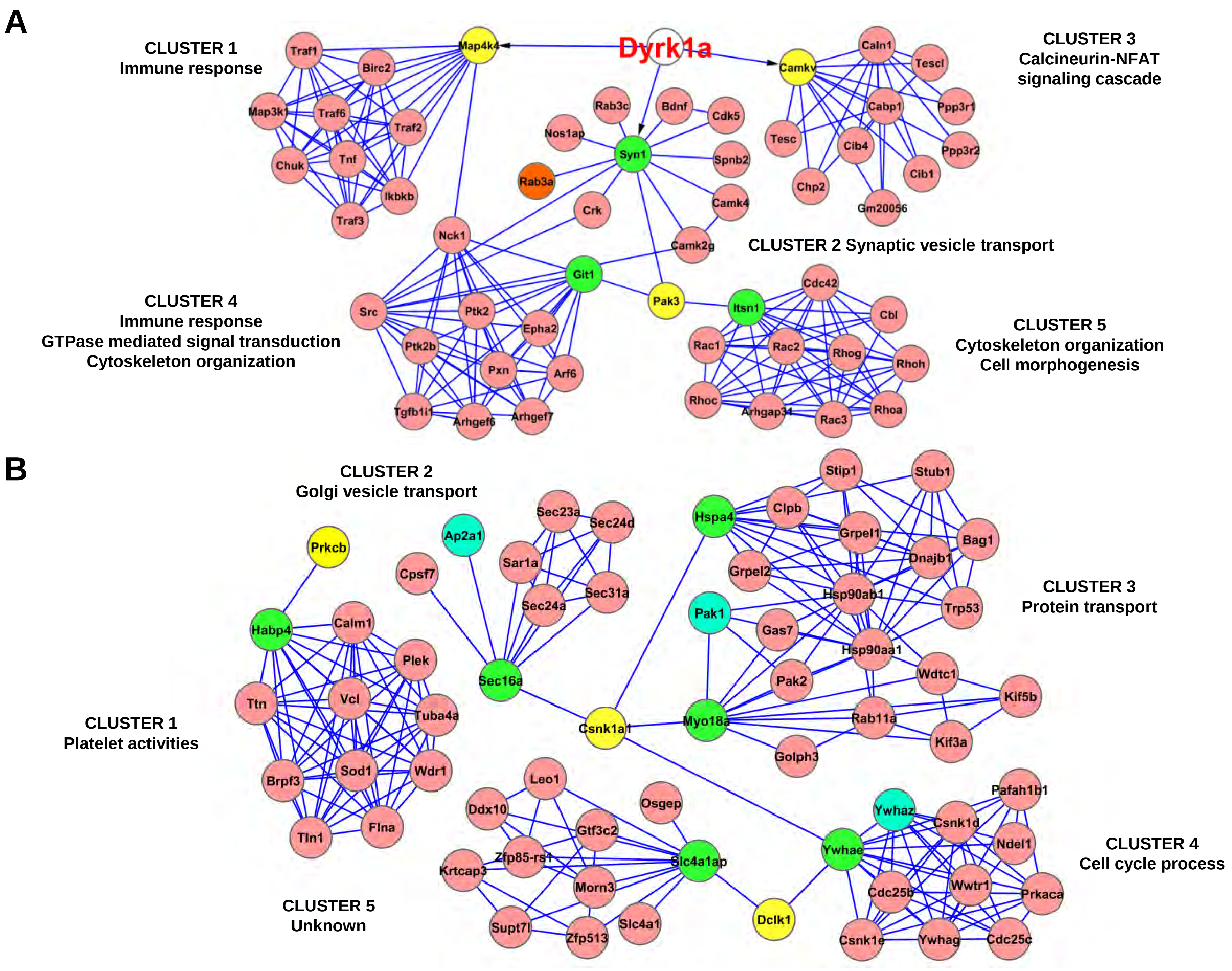

Suppl. Figure S4. Example of direct (A) and indirect (B) phosphorylation networks modulated in the transgenic line Tg(Dyrk1a) and sensitive to the L41 treatment.

Disease Models & Mechanisms • Supplementary information

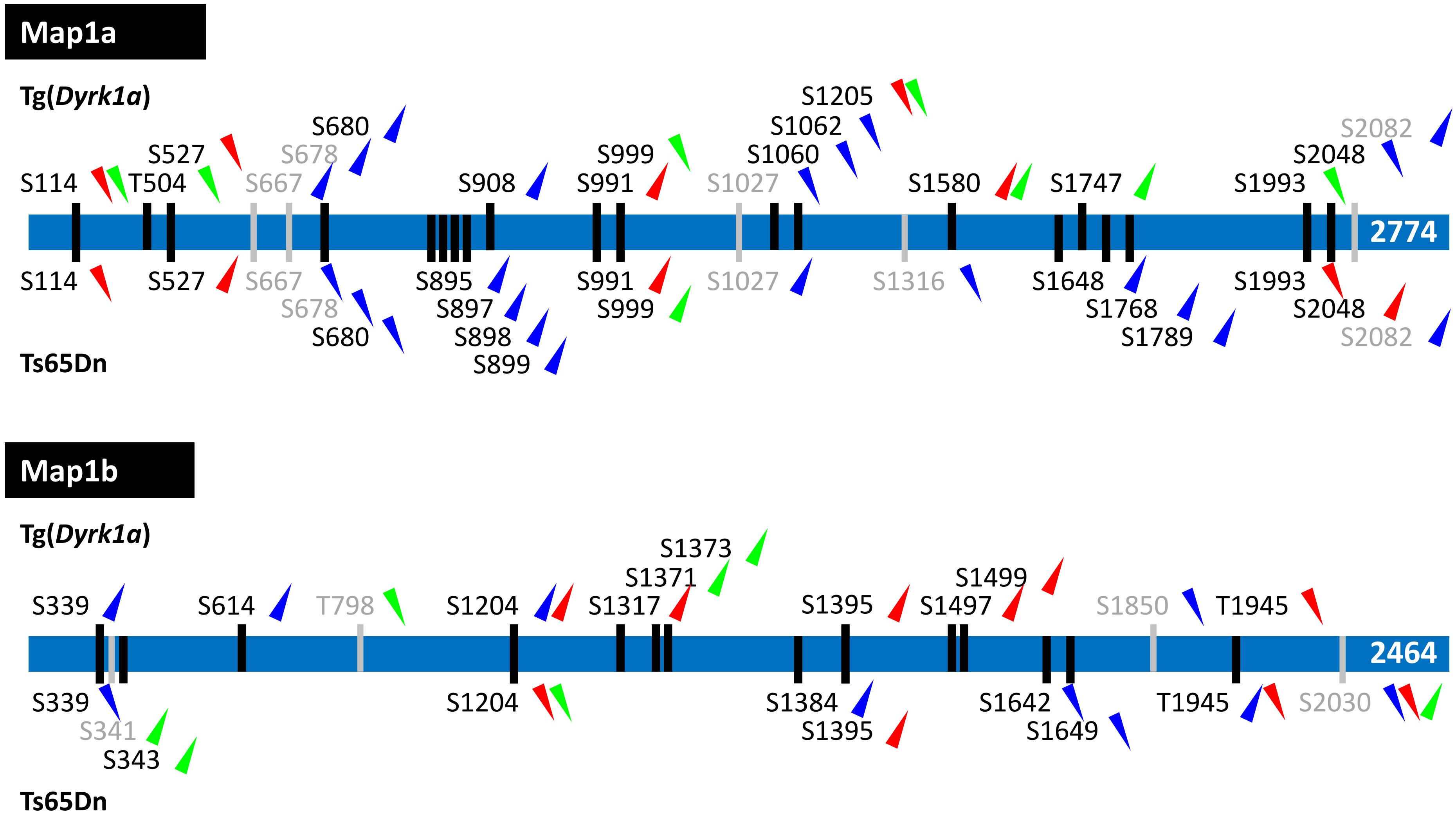

Disease Models & Mechanisms • Supplementary information

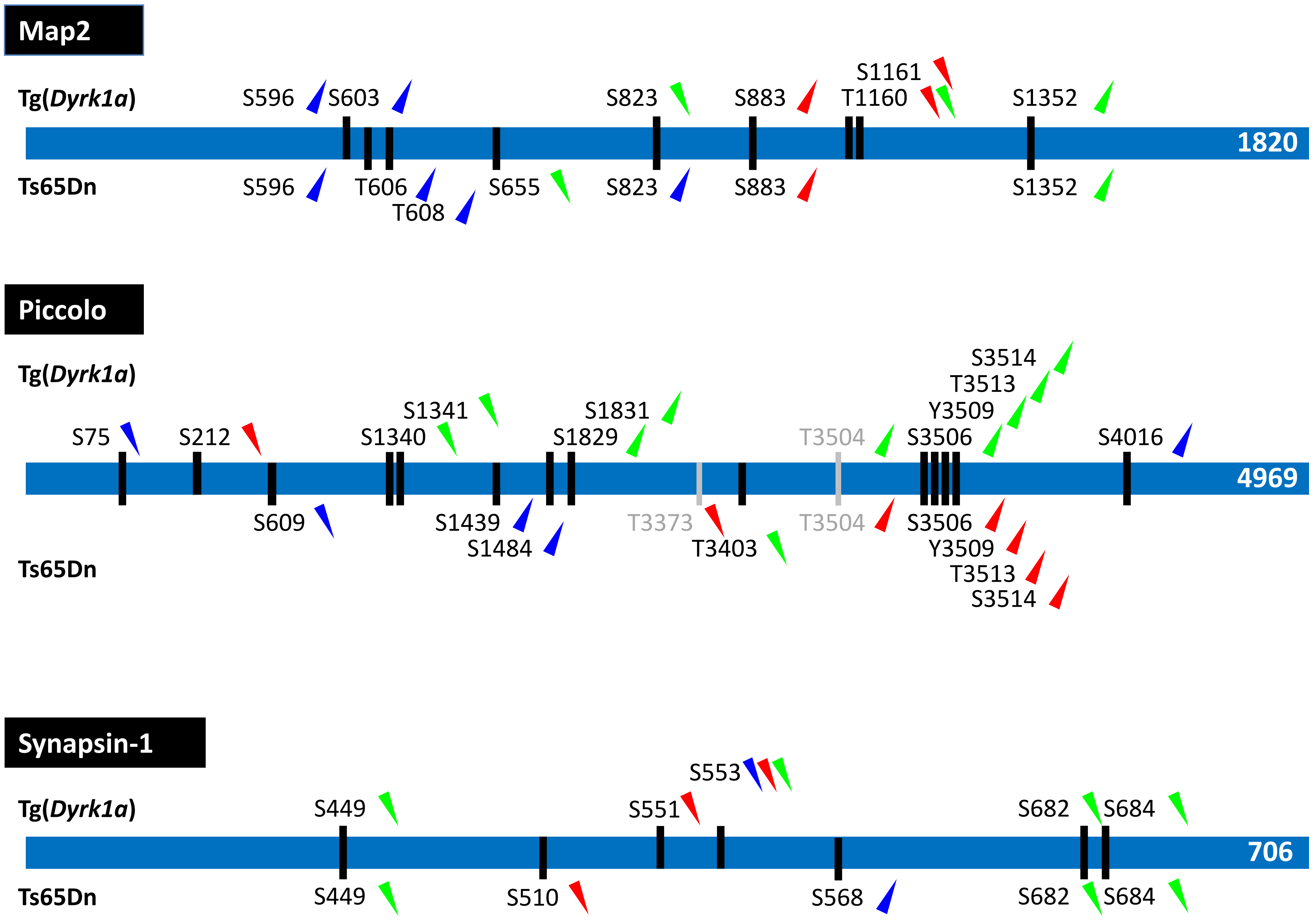

Suppl. Figure S5. Five phosphoproteins shared between Tg(*Dyrk1a*) and Ts65Dn mice common to the hippocampus, cortex and cerebellum. Schematic representation of each of the 5 common phosphoproteins displaying the identified phosphorylation sites in both *Tg(Dyrk1a)* (upper part) and Ts65Dn (lower part) models. Sites conserved in humans are indicated in black, while sites only seen in mice are in grey. Blue, red and green arrows show modulated phospho-sites in hippocampus, cortex and cerebellum, respectively. Downwards arrows indicate a site which is more phosphorylated in the transgenic vs. WT and less phosphorylated in L41 vs. vehicle treated mice. Upwards arrows indicate a site which is less phosphorylated in the transgenic vs. WT mice and more phosphorylated in L41 vs. vehicle treated mice.

Disease Models & Mechanisms • Supplementary information

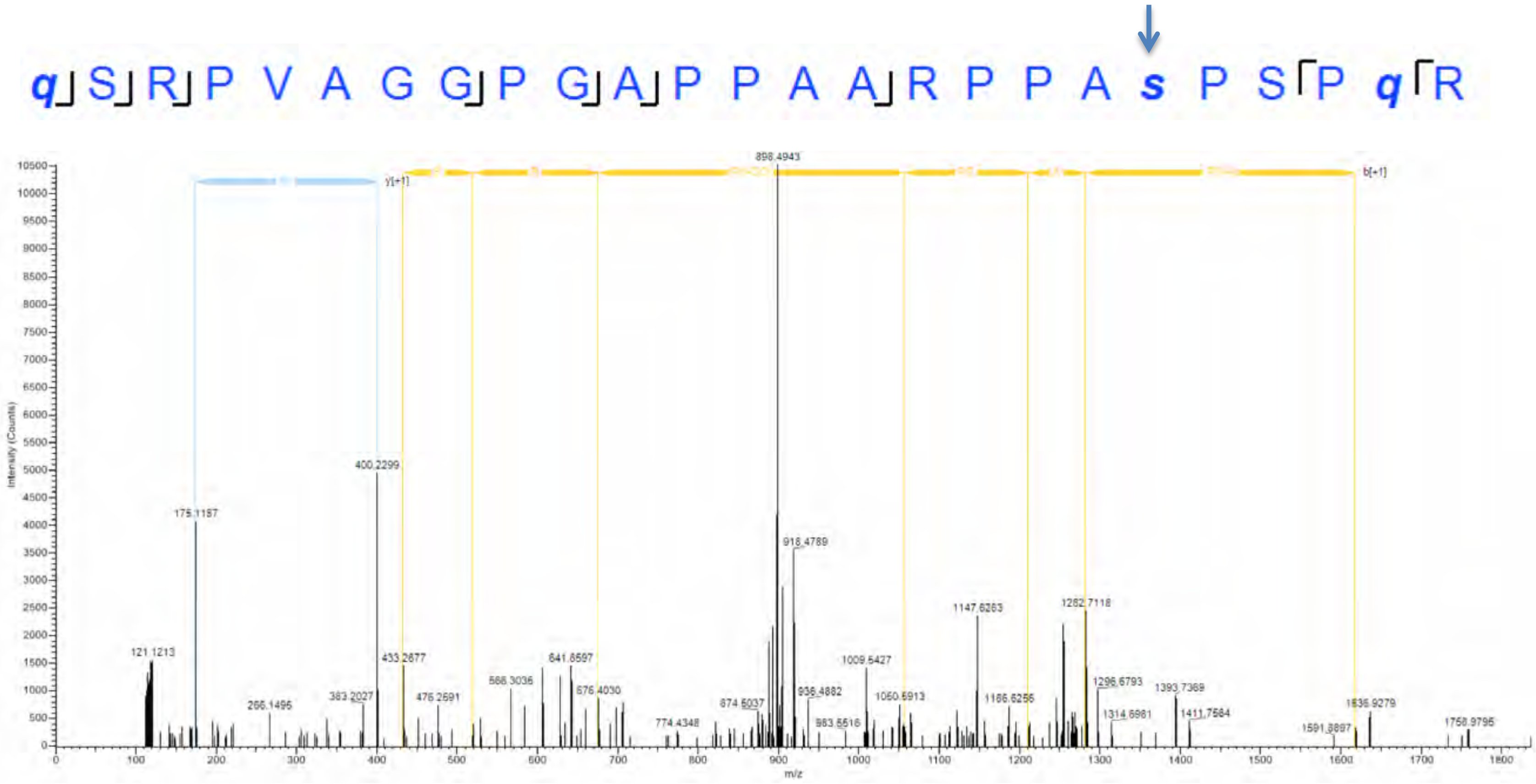

Suppl. Figure S6. Ultra-high resolution product ion spectrum of proteotypic peptide qSRPVAGGPGAPPAARPPAsPSPqR encoding for the phosphorylated residue Ser551 (denoted with lower case"s").

Disease Models & Mechanisms • Supplementary information

Supplementary Table S1. Proteins up and down phosphorylated in the hippocampus of Tg(*Dyrk1a*) mice and reverted by L41 treatment.

Gene name	Protein Group Accessions	Sequence	phosphoRS Site Probabilities	TgDyrk1a vs WT	TgDyrk1a +/-L41
Map1b	P14873	DLTTSSVEK	T(3): 0.0; T(4): 5.6; S(5): 88.8; S(6): 5.6	3,35	-2,96
Kat7	Q5SVQ0-4	SSGSETEQVVDFSDR	S(1): 44.4; S(2): 5.6; S(4): 44.4; T(6): 5.6; S(13): 0.0	2,53	-0,64
Sptbn1	Q62261	ESSPVPSPTLDR	S(2): 93.7; S(3): 6.3; S(7): 0.0; T(9): 0.0	2,11	-1,83
Mast2	B1AST8	NQSLGQSAPSLTAGLK	S(3): 0.0; S(7): 0.1; S(10): 0.1; T(12): 99.7	1,71	-1,36
Cep170b	Q80U49	HEDGTQSDSEDPLAK	T(5): 33.3; S(7): 33.3; S(9): 33.3	1,63	-0,59
Pp2d1	Q8BVT6	ECIISNLLR	S(5): 100.0	1,62	-0,97
4921517D22Rik	Q8CET0	TLNVSPLDSSETVSGNER	T(1): 0.0; S(5): 96.6; S(9): 0.4; S(10): 2.4; T(12): 0.4; S(14): 0.0	1,54	-1,34
Tsc22d2	E9Q7M2	TEDVSSEIFDVSR	T(1): 0.0; S(5): 50.0; S(6): 50.0; S(12): 0.0	1,42	0,06
Cttnbp2	B9EJA2-5	FQGNANDPDQNGNNTQSPPSR	T(15): 0.2; S(17): 88.2; S(20): 11.6	1,41	-0,20
Cep170b	Q80U49	HEDGTQSDSEDPLAK	T(5): 0.8; S(7): 98.5; S(9): 0.8	1,37	-0,75
Spink4	O35679	ELMVSAGSLVFPR	S(5): 87.6; S(8): 12.4	1,32	-0,39
H2-M10.4	Q85ZW8	TLIYMVTENNNK	T(1): 0.0; Y(4): 99.3; T(7): 0.7	1,31	0,32
Pak3	Q61036-2	IGQGASGTVYTALDIATGQEVAIK	S(6): 0.0; T(8): 0.0; Y(10): 0.0; T(11): 0.0; T(17): 100.0	1,31	-0,19
Pcdh11x	B1AZR7	GDSPIIEEHPL	S(3): 100.0	1,31	-0,93
Snap25	P60879	RADQLADESLESTR	S(9): 100.0; S(12): 0.0; T(13): 0.0	1,30	-0,33
Usp9x	Q4FE56	AQENYEGGEEVSPPQTK	Y(5): 0.0; S(12): 99.9; T(16): 0.1	1,28	-0,72
Prkcb	P68404-2	NIDQSEFEGFSFVNSEFLKPEVK	S(5): 0.5; S(11): 49.8; S(15): 49.8	1,24	-1,29
Mug2	P28666	FDLAFSSEVSGTLQK	S(6): 49.8; S(7): 49.8; S(10): 0.3; T(12): 0.0	1,23	-0,46
Cycs	P62897	TGQAAGFSYTDANK	T(1): 100.0; S(8): 0.0; Y(9): 0.0; T(10): 0.0	1,23	-2,83
Tln2	E9PUM4	FGLEGDEESTMLEESVSPK	S(9): 0.0; T(10): 0.0; S(15): 0.0; S(17): 100.0	1,22	-0,19
Gal	P47212	EAGALDSLPGIPLATSSEDLEK	S(7): 0.0; T(15): 7.5; S(16): 85.0; S(17): 7.5	1,12	-0,88
Scg2	Q03517	VPSPVSSEDDLQEEEQLEQAIK	S(3): 0.0; S(6): 50.0; S(7): 50.0	1,10	-0,48
Speg	E9QQ25	MPSIPEEPEHGDLER	S(3): 100.0	1,09	-0,41
Sept2	P42128	EGSPIPHDPDLGSK	S(3): 100.0; S(13): 0.0	1,09	-0,59
Myo18a	B2RRE2	EPADLDPEAASPAYSQAK	S(11): 100.0; Y(14): 0.0; S(15): 0.0	1,09	-0,22
Wfs1	Q3UN10	LNATASLEQDK	T(4): 6.4; S(6): 93.6	1,08	-0,38
Gbf1	Q6A099	ADAPDAGAQSDSELPSYHQNDVSLDR	S(10): 49.7; S(12): 49.7; S(16): 0.3; Y(17): 0.3; S(23): 0.0	1,08	-0,40
Slc1a3	P56564	DVEMGNSVIEENEMK	S(7): 100.0	1,07	-0,75
Sgk071	Q80YS9	SSSVALNMQRQK	S(1): 5.9; S(2): 47.0; S(3): 47.0	1,03	-0,53
Hspa4	Q3U2G2	MQVDQEEPHTEEQQQPQTPAENK	T(10): 0.0; T(19): 100.0	1,03	-0,60
Ppfia3	B8QI35	QLSIALPQEFAALTK	S(3): 100.0	1,01	-0,22
Ranbp2	Q9ERU9	NRPGYVSEEEEDDEDYEMAVK	Y(5): 0.6; S(7): 99.4; Y(16): 0.0	0,99	0,15
Plcl1	Q3USB7	KLPSESDLLEGEVTDEDEEAEMSR	S(4): 0.0; S(6): 0.0; T(14): 100.0; S(23): 0.0	0,99	-0,31
Slc4a1ap	E9Q585	MLGEDSDEEEEANTTEGK	S(6): 100.0; T(14): 0.0; T(15): 0.0	0,99	-0,74
Map2	P20357	LASVSADAEVAR	S(3): 100.0; S(5): 0.0	0,98	-0,50
Jup	Q02257	NEGTATYAAAVLFR	T(4): 0.1; T(6): 49.9; Y(7): 49.9	0,97	-0,67
Wdr59	Q8C0M0-2	ETEHISSPWGESSPEELR	T(2): 0.0; S(6): 0.0; S(7): 0.0; S(12): 50.0; S(13): 50.0	0,97	0,38
Bcl2a1d	O55179	LPQEQIALDVGAYK	Y(13): 100.0	0,94	-0,31
Eif2ak4	A2AUM0	EILDGLAYIHEK	Y(8): 100.0	0,93	0,13
Map1a	Q9QYR6	VPLSRSPQAQDTLGSLAGGQTGCTIQLLPEQDK	S(4): 47.2; S(6): 47.2; T(12): 2.3; S(15): 0.6; T(21): 2.3; T(24): 0.6	0,91	-0,56
Cx3cl1	O35188	SLGSEEINPVHTDNFQER	S(1): 0.0; S(4): 100.0; T(12): 0.0	0,91	-0,21
Huwe1	Q7TMY8	DLSMSEEDQMMR	S(3): 91.9; S(5): 8.1	0,89	-0,71
Duox2	A2AQ99	SFIEISNNCLSK	S(1): 0.0; S(6): 0.6; S(11): 99.4	0,86	-0,59
Dclk1	Q9JLM8	AQPAPPELNSESEDYSPSSSETVR	S(10): 0.0; S(12): 0.0; Y(15): 0.7; S(16): 98.6; S(18): 0.7; S(19): 0.0; S(20): 0.0; T(22): 0.0	0,86	-0,19
Camkv	Q3UHL1	SATPATDGRATPATEESTVPATQSSALPAAK	S(1): 2.4; T(3): 2.4; T(6): 2.4; T(11): 44.8; T(14): 44.8; S(17): 2.4; T(18): 0.6; T(22): 0.0; S(24): 0.0; S(25): 0.0	0,85	-0,26
Uhrf1bp1l	A2RSJ4	SPTHASPQHTQTEK	S(1): 0.0; T(3): 50.0; S(6): 50.0; T(10): 0.0; T(12): 0.0	0,84	0,00

Disease Models & Mechanisms • Supplementary information

Itsn1	E9Q0N0	SAFTPATATGSSPSPVLGQGEK	S(1): 0.0; T(4): 0.0; T(7): 0.0; T(9): 0.0; S(11): 0.0; S(12): 0.0; S(14): 100.0	0,83	-0,43
Pdap1	Q3UHX2	QYTSPEEIDAQLQAEK	Y(2): 5.0; T(3): 94.8; S(4): 0.3	0,83	-0,85
Map1b	P14873	ASLSPMDEPVPDSESPVEK	S(2): 0.0; S(4): 0.0; S(13): 0.0; S(15): 100.0	0,82	-0,09
Pclo	Q9QYX7	GSVPAAAAESPSMHR	S(2): 0.0; S(10): 94.2; S(12): 5.8	0,79	-0,74
Nrde2	Q80XC6	AYVQIQNK	Y(2): 100.0	0,78	-0,65
Txlna	Q6PAM1	EQGVESPGAQPASSPR	S(6): 0.0; S(13): 9.0; S(14): 91.0	0,78	-0,37
Ncl	P09405	NLSFNITEDELK	S(3): 100.0; T(7): 0.0	0,78	-0,52
Mtmr7	Q9Z2C9	SPSQGDEDSALILTQDNLK	S(1): 0.6; S(3): 92.2; S(9): 7.2; T(14): 0.0	0,77	-0,47
Marcks	P26645	KESGEGAEAEGATAEGAK	S(3): 100.0; T(13): 0.0	0,76	-0,53
Stab1	Q8R4Y4-2	TVGQILASTEVFTR	T(1): 0.0; S(8): 50.0; T(9): 50.0; T(13): 0.0	0,76	-0,48
Me1	P06801	LFYSVLMSDVEK	Y(3): 47.9; S(4): 47.9; S(8): 4.3	0,76	-0,07
Epb4.1l1	E9PV14	MIASPEGSETMR	S(4): 100.0; S(8): 0.0; T(10): 0.0	0,75	-0,35
Git1	Q68FF6	HGSGADSDYENTQSGDPLLGLEGK	S(3): 100.0; S(7): 0.0; Y(9): 0.0; T(12): 0.0; S(14): 0.0	0,74	-0,22
Nsun2	Q1HFZ0	EGVILTNENAASPEQPGDEDAK	T(6): 0.0; S(12): 100.0	0,74	-0,74
Syt7	E9PZA8	QQSLQQPLSQNQQGR	S(3): 99.7; S(9): 0.3	0,74	-0,20
Tom1l2	Q5SRX1	AAETVPDLPSPPTEAPAPASNTSTR	T(4): 0.0; S(10): 99.8; T(13): 0.2; S(20): 0.0; T(22): 0.0; S(23): 0.0; T(24): 0.0	0,73	0,29
Ssrp1	A2AW05	EGINPGYDDYADSDEDQHDAYLER	Y(7): 5.5; Y(10): 47.3; S(13): 47.3; Y(21): 0.0	0,73	-0,08
Ppp6r3	Q922D4-2	IQQFDDGGSDEEDIWEEK	S(9): 100.0	0,71	-0,13
Nefh	P19246	SPAEPKSPAEAK	S(1): 0.0; S(7): 100.0	0,71	-0,03
Map1a	Q9QYR6	SPPCEDFSVTGESEK	S(1): 100.0; S(8): 0.0; T(10): 0.0; S(13): 0.0	0,71	-0,21
Arfgap1	Q9EPJ9	NSNSDGWESWEGASGEGR	S(2): 50.0; S(4): 50.0; S(9): 0.0; S(14): 0.0	0,71	-0,28
Syn1	O88935	QSRPVAGGPGAPPAARPPASPSPQR	S(2): 0.0; S(20): 0.3; S(22): 99.7	0,70	0,30
Bai1	Q3UHD1	NENVATLSVSSLER	S(8): 0.0; S(10): 0.3; S(11): 99.7	0,69	-0,44
Srgap2	Q91Z67	QTPRSPDSTANVR	T(2): 0.0; S(5): 100.0; S(8): 0.0; T(9): 0.0	0,69	-0,17
Usp37	Q8C0R0-2	DYSSGVTNLDR	Y(2): 0.0; S(3): 50.0; S(4): 50.0; T(7): 0.0	0,68	-0,80
Nufip2	Q5F2E7	GADNDGSGSESGYTTPK	S(7): 0.1; S(9): 8.3; S(11): 91.5; Y(13): 0.1; T(14): 0.0; T(15): 0.0	0,68	-0,28
Hspa4l	P48722	SFDDPIVQTER	S(1): 100.0; T(9): 0.0	0,68	-0,24
Dnm2	Q3T9X3	RIEGSGDQVDTLELSGGAR	S(5): 0.0; T(11): 100.0; S(15): 0.0	0,68	-0,09
Farp1	F8VPU2	LGAPENSGISTLER	S(7): 0.7; S(10): 99.3	0,67	-0,35
Scrn1	Q9CZC8	AQSPCFGDDDPAK	S(3): 100.0	0,67	-0,35
Apba2	E9Q505	SASQDCIETTPGAQEGK	S(1): 50.0; S(3): 50.0; T(9): 0.0; T(10): 0.0	0,65	-0,06
Ranbp2	Q9ERU9	NRPGYVSEEEEDDEDYEMAVK	Y(5): 10.1; S(7): 89.9; Y(16): 0.0	0,65	-0,16
Hepacam	Q640R3	STTEPGPPGYSVSPPVPGRSPGLPIR	S(1): 0.0; S(11): 0.0; S(13): 0.0; S(20): 100.0	0,65	-0,36
Sdc4	O35988	VSMSSTAQGSNIFER	S(2): 0.7; S(4): 0.7; S(5): 5.4; T(6): 46.6; S(10): 46.6	0,64	-0,17
Pcdhga9	Q91XX9	AGQIRYSVPEETEK	Y(6): 0.0; S(7): 0.0; T(12): 100.0	0,64	-0,04
Ywhae	P62259	LGLALNFSVFYYEILNSPDR	S(8): 0.0; Y(11): 0.0; Y(12): 0.0; S(17): 100.0	0,64	-0,20
Sec16a	A2AIX1	APSLTSDSEGK	S(3): 90.3; T(5): 8.7; S(6): 1.0; S(8): 0.0	0,63	0,02
Csnk1a1	E9Q4G7	AAQQAASSSGQGQQAQTPTGK	S(7): 0.0; S(8): 0.0; S(9): 0.0; T(17): 99.6; T(19): 0.4	0,62	-1,05
Sort1	Q6PHU5	SGYHDDSDEDLLE	S(1): 0.0; Y(3): 0.0; S(7): 100.0	0,61	-0,46
D430041D05Rik	D3YZ21	GHSETSTLSSQPSIDEVR	S(3): 0.5; T(5): 97.7; S(6): 0.5; T(7): 0.5; S(9): 0.5; S(10): 0.5; S(13): 0.0	0,61	-0,26
Brap	Q99MP8-2	EQSESVNTAPESPSK	S(3): 0.0; S(5): 0.0; T(8): 0.1; S(12): 7.1; S(14): 92.8	0,60	-0,53
Slc6a11	P31650	AAEEARGSETLGGGGGGAAGTR	S(8): 100.0	0,60	-0,14
Lmtk3	Q5XJV6	VAENGLESPEKEER	S(8): 100.0	0,60	-0,19
Srrm2	Q8BTI8-3	ELSHSPPRENSFESSLEFK	S(3): 12.7; S(5): 86.9; S(11): 0.1; S(14): 0.1; S(15): 0.3	0,60	-0,35
Wdr20a	Q3UWE6	RNSTDSRPVSVTYR	S(3): 99.6; T(4): 0.4; S(6): 0.0; S(10): 0.0; T(12): 0.0; Y(13): 0.0	0,60	-0,51
Tnr	Q8BYI9	ITFTPSSGISSEVTVPR	S(6): 49.8; S(7): 49.8; S(10): 0.2; S(11): 0.2	0,59	-0,91
Aak1	Q3UHJ0-2	ILSDVTHSAVFGVPASK	S(3): 0.4; T(6): 93.9; S(8): 5.7; S(16): 0.0	0,59	-0,63
Dnajc19	D3Z5K6	EAALILGVSPTANK		0,59	-0,07

Disease Models & Mechanisms • Supplementary information

Ndrg3	Q9QYF9	SVTSNQSDGTQESCESPDVLDR	S(1): 0.0; T(3): 0.0; S(4): 0.0; S(7): 0.0; T(10): 0.0; S(13): 1.4; S(16): 98.6	0,59	-0,57
Unc13a	Q4KUS2	ESYSDSMHSYEEFSEPR	S(2): 0.0; Y(3): 0.0; S(4): 0.0; S(6): 0.0; S(9): 93.7; Y(10): 6.3; S(14): 0.0	0,59	-0,33
Eif4g1	Q6NZJ6	EATLPPVSPPK	T(3): 0.0; S(8): 100.0	0,58	-0,16
Zswim8	Q3UHH1-5	RTFANGSADEFQR	T(2): 91.1; S(7): 8.9	0,58	-0,08
Lmtk3	Q5XJV6	VAENGLESPEKEER	S(8): 100.0	0,58	-0,33
Col4a1	P02463	GFPGPPGPDGLPGSMGPPGTPSVDHGFLVTR	S(14): 98.9; T(20): 1.1; S(22): 0.0; T(30): 0.0	0,58	-0,29
6430548M08Rik	Q3TA40	RSSSNESFSSNQSADSAPDEETLALR	S(2): 33.3; S(3): 33.3; S(4): 33.3; S(7): 0.1; S(9): 0.0; S(10): 0.0; S(13): 0.0; S(16): 0.0	0,58	0,19
Fxyd7	P59648	SELPSSAPGGGGV	S(1): 0.8; S(5): 91.2; S(6): 8.0	0,57	0,01
Pacsin1	Q61644	GRLDSGQLGLYPANYVEAI	S(5): 100.0; Y(11): 0.0; Y(15): 0.0	0,57	-0,05
Tcp11l1	Q8BTG3	QEAENGALDISK	S(11): 100.0	0,57	-0,16
Hspa4	Q3U2G2	STNEAMEWMNSK		0,57	-0,21
Plcl2	Q8K394	LSSNCSGVEGDVTDEDEGAEMSQR	S(2): 0.0; S(3): 0.0; S(6): 0.0; T(13): 100.0; S(22): 0.0	0,56	0,03
Clns1a	Q923F1	LGEESKEPLSDEDEEDNDDVEPISEFR	S(5): 50.0; S(10): 50.0; S(24): 0.0	0,56	-0,28
Rnf20	Q5DTM8	ALVVPEPEPDSDSNQER	S(11): 6.2; S(13): 93.8	0,56	-0,52
Amph	Q7TQF7	TPSPPEEPSPLPSPTASPNHTLAPASPAPVRPR	T(1): 4.5; S(3): 92.6; S(9): 0.3; S(13): 0.3; T(15): 1.1; S(17): 0.1; T(21): 1.1; S(26): 0.0	0,56	-0,56
Bcmo1	E9Q321	YLQSDTYIANIEANR	Y(1): 0.0; S(4): 0.1; T(6): 7.2; Y(7): 92.8	0,56	-0,28
Gas2l1	Q8JZP9	RYSGDSDSSASSAQSGPMGAR	Y(2): 8.0; S(3): 8.0; S(6): 82.4; S(8): 0.8; S(9): 0.8; S(11): 0.0; S(12): 0.0; S(15): 0.0	0,55	-0,73
Gvin1	L7N451	NSQEILDNQK	S(2): 100.0	0,55	0,04
Ggt7	Q99JP7	LPSSSSEMGSQDGSPLR	S(3): 0.0; S(4): 0.0; S(5): 0.0; S(6): 0.0; S(10): 0.0; S(14): 100.0	0,55	0,18
Pkd1l2	E9Q4D4	SFSQGFSPVLGFR	S(1): 50.0; S(3): 50.0; S(7): 0.0	0,55	-0,50
Sparcl1	P70663	AEKPSALNSEEETHEQSTEQDK	S(5): 33.1; S(9): 33.1; T(13): 33.1; S(17): 0.3; T(18): 0.3	0,54	0,10
Chd3	F7C528	ELQGDGPPSSPTNDPTVK	S(9): 33.3; S(10): 33.3; T(12): 33.3; T(16): 0.0	0,54	-0,60
Tmem161a	M0QWV0	YIHPSEEELR	Y(1): 50.0; S(5): 50.0	0,54	-0,83
Gfpt1	P47856-2	VDSTTCLFPVEEK	S(3): 3.7; T(4): 3.7; T(5): 92.7	0,53	-0,10
Stk39	Q9Z1W9	TEDGDWEWSDDEMDEK	T(1): 0.0; S(9): 100.0	0,53	-0,65
Jhdm1d	Q3UWM4	EDGFQPQSYLVQGVK	S(8): 92.9; Y(9): 7.1	0,53	-0,22
Tom1	O88746	GLEFPMTDLDMLSPIHTPQR	T(7): 0.0; S(13): 100.0; T(17): 0.0	0,53	-1,27
Ubap2	A2AMY5	IAYQSSASPPDSAPGSVANGHGGGR	Y(3): 1.3; S(5): 9.9; S(6): 9.9; S(8): 78.9; S(12): 0.0; S(16): 0.0	0,53	-0,27
Stim1	P70302	LIEGVHPGSLVEK		0,53	-0,21
Caskin1	Q6P9K8-2	AAASVVSGPPVASDCASPGDSAR	S(4): 0.0; S(7): 0.0; S(13): 2.5; S(17): 97.0; S(21): 0.4	0,53	-0,01
Alad	P10518	DAAQSSPAFGDR	S(5): 50.0; S(6): 50.0	0,52	-0,07
Slc12a5	Q91V14	ETDPEVHLTWTK	T(2): 0.0; T(9): 99.6; T(11): 0.4	0,52	-0,59
Nefm	P08553	QASHAQLGDAYDQEIR	S(3): 100.0; Y(11): 0.0	0,51	0,03
Ube2w	D6RCG4	ELLALQNDPPPGMTLNEK	T(14): 100.0	0,51	-0,60
Mtdh	Q80WJ7	SETNWESPK	S(1): 0.0; S(7): 100.0	0,50	-0,39
Tbc1d5	Q80XQ2	EFTGSPPPSATK	T(3): 0.0; S(5): 100.0; S(9): 0.0; T(11): 0.0	0,50	-0,59
Habp4	Q9JKS5	SLAASGAQQPDSPGGPQPPGQK	S(1): 0.0; S(5): 0.0; S(12): 100.0	0,50	-0,63
Nvl	Q9DBY8	FEDVGGNDATLK	T(10): 100.0	0,50	0,22
Wwp1	Q8BZZ3	SSSPPIEIQQNGDALHENGDPATR	S(1): 0.4; S(2): 49.8; S(3): 49.8; T(23): 0.0	0,49	0,17
Ephb2	A3KG01	ELSEYNATAIK	S(3): 10.0; Y(5): 79.9; T(8): 10.0	0,49	-0,45
Shank1	D3YZU5	EQDVVPFQESPK	S(10): 100.0	0,49	0,01
Slc7a14	Q8BXR1	ETCSPVSEGEEFSSPATNTCGAK	T(2): 33.3; S(4): 33.3; S(7): 33.3; S(13): 0.0; S(14): 0.0; T(17): 0.0; T(19): 0.0	0,48	-0,88
Map4k4	F8VPL5	RDSPLQGGGQQNSQAGQR	S(3): 100.0; S(13): 0.0	0,48	-0,20
Csrp1	P97315	GFGFGQGAGALVHSE	S(14): 100.0	0,48	-0,17
Map1a	Q9QYR6	SHWDDGTNDSDLEK	S(1): 0.0; T(7): 0.0; S(10): 100.0	0,48	-0,31

Disease Models & Mechanisms • Supplementary information

Xirp2	Q4U4S6-2	VDASHQIEVEGVTR		0,48	-0,09
Epb4.1l1	A2AUK5	SDSETEGLVFAR	S(1): 0.0; S(3): 100.0; T(5): 0.0	0,48	-0,29
Slc1a4	O35874	SEEETSPLVTHQNPAGPVAIAPELESK	S(1): 0.0; T(5): 98.9; S(6): 1.1; T(10): 0.0; S(26): 0.0	0,47	-0,81
Misp	Q9D279	EMWYAGINPSDSVNSEVLGATRVK	Y(4): 0.1; S(10): 0.7; S(12): 0.7; S(15): 2.0; T(21): 96.5	0,47	-0,92
Grin2b	G3X9V4	QHSYDTFVDLQK	S(3): 2.9; Y(4): 97.1; T(6): 0.0	0,47	-0,09
Fubp1	Q3TUE1	QQAAYYAQTSPQGMPQHPPAPQGQ	Y(5): 0.0; Y(6): 0.0; T(9): 1.6; S(10): 98.3	0,47	-0,23
Map1a	Q9QYR6	SEPQDFQEDSWGDTK	S(1): 0.0; S(10): 5.4; T(14): 94.6	0,47	-0,05
Rbbp8	Q80YR6	MDVTVIDTK	T(4): 100.0; T(8): 0.0	0,46	-0,72
Arhgef2	H3BJU7	LSPPHSPR	S(2): 100.0; S(6): 0.0	0,46	-0,59
Hn1	P97825	SNSSEASSGDFLDLK	S(1): 0.0; S(3): 50.0; S(4): 50.0; S(7): 0.0; S(8): 0.0	0,46	-0,03
Uncharacterized protein C17orf59 homolog	Q9D6W8	TLSGEEEAESVGVSSR	T(1): 0.0; S(3): 100.0; S(10): 0.0; S(14): 0.0; S(15): 0.0	0,46	-0,35
Epb4.1l1	A2AUK5	DKSDSETEGLVFAR	S(3): 100.0; S(5): 0.0; T(7): 0.0	0,46	-0,17
Nefh	P19246	SPAAVKSPAEAK	S(1): 0.0; S(7): 100.0	0,46	-0,04
Gm4763	Q8CFJ5	SQTSIGIFSVVEVSNPGSSK	S(1): 1.6; T(3): 48.3; S(4): 48.3; S(9): 1.6; S(14): 0.0; S(18): 0.0; S(19): 0.0	0,46	-0,29
Stmn1	P54227	SKESVPDFPLSPPK	S(1): 0.0; S(4): 0.0; S(11): 100.0	-0,43	1,15
Trp53i11	A2AGS6	KHSQTDLVSR	S(3): 100.0; T(5): 0.0; S(9): 0.0	-0,43	0,11
Npnt	D3YTX1	VTGLHSGTLQVFVRK	T(2): 50.0; S(6): 50.0; T(8): 0.1	-0,43	0,41
Uncharacterized protein C4orf21 homolog	Q0VGT4	SSSELSEVINNMSLLK	S(1): 0.5; S(2): 2.3; S(3): 12.9; S(6): 0.5; S(13): 83.9	-0,43	0,53
Fxr1	Q61584-4	VPGVTAIELDEDTGTFR	T(5): 0.0; T(13): 0.3; T(15): 99.7	-0,43	0,53
Apc	E9QLQ9	SPSEGPGATTSPR	S(1): 0.0; S(3): 0.0; T(9): 1.2; T(10): 9.9; S(11): 88.9	-0,43	0,47
Nktr	P30415	SRASSSSSHHSSK	S(1): 0.0; S(4): 0.0; S(5): 0.0; S(6): 0.0; S(7): 1.2; S(8): 1.2; S(11): 87.9; S(12): 9.7	-0,43	1,35
Camsap2	Q8C1B1	DYTVSLDSDMDDASK	Y(2): 0.2; T(3): 4.5; S(5): 90.7; S(8): 4.5; S(14): 0.0	-0,44	-0,14
Map2	P20357	VGSLDNAHHVPGGGNVK	S(3): 100.0	-0,44	0,08
Prrt2	E9PUL5	QENGAVVPLQAGDGEEGPAPQPHSPPSTK	S(24): 100.0; S(27): 0.0; T(28): 0.0	-0,44	0,20
Fam169a	Q5XG69	SQSEEQSEASSEHLEQFTQSAEK	S(1): 0.0; S(3): 0.0; S(7): 0.0; S(10): 33.2; S(11): 33.2; T(18): 33.2; S(20): 0.3	-0,44	0,00
Nyap2	Q8BM65-2	TPTSPLEELTSLFTSGR	T(1): 0.4; T(3): 0.0; S(4): 99.6; T(10): 0.0; S(11): 0.0; T(14): 0.0; S(15): 0.0	-0,45	0,35
Map7d1	A8Y5P4	RSSQPSPTTVPASDSPPAK	S(2): 6.6; S(3): 93.4; S(6): 0.0; T(8): 0.0; T(9): 0.0; S(13): 0.0; S(15): 0.0	-0,45	-0,19
Myo5a	D3YZ62	TSSIADEGTYTLDSILR	T(1): 0.0; S(2): 0.5; S(3): 99.5; T(9): 0.0; Y(10): 0.0; T(11): 0.0; S(14): 0.0	-0,45	0,07
Trappc8	E9PWG2	EFHMVQVSSSSK		-0,45	0,31
Specc1	Q5SXY1	GSPTGSSPNNASELSLASLTEK	S(2): 31.6; T(4): 31.6; S(6): 31.6; S(7): 5.0; S(12): 0.2; S(15): 0.0; S(18): 0.0; T(20): 0.0	-0,46	-0,05
Kctd12	Q6WVG3	SPSGGAAGPLLTPSQSLDGSR	S(1): 99.5; S(3): 0.5; T(12): 0.0; S(14): 0.0; S(16): 0.0; S(20): 0.0	-0,46	0,61
Cep170b	Q80U49	ESPLSPPTVPDPGGATPGSAR	S(2): 0.6; S(5): 99.4; T(8): 0.0; T(16): 0.0; S(19): 0.0	-0,46	0,36
Gas7	B1ATI9	STGDSQNLGSSSPGR	S(1): 0.0; T(2): 0.0; S(5): 0.0; S(10): 5.4; S(11): 5.4; S(12): 89.2	-0,46	0,18
C2cd2l	Q80X80	SLSPAATVTAELHYEQGSPR	S(1): 0.0; S(3): 0.0; T(7): 0.0; T(9): 0.0; Y(14): 0.0; S(18): 100.0	-0,46	0,59
Prrc2b	F8WHT3	LKFSDDEDEEDVVK	S(4): 100.0	-0,47	0,48
Rgs14	P97492	SLGSGESESESRPGK		-0,47	0,33
Map6	Q7TSJ2	GQSPTAPGPTK	S(3): 100.0; T(5): 0.0; T(10): 0.0	-0,47	0,34
Sept_4	P28661-2	VARPQILEPRPQSPDLCDDDVEFR	S(13): 100.0	-0,47	0,17
Map2	P20357	STELGSDYYELSDSR	S(1): 0.0; T(2): 0.0; S(6): 0.0; Y(8): 0.0; Y(9): 0.0; S(12): 0.0; S(14): 100.0	-0,47	0,25
Slc4a10	Q5DTL9-2	DSGLEDGRESPSFDTPSQR	S(2): 0.0; S(10): 99.5; S(12): 0.5; T(15): 0.0; S(17): 0.0	-0,47	-0,39

Disease Models & Mechanisms • Supplementary information

Utp20	Q5XG71	EVMEANLPSILLK	S(9): 100.0	-0,47	0,09
Basp1	Q91XV3	SDAAPAASDSKPSSAEPAPSSK	S(1): 0.0; S(8): 98.8; S(10): 0.6; S(13): 0.6; S(14): 0.0; S(20): 0.0; S(21): 0.0	-0,48	0,54
Srrm2	Q8BTI8-3	SLLPNSSQDELMEVEK	S(1): 0.2; S(6): 49.9; S(7): 49.9	-0,48	0,30
Stmn1	P54227	ESVPDFPLSPPKK	S(2): 0.0; S(9): 100.0	-0,48	0,42
Zfp26	P10076	AFSTSSGLVEHIR		-0,48	-0,35
Trhde	Q8K093	ILITQQHFIYDIGAK	T(4): 50.0; Y(10): 50.0	-0,48	0,42
Myh9	Q8VDD5	DLQGRDEQSEEK	S(9): 100.0	-0,48	0,53
Cacna1a	P97445	EMGTDGYSDSEHYLPMEGQTR	T(4): 0.0; Y(7): 49.9; S(8): 49.9; S(10): 0.3; Y(13): 0.0; T(20): 0.0	-0,49	0,21
Rab3a	P63011	MSESLDTADPAVTGAK	S(2): 100.0; S(4): 0.0; T(7): 0.0; T(13): 0.0	-0,49	-0,26
Palm2	Q8BR92	AELVLIDEDDEKSLR	S(13): 100.0	-0,50	0,25
Ctnnb1	Q02248	RTSMGGTQQQFVEGVR	T(2): 50.0; S(3): 50.0; T(7): 0.0	-0,50	-0,04
Prrc2c	Q3TLH4-5	AFGSGIDIKPGTPPIGGR		-0,50	0,49
Mtpap	Q9D0D3	EFFEYFGNFAFNK	Y(5): 100.0	-0,51	0,20
Rbm25	B2RY56	LGASNSPGQPNSVK	S(4): 0.0; S(6): 100.0; S(12): 0.0	-0,51	-0,15
Arhgef28	G5E8P2	LSLPMAAGHGGDAGDGAEENILYL	S(2): 96.9; Y(23): 3.1	-0,51	0,37
Lrrc7	Q80TE7	LETTPTTSPLPER	T(3): 2.1; T(4): 32.6; T(6): 32.6; T(7): 32.6; S(8): 0.0	-0,52	0,45
Vps26b	Q8C0E2	SMSHQAAIASQR	S(1): 0.9; S(3): 99.1; S(10): 0.0	-0,52	-0,19
Nap1l4	Q78ZA7	EFITGDVEPTDAESAWHSENEEEDK	T(4): 0.0; T(10): 0.2; S(14): 1.3; S(18): 98.6	-0,52	0,39
C2cd2l	Q80X80	EAGLSQSHDDLSNTTATPSVR		-0,53	0,29
Pacsin1	Q61644	KAEGATLSNATGAVESTSQAGDRGSVSSYDR	T(6): 0.0; S(8): 0.0; T(11): 0.0; S(16): 2.9; T(17): 2.9; S(18): 2.9; S(25): 87.7; S(27): 2.9; S(28): 0.6; Y(29): 0.1	-0,53	0,28
Basp1	Q91XV3	KAEGAGTEEEGTPK	T(7): 0.0; T(12): 100.0	-0,53	0,20
Atp5j	P97450	QASGGPVDIGPEYQQDLDR	S(3): 100.0; Y(13): 0.0	-0,54	-0,41
Specc1	Q5SXY1	ASSEDTLNKPGSASSGVAR	S(2): 50.0; S(3): 50.0; T(6): 0.0; S(12): 0.0; S(14): 0.0; S(15): 0.0	-0,54	0,38
Gm14597	B1AXE0	VLAFVEEDPLSHELK		-0,54	-0,19
Palmd	Q9JHU2	RSNGPTHTSPTRPTPQPR	S(2): 0.0; T(6): 0.0; T(8): 50.0; S(9): 50.0; T(11): 0.0; T(14): 0.0	-0,55	0,70
Dhx9	E9QNN1	AEAENNSGVESSGYGSPGPTWDR	S(7): 0.0; S(11): 0.0; S(12): 0.0; Y(14): 0.0; S(16): 99.1; T(20): 0.8	-0,55	-0,10
Ppp1r9a	H3BL28	DLTGGDLTSPDASASSCGR	T(3): 0.0; T(8): 50.0; S(9): 50.0; S(13): 0.0; S(15): 0.0; S(16): 0.0	-0,55	0,45
Slc9a6	A1L3P4	LVLPMDDSEPALNSLDDTRHSPA	S(8): 0.0; S(14): 0.0; T(18): 6.0; S(21): 94.0	-0,55	-0,02
Bsn	O88737	TLPSPPPEEAHLPLAGQVPSQLYAASLLQR	T(1): 0.0; S(4): 13.3; S(20): 86.4; Y(23): 0.4; S(26): 0.0	-0,55	-0,27
Col3a1	P08121	GPAGPPGPPGASGSPGLQGMPGER	S(12): 50.0; S(14): 50.0	-0,55	-0,15
Rbm5	Q91YE7-2	GLVAAYSGDSDNEEELVER	Y(6): 0.0; S(7): 0.0; S(10): 100.0	-0,56	0,52
Tstd1	E9PY03	GLQATQLAQGLGYTGAR	T(5): 99.8; Y(13): 0.1; T(14): 0.1	-0,56	0,22
Marcksl1	P28667	AAATPESQEPQAK	T(4): 50.0; S(7): 50.0	-0,56	0,32
Zranb2	D3Z4U0	YNLDASEEEDSNK	Y(1): 0.0; S(6): 100.0; S(11): 0.0	-0,56	0,50
Atrx	Q61687	YVESDDEKPTDENVNEK	Y(1): 11.2; S(4): 88.6; T(10): 0.2	-0,57	-0,18
Dmtn	Q9WV69	ESVGGSPQSK		-0,58	0,41
Tsg101	Q61187	QILNLYAEENAIEDTIFYLGEALR	Y(6): 50.0; T(15): 50.0; Y(18): 0.0	-0,58	0,48
Slc9a6	A1L3P4	LVLPMDDSEPALNSLDDTRHSPA	S(8): 0.0; S(14): 0.0; T(18): 0.7; S(21): 99.3	-0,59	0,25
Dclk1	Q9JLM8	VCSSMDENDGPGEGDELGR	S(3): 6.9; S(4): 93.1	-0,59	0,16
Rtn3	Q9ES97-2	ETTGGAPTMSPDLEQEQLTIR	T(2): 0.0; T(3): 0.0; T(8): 0.0; S(10): 100.0; T(19): 0.0	-0,59	0,32
Dbn1	Q9QXS6	SPSDSSTASTPIAEQIER	S(1): 0.0; S(3): 99.9; S(5): 0.1; S(6): 0.0; T(7): 0.0; S(9): 0.0; T(10): 0.0	-0,59	0,46
Map1a	Q9QYR6	GFKSPPCEDFSVTGESEK	S(4): 100.0; S(11): 0.0; S(16): 0.0	-0,59	0,18
Ctnna2	Q61301-2	QVQEAIAGISSAAQATSPTDEAK	S(10): 0.0; S(11): 0.0; T(16): 0.4; S(17): 5.8; T(19): 93.9	-0,60	0,88
Shisa7	F6T9T6	SSSLTPGLGGPDSMAPR	S(1): 4.7; S(2): 47.6; S(3): 47.6; T(5): 0.1; S(13): 0.0	-0,60	0,16

Disease Models & Mechanisms • Supplementary information

Map1b	P14873	DYNASASTISPPSSMEEDK	Y(2): 0.0; S(5): 0.0; S(7): 0.0; T(8): 0.0; S(10): 99.6; S(13): 0.2; S(14): 0.2	-0,61	-0,25
Pacsin1	Q61644	STSGPGMPMNWPQFEEWNPDLPHTTAK	S(1): 0.0; T(2): 0.0; S(3): 1.1; T(24): 97.9; T(25): 1.1	-0,61	0,41
8030462N17Rik	Q0VAW6	SRSESETSTMAAK	S(1): 0.2; S(3): 99.7; S(5): 0.0; T(7): 0.0; S(8): 0.0; T(9): 0.0	-0,61	0,22
Tmem163	Q8C996	ISESGQFSDGLEDR	S(2): 0.0; S(4): 100.0; S(8): 0.0	-0,61	0,55
Slc7a8	Q9QXW9	NHPGSDTSPEAEASSGGGGVALK	S(5): 0.2; T(7): 49.9; S(8): 49.9; S(14): 0.0; S(15): 0.0	-0,62	0,07
Sirt2	Q8VDQ8	REHANIDAQSGSQAPNPSTTISPGKSPPPAK	S(10): 0.0; S(12): 0.0; S(18): 0.7; T(19): 0.7; T(20): 32.9; S(22): 32.9; S(26): 32.9	-0,62	0,21
Lman1l	Q8VCD3-2	QSQILQALQALSR	S(2): 100.0; S(12): 0.0	-0,62	0,53
Cep170b	Q80U49	GSLDWPEEER	S(2): 100.0	-0,63	0,29
Add1	Q9QYC0	SPPDQSAVPNTPPSTPVKLEEDLPQEPTSR	S(1): 0.0; S(6): 0.3; T(11): 77.5; S(14): 11.1; T(15): 11.1; T(28): 0.0; S(29): 0.0	-0,63	0,34
Gap43	P06837	ATTDNSPSSK	S(6): 94.8; S(8): 5.2; S(9): 0.0	-0,63	0,70
ptplad1	Q8K2C9	WLDESDAEMELR	S(5): 100.0	-0,63	0,21
Add2	Q9QYB8	TESVTSGPLSPEGSPSKSPSK	T(1): 0.0; S(3): 0.0; T(5): 0.0; S(6): 0.0; S(10): 0.0; S(14): 0.0; S(16): 0.0; S(18): 93.7; S(20): 6.3	-0,63	0,50
Basp1	Q91XV3	AEGAGTEEEGTPKESEPQAAADATEVK	T(6): 1.4; T(11): 97.2; S(15): 1.4; T(24): 0.0	-0,63	0,57
Prkar1a	Q9DBC7	TDSREDEISPPPPNPVVK	T(1): 0.0; S(3): 0.0; S(9): 100.0	-0,63	0,67
Tppp	Q7TQD2	AANKTPPKSPGDPAR	T(5): 100.0; S(9): 0.0	-0,63	0,67
Dmtn	Q9WV69	KGAEEEEEEEDDDSEEEIK	S(14): 100.0	-0,64	0,90
Palmd	Q9JHU2	SGPQCSSPTCQEETEDVR	S(1): 0.0; S(6): 50.0; S(7): 50.0; T(9): 0.0; T(14): 0.0	-0,64	0,37
Rem2	E9Q4D5	QASPLGTPTPEADTTLLK	S(3): 100.0; T(7): 0.0; T(9): 0.0; T(14): 0.0; T(15): 0.0	-0,64	0,29
Syk	E9PWE9	ELNGTYAISGGR	T(5): 50.0; Y(6): 50.0; S(9): 0.0	-0,64	0,17
Hnrnpll	Q921F4	LKTEEGEIVYSAEESENR	T(3): 0.0; Y(10): 0.0; S(11): 100.0; S(15): 0.0	-0,64	0,56
Akap5	D3YVF0	GPVAASPQK	S(6): 100.0	-0,65	0,18
Map1b	P14873	EEQSPVKAEVAEK	S(4): 100.0	-0,65	-0,10
Gap43	P06837	KEGDGSATTDAAPATSPKAEEPSK	S(6): 0.0; T(8): 0.0; T(9): 0.0; T(15): 7.2; S(16): 92.8; S(23): 0.0	-0,65	0,72
Caskin1	Q6P9K8-2	AQPGSPQALGGPHGPATAK	S(5): 100.0; T(17): 0.0	-0,65	0,00
Ace	P09470	GPQFGSEVELR	S(6): 100.0	-0,67	0,17
Strip2	E9QN41	QDSLDIYNER	S(3): 100.0; Y(7): 0.0	-0,67	0,98
Marcksl1	P28667	AAATPESQEPQAK	T(4): 99.5; S(7): 0.5	-0,67	0,41
Dnah7b	L7N1Y0	SSDEVVNEVAGDILSK	S(1): 7.5; S(2): 92.5; S(15): 0.0	-0,67	-0,02
Thrap3	Q569Z6	ASVSDLSPR	S(2): 0.0; S(4): 0.0; S(7): 100.0	-0,68	0,25
Agap2	Q3UHD9	TDSQSEAVAIQAIR	T(1): 0.0; S(3): 99.8; S(5): 0.2	-0,68	0,34
Clip2	Q9Z0H8	TGNESGSNLSDSGSVK	S(5): 0.0; S(7): 91.1; S(10): 8.9; S(12): 0.0; S(14): 0.0	-0,69	-0,06
Hnrnpa0	Q9CX86	LFIGGLNVQTSESGLR		-0,69	0,20
Kif2a	P28740-1	ARPSQLPEQSSSAQQNGSVSDISPVQAAK	S(4): 0.0; S(10): 0.0; S(11): 0.0; S(12): 0.0; S(18): 0.0; S(20): 0.0; S(23): 100.0	-0,70	0,50
Krt26	Q3TRJ4	EALSEELTYLQK	S(4): 0.0; T(8): 50.0; Y(9): 50.0	-0,70	0,72
Nudt3	Q9JI46	SESEEEVLLVSSSR	S(1): 0.0; S(3): 100.0; S(11): 0.0; S(12): 0.0; S(13): 0.0	-0,70	0,61
Cend1	Q9JKC6	SASSPKPDTK	S(1): 0.0; S(3): 4.9; S(4): 95.1; T(9): 0.0	-0,71	0,51
Prrt2	E9PUL5	ASPSPSPSEAR	S(2): 99.3; S(4): 0.1; S(6): 0.7; S(8): 0.0	-0,71	0,37
Uncharacterized protein C17orf59 homolog	Q9D6W8	RGSPGGVEMNVELPQQEGDDDDDEDEEAAAGR	S(3): 100.0	-0,72	0,74
Map1a	Q9QYR6	AELEEMEEVHPSDEEEEETKAESFYQK	S(12): 33.3; T(19): 33.3; S(23): 33.3; Y(25): 0.2	-0,72	0,55
Nsmf	A2AJ96	MYSVDGVSDDVPIR	Y(2): 0.0; S(3): 100.0; S(8): 0.0	-0,72	0,29
Nucks1	Q80XU3	ATVTPSPVK	T(2): 0.0; T(4): 0.0; S(6): 100.0	-0,73	0,30
Aldoa	P05064	GILAADESTGSIAK	S(8): 0.3; T(9): 5.5; S(11): 94.2	-0,73	0,34
Pde4d	B1PSD9	EWYQSTIPQSPSPAPDDQEEGR	S(5): 0.0; S(10): 99.8; S(12): 0.2	-0,73	0,51
Cep170b	Q80U49	QESFTKEPTSGPPAPGK	S(3): 93.6; T(5): 6.4; T(9): 0.0; S(10): 0.0	-0,75	0,63
Fam171a2	A2A699	SVEGPGGLEPSLDEYR	S(1): 100.0; S(11): 0.0; Y(15): 0.0	-0,75	0,71
Shank1	D3YZU5	GSSTEDGPGPVPPPSPR	S(2): 13.5; S(3): 86.5; S(14): 0.0	-0,76	0,42

Disease Models & Mechanisms • Supplementary information

Zc2hc1a	Q8BJH1	ASSVNSPLGNKPQTLSPSHR	S(2): 0.0; S(3): 0.0; S(6): 0.0; T(14): 0.0; S(16): 99.5; S(18): 0.5	-0,76	0,63
Hcn2	O88703	RAPPGPLPPAASPGPPAASPPAAPSSPR	S(12): 0.0; S(19): 100.0; S(25): 0.0; S(26): 0.0	-0,77	0,46
Marcksl1	P28667	GDVTAEEAAGASPAK	T(4): 0.0; S(12): 100.0	-0,77	0,41
Pcm1	Q9R0L6	VTNAISPESSPGVGR	T(2): 0.0; S(6): 100.0; S(9): 0.0; S(10): 0.0	-0,78	0,15
Dzip1l	Q499E4-2	GSGGLASSGTLVQSIVK	S(2): 0.0; S(7): 91.1; S(8): 8.1; T(10): 0.8; S(14): 0.0	-0,79	1,35
Sirt2	Q8VDQ8	EHANIDAQSGSQAPNPSTTISPGKSPPPAK	S(9): 0.0; S(11): 0.0; S(17): 42.8; T(18): 42.8; T(19): 6.6; S(21): 1.1; S(25): 6.6	-0,81	0,24
Nap1l4	Q78ZA7	ASGDGESLDEDSEFTLASDFEIGHFFR	S(2): 0.0; S(7): 100.0; S(12): 0.0; T(15): 0.0; S(18): 0.0	-0,81	0,49
Chgb	P16014	EGVDDQESLRPSNQQASK		-0,82	-0,01
Slc7a14	Q8BXR1	HKQNSEALIANDELDCSPE	S(5): 0.0; S(17): 100.0	-0,83	0,24
Rimbp2	Q80U40-2	DHLSPDFYDESETDPGAEELPAR	S(4): 100.0; Y(8): 0.0; S(11): 0.0; T(13): 0.0	-0,84	0,16
Mtfr1l	Q9CWE0	ASSFADMMGILK	S(2): 50.0; S(3): 50.0	-0,84	1,02
Mccc2	Q3ULD5	AYHGDSVARLGTQPDSASSTYQENYEQMK	S(6): 0.0; S(16): 9.7; S(18): 45.1; S(19): 45.1	-0,85	0,47
Ankrd34a	B2RW11	APSLPAPPPSGAPGSPR	S(3): 0.0; S(10): 0.0; S(15): 100.0	-0,85	0,39
Basp1	Q91XV3	ETPAASEAPSSAAK	T(2): 0.0; S(6): 0.0; S(10): 5.8; S(11): 94.2	-0,86	0,60
Luzp1	Q8R4U7	EKPDSDDDLDIESFVTAK	S(5): 100.0; S(13): 0.0; T(16): 0.0	-0,88	0,85
Ogfr	Q99PG2	IALNLEGCALSPTSQEPR	S(11): 90.4; T(13): 8.7; S(14): 0.9	-0,89	-0,38
Ppp1r18	Q8BQ30	EEAEAQSRPLR		-0,90	0,41
Akap5	D3YVF0	GPVAASPQKER	S(6): 100.0	-0,90	0,56
Marcks	P26645	GEATAERPGEAAVASSPSK	T(4): 0.0; S(15): 0.8; S(16): 98.3; S(18): 0.8	-0,92	0,04
Lmna	P48678	ASSHSSQSQGGGSVTK	S(2): 6.8; S(3): 6.8; S(5): 0.6; S(6): 85.1; S(8): 0.6; S(13): 0.0	-0,94	0,00
Cacna1b	A2AIS0	ASCEALYSEMDPEER	S(2): 100.0; Y(7): 0.0; S(8): 0.0	-0,94	0,02
Gpr123	Q8C4G9	ALPCPGADQPPYPK	Y(12): 100.0	-0,96	-0,34
Arhgap5	P97393	GSEEDPLLSPVETWK	S(2): 0.0; S(9): 100.0; T(13): 0.0	-0,97	0,68
Synpr	D3Z5Q8	QASDWHSLSTLQLSLGLYK	S(3): 10.1; S(7): 89.9; S(9): 0.0; S(14): 0.0	-0,98	0,05
Nucks1	Q80XU3	VVDYSQFQESDDADEDYGR	Y(4): 0.0; S(5): 0.0; S(10): 100.0; Y(17): 0.0	-1,02	0,13
Pclo	Q9QYX7	RSQEVTDFLAPLQTSSR	S(2): 100.0; S(15): 0.0; S(16): 0.0	-1,02	0,58
Vps26b	Q8C0E2	SMSHQAAIASQR	S(1): 0.2; S(3): 99.8; S(10): 0.0	-1,03	-0,25
Psd3	E9PUC5	ISNSSEFSAK	S(2): 0.0; S(4): 100.0; S(5): 0.0; S(8): 0.0	-1,04	0,81
Plau	P06869	EDSLAYHNDIALLK	S(3): 1.8; Y(6): 98.2	-1,06	1,52
Marcks	P26645	VNGDASPAAAEPGAK	S(6): 100.0	-1,08	-0,06
Pgm1	Q9D0F9	AIGGIILTASHNPGGPNGDFGIK	T(8): 9.7; S(10): 90.3	-1,09	0,55
Ppp1r12a	Q9DBR7	STGVSFWTQDSDENEQERQSDTEDGSSK	S(1): 0.0; T(2): 0.0; S(5): 0.0; T(8): 0.0; S(11): 0.0; S(20): 97.9; T(22): 2.1; S(26): 0.0; S(27): 0.0	-1,11	0,67
Reps2	A2AFI8	APSQAAESSPTK	S(3): 0.0; S(8): 0.3; S(9): 99.7	-1,14	0,51
Amigo1	Q80ZD8	RMSDPESVSSVFSDTPIVV	S(3): 88.2; S(7): 11.5; S(9): 0.0; S(10): 0.0; S(13): 0.2; T(15): 0.0	-1,15	1,36
Slc7a8	Q9QXW9	NHPGSDTSPEAEASSGGGGVALK	S(5): 5.2; T(7): 47.4; S(8): 47.4; S(14): 0.0; S(15): 0.0	-1,21	-0,20
Rltpr	Q3V3V9-2	LEAPPSPSLGSGLGSKPLPPYPTEPSSPER	S(6): 0.0; S(8): 0.0; S(11): 0.0; S(15): 0.0; S(26): 50.0; S(27): 50.0	-1,26	0,34
Slc23a2	Q9EPR4	SSLAETLDSTGSLDPQR	S(1): 0.0; S(2): 0.0; T(6): 0.0; S(9): 0.0; T(10): 0.0; S(12): 100.0	-1,30	-0,10
Basp1	Q91XV3	KAEGAGTEEEGTPKESEPQAAADATEVK	T(7): 0.1; T(12): 49.9; S(16): 49.9; T(25): 0.0	-1,30	1,22
2010300C02Rik	E9Q3M9	VFSQENVCDR	S(3): 100.0	-1,44	0,75
Marcks	P26645	AEDGAAPSPSSETPKK		-1,52	1,15
Cend1	Q9JKC6	ADPVLLNNHSNLKPAPTVPAAPSSPDATSEPK	S(10): 0.0; T(17): 0.0; S(23): 88.6; S(24): 11.4; T(28): 0.0; S(29): 0.0	-1,54	1,50
Sipa1l3	G3X9J0	SSSEITLSECDVEEPGDPR	S(1): 8.5; S(2): 8.5; S(3): 83.1; T(6): 0.0; S(8): 0.0	-1,57	0,30
Uncharacterized protein C15orf39 homolog	Q3TEI4	GAAYQAGGLGSPYLR	Y(4): 100.0; S(11): 0.0; Y(13): 0.0	-2,00	0,95
Ubap2l	Q80X50	STSAPQMSPGSSDNQSSSPQPAQQK	S(1): 0.0; S(3): 0.0; S(8): 4.2; S(11): 47.9; S(12): 47.9; S(16): 0.0; S(17): 0.0; S(18): 0.0	-2,04	1,03

Disease Models & Mechanisms • Supplementary information

Supplementary Table S2. Proteins up and down phosphorylated in the Cortex of Tg(*Dyrk1a*) mice and reverted by L41 treatment.

Gene name	Protein Group Accessions	Sequence	phosphoRS Site Probabilities	TgDyrk1a vs WT	TgDyrk1a +/-L41
Asap1	H3BL41	QASEDSNDLTPTLPETPVPLPR	S(3): 87.1; S(6): 12.9; T(10): 0.0; T(12): 0.0; T(16): 0.0	3,10	-1,42
Atp1a1	Q8VDN2	AVAGDASESALLK	S(7): 0.1; S(9): 99.9	2,52	-2,15
Avl9	Q80U56	DSLPSDSPPITVQPQANNR	S(2): 0.0; S(5): 0.7; S(7): 99.3; T(11): 0.0	2,25	-1,20
Ralgapb	E9Q0J2	TNSGISSASGGSTEPTTPDSERPAQALLR	T(1): 0.0; S(3): 0.0; S(6): 0.5; S(7): 0.5; S(9): 0.5; S(12): 0.5; T(13): 0.5; T(16): 48.7; T(17): 48.7; S(20): 0.0	1,96	-1,12
Ctnnb1	Q02248	TSMGGTQQQFVEGVR	T(1): 50.0; S(2): 50.0; T(6): 0.0	1,95	-1,76
Szrd1	A2AA86	ILGSASPEEEQEKPILDRPTR	S(4): 0.0; S(6): 100.0; T(20): 0.0	1,94	-1,43
Strip1	Q8C079	AASPPASASDLIEQQQK	S(3): 99.1; S(7): 0.4; S(9): 0.4	1,85	-1,57
Fam117b	Q3U3E2	NGSPTPAGALAGGAVGPPGGPGSR	S(3): 99.4; T(5): 0.6; S(23): 0.0	1,85	-1,40
Atp1a2	Q6PIC6	CIELSSGSVK	S(5): 0.3; S(6): 94.3; S(8): 5.3	1,77	-1,85
Gpkow	Q53YU8	VEDLNTFGPK	T(6): 100.0	1,66	-1,43
Ralgapb	E9Q0J2	TNSGISSASGGSTEPTTPDSERPAQALLR	T(1): 0.0; S(3): 0.0; S(6): 0.0; S(7): 0.0; S(9): 0.0; S(12): 0.0; T(13): 0.0; T(16): 3.6; T(17): 95.5; S(20): 0.8	1,40	-0,62
Gm996	A2AJA9	ASQGLATAPASPPVLQR	S(2): 0.0; T(7): 0.0; S(11): 100.0	1,38	-0,52
Ap2a1	P17426-2	RDTSSNDINGGVEPTPSTVSTPSPSADLLGLR	T(3): 0.0; S(4): 0.0; S(5): 0.0; T(15): 0.0; S(17): 0.0; T(18): 0.0; S(20): 0.6; T(21): 0.6; S(23): 98.3; S(25): 0.6	1,37	-0,78
Atp13a1	Q9EPB4	EIHPYLVMDLEQS	Y(5): 0.0; S(13): 100.0	1,31	-0,30
Sipa1l1	Q8C0T5	LIDLESPTPESQK	S(6): 99.7; T(8): 0.3; S(11): 0.0	1,30	-1,29
Setd1b	Q8CFG0	FGGGGGSRALSNLVPK	S(7): 0.0; S(11): 100.0	1,27	-0,16
Ywhaz	P63101	TAFDEAIAELDTLSEESYK	T(1): 0.0; T(12): 0.0; S(14): 100.0; S(17): 0.0; Y(18): 0.0	1,19	-1,35
Ovos	Q3UST5	AGQMANTLSALLQK	T(7): 9.7; S(9): 90.3	1,15	-1,45
Anln	Q8K212	DTTSPMELAALEK	T(2): 33.3; T(3): 33.3; S(4): 33.3	1,11	-1,67
Fmnl1	A2AB60	GPGDVVSIEILPGAAATPSGDDAQAPR	S(7): 0.0; T(17): 92.6; S(19): 7.4	1,09	-0,20
Sirt2	Q8VDQ8-2	EHANIDAQSGSQAPNPSTTISPGK	S(9): 0.0; S(11): 0.0; S(17): 0.0; T(18): 0.0; T(19): 0.0; S(21): 100.0	1,09	-0,55
Lmtk3	Q5XJV6	ALVNGEPMSPEAGEK	S(9): 100.0	1,08	0,12
Arhgap33	Q80YF9	AQQVAEQQSQQEQGGTPPAPHSPFR	S(9): 0.0; T(16): 100.0; S(22): 0.0	1,07	-0,55
Atp1a3	Q6PIC6	VDNSSLTGESEPQTRSPDCTHDNPLETR	S(4): 16.7; S(5): 16.7; T(7): 16.7; S(10): 16.7; T(14): 16.7; S(16): 16.7; T(20): 0.0; T(27): 0.0	1,07	-0,01
Dhx16	Q04690-2	SSSGLATYSPPMGAVSER	S(1): 0.0; S(2): 0.0; S(3): 0.0; T(7): 0.0; Y(8): 6.8; S(9): 93.2; S(16): 0.0	1,04	-1,81
Map1a	Q9QYR6-2	VAELEEEQSQGSSSYSDWVK	S(9): 100.0; S(12): 0.0; S(13): 0.0; S(14): 0.0; Y(15): 0.0; S(16): 0.0	0,98	-0,61
Trim46	D3YXA6	EVLGQQGYIGHGGDPSSEPTSPASTPSTR	Y(8): 0.0; S(16): 0.0; S(17): 0.0; T(20): 48.8; S(21): 48.8; S(24): 1.0; T(25): 1.0; S(27): 0.2; T(28): 0.2	0,97	-0,75
Pde10a	Q8CA95-3	LTECFLSPSLTDEK	T(2): 0.0; S(7): 100.0; S(9): 0.0; T(11): 0.0	0,92	-0,43
Snx27	Q3UHD3-2	LTLQDQVDTLTFQSQSLR	T(2): 0.0; T(9): 33.3; T(11): 33.3; S(14): 33.3; S(16): 0.0	0,88	-3,09
Vdac1	Q60829	ATLSEPGEEPQHPSPP	T(2): 0.0; S(4): 0.0; S(14): 100.0	0,88	-0,13
Map2	P20357	ETSPETSLIQDEVALK	T(2): 47.5; S(3): 47.5; T(6): 2.5; S(7): 2.5	0,87	-1,31
Sec22b	O08547	NLGSINTELQDVQR	S(4): 93.0; T(7): 7.0	0,87	0,02
Crmp1	P97427	SIPHITSDR	S(1): 100.0; T(6): 0.0; S(7): 0.0	0,87	-0,24
Syne1	Q6ZWQ5	RYSDFEWLK	Y(2): 0.0; S(3): 100.0	0,86	-0,07
Bod1l	E9Q6J5	GSDDVLVSGEVPECEVGHMSPR	S(2): 0.0; S(8): 0.0; S(20): 100.0	0,86	-0,91
8430427H17Rik	Q3TXS7	TVGTPIASVPGSTNTGTVPGSEK	T(1): 14.7; T(4): 85.1; S(8): 0.1; S(12): 0.0; T(13): 0.0; T(15): 0.0; T(17): 0.0; S(21): 0.0	0,82	0,07
Ctps1	P70698	SGSSSPDSEITELK	S(1): 0.0; S(3): 0.0; S(4): 0.0; S(5): 0.2; S(8): 99.8;	0,82	-1,17

Disease Models & Mechanisms • Supplementary information

			T(11): 0.0		
Wdr20a	Q3UWE6	RNSTDSRPVSVTYR	S(3): 50.0; T(4): 50.0; S(6): 0.0; S(10): 0.0; T(12): 0.0; Y(13): 0.0	0,81	-0,76
Map7	D3YWN7	LSSSSATLLNSPDR	S(2): 0.0; S(3): 0.0; S(4): 0.0; S(5): 0.0; T(7): 0.0; S(11): 100.0	0,81	-0,35
Tnik	B2RQ80	KNSPGNGSALGPR	S(3): 99.5; S(8): 0.5	0,80	-0,99
Ap4e1	Q80UK0	FNGSDGGVSWSPMDDELLAQPQVMK	S(4): 1.9; S(9): 49.0; S(11): 49.0	0,79	-0,47
Ubap2l	Q80WJ7	SETNWESPK	S(1): 0.0; T(3): 0.0; S(7): 100.0	0,78	-0,02
Map1a	Q9QYR6-2	ELALSSPEDLTQDFEELK	S(5): 4.0; S(6): 96.0; T(11): 0.0	0,77	-0,46
Ablim3	Q69ZR9-2	DAQSPLLEVDAASVK	S(4): 100.0; S(13): 0.0	0,76	-0,67
Sgip1	Q8VD37	LPSISDLDSIFGPVLSPK	S(3): 0.0; S(5): 0.0; S(9): 0.0; S(16): 100.0	0,76	-0,15
Lrfn1	Q2TUM3-3	DGALFSFILDFLR	S(6): 100.0	0,75	-0,98
Pclo	Q9QYX7	SEGITKPSLQQPSPK	S(1): 0.0; T(5): 0.0; S(8): 0.0; S(13): 100.0	0,74	-0,80
Mycbp2	F6SMY7	QVSTENDSTLVHR	S(3): 50.0; T(4): 50.0; S(8): 0.0; T(9): 0.0	0,74	-0,27
Chga	P26339	GELEHSQQEEDGEEAMVGTPQGLFPQGGK	S(6): 100.0; T(19): 0.0	0,73	-0,52
Dync1li1	Q8R1B4	DFESHITNYK	S(4): 0.0; T(7): 0.4; Y(9): 99.6	0,72	-0,06
Recql4	Q71M36	TPSELHNDNFSLSTIAEGSHPNVR	T(1): 49.4; S(3): 0.4; S(11): 49.4; S(13): 0.4; T(14): 0.4; S(19): 0.0	0,72	-0,75
Cacna1b	A2AIR9	EVAEVSPMSAANISIAAR	S(6): 99.9; S(9): 0.1; S(14): 0.0	0,71	-0,18
Maoa	Q640R3	QNSLEYMDQNDDRLK	S(3): 99.7; Y(6): 0.3	0,70	-0,26
Agap2	Q3UHD9	AISAFGPSASINGLVK	S(3): 0.0; S(8): 50.0; S(10): 50.0	0,70	-0,23
Npm1	Q61937	CGSGPVHISGQHLVAVEEDAESEDEDEEDVK	S(3): 0.0; S(9): 0.0; S(22): 100.0	0,69	-0,32
Mkl1	D3YUI2	FGSTGSTPPVSPTPSER	S(3): 0.0; T(4): 0.0; S(6): 0.7; T(7): 99.3; S(11): 0.0; T(13): 0.0; S(15): 0.0	0,68	-0,67
Lgalsl	Q8VE70	FLQTIK	T(4): 100.0	0,68	-0,68
Diras2	Q5PR69-2	ESSDNQPPSPSLPEELK	S(2): 0.2; S(3): 0.2; S(9): 99.5; S(11): 0.0	0,68	-0,29
Rmdn3	Q3UJU9	SHSLPNSLDYAQASER	S(1): 0.0; S(3): 100.0; S(7): 0.0; Y(10): 0.0; S(14): 0.0	0,68	-0,58
Dopey1	Q8BL97-4	VYVGNLGTGAGK	Y(2): 100.0; T(8): 0.0	0,67	0,02
Kcnd1	Q03717	STSSLPPEPMEIVR	S(1): 0.0; T(2): 0.2; S(3): 9.9; S(4): 90.0	0,67	-1,04
Lpin2	Q99P72	ESAEFSVLEYSEMGSSFNGSPK	S(2): 0.0; S(6): 0.0; Y(10): 0.0; S(11): 0.0; S(15): 0.0; S(16): 6.7; S(20): 93.3	0,67	-0,31
Ppp1r9b	Q6R5C6	NQALEMLSR	S(8): 100.0	0,66	-0,42
Mark2	E9QMP6	RSSDQAVPAIPTSNSYSK	S(2): 50.0; S(3): 50.0; T(12): 0.0; S(13): 0.0; S(15): 0.0; Y(16): 0.0; S(17): 0.0	0,66	-0,44
Apc	B2RUG9	QSVGSGSPVQTVGLETR	S(2): 0.0; S(5): 0.4; S(7): 99.5; T(11): 0.0; T(16): 0.0	0,66	-0,39
Lrrc7	Q80TE7	SQSIDEIDVGTYK	S(1): 3.2; S(3): 96.8; T(11): 0.0; Y(12): 0.0	0,66	-0,85
Ppp1r1b	Q60829	AVQHLQTISNLSENQASEEEDELGELR	T(7): 0.0; S(9): 0.0; S(12): 0.0; S(17): 100.0	0,65	-0,01
Synj1	E9Q7S0	TPGPPSSQGSPVDTQPAAQK	T(1): 0.0; S(6): 0.7; S(7): 7.6; S(10): 91.7; T(14): 0.0	0,65	-0,42
Pnck	Q9QYG0	TASLTSAASIDGSR	T(1): 0.0; S(3): 0.0; T(5): 0.1; S(6): 0.1; S(9): 98.6; S(13): 1.1	0,64	-0,62
Dos	E9QP85	HASLDGASPYFK	S(3): 100.0; S(8): 0.0; Y(10): 0.0	0,63	-0,12
Lppr4	Q7TME0	QTYELNDLNR	T(2): 50.0; Y(3): 50.0	0,63	-0,33
Gdi1	P50396	MAGSAFDFENMK	S(4): 100.0	0,62	-0,08
Iqsec2	E9Q8N8	MFSNPDNGSPAMTHR	S(3): 0.0; S(9): 100.0; T(13): 0.0	0,61	-0,74
Syngap1	F6SEU4	DLNSSIDLQSFMAR	S(4): 0.3; S(5): 99.7; S(10): 0.0	0,61	-0,13
Col3a1	P08121	GENGSPGAPGAPGHPGPPGPVGPSGK	S(5): 100.0; S(24): 0.0	0,60	-0,19
Pik3c3	E9QLS6	DGDESSPILTSFELVK	S(5): 4.8; S(6): 95.2; T(10): 0.0; S(11): 0.0	0,59	-0,40
Rab3a	P63011	MSESLDTADPAVTGAK	S(2): 90.3; S(4): 4.8; T(7): 4.8; T(13): 0.0	0,59	-0,25
Hrh3	E9Q7T5	VQPHGSLEQCWK	S(6): 100.0	0,59	-0,13
Ndel1	Q9EQG9	SSSMSSIDLVSASDDVHR	S(1): 0.3; S(2): 49.9; S(3): 49.9; S(5): 0.0; S(6): 0.0; S(11): 0.0; S(13): 0.0	0,58	-0,29
Dbnl	Q62418-3	VVTEKSPTDWALFTYEGNSNDIR	T(3): 33.3; S(6): 33.3; T(8): 33.3; T(14): 0.0; Y(15): 0.0; S(19): 0.0	0,58	0,15
D430041D05Rik	D3YZ21	QSLNSPSPGETEMDLLVTR	S(2): 0.0; S(5): 100.0; S(7): 0.0; T(11): 0.0; T(18): 0.0	0,58	-0,10
Atxn2l	Q7TQH0-2	EVDGLLTSDPMGSPVSSK	T(7): 0.0; S(8): 0.1; S(13): 98.7; S(16): 1.1; S(17): 0.1	0,58	-0,70

Disease Models & Mechanisms • Supplementary information

Prpf31	Q8CC35-2	ASPAAAEEAVPEWASCLK	S(2): 100.0; S(15): 0.0	0,58	-0,83
Rap1gap2	Q5SSL4	SQGGGDSVSPTPPEGLAPGVEAGK	S(1): 0.0; S(7): 0.1; S(9): 90.7; T(11): 9.2	0,58	-1,13
Cadm4	Q8R3Y8-2	AGGASPAASSTTQPPAQHR	S(5): 100.0; S(9): 0.0; S(10): 0.0; T(11): 0.0; T(12): 0.0	0,58	-0,40
Uqcrh	P99028	SQTEEDCTEELFDFLHAR	S(1): 8.2; T(3): 91.8; T(8): 0.0	0,57	0,07
Map1a	Q9QYR6-2	QLSPESLGTLQFGELSLGK	S(3): 88.6; S(6): 5.7; T(9): 5.7; S(16): 0.0	0,57	-0,05
Fubp1	Q3TSG4	RGSFSSENYWR	S(3): 100.0; S(5): 0.0; S(6): 0.0; Y(9): 0.0	0,57	-0,54
Samd14	Q8K031	SLSIESLCPDEGR	S(1): 0.1; S(3): 99.9; S(6): 0.1	0,57	-0,10
Camk2d	E9Q1T1	ENFSGGTSLWQNI	S(4): 88.3; T(7): 5.8; S(8): 5.8	0,57	0,12
Cspg5	Q71M36	TPSELHNDNFSLSTIAEGSHPNVR	T(1): 99.4; S(3): 0.6; S(11): 0.0; S(13): 0.0; T(14): 0.0; S(19): 0.0	0,57	-0,76
Usp8	Q80U78-2	SMDELNHDFQALALEGR	S(1): 100.0	0,56	-0,73
Pnpo	Q91WH7	VDNSSLTGESEPQTRSPECTHESPLETR	S(4): 0.0; S(5): 0.0; T(7): 0.2; S(10): 0.7; T(14): 77.4; S(16): 15.3; T(20): 3.2; S(23): 3.2; T(27): 0.0	0,56	-0,12
Tmem163	Q8C996	GHAPSTAAPAPSPAPMSSSVQSDEER	S(5): 0.0; T(6): 0.0; S(12): 0.0; S(17): 0.0; S(18): 0.0; S(19): 0.0; S(22): 100.0	0,56	-0,35
Map1b	P14873	TPEEGGYSYEISEK	T(1): 100.0; Y(7): 0.0; S(8): 0.0; Y(9): 0.0; S(12): 0.0	0,56	-0,01
Npat	Q8BL99-5	ASSPDDDVQQVVFDLICK	S(2): 4.7; S(3): 95.3	0,56	-1,43
Ifi205a	Q8CGA3	LCLSTVDLEVK	S(4): 2.8; T(5): 97.2	0,56	-0,65
Tpp2	Q64511	FDSNEEDTASVFAPSFGLK	S(3): 100.0; T(8): 0.0; S(10): 0.0; S(15): 0.0	0,56	-0,23
Ctnnd2	E9QKH8	TGSQHGPQNAAAATFQR	T(1): 8.4; S(3): 91.6; T(14): 0.0	0,55	-0,97
Dlgap2	Q8BII1	IQPQAGVPLGNSTLARPLDSPMCPVSPR	S(12): 50.0; T(13): 50.0; S(20): 0.0; S(26): 0.0	0,55	-0,37
Tjp1	B9EHJ3	IDSPGLKPASQQVYR	S(3): 100.0; S(10): 0.0; Y(14): 0.0	0,55	-0,28
Gm15800	E9Q2E4	SMSAPSDLEMIGNEDLEFTR	S(1): 47.3; S(3): 47.3; S(6): 5.4; T(19): 0.0	0,54	-1,11
Synj1	E9Q7S0	TPGPPSSQGSPVDTQPAAQK	T(1): 0.0; S(6): 1.3; S(7): 10.2; S(10): 87.2; T(14): 1.3	0,54	-0,78
Slc9a6	A1L3P4	LVLPMDDSEPALNSLDDTRHSPA	S(8): 0.0; S(14): 0.0; T(18): 11.0; S(21): 89.0	0,54	-0,49
Uncharacterized protein C15orf39 homolog	Q3TEI4	GAAYQAGGLGSPYLR	Y(4): 83.6; S(11): 13.8; Y(13): 2.6	0,54	-0,57
Cep170b	Q80U49	GASPVTPSTTPPPPTDPQLTK	S(3): 0.0; T(6): 0.0; S(8): 0.0; T(9): 0.0; T(10): 0.4; T(15): 99.6; T(20): 0.0	0,54	0,18
Fam117b	Q3U3E2	TSPTVATQTGASVTSTR	T(1): 50.0; S(2): 50.0; T(4): 0.0; T(7): 0.0; T(9): 0.0; S(12): 0.0; T(14): 0.0; S(15): 0.0; T(16): 0.0	0,53	-0,09
Arhgap21	B7ZCJ1	DQGEPVSPSEDEPFSWPGPK	S(7): 91.8; S(9): 8.2; S(15): 0.0	0,53	-0,67
Ank2	Q8C878	TLYLQSVTSIEER	T(1): 0.1; Y(3): 0.1; S(6): 49.3; T(8): 49.3; S(9): 1.2	0,52	-0,43
Lgalsl	Q8VED9	LDDGHLNNSLGSPVQADVYFPR	S(9): 0.0; S(12): 100.0; Y(19): 0.0	0,52	-0,30
Atp1a1	Q8VDM4	DKTPVQSQQPSATTPSGADEK	T(3): 0.0; S(7): 0.0; S(11): 0.0; T(13): 6.2; T(14): 93.8; S(16): 0.0	0,52	0,15
Bnc2	H3BLG6	ATSGAATPVIASTK	T(2): 0.0; S(3): 0.0; T(7): 33.3; S(12): 33.3; T(13): 33.3	0,52	0,00
Pcm1	Q9R078	CSDVSELSSSPPGPYHQEPYMSKPEER	S(2): 0.0; S(5): 0.0; S(8): 0.3; S(9): 49.8; S(10): 49.8; Y(15): 0.0; Y(20): 0.0; S(22): 0.0	0,52	-0,37
4932429P05Rik	A2ADI4	YNMLNSAIIELFEFIR	Y(1): 0.0; S(6): 100.0	0,52	-0,19
Begain	F8WIG2	GDIYCSDPALYCPDER	Y(4): 0.0; S(6): 100.0; Y(11): 0.0	0,52	0,26
Pygo2	Q3V0I2	ALELEPLELEGSLAGSPPGLAPPPPPHR	S(12): 0.0; S(16): 100.0	0,51	0,03
Fbxl20	Q9CZP3	EQDLQVFDLESK	S(11): 100.0	0,51	-0,73
Srcin1	B1AQX6	GEPDPERSSPPMLSADDAEYPR	S(8): 88.5; S(9): 11.4; S(14): 0.0; Y(20): 0.0	0,51	-0,08
Mag	P20917	GESPELDLSYSHSDLGK	S(3): 100.0; S(9): 0.0; Y(10): 0.0; S(11): 0.0; S(13): 0.0	0,51	0,19
Adcy5	P84309	APPAGGSGSSAAAAAAAGGTEVRPR	S(7): 0.7; S(9): 49.7; S(10): 49.7; T(20): 0.0	0,51	-0,33
Brsk1	Q5RJI5	MQVPTAEEMSSLTPESSPELAK	T(5): 0.0; S(10): 0.0; S(11): 0.0; T(13): 0.0; S(16): 50.0; S(17): 50.0	0,51	-0,09
Pkn2	G3UZM9	EDVSNFDDEFTSEAPILTPPREPR	S(4): 25.0; T(11): 25.0; S(12): 25.0; T(18): 25.0	0,50	-0,97
Trim46	D3YXA6	EVLGQQGYIGHGGDPSSEPTSPASTPSTR	Y(8): 0.0; S(16): 0.0; S(17): 0.0; T(20): 0.8; S(21): 97.5; S(24): 0.8; T(25): 0.8; S(27): 0.0; T(28): 0.0	0,50	-0,78
Lppr2	Q8VCY8-2	LSPWEDLSQAPTMDSPLEK	S(2): 0.0; S(8): 0.0; T(12): 0.0; S(15): 100.0	0,50	-0,12
2010300C02Rik	E9Q3M9	LQHSMAVSASMEEGGCPR	S(4): 0.0; S(8): 0.0; S(10): 100.0	0,50	-0,01
Camsap2	Q8C1B1	RFSPSQVPIQTR	S(3): 100.0; S(5): 0.0; T(11): 0.0	0,50	-0,32

Disease Models & Mechanisms • Supplementary information

Gprin1	Q3UNH4	AETVSPGEVDAMTLGK	T(3): 0.3; S(5): 99.7; T(13): 0.0	0,49	0,00
Hspb1	P14602-3	QLSSGVSEIR	S(3): 100.0; S(4): 0.0; S(7): 0.0	0,49	-0,17
Myo9b	E9QKV6	AQDKPESPSGSTQIQR	S(7): 100.0; S(9): 0.0; S(11): 0.0; T(12): 0.0	0,49	-0,35
C2cd2l	Q80X80	DEEQPELSTVEELIK	S(8): 6.0; T(9): 94.0	0,49	0,09
Lppr4	Q7TME0	MSLQVMDTEPEGQSPPR	S(2): 0.0; T(8): 0.0; S(14): 100.0	0,49	-0,45
Adcy9	P51830	VQVDGSIGRSPTDEIANLVPSVQYSDK	S(6): 33.3; S(10): 33.3; T(12): 33.3; S(21): 0.0; Y(24): 0.0; S(25): 0.0	0,49	-0,41
Epn1	Q80V94	IIEQPECSSPVIETER	S(8): 50.0; S(9): 50.0; T(14): 0.0	0,48	-0,42
Prl8a1	Q9DA80	VSSQPAPSQER	S(2): 50.0; S(3): 50.0; S(8): 0.0	0,48	-0,01
Hdac4	Q6NXL1	VLPVYMNSLLK	Y(5): 100.0; S(8): 0.0	0,48	0,30
Mark1	Q8VHG2	DTTVISHSPNTSYDTALEAR	T(2): 0.0; T(3): 0.0; S(6): 0.0; S(8): 100.0; T(11): 0.0; S(12): 0.0; Y(13): 0.0; T(15): 0.0	0,48	-0,49
Mtus2	Q3UHD1	NENVATLSVSSLER	T(6): 0.0; S(8): 0.0; S(10): 0.3; S(11): 99.7	0,48	-0,12
Rtn1	Q8K0T0	GSVSEDELIAAIK	S(2): 3.4; S(4): 96.6	0,48	-0,53
Syn1	O88935	QSRPVAGGPGAPPAARPPASPSPQR	S(2): 0.0; S(20): 50.0; S(22): 50.0	0,47	-0,29
Mllt6	B1AR09	HSSGGGGGAGGGSSGGGGGGSSSASGGGGGTGGGSGNSFLSGR	S(2): 2.4; S(3): 3.7; S(13): 6.3; S(14): 6.3; S(21): 23.3; S(22): 23.3; S(23): 11.6; S(25): 11.6; T(31): 3.7; S(35): 3.7; S(38): 1.7; S(41): 2.4	0,47	0,28
Map1s	Q8C008	FTQGLEDQLSPPGNR	T(2): 0.0; S(10): 100.0	0,47	-0,51
Map6d1	Q149S1	LLSEVEELNMSLR	S(3): 2.5; S(11): 97.5	0,47	-0,73
Pcdh1	F7BJK1	SNSPLPSIQLQPQSPSASK	S(1): 0.0; S(3): 0.0; S(7): 0.0; S(14): 47.4; S(16): 47.4; S(18): 5.2	0,47	-0,20
Ttc7b	E9Q6P5	VEQALSEVASSLQSSAPK	S(6): 0.0; S(10): 6.4; S(11): 93.6; S(14): 0.0; S(15): 0.0	0,47	0,03
Tmx2	D3Z2J6	AVSWTFSEENVIR	S(3): 7.5; T(5): 91.9; S(7): 0.7	0,47	-0,72
Syn1	O88935	QSRPVAGGPGAPPAARPPASPSPQR	S(2): 0.0; S(20): 1.3; S(22): 98.7	0,46	-0,16
Aff4	Q9ES97-2	ETTGGAPTMSPDLEQEQLTIR	T(2): 0.0; T(3): 0.0; T(8): 1.6; S(10): 98.4; T(19): 0.0	-0,36	0,46
Dennd4b	Q3U1V8	GIDGLAVAYGVAMNK	Y(9): 100.0	-0,36	-0,17
Pycard	Q9EP53-4	GTPSGTPATSPPPAPPCPQDDCVHGSAAQASATAPR	T(2): 0.0; S(4): 0.0; T(6): 0.5; T(9): 49.7; S(10): 49.7; S(26): 0.0; S(31): 0.0; T(33): 0.0	-0,36	0,01
Nucks1	Q80XQ2	TISSSPSIESLPGGR	T(1): 0.0; S(3): 0.3; S(4): 1.8; S(5): 0.3; S(7): 95.8; S(10): 1.8	-0,36	-0,38
Kcnrg	Q2KN98	KGSSGNASEVSVACLTER	S(3): 99.4; S(4): 0.6; S(8): 0.0; S(11): 0.0; T(16): 0.0	-0,36	-0,47
Hspa4l	P48722	QLTQDLLNSYIENEGK	T(3): 100.0; S(9): 0.0; Y(10): 0.0	-0,36	0,36
Map1a	Q9QYR6-2	MASPPPSGPPSAAHTPFHQSPVEEK	S(3): 95.4; S(7): 2.3; S(11): 2.3; T(15): 0.0; S(20): 0.0	-0,36	0,22
Add1	Q9QYC0	SPPDQSAVPNTPPSTPVKLEEDLPQEPTSR	S(1): 0.0; S(6): 0.0; T(11): 97.8; S(14): 1.1; T(15): 1.1; T(28): 0.0; S(29): 0.0	-0,36	0,29
Atg2b	Q80X80	NLGTPTSSTPRPSITPTK	T(4): 99.9; T(6): 0.0; S(7): 0.0; S(8): 0.0; T(9): 0.0; S(13): 0.0; T(15): 0.0; T(17): 0.0	-0,37	-0,06
Top2b	Q64337	LTPTTPESSSTGTEDK	T(2): 88.8; T(4): 9.9; T(5): 1.2; S(8): 0.0; S(9): 0.0; S(10): 0.0; T(11): 0.0; T(13): 0.0	-0,37	-0,09
Rnmt	Q9D0L7	SAEDLTDGSYDDILNAEQLK	S(1): 32.2; T(6): 32.2; S(9): 3.4; Y(10): 32.2	-0,37	0,21
Gpsm1	Q6GYP7	HFSQSEDTGNEVFGALHEEQPLPR	S(3): 100.0; S(5): 0.0; T(8): 0.0	-0,37	-0,04
Tmsb4x	P20065-2	TETQEKNPLPSK	T(1): 0.0; T(3): 100.0; S(11): 0.0	-0,37	-0,36
Phkb	Q7TQH0-2	EVDGLLTSDPMGSPVSSK	T(7): 0.0; S(8): 0.0; S(13): 99.8; S(16): 0.2; S(17): 0.0	-0,38	0,27
Ppme1	Q8BVL3	VTSSVPLPSGGTSSPSR	T(2): 0.0; S(3): 0.0; S(4): 0.0; S(9): 0.5; T(12): 6.3; S(13): 80.5; S(14): 6.3; S(16): 6.3	-0,38	0,38
Tp53bp1	P70399	ASQEPFSPAEDVMETDLLEGLAANQDRPSK	S(2): 0.0; S(7): 1.4; T(15): 98.6; S(29): 0.0	-0,38	0,23
Sptbn1	Q62261	AQTLPTSVVTITSESSPGKR	T(3): 0.0; T(6): 0.0; S(7): 0.0; T(10): 0.1; T(12): 4.4; S(13): 4.4; S(15): 45.6; S(16): 45.6	-0,38	-0,09
Echdc1	Q9D8U8	SVSVDLNVDPSLQIDIPDALSER	S(1): 12.1; S(3): 87.9; S(11): 0.0; S(21): 0.0	-0,38	1,04
Ralgapa1	Q6DID3	ASEPVKEPVQTAQSPAPVEK	S(2): 0.0; T(11): 8.3; S(14): 91.7	-0,39	0,03
Usp10	P52479-2	TCDSPQNPVDFISGPVPDSPFPR	T(1): 11.1; S(4): 88.9; S(13): 0.0; S(19): 0.0	-0,39	0,41
Trim3	Q3B7Z2	GATVLPANPPGSAGSGK	T(3): 100.0; S(12): 0.0; S(15): 0.0	-0,39	-0,08
Map1a	Q9QYR6-2	EMTLDQKSPEK	T(3): 1.2; S(8): 98.8	-0,39	0,17

Disease Models & Mechanisms • Supplementary information

Map1b	P14873	TLEVVSPSQSVTGSAGHTPYYQSPTDEK	T(1): 0.0; S(6): 94.1; S(8): 0.8; S(10): 0.8; T(12): 0.8; S(14): 0.8; T(18): 0.8; Y(20): 0.8; Y(21): 0.8; S(23): 0.0; T(25): 0.0	-0,39	0,08
Gbe1	Q9D5W4	QEEYSQSLLK	Y(4): 7.3; S(5): 92.0; S(7): 0.7	-0,39	-0,33
Kif1b	Q60575-2	SHAVFTIVFTQK	S(1): 100.0; T(6): 0.0; T(10): 0.0	-0,40	0,46
Tbr1	Q64336	SKLSPVLDGVSELR	S(1): 0.0; S(4): 100.0; S(11): 0.0	-0,40	0,30
Srcin1	B1AQX9	EVSGPNETSSPGSEKPSGSR	S(3): 0.0; T(8): 33.3; S(9): 33.3; S(10): 33.3; S(13): 0.0; S(17): 0.0; S(19): 0.0	-0,40	-0,30
Atxn7l1	B2RW07	FALNSMVEK	S(5): 100.0	-0,41	0,18
Map1a	Q9QYR6-2	MASPPPSGPPSAAHTPFHQSPVEEK	S(3): 100.0; S(7): 0.0; S(11): 0.0; T(15): 0.0; S(20): 0.0	-0,41	0,46
Tenm4	Q3UHK1	SLLAAESAASLQGAELER	S(1): 0.0; S(7): 89.1; S(10): 10.9	-0,41	-0,03
Dpysl5	Q9EPR4-2	SSLAETLDSTGSLDPQR	S(1): 0.0; S(2): 0.0; T(6): 0.1; S(9): 0.7; T(10): 49.6; S(12): 49.6	-0,41	-0,28
Scin	Q60598	QTPPASPSPQPIEDRPPSSPIYEDAAPFK	T(2): 0.0; S(6): 0.2; S(8): 99.8; S(18): 0.0; S(19): 0.0; Y(22): 0.0	-0,41	-0,09
Map1a	Q9QYR6-2	APISLSQDPSPPLNGSTTSCGPDR	S(4): 0.0; S(6): 0.0; S(10): 100.0; S(16): 0.0; T(17): 0.0; T(18): 0.0; S(19): 0.0	-0,42	0,16
Map1a	Q9QYR6-2	EMTLDQKSPEK	T(3): 0.1; S(8): 99.9	-0,42	0,14
Larp1	J3QNB1	SLPTTVPESPNYR	S(1): 0.0; T(4): 0.1; T(5): 0.1; S(9): 97.3; Y(12): 2.5	-0,43	-0,22
Supt3	Q8WTY4	EALSPEEVQSVQEHLGYHSDSLR	S(4): 100.0; S(10): 0.0; Y(17): 0.0; S(19): 0.0; S(21): 0.0	-0,43	0,08
Map1b	P14873	DYNASASTISPPSSMEEDKFSK	Y(2): 0.0; S(5): 0.0; S(7): 0.6; T(8): 0.6; S(10): 98.8; S(13): 0.0; S(14): 0.0; S(21): 0.0	-0,43	-0,24
C2cd2l	Q80X50	RYPSSISSSPQK	Y(2): 0.0; S(4): 6.1; S(5): 0.5; S(7): 0.5; S(8): 6.1; S(9): 86.9	-0,43	0,17
Synpo	Q8CC35-2	DRASPAAAEEAVPEWASCLK	S(4): 100.0; S(17): 0.0	-0,43	0,55
Trappc8	E9PWG2	NSDSNLLSLDGLDNEVK	S(2): 50.0; S(4): 50.0; S(8): 0.1	-0,43	0,26
Gprin3	Q8BVY4	IAQDLINSIDQTGELLAMFEDNELDDVK	S(8): 50.0; T(12): 50.0	-0,43	0,76
Astn2	E9Q8T4	TGAAAGAGAGTGAGAGAAAAAASAASPGSAGSAGTAAESR	T(1): 0.0; T(11): 0.5; S(23): 7.1; S(26): 59.9; S(29): 7.1; S(32): 20.0; T(35): 2.7; S(39): 2.7	-0,43	0,68
Eml2	E9QK48	VGGYATSPSSPK	Y(4): 0.0; T(6): 0.3; S(7): 33.2; S(9): 33.2; S(10): 33.2	-0,44	2,07
Otud7a	Q8R550	SIEVENDFLPVEK	S(1): 100.0	-0,44	0,44
Htt	P42859	SGSIVELLAGGGSSCSPVLSR	S(1): 50.0; S(3): 50.0; S(13): 0.0; S(14): 0.0; S(16): 0.0; S(20): 0.0	-0,44	0,54
Map1b	P14873	LGGDVSPTQIDVSQFGSFK	S(6): 50.0; T(8): 50.0; S(13): 0.0; S(17): 0.0	-0,45	0,04
Dlg4	Q62073	SIQDLTVTGTEPGQVSSR	S(1): 100.0; T(6): 0.0; T(8): 0.0; T(10): 0.0; S(16): 0.0; S(17): 0.0	-0,45	-0,15
Magi2	Q9WVF8	RGSMFYDEDGDLAHEFYEETIVTK	S(3): 90.5; Y(6): 9.5; Y(17): 0.0; T(20): 0.0; T(23): 0.0	-0,46	-0,14
Snap91	Q61423	FYYSEEDHGDGCSYTDLLPQDDGGGGGYSSVR	Y(2): 2.2; Y(3): 11.3; S(4): 11.3; S(13): 61.8; Y(14): 11.3; T(15): 2.2; Y(28): 0.0; S(29): 0.0; S(30): 0.0	-0,46	-0,46
Pnn	O35691	SLSPGKENINSQEVEK	S(1): 0.3; S(3): 99.7; S(11): 0.0	-0,46	0,36
Zzef1	Q5SRX1	AAETVPDLPSPPTEAPAPASNTSTR	T(4): 0.0; S(10): 38.5; T(13): 38.5; S(20): 1.4; T(22): 7.2; S(23): 7.2; T(24): 7.2	-0,47	-0,01
Syt5	Q9R0L6	VTNAISPESSPGVGR	T(2): 0.0; S(6): 100.0; S(9): 0.0; S(10): 0.0	-0,47	-0,09
Plxnb3	Q9QXY6	DKPMYDEIFYTLSPVDGK	Y(5): 0.0; Y(10): 0.0; T(11): 0.0; S(13): 100.0	-0,47	0,09
Aldoc	P05063	GILAADESVGSMAK	S(8): 100.0; S(11): 0.0	-0,49	0,08
Lsm14a	Q8K2F0	SESPPPLSEPK	S(1): 0.0; S(3): 100.0; S(8): 0.0	-0,49	0,37
Dnahc8	Q91XM9-7	NLSQIENVHGYVLQSHISPLK	S(3): 0.0; Y(11): 0.0; S(15): 1.0; S(18): 99.0	-0,50	0,50
Zc3hc1	H3BKM2	SMGTGDSAGVEVPSSPLR	S(1): 0.0; T(4): 0.0; S(7): 0.0; S(14): 50.0; S(15): 50.0	-0,50	0,24
Slc1a4	O35874	SEEETSPLVTHQNPAGPVAIAPELESK	S(1): 0.0; T(5): 0.7; S(6): 99.3; T(10): 0.0; S(26): 0.0	-0,51	0,34
Aatk	Q80Y17	DIILAPESCEGSPSSAHSK	S(8): 0.0; S(12): 93.2; S(14): 6.3; S(15): 0.5; S(18): 0.0	-0,51	0,26
Bora	Q8BRF7	VNLEESTGVENSPAGARPK	S(6): 0.0; T(7): 0.0; S(12): 100.0	-0,53	1,06
Usp45	E9Q4D8	ISLSSAPQLEPLVVELSSPGPLTSALFL	S(2): 69.4; S(4): 15.3; S(5): 15.3; S(17): 0.0; S(18): 0.0; T(23): 0.0; S(24): 0.0	-0,53	0,31
Gpr162	Q3UMB5	VASPANVGTLHTLSR	S(3): 100.0; T(9): 0.0; T(12): 0.0; S(14): 0.0	-0,54	-0,06
Trank1	Q8BTI8	SGTPPRPGSVTNMQADECTATPQR	S(1): 0.0; T(3): 100.0; S(9): 0.0; T(11): 0.0; T(19): 0.0; T(21): 0.0	-0,54	-0,01
Rem2	E9Q4D5	QASPLGTPTPEADTTLLK	S(3): 100.0; T(7): 0.0; T(9): 0.0; T(14): 0.0; T(15): 0.0	-0,54	-0,06

Disease Models & Mechanisms • Supplementary information

Abca2	A2AJ26	QPAEPGTSQEPGLASSPSGCPR	T(7): 0.3; S(8): 0.3; S(15): 49.5; S(16): 49.5; S(18): 0.3	-0,55	0,61
Twf1	Q91YM2	AGSPLCNSNLQDSEEDVEPPSYHLFR	S(3): 100.0; S(8): 0.0; S(13): 0.0; S(21): 0.0; Y(22): 0.0	-0,55	-0,08
Mtfr1l	Q9CW07	SFSLPADPILQAAK	S(1): 3.8; S(3): 96.2	-0,56	0,09
Ndrg1	Q62419	EASSRPK	S(3): 93.4; S(4): 6.6	-0,56	-0,33
Yap1	P46938-2	GDSETDLEALFNAVMNPK	S(3): 50.0; T(5): 50.0	-0,57	0,25
Arhgap32	Q810B9	LFEDSGGNSGGSGGGGRPTLSSPEK	S(5): 0.0; S(9): 0.3; S(12): 0.3; T(19): 33.1; S(21): 33.1; S(22): 33.1	-0,58	-0,25
Cep170b	Q80TZ3	GASSPDMEPSYGGGLFDMVK	S(3): 6.9; S(4): 93.1; S(10): 0.0; Y(11): 0.0	-0,58	0,39
Pml	D3YXR5	ATSPPHLDGTSNPESTVPEK	T(2): 9.6; S(3): 90.4; T(10): 0.0; S(11): 0.0; S(15): 0.0; T(16): 0.0	-0,58	-0,21
Camsap2	Q8C0T7	SEGTLQGAATTSTARR	S(1): 0.0; T(4): 0.1; T(10): 98.7; T(11): 1.1; S(12): 0.0; T(13): 0.0	-0,58	0,15
Mark3	Q02357-6	LNTPPPLAEEEGLASR	T(3): 100.0; S(15): 0.0	-0,58	0,33
Gm15800	E9Q2E4	SMSAPSDLEMIGNEDLEFTR	S(1): 49.9; S(3): 49.9; S(6): 0.1; T(19): 0.0	-0,59	0,39
Cend1	Q9JK24	EESRAVFLQR	S(3): 100.0	-0,59	-0,36
Sptbn2	Q68FF6	SMDSSDLSDGAVTLQEYLELK	S(1): 99.9; S(4): 0.0; S(5): 0.0; S(8): 0.0; T(13): 0.0; Y(17): 0.0	-0,60	-0,11
Trappc12	Q8K2F8	KSPTMEQAVQTASAHLPAPAPVGR	S(2): 99.4; T(4): 0.6; T(11): 0.0; S(13): 0.0	-0,60	0,08
Map1b	P14873	DYNASASTISPPSSMEEDK	Y(2): 0.0; S(5): 0.0; S(7): 0.0; T(8): 0.0; S(10): 100.0; S(13): 0.0; S(14): 0.0	-0,61	0,68
Adrbk2	Q3UYC0	RSSLPNGEGLQLK	S(2): 0.2; S(3): 99.8	-0,61	0,21
Gm15800	E9Q2E4	TEGTPPPPGQPAK	T(1): 0.0; T(4): 100.0	-0,62	0,10
Ehd3	Q9QXY6	DKPMYDEIFYTLSPVDGK	Y(5): 0.0; Y(10): 0.1; T(11): 8.1; S(13): 91.9	-0,62	-0,42
Map2	P20357	YTVPLPSPVQDSENLSGESGSFYEGTDDK	Y(1): 0.0; T(2): 0.0; S(7): 99.7; S(12): 0.2; S(16): 0.0; S(19): 0.0; S(21): 0.0; Y(23): 0.0; T(26): 0.0	-0,62	0,10
Rgs6	F6WDS8	SVYGVTDETQSQSPVHIPSQPIR	S(1): 0.1; Y(3): 0.1; T(6): 2.4; T(9): 2.4; S(11): 13.6; S(13): 81.4; S(19): 0.0	-0,62	-0,03
Rbm25	B2RY56	LGASNSPGQPNSVK	S(4): 1.3; S(6): 98.7; S(12): 0.0	-0,63	0,00
Cttn	Q60575-2	GDSLILEHQWELEK	S(3): 100.0	-0,63	-0,06
Mpp5	Q9JL04	TSSGSDTDIYSFHSATEQEDLLSDIQQAIR	T(1): 0.0; S(2): 0.3; S(3): 49.7; S(5): 49.7; T(7): 0.3; Y(10): 0.0; S(11): 0.0; S(14): 0.0; T(16): 0.0; S(23): 0.0	-0,63	0,30
Add2	Q9QY81	ELASPLALTPR	S(4): 0.8; T(9): 99.2	-0,63	0,29
Brsk1	Q5RJI5	SPVFSFSPEPGAGDEAR	S(1): 99.9; S(5): 0.1; S(7): 0.0	-0,64	-0,08
Nsd1	O88491	LMTAQNLASYR	T(3): 87.8; S(9): 0.4; Y(10): 11.8	-0,65	0,06
Braf	F6SZ47	SSSAPNVHINTIEPVNIDDLIR	S(1): 47.2; S(2): 47.2; S(3): 5.6; T(11): 0.0	-0,65	-0,10
Nim1	Q8BHE7	DGATLASSDVASYRAK	T(4): 0.0; S(7): 0.0; S(8): 0.0; S(12): 50.0; Y(13): 50.0	-0,65	-0,27
Gorasp2	Q99JP7	LPSSSSEMGSQDGSPLR	S(3): 0.0; S(4): 0.0; S(5): 0.0; S(6): 0.0; S(10): 0.0; S(14): 100.0	-0,65	-0,13
Mark2	E9QMP6	RSSDQAVPAIPTSNSYSK	S(2): 0.5; S(3): 99.5; T(12): 0.0; S(13): 0.0; S(15): 0.0; Y(16): 0.0; S(17): 0.0	-0,66	-0,03
Ppp1r1b	Q60823	YFDDEFTAQSITITPPDR	Y(1): 0.0; T(7): 0.0; S(10): 0.0; T(12): 0.2; T(14): 99.8	-0,66	0,54
Kank3	Q9Z1J2-2	AAEQEAQLLSQLK	S(10): 100.0	-0,67	0,06
Tekt4	Q0VGU4	DPEEDDRSEELEALASLLQELR	S(8): 100.0; S(16): 0.0	-0,68	0,03
Mfsd6	Q8CA95-3	GEETAMWISGPGPAPSKSTPEK	T(4): 0.0; S(9): 0.7; S(16): 0.7; S(18): 0.7; T(19): 97.8	-0,69	-0,25
Stard8	Q8K019-2	ELFDYSPPLHK	Y(5): 5.0; S(6): 95.0	-0,69	0,57
Heatr5b	Q8C4T8	LTESMTNVLEGDSMDQDVESPVAIHQPK	T(2): 0.0; S(4): 0.0; T(6): 0.0; S(13): 0.6; S(20): 99.4	-0,70	0,25
Rilpl1	Q9JIX8-4	SQSPSPPPLPEDLEK	S(1): 0.3; S(3): 99.3; S(5): 0.3	-0,70	0,09
Abcb5	B5X0E4	EVADAANIHSFIEGLPR	S(10): 100.0	-0,70	0,69
Comt	O88587-2	AVYQGPGSSPVK	Y(3): 0.0; S(8): 50.0; S(9): 50.0	-0,70	0,39
Grid1	Q61624	AGIATQFSTANGQVNLR	T(5): 49.8; S(8): 49.8; T(9): 0.5	-0,70	-0,18
Sgip1	Q8VCY8-2	LSPWEDLSQAPTMDSPLEK	S(2): 0.0; S(8): 0.0; T(12): 0.4; S(15): 99.6	-0,70	0,03
Ankrd6	A2ANY8	VLLEAGADTTIVNNILR	T(9): 50.0; T(10): 50.0	-0,70	-0,22
Crkl	P47941	YPSPPVGSVSAPNLPTAEENLEYVR	Y(1): 12.0; S(3): 75.6; S(8): 12.0; S(10): 0.4; T(16): 0.0; Y(23): 0.0	-0,73	0,73
4921517D22Rik	Q8CE22-4	IIQPSLQAPWQR	S(5): 100.0	-0,74	0,63
Zc3h13	E9Q784	SLSPSHLTEDR	S(1): 0.4; S(3): 99.6; S(5): 0.0; T(8): 0.0	-0,75	0,48

Disease Models & Mechanisms • Supplementary information

Fam195b	Q3UED7	LYFLDMVTEDAKTLLK	Y(2): 100.0; T(8): 0.0; T(13): 0.0	-0,76	0,57
Arhgef7	Q9ES00	LAGGQTSQPTTPLTSPQR	T(6): 0.0; S(7): 0.0; T(10): 0.0; T(11): 0.0; T(14): 9.5; S(15): 90.5	-0,78	0,00
Scn2a1	B1AWN6	SSSYHVSMDLLEDPTSR	S(1): 10.2; S(2): 10.2; S(3): 1.5; Y(4): 1.5; S(7): 76.5; T(15): 0.0; S(16): 0.0	-0,80	-0,23
Map1b	P14873	VLSPLRSPPLLGSESPYEDFLSADSK	S(3): 0.8; S(7): 96.9; S(13): 0.8; S(15): 0.8; Y(17): 0.8; S(22): 0.0; S(25): 0.0	-0,81	0,34
Klc2	D3YXZ3	DSLDDLFPNEDEQSPAPSPGGGDVAAQHGGYEIPAR	S(2): 0.0; S(14): 100.0; S(18): 0.0; Y(31): 0.0	-0,85	0,42
Dchs1	F6TP58	ITFNLLAGGDGLFTVDPTTGHVR	T(2): 0.1; T(14): 43.8; T(18): 12.3; T(19): 43.8	-1,01	-0,28
Ubxn1	Q922S4	EFFSQGDLEK	S(4): 100.0	-1,01	0,27
CA198	Q8C3Q5-2	SSSLTPGLGGPDSMAPR	S(1): 0.1; S(2): 8.8; S(3): 82.4; T(5): 8.8; S(13): 0.0	-1,03	-0,22
Ccny	Q8BGT8	LDHALSSPSSPCEEIK	S(6): 0.0; S(7): 0.0; S(9): 0.2; S(10): 99.8	-1,05	-0,49
Tmem145	E9PUE6	VVTMAEPGAASPPPPAR	T(3): 0.0; S(11): 100.0	-1,13	0,08
Bckdha	Q3U3E2	RNGSPTPAGALAGGAVGPPGGPGSR	S(4): 19.2; T(6): 80.8; S(24): 0.0	-1,13	1,24
Khsrp	Q3U0D9-4	ELLLSGMPEIDVNDWIK	S(5): 100.0	-1,17	0,83
Dnaja1	P63037	NVVHQLSVTLEDLYNGATR	S(7): 100.0; T(9): 0.0; Y(14): 0.0; T(18): 0.0	-1,22	0,20
Sptbn1	Q62108-3	GNSGLGFSIAGGTDNPHIGDDPSIFITK	S(3): 33.3; S(8): 33.3; T(13): 33.3; S(23): 0.0; T(27): 0.0	-1,25	1,04
Xrn2	Q9DB05	AIDIYEQVGTSAMDSPLLK	Y(5): 0.0; T(10): 0.0; S(11): 0.0; S(15): 100.0	-1,33	0,96
Crmp1	Q6P1B3	QEESQQPLTDLSPAGVTVLGAFGDSPTPTPDHEEPR	S(4): 0.0; T(9): 0.0; S(12): 0.0; T(17): 0.0; S(25): 46.7; T(27): 46.7; T(29): 6.5	-1,39	1,34
Kif1b	Q60520	VEDALSYLDQVK	S(6): 0.1; Y(7): 99.9	-2,20	0,33
Ehd3	Q9QXW9	NHPGSDTSPEAEASSGGGGVALK	S(5): 6.3; T(7): 6.3; S(8): 87.5; S(14): 0.0; S(15): 0.0	-2,43	0,58

Disease Models & Mechanisms • Supplementary information

Supplementary Table S3. Proteins up and down phosphorylated in the cerebellum of Tg(*Dyrk1a*) mice and reverted by L41 treatment.

Gene name	Protein Group Accessions	Sequence	phosphoRS Site Probabilities	TgDyrk1a vs WT	TgDyrk1a +/-L41
Dst	E9QL23	SESNSSITATQPTLAK	S(1): 49.4; S(3): 49.4; S(5): 1.1; S(6): 0.0; T(8): 0.0; T(10): 0.0; T(13): 0.0	3,45	-2,73
Stxbp5	D3Z2Q2	RPVSVSPSSSQEISENQYAVICSEK	S(4): 1.2; S(6): 95.4; S(8): 1.2; S(9): 1.2; S(10): 1.2; S(14): 0.0; Y(18): 0.0; S(23): 0.0	2,94	-2,21
Acot11	Q8VHQ9	SISHPESGDPPTMAEGEGYR	S(1): 100.0; S(3): 0.0; S(7): 0.0; T(12): 0.0; Y(19): 0.0	2,75	-2,76
Mprip	P97434-2	DFASEAPTAPLSDACPLSPHR	S(4): 0.0; T(8): 0.0; S(12): 0.0; S(18): 100.0	2,12	-0,71
Sgip1	Q8VD37	HVPSPLNLEEVQK	S(4): 100.0	2,10	-1,78
Gbf1	Q6A099	ADAPDAGAQSDSELPSYHQNDVSLDR	S(10): 100.0; S(12): 0.0; S(16): 0.0; Y(17): 0.0; S(23): 0.0	1,57	-0,32
Dclk2	Q6PGN3-3	SSSSSPTSPGSFR	S(1): 0.0; S(2): 0.0; S(3): 0.0; S(4): 0.0; S(5): 99.0; T(7): 0.5; S(8): 0.5; S(11): 0.0	1,52	-1,25
Pcyt1a	P49586	AVTCDISEDEED	T(3): 0.0; S(7): 100.0	1,50	0,07
Rgl3	Q3UYI5	NREPPPPGSPPASPGPQSPSTK	S(9): 100.0; S(13): 0.0; S(18): 0.0; S(20): 0.0; T(21): 0.0	1,44	-1,37
Ssrp1	A2AW05	EGINPGYDDYADSDEDQHDAYLER	Y(7): 0.0; Y(10): 0.0; S(13): 100.0; Y(21): 0.0	1,37	0,00
Srgap2	Q91Z67	TSPVVAPTSEPSSPLHTQLLK	T(1): 0.0; S(2): 0.0; T(8): 0.0; S(9): 0.0; S(12): 49.6; S(13): 49.6; T(17): 0.7	1,33	-0,93
Epb4.1	A2AD32	TLNINGQVPTGDGVSTVSTGP	T(1): 100.0; T(10): 0.0; S(15): 0.0; T(16): 0.0; S(18): 0.0; T(19): 0.0	1,32	-0,89
Ehd2	Q8BH64	GPDEAIEDGEEGSEDDAEWVVTK	S(13): 100.0; T(22): 0.0	1,20	-0,55
Ctage5	H3BK44	AFLSPPTLLEGPLR	S(4): 99.3; T(7): 0.7	1,18	-0,37
Caskin1	Q6P9K8-2	HISSSQELLGDGPPGPGSPMSR	S(3): 6.2; S(4): 6.2; S(5): 87.6; S(18): 0.0; S(21): 0.0	1,09	-0,91
Mier1	Q5UAK0-4	APSPPPTASNSSNSQSEK	S(3): 0.8; T(7): 90.7; S(9): 8.5; S(11): 0.0; S(12): 0.0; S(14): 0.0; S(16): 0.0	1,09	-1,33
Ifi44	Q8BV66	DMLILSALK	S(6): 100.0	1,08	-0,49
Usp9x	Q4FE56	AQENYEGGEEVSPPQTK	Y(5): 0.0; S(12): 100.0; T(16): 0.0	1,07	-0,51
Map7d2	A2AG50	SNSLDDSTEDVQSMDVSPVSK	S(1): 46.0; S(3): 46.0; S(7): 4.0; T(8): 4.0; S(13): 0.0; S(17): 0.0; S(20): 0.0	1,04	-0,94
Dok1	P97465	GFSSDTALYSQVQK	S(3): 49.6; S(4): 49.6; T(6): 0.7; Y(9): 0.0; S(10): 0.1	1,03	0,02
Kcnd1	Q03719	QNGGLEDSGSGDGQMLCVR	S(8): 1.7; S(10): 98.3	1,02	-0,56
Tbc1d5	Q80XQ2	EFTGSPPPSATK	T(3): 0.5; S(5): 99.5; S(9): 0.0; T(11): 0.0	1,02	-0,81
Scap	Q6GQT6	RDSCGGGAFETQENWER	S(3): 100.0; T(11): 0.0	1,02	-0,71
Nop2	Q922K7	AQDATAGVLWNEEDTDEDEDDDGVSPESHPR	T(5): 0.0; T(15): 100.0; S(25): 0.0; S(28): 0.0	0,99	-0,91
Arhgef6	Q8K4I3	KDSVPQVLLPEEEK	S(3): 100.0	0,99	-0,62
Zfml	E9QML5	QSSVTQVTEQSPK	S(2): 0.0; S(3): 0.0; T(5): 0.0; T(8): 0.0; S(11): 100.0	0,99	-0,21
Dync1i2	O88487	SVSTPSEAGSQDSGDGAVGSR	S(1): 0.0; S(3): 0.0; T(4): 0.0; S(6): 0.0; S(10): 12.1; S(13): 86.1; S(20): 1.8	0,97	-1,03
Cadm4	Q8R464	GSYLTHEASGLDEQGEAR	S(2): 0.0; Y(3): 0.0; T(5): 50.0; S(9): 50.0	0,97	-1,10
Tom1l2	Q5SRX1	AAETVPDLPSPPTEAPAPASNTSTR	T(4): 0.0; S(10): 38.8; T(13): 38.8; S(20): 1.4; T(22): 7.0; S(23): 7.0; T(24): 7.0	0,95	-0,11
Stx1a	O35526	TAKDSDDDDDVTVTVDR	T(1): 0.0; S(5): 100.0; T(12): 0.0; T(14): 0.0	0,93	-0,74
Ddx55	E9Q9T6	TVQLTDLGVSDLEEDS	T(1): 0.0; T(5): 0.0; S(10): 100.0; S(16): 0.0	0,90	-0,61
Arid1a	E9QAQ7	SHHAPMSPGSSGGGGQPLAR	S(1): 0.0; S(7): 100.0; S(10): 0.0; S(11): 0.0	0,89	-0,48
Rnpep	Q8VCT3	KPFVYTQGQAVLNR	Y(5): 100.0; T(6): 0.0	0,85	-1,41
Map1b	P14873	TTEAAATAVGTAATTAAVVAAAGIAASGPVK	T(1): 0.0; T(2): 0.2; T(7): 16.3; T(11): 83.3; T(14): 0.2; T(15): 0.0; S(27): 0.0	0,83	-0,66
Zswim8	Q3UHH1-3	KQSAGPNSPTGGGGGGGGSGGTR	S(3): 0.0; S(8): 99.8; T(10): 0.2; S(18): 0.0; T(21): 0.0	0,82	0,02

Disease Models & Mechanisms • Supplementary information

Pkp4	Q68FH0	TEPEQGTLYSPEQTSLHESEGSLGNSR	T(1): 0.0; T(7): 0.0; Y(9): 0.0; S(10): 0.4; T(14): 49.8; S(15): 49.8; S(19): 0.0; S(22): 0.0; S(26): 0.0	0,82	-1,08
Syn1	O88935	QTSQQPAGPPAQQRPPPQGGPPQPGPGPQR	T(2): 15.6; S(3): 84.4	0,80	-0,73
Map1a	Q9QYR6	MASPPPSGPPSAAHTPFHQSPVEEK	S(3): 11.2; S(7): 11.2; S(11): 77.5; T(15): 0.0; S(20): 0.0	0,79	-0,28
Htt	P42859	ASDPSPATPDSESVIVAMER	S(2): 0.0; S(5): 0.3; T(8): 97.7; S(11): 1.8; S(13): 0.3	0,78	-1,61
Slc43a2	Q8CGA3	LCLSTVDLEVK	S(4): 50.0; T(5): 50.0	0,77	-0,78
Map2	P20357	ETSPETSLIQDEVALK	T(2): 99.7; S(3): 0.2; T(6): 0.0; S(7): 0.0	0,76	-0,66
Synj1	E9Q7S0	TPGPPSSQGSPVDTQPAAQK	T(1): 2.3; S(6): 2.3; S(7): 13.3; S(10): 82.0; T(14): 0.1	0,76	-0,72
Ube4b	Q9ES00	LAGGQTSQPTTPLTSPQR	T(6): 0.0; S(7): 0.0; T(10): 0.0; T(11): 0.0; T(14): 50.0; S(15): 50.0	0,75	-0,03
Arid1a	E9QAQ7	SHHAPMSPGSSGGGGQPLAR	S(1): 0.0; S(7): 100.0; S(10): 0.0; S(11): 0.0	0,74	-0,76
Gm996	A2AJA9	SLEQLDELITDLVIDSR	S(1): 100.0; T(10): 0.0; S(16): 0.0	0,74	-0,08
Stxbp1	O08599-2	ESSRVSFEDQAPTME	S(2): 0.0; S(3): 0.0; S(6): 99.9; T(13): 0.0	0,73	-0,53
Ccdc88a	Q5SNZ0-3	SSSQENLLDEVMK	S(1): 33.3; S(2): 33.3; S(3): 33.3	0,73	-0,52
Synj1	E9Q7S0	TPGPPSSQGSPVDTQPAAQK	T(1): 0.0; S(6): 0.1; S(7): 0.0; S(10): 99.9; T(14): 0.0	0,71	-0,56
Sorbs1	Q62417	SAQDLSDVSTDEVGIPLRNTERSK	S(1): 0.0; S(6): 0.0; S(9): 0.0; T(10): 0.0; T(20): 1.8; S(23): 98.2	0,70	-0,69
Ccdc88a	Q5SNZ0-3	DTTSFEDISPQGISDDSSTGSR	T(2): 0.2; T(3): 6.6; S(4): 46.1; S(9): 46.1; S(14): 1.0; S(17): 0.0; S(18): 0.0; T(19): 0.0; S(21): 0.0	0,70	-1,52
Lgalsl	Q8VED9	LDDGHLNNSLGSPVQADVYFPR	S(9): 0.0; S(12): 100.0; Y(19): 0.0	0,70	-0,52
Eif4b	Q8BGD9	ARPTTDSFDDYPPR	T(4): 0.0; T(5): 0.0; S(7): 100.0; Y(11): 0.0	0,70	-0,71
Strip1	Q8C079-3	AASPPASASDLIEQQQK	S(3): 95.0; S(7): 5.0; S(9): 0.0	0,69	-0,64
Arhgap5	E9PYT0	GGIDNPAITSDQEVDDK	T(9): 7.6; S(10): 92.4	0,69	-0,63
Eif4b	Q8BGD9	SPPYTAFLGNLPYDVTEDSIK	S(1): 33.3; Y(4): 33.3; T(5): 33.3; Y(13): 0.0; T(16): 0.0; S(19): 0.0	0,68	-0,50
Nos1ap	Q9D3A8	IFSYIARDGASNIFR	S(3): 50.0; Y(4): 50.0; S(11): 0.0	0,67	-0,30
Camsap2	Q8C1B1	SESVEGFLSPSR	S(1): 0.0; S(3): 0.0; S(9): 99.7; S(11): 0.3	0,67	-0,39
Gm21985	Q6P6P5	IDDIPGLSDTSPDLSSR	S(8): 6.2; T(10): 87.6; S(11): 6.2; S(15): 0.0; S(16): 0.0	0,66	-0,64
Sfr1	Q8BP27-2	ENPPSPHSNSSGK	S(5): 100.0; S(8): 0.0; S(10): 0.0; S(11): 0.0	0,66	-0,34
Pclo	Q9QYX7	KDSFSQESSPSSPSDLAK	S(3): 0.0; S(5): 0.0; S(8): 0.0; S(9): 0.0; S(11): 48.1; S(12): 48.1; S(14): 3.8	0,65	-0,42
Cep170	H7BX26	QGSFTIDKPSSNIPIELIPHINK	S(3): 50.0; T(5): 50.0; S(10): 0.0; S(11): 0.0	0,65	-0,82
Cpeb3	D3Z1R6	SSLFPFEDAFLDDSHGDQALSSGLSSPTR	S(1): 0.0; S(2): 0.0; S(14): 0.0; S(21): 0.0; S(22): 0.0; S(25): 10.0; S(26): 80.0; T(28): 10.0	0,65	-0,42
Ptprz1	B9EKR1	EVSADVSEEENFLTDFK	S(3): 0.0; S(7): 100.0; T(14): 0.0	0,65	-0,66
Cyp17a1	P27786	MNAENNNTGEGQDPSVFSDK	T(8): 0.0; S(15): 10.1; S(18): 89.9	0,65	-1,15
Sltm	Q8CH25-2	DVQDAIAQSPEK	S(9): 100.0	0,64	-0,45
Cstf2t	Q8C7E9	QGGGQPSSFSPGQSQVTPQDQEK	S(7): 0.0; S(8): 0.0; S(10): 99.9; S(14): 0.0; T(17): 0.0	0,64	-0,46
Epn2	Q8CHU3	AGGSPASYHGSTSPR	S(4): 100.0; S(7): 0.0; Y(8): 0.0; S(11): 0.0; T(12): 0.0; S(13): 0.0	0,64	0,21
Plcl1	Q3USB7	KLPSESDLLEGEVTDEDEEAEMSR	S(4): 0.0; S(6): 0.0; T(14): 100.0; S(23): 0.0	0,64	0,04
Dcps	Q9DAR7	EAGVGNGTSAPVR	T(8): 50.0; S(9): 50.0	0,64	-0,27
Kif2a	P28740-1	ARPSQLPEQSSSAQQNGSVSDISPVQAAK	S(4): 0.0; S(10): 0.0; S(11): 0.0; S(12): 0.0; S(18): 0.0; S(20): 0.0; S(23): 100.0	0,63	0,01
Chmp7	Q8R1T1	VSPVNDVDVGVYQLMQSEQLLSR	S(2): 100.0; Y(12): 0.0; S(17): 0.0; S(22): 0.0	0,62	-0,72
Tnr	Q8BYI9	ITFTPSSGISSEVTVPR	T(2): 0.0; T(4): 1.0; S(6): 48.8; S(7): 48.8; S(10): 0.2; S(11): 1.0; T(14): 0.2	0,62	-0,92
Dhx9	O70133	AEENNSGVESSGYGSPGPTWDR	S(6): 0.0; S(10): 0.0; S(11): 0.0; Y(13): 0.0; S(15): 99.9; T(19): 0.0	0,62	-0,72
Zdhhc5	Q8VDZ4	SIGSASPGPGQPPLSSPTR	S(1): 0.0; S(4): 0.0; S(6): 0.0; S(15): 7.6; S(16): 84.9; T(18): 7.6	0,62	-0,52
Gclm	O09172	TLNEWSSQISPDLVR	T(1): 0.0; S(6): 0.0; S(7): 0.0; S(10): 100.0	0,61	-0,27
Pacsin1	Q61644	ALYDYDGQEQDELSFK	Y(3): 0.0; Y(5): 0.0; S(14): 100.0	0,61	-0,36
Dock4	P59764	NSAPASMSPDGTR	S(2): 0.0; S(6): 8.8; S(8): 91.2; T(12): 0.0	0,60	-0,59

Disease Models & Mechanisms • Supplementary information

Ralgapa1	Q6GYP7	SATTTGSPGTEK	S(1): 0.0; T(3): 0.0; T(4): 0.0; T(5): 0.6; S(7): 99.3; T(10): 0.1	0,59	-0,63
Top2b	Q64511	KASGSENEGDYNPGR	S(3): 50.0; S(5): 50.0; Y(11): 0.0	0,59	0,19
Fam117b	Q3U3E2	TSPTVATQTGASVTSTR	T(1): 50.0; S(2): 50.0; T(4): 0.0; T(7): 0.0; T(9): 0.0; S(12): 0.0; T(14): 0.0; S(15): 0.0; T(16): 0.0	0,59	0,00
BC051076	Q80XE3	NLLSLAEEIFNASMR	S(4): 99.9; S(13): 0.1	0,58	-0,92
Scn11a	Q9R053	QRALSAVSILTITMQEQEK	S(5): 50.0; S(8): 50.0; T(11): 0.0; T(13): 0.0	0,58	-0,39
Barx1	Q9ER42	NSIPTSEQLTEQER	S(2): 43.4; T(5): 6.6; S(6): 43.4; T(10): 6.6	0,58	-0,37
Zc3hc1	H3BKM2	SQDATVSPGSEQSEKSPGPIVSR	S(1): 0.0; T(5): 0.0; S(7): 0.0; S(10): 0.0; S(13): 0.5; S(16): 99.5; S(22): 0.0	0,58	-0,14
Myo9b	Q9QY06	AQDKPESPSGSTQIQR	S(7): 100.0; S(9): 0.0; S(11): 0.0; T(12): 0.0	0,58	-0,43
Park2	Q9WVS6	SHETNASGGDEPQSTSEGSIWESR	S(1): 10.2; T(4): 89.6; S(7): 0.2; S(14): 0.0; T(15): 0.0; S(16): 0.0; S(19): 0.0; S(23): 0.0	0,58	-0,30
Efhd2	Q8C845	ADLNQGIGEPQSPSRR	S(12): 98.8; S(14): 1.2	0,58	-0,51
Gpsm1	Q6IR34-3	AAQSSVTASPQTEEFFDLIASSQSR	S(4): 0.0; S(5): 0.0; T(7): 8.5; S(9): 83.0; T(12): 8.5; S(21): 0.0; S(22): 0.0; S(24): 0.0	0,57	-0,17
Lcp1	Q61233	GSVSDEEMMELR	S(2): 100.0; S(4): 0.0	0,57	0,10
Sgip1	Q8VD37	YNSPELDEEGYSIRPEEPGSTK	Y(1): 0.0; S(3): 0.0; Y(11): 0.0; S(12): 0.0; S(20): 89.2; T(21): 10.8	0,57	-0,43
Fam117b	Q3U3E2	TSPTVATQTGASVTSTR	T(1): 50.0; S(2): 50.0; T(4): 0.0; T(7): 0.0; T(9): 0.0; S(12): 0.0; T(14): 0.0; S(15): 0.0; T(16): 0.0	0,56	-0,09
Sacs	Q9JLC8-3	EVMNTFWPGR	T(5): 100.0	0,56	-0,06
Il16	O54824	DPGLPESPPPGQRPSTK	S(7): 100.0; S(15): 0.0; T(16): 0.0	0,55	-0,15
Ctnna2	Q61301-2	HISPVQALSEFK	S(3): 100.0; S(9): 0.0	0,53	-1,15
Vmn2r100	E9QAZ9	LAIDAEYEILNLWNLPK	Y(7): 100.0	0,53	-0,71
Coro1a	O89053	RATPEPSGTPSSDTVSR	T(3): 99.8; S(7): 0.1; T(9): 0.0; S(11): 0.0; S(12): 0.0; T(14): 0.0; S(16): 0.0	0,53	-0,03
Atp13a1	Q9EPE9	RPRDSPVLSNSGPR	S(5): 100.0; S(9): 0.0; S(11): 0.0	0,52	0,01
Ctnnb1	Q02248	RTSMGGTQQQFVEGVR	T(2): 50.0; S(3): 50.0; T(7): 0.0	0,52	-0,39
Zfr2	E9Q5M4	SPDSDSVSPGPQRPPPSSQR	S(1): 100.0; S(4): 0.0; S(6): 0.0; S(8): 0.0; S(17): 0.0; S(18): 0.0	0,52	-0,32
Akap12	Q9WTQ5	QSQVTEEEAAAAQTEGPSTPSSFPAQEEHR	S(2): 0.0; T(5): 0.0; T(14): 0.0; S(18): 92.3; T(19): 7.7; S(21): 0.0; S(22): 0.0	0,52	-1,14
Lztr1	E9PUQ1	SFMPEDGQVNISIGEMVPSR	S(1): 99.9; S(12): 0.1; S(19): 0.0	0,52	-0,28
Oxct1	Q9D0K2	SARNFNLPMCK	S(1): 100.0	0,51	-0,45
Gpr124	J3QPB6	FLGYVDQIK	Y(4): 100.0	0,51	-0,46
Clns1a	Q923F1	LGEESKEPLSDEDEEDNDDVEPISEFR	S(5): 0.0; S(10): 100.0; S(24): 0.0	0,50	0,29
Spast	Q9QYY8	GIAVIVTGQGEQYER	T(7): 1.4; Y(13): 98.6	0,50	-0,52
Sptbn1	Q62261	TLETPAAQMEGFLNR	T(1): 0.0; T(4): 100.0	0,50	-0,13
Map1a	Q9QYR6	ELSSEPRTPPAQK	S(3): 0.0; S(4): 0.0; T(8): 100.0	0,50	0,03
Map1a	Q9QYR6	ELSSAVSPPNLHSDTPTFSYASLAGPTIPPR	S(3): 0.0; S(4): 0.0; S(7): 98.9; S(13): 0.5; T(15): 0.5; T(17): 0.0; S(19): 0.0; Y(20): 0.0; S(22): 0.0; T(27): 0.0	0,50	-0,47
Nufip2	Q5F2E7	GADNDGSGSESGYTTPK	S(7): 92.0; S(9): 7.9; S(11): 0.1; Y(13): 0.1; T(14): 0.0; T(15): 0.0	0,50	-0,32
Map7d2	A2AG50	SNSLDDSTEDVQSMDVSPVSK	S(1): 0.0; S(3): 0.0; S(7): 0.0; T(8): 0.0; S(13): 0.0; S(17): 100.0; S(20): 0.0	0,50	-0,40
Prkcb	P68404-2	NIDQSEFEGFSFVNSEFLKPEVK	S(5): 0.0; S(11): 50.0; S(15): 50.0	0,49	-0,53
Syn1	O88935	QSRPVAGGPGAPPAARPPASPSPQR	S(2): 0.0; S(20): 1.2; S(22): 98.8	0,49	-0,31
Serinc1	Q9QZI8	LTLTSDESTLIEDGNGR	T(2): 0.0; T(4): 92.4; S(5): 7.0; S(8): 0.6; T(9): 0.0	0,49	-0,42
Myo5a	D3YZ62	IGELEVGQMENISPGQIIDEPIRPVNIPR	S(13): 100.0	0,49	-1,08
Syn1	O88935	SQSLTNAFNLPEPAPPRPSLSQDEVK	S(1): 0.0; S(3): 0.0; T(5): 0.0; S(19): 50.0; S(21): 50.0	0,48	-0,27
Ndrg4	Q8BTG7-3	RLSGGAVPSASMTR	S(3): 0.9; S(9): 90.2; S(11): 8.8; T(13): 0.1	0,48	-0,21
Abcf1	Q6P542	GGNVFEALIQDDSEEEEEEENR	S(13): 100.0	0,48	-1,07
Brsk1	Q5RJI5	SPVFSFSPEPGAGDEAR	S(1): 100.0; S(5): 0.0; S(7): 0.0	0,47	-0,07

Disease Models & Mechanisms • Supplementary information

Rif1	Q6PR54-2	RASQGLISAVENSESDSSEAK	S(3): 97.3; S(8): 2.7; S(13): 0.0; S(15): 0.0; S(17): 0.0; S(18): 0.0	0,47	-0,32
Fam103a1	Q9CQY2	RPPESPPIVEEWNSR	S(5): 100.0; S(14): 0.0	0,47	-0,37
Uncharacterized protein C17orf59 homolog	Q9D6W8	RGSPGGVEMNVELPQQEGDDDDDEDEEAAAGR	S(3): 100.0	0,47	0,18
Srgap1	Q91Z69	RPGHGSLTNISR	S(6): 100.0; T(8): 0.0; S(11): 0.0	0,47	-0,56
Hhip	Q7TN16	GPASNYLGQMEDYEK	S(4): 88.0; Y(6): 12.0; Y(13): 0.0	0,47	-0,36
Kif2a	P28740-1	EFGISPSDIPFSQGGGSRPDLSPSYDYDDFSPSITR	S(5): 0.3; S(7): 0.3; S(12): 32.9; S(17): 32.9; S(22): 32.9; S(24): 0.3; Y(25): 0.3; Y(27): 0.0; S(31): 0.0; S(33): 0.0; T(35): 0.0	0,47	-0,09
Tanc1	E9QAF9	RADNCSPVAEEETTGSAESVLPK	S(6): 100.0; T(13): 0.0; T(14): 0.0; S(16): 0.0; S(19): 0.0	0,46	-0,28
Dpysl3	E9PWE8	GMYDGPVFDLTTTPK	Y(3): 0.0; T(11): 0.4; T(12): 6.0; T(13): 93.6	0,46	-0,09
Ptchd3	Q0EEE2	GFIQIVDVSSSSNK	S(9): 25.0; S(10): 25.0; S(11): 25.0; S(12): 25.0	0,45	-0,75
Znf687	Q9D2D7	SDPEGGDSPLPAPGDPLTCK	S(1): 0.0; S(8): 100.0; T(18): 0.0	0,45	-0,27
Bcan	Q61361	EVGGETGSPELSGVPR	T(6): 8.4; S(8): 91.6; S(12): 0.0	0,45	0,06
Tmem100	Q9CQG9	RRESQTALVVNQR	S(4): 100.0; T(6): 0.0	0,45	-0,36
Epn1	Q80VP1	SPGAFDMSGVGGSLAESVGSPPPAATPTPTPPTR	S(1): 0.0; S(8): 0.2; S(13): 4.0; S(17): 4.0; S(20): 91.5; T(26): 0.2; T(28): 0.0; T(30): 0.0; T(33): 0.0	0,45	-0,03
Alad	P10518	DAAQSSPAFGDR	S(5): 50.0; S(6): 50.0	0,44	-0,07
Lrrfip2	E9QN52	RGSGDTSSLIDPDTSLSELR	S(3): 99.5; T(6): 0.4; S(7): 0.0; S(8): 0.0; T(14): 0.0; S(15): 0.0; S(17): 0.0	0,43	0,09
Dyrk1a	A1L341	IYQYIQSR	Y(2): 0.0; Y(4): 100.0; S(7): 0.0	0,43	-0,07
Mtss1l	F7D291	GGGAPWPGGAQTYSPSSTCR	T(12): 1.0; Y(13): 1.0; S(14): 88.8; S(16): 9.0; S(17): 0.1; T(18): 0.1	0,43	-0,52
Dsg2	O55111	LDLSIIVTNK	S(4): 100.0; T(8): 0.0	0,43	0,07
Plxnb3	Q9QY40	QEQGQIILSVPR	S(9): 100.0	0,43	-0,17
Irf2bp2	E9Q1P8	NSSSPPSPSSMNQR	S(2): 0.0; S(3): 50.0; S(4): 50.0; S(7): 0.0; S(9): 0.0; S(10): 0.0	0,43	0,10
Akap12	Q9WTQ5	QAQSSTEIPLQAESGQGTEEEAAKDGEENR	S(4): 33.3; S(5): 33.3; T(6): 33.3; S(14): 0.0; T(18): 0.0	0,43	-0,04
Dclk1	Q9JLM8	SKSPASTSSVNGTPGSQLSTPR	S(1): 49.2; S(3): 49.2; S(6): 0.5; T(7): 0.5; S(8): 0.5; S(9): 0.0; T(13): 0.0; S(16): 0.0; S(19): 0.0; T(20): 0.0	0,43	0,25
2010300C02Rik	E9Q3M9	DMSPPEGDVAPPK	S(3): 100.0	0,43	-0,15
Reps1	D3Z2E3	RQSSSYEDPWK	S(3): 99.4; S(4): 0.2; S(5): 0.2; Y(6): 0.2	0,41	-0,11
Tppp	Q7TQD2	RLSLESEGANEGATAAPELSALEEAFR	S(3): 93.2; S(6): 6.8; T(14): 0.0; S(20): 0.0	0,41	0,11
Atp1a3	Q6PIC6	CIELSSGSVK	S(5): 0.2; S(6): 49.9; S(8): 49.9	0,41	-0,26
Map2	P20357	LASVSADAEVAR	S(3): 94.4; S(5): 5.6	0,41	-0,22
Coil	Q5SU73	LMEDEETDQGYK	T(7): 100.0; Y(11): 0.0	0,41	-0,24
Map1a	Q9QYR6	VAELEEEQSQGSSSYSDWVK	S(9): 100.0; S(12): 0.0; S(13): 0.0; S(14): 0.0; Y(15): 0.0; S(16): 0.0	0,41	-0,66
Iyd	Q9DCX8	AAGTAPSGAHTEPWTFVVVK	T(4): 0.0; S(7): 0.7; T(11): 3.3; T(15): 95.9	0,41	-0,32
Camkk2	Q8C078	KMSLQEPSQGGPASSSNSLDMNGR	S(3): 100.0; S(8): 0.0; S(14): 0.0; S(15): 0.0; S(16): 0.0; S(18): 0.0	0,41	-0,55
Plcl1	Q3USB7	KLPSESDLLEGEVTDEDEEAEMSR	S(4): 0.0; S(6): 0.0; T(14): 100.0; S(23): 0.0	0,41	-0,23
Bad	F7ABX5	RMSDEFEGSFK	S(3): 100.0; S(9): 0.0	0,41	0,16
Rem2	Q8VEL9-2	GHAGGQRPEPSSPDGPAPPTR	S(11): 50.0; S(12): 50.0; T(20): 0.0	0,40	0,08
Stim2	P83093	GSPECVGLTETK	S(2): 100.0; T(9): 0.0; T(11): 0.0	0,40	-0,14
Map1a	Q9QYR6	QLSPESLGTLQFGELSLGK	S(3): 100.0; S(6): 0.0; T(9): 0.0; S(16): 0.0	0,40	-0,20
Usp8	Q80U87	SYSSPDITQALQEEEK	S(1): 0.3; Y(2): 0.3; S(3): 4.8; S(4): 89.8; T(8): 4.8	0,40	-0,10
Ryr2	E9Q401	RISQTSQVSIDAAHGYSPR	S(3): 100.0; T(5): 0.0; S(6): 0.0; S(9): 0.0; Y(16): 0.0; S(17): 0.0	0,40	-0,29
Chmp6	B1AZ42	MHQVMSIEEVER	S(6): 100.0	0,40	-0,05
Arhgef12	Q8R4H2	SEGVQDAEPQSLVGSPSTR	S(1): 0.0; S(11): 0.0; S(15): 83.9; S(17): 8.0; T(18): 8.0	0,40	-0,16
Map4k4	E9PVG7	RDSPLQGGGGQQNSQAGQR	S(3): 100.0; S(13): 0.0	0,40	-0,26
Map3k7	Q62073	SIQDLTVTGTEPGQVSSR	S(1): 100.0; T(6): 0.0; T(8): 0.0; T(10): 0.0; S(16): 0.0; S(17): 0.0	0,40	-0,21

Arhgef18	Q6P9R4-2	RAETFGGYDSVGSPSK	T(4): 0.0; Y(8): 0.0; S(10): 0.0; S(13): 100.0; S(15): 0.0	0,39	-0,28
Tspyl4	Q8VD63	EMEAAGEAGADGGASPDSEHCGPELCFR	S(15): 99.5; S(18): 0.5	0,39	-0,42
Akap12	Q9WTQ5	SATLSSTESTASGMQDEVR	S(1): 0.0; T(3): 0.0; S(5): 0.0; S(6): 0.0; T(7): 0.0; S(9): 0.0; T(10): 0.0; S(12): 100.0	0,39	-0,09
Orai3	D3Z6X8	GGEGDTGEQAPLNPEVDSPAGSATYR	T(6): 0.0; S(18): 85.1; S(22): 2.0; T(24): 0.3; Y(25): 12.6	0,39	0,30
Map7	D3YWN7	LSSSSATLLNSPDR	S(2): 0.0; S(3): 0.0; S(4): 0.0; S(5): 0.0; T(7): 1.2; S(11): 98.8	0,39	-0,35
Park2	Q9WVS6	SHETNASGGDEPQSTSEGSIWESR	S(1): 1.6; T(4): 96.8; S(7): 1.6; S(14): 0.0; T(15): 0.0; S(16): 0.0; S(19): 0.0; S(23): 0.0	0,39	-0,30
Lnp	Q7TQ95	ADSVPNLEPSEESLVTK	S(3): 100.0; S(10): 0.0; S(13): 0.0; T(16): 0.0	0,38	-0,39
Sp4	K4DI62	ENNVSQPASSSSSSSSSNNGSSSPTK	S(5): 0.0; S(9): 0.0; S(10): 0.0; S(11): 0.0; S(12): 0.0; S(13): 0.0; S(14): 0.0; S(15): 0.0; S(16): 0.0; S(17): 0.0; S(21): 0.4; S(22): 6.1; S(23): 87.3; T(25): 6.1	0,38	-0,39
Pclo	Q9QYX7	KDSFSQESSPSSPSDLAK	S(3): 0.0; S(5): 0.0; S(8): 0.0; S(9): 0.0; S(11): 94.8; S(12): 5.2; S(14): 0.0	0,38	-0,25
Pdha1	P35486	YHGHSMSDPGVSYR	Y(1): 0.0; S(5): 100.0; S(7): 0.0; S(12): 0.0; Y(13): 0.0	0,38	0,25
Rims2	D9HP81	TGSVQTSPSSTPGTGR	T(1): 0.2; S(3): 33.0; T(6): 33.0; S(7): 33.0; S(9): 0.2; S(10): 0.2; T(11): 0.2; T(14): 0.0	0,38	-0,34
Xkr4	Q5GH67	TVVSSGSAAGEGEVRPSTPQR	T(1): 0.0; S(4): 0.0; S(5): 0.0; S(7): 0.0; S(17): 13.3; T(18): 86.6	0,37	-0,33
Lgalsl	Q8VED9	LDDGHLNNSLGSPVQADVYFPR	S(9): 0.0; S(12): 100.0; Y(19): 0.0	0,37	-0,32
Chgb	P16014	ADEFPDFYDSEEQMGPHQEANDEK	Y(8): 0.7; S(10): 99.3	0,37	-0,19
Frmd6	Q8C0V9-2	HSLSLDDIR	S(2): 0.0; S(4): 100.0	0,37	-0,47
Gulo	P58710	GDDILLSPCFQR	S(7): 100.0	0,37	-0,28
Mcf2l	E9PXE2	TSSTGEEEESLAILR	T(1): 0.4; S(2): 99.3; S(3): 0.4; T(4): 0.0; S(10): 0.0	0,36	-0,18
Acta1	P68134	DLYANNVMSGGTTMYPGIADR	Y(3): 0.0; S(9): 100.0; T(12): 0.0; T(13): 0.0; Y(15): 0.0	0,36	0,14
6430548M08Rik	Q3TA40	TVTMISPEDEQK	T(1): 0.0; T(3): 0.0; S(6): 100.0	0,36	-0,38
Fez1	Q8K0X8	SMEDLVNEFDEK	S(1): 100.0	0,36	-0,07
Lppr3	Q7TPB0	QPGMGPGSPVSDVDQEEPR	S(8): 100.0; S(11): 0.0	0,36	-0,89
Sptbn4	E9PX29	QESADHEGPHSLTLGR	S(3): 100.0; S(11): 0.0; T(13): 0.0	0,36	-0,18
Gsk3b	Q9WV60	IQAAASPPANATAASDTNAGDR	S(6): 100.0; T(12): 0.0; S(15): 0.0; T(17): 0.0	0,36	0,09
Map3k7	Q62073	SIQDLTVTGTEPGQVSSR	S(1): 100.0; T(6): 0.0; T(8): 0.0; T(10): 0.0; S(16): 0.0; S(17): 0.0	0,36	-0,15
Pi4ka	E9Q3L2	TSSVSSISQVSPER	T(1): 0.0; S(2): 0.0; S(3): 0.0; S(5): 0.0; S(6): 0.0; S(8): 0.0; S(11): 100.0	0,36	-0,47
Rufy3	Q9D394	ELDDISLTPDPEPTHEDPNYLMANER	S(6): 0.8; T(8): 99.2; T(14): 0.0; Y(20): 0.0	0,36	-0,21
Oxr1	Q4KMM3	ESLPIELDQLSGR	S(2): 0.0; S(11): 100.0	0,36	-0,23
Rph3a	P47708	WHQLQNENHVSSD	S(11): 50.0; S(12): 50.0	0,36	-0,25
Akt2	Q60823	YFDDEFTAQSITITPPDR	Y(1): 0.0; T(7): 0.0; S(10): 0.0; T(12): 0.1; T(14): 99.9	-0,37	0,14
Slc1a2	P43006-2	ESNSNQCVYAAHNSVVIDECK	S(2): 50.0; S(4): 50.0; Y(9): 0.0; S(14): 0.0	-0,37	0,22
Erbb4	Q61527-3	ILNETTGPK	T(5): 50.0; T(6): 50.0	-0,37	0,07
Cask	O70589-3	TQSSSCEDLPSTTQPK	T(1): 0.7; S(3): 5.5; S(4): 46.9; S(5): 46.9; S(11): 0.0; T(12): 0.0; T(13): 0.0	-0,37	0,17
Slc12a5	Q91V14	ESSPFINSTDTEK	S(2): 7.1; S(3): 92.9; S(8): 0.0; T(9): 0.0; T(11): 0.0	-0,37	0,18
Leo1	Q5XJE5	KLNSDEEGESSGK	S(4): 100.0; S(10): 0.0; S(11): 0.0	-0,37	0,38
Ctnna1	P26231	TPEELDDSDFETEDFDVR	T(1): 0.0; S(8): 100.0; T(12): 0.0	-0,37	0,11
Kcnt1	Q6ZPR4	SDPLAHVASSSQSR	S(1): 0.0; S(9): 49.9; S(10): 49.9; S(11): 0.1; S(13): 0.0	-0,37	0,00
Dmtn	Q9WV69	LQSTEFSPSGSEAGSPGLQNGEGQR	S(3): 46.9; T(4): 46.9; S(7): 6.1; S(9): 0.1; S(11): 0.0; S(15): 0.0	-0,37	-0,12
Ywhaz	P63101	TAFDEAIAELDTLSEESYKDSTLIMQLLR	T(1): 0.0; T(12): 49.9; S(14): 49.9; S(17): 0.1; Y(18): 0.1; S(21): 0.0; T(22): 0.0	-0,37	0,85
Adcyap1r1	E9Q3E8	HPSLASSGVNGGTQLSILSK	S(3): 100.0; S(6): 0.0; S(7): 0.0; T(13): 0.0; S(16): 0.0; S(19): 0.0	-0,37	0,09

Disease Models & Mechanisms • Supplementary information

Gprin1	Q3UNH4	AETVSPGEVDAMTLGK	T(3): 0.3; S(5): 99.7; T(13): 0.0	-0,37	0,41
Slc4a4	E9Q8N8	MFSNPDNGSPAMTHR	S(3): 0.0; S(9): 99.8; T(13): 0.2	-0,37	0,13
Amph	Q7TQF7	AFSIQGAPSDSGPLR	S(3): 0.0; S(9): 7.2; S(11): 92.8	-0,37	0,45
Dbi	Q4VWZ5	TQPTDEEMLFIYSHFK	T(1): 0.0; T(4): 0.0; Y(12): 95.8; S(13): 4.2	-0,38	0,33
Npm1	Q61937	CGSGPVHISGQHLVAVEEDAESEDEDEEDVK	S(3): 0.0; S(9): 0.0; S(22): 100.0	-0,38	0,24
Slc12a5	Q91V14	GPSPVSSEGIK	S(3): 100.0; S(6): 0.0; S(7): 0.0	-0,38	-0,19
Kdm4d	Q3U2K5	QVSRSLLPIATVSNVPCNMQVCHTSR	S(3): 0.0; S(5): 0.0; T(11): 0.0; S(13): 0.0; T(24): 13.4; S(25): 86.6	-0,38	0,11
Srrm2	Q8BTI8	SGMSPEQSK	S(1): 0.0; S(4): 100.0; S(8): 0.0	-0,38	0,29
Arvcf	P98203-2	SLAADDEGGPDLEPDYSTATR	S(1): 100.0; Y(16): 0.0; S(17): 0.0; T(18): 0.0; T(20): 0.0	-0,38	-0,34
Nrg2	P56974-2	SDSPPLCPAADSR	S(1): 0.2; S(3): 99.8; S(12): 0.0	-0,38	0,32
Ppfia3	B8QI35	ESVSLAGTPSDETLATDPLGLAK	S(2): 0.0; S(4): 0.0; T(8): 0.6; S(10): 0.6; T(13): 98.7; T(16): 0.1	-0,38	0,06
Grid2ip	Q0QWG9-2	SRASPPVPSLVGTSGPR	S(1): 0.5; S(4): 99.5; S(9): 0.0; T(13): 0.0; S(14): 0.0	-0,38	-0,05
Phkb	Q7TSH2	EGPNFITMEGTVSDHIER	T(7): 10.0; T(11): 10.0; S(13): 80.1	-0,38	-0,21
Dagla	Q6WQJ1	LLSPVAAASAAR	S(3): 100.0; S(9): 0.0	-0,38	0,28
Csnk1d	Q9DC28	GTQEVAPPTPLTPTSHTANTSPRPVSGMER	T(2): 0.0; T(9): 0.0; T(12): 0.0; T(14): 0.0; S(15): 0.0; T(17): 0.0; T(20): 1.2; S(21): 1.2; S(26): 97.7	-0,38	-0,02
Fry	E9Q8I9	KSTGQLNVNPGTPGSGGGGGGSGNTTTAER	S(2): 88.1; T(3): 11.9; T(12): 0.0; S(15): 0.0; S(22): 0.0; T(25): 0.0; T(26): 0.0; T(27): 0.0	-0,39	-0,06
Col6a5	A6H584	GITIFAVGVGNANQDELETMAGK	T(3): 100.0; T(19): 0.0	-0,39	0,03
Pak1	G5E884	DVATSPISPTENNTTPPDALTR	T(4): 0.0; S(5): 0.4; S(8): 99.6; T(10): 0.0; T(14): 0.0; T(15): 0.0; T(21): 0.0	-0,39	-0,07
Preb	D3Z3S1	NLEAVQTDFSNEPLQK	T(7): 0.4; S(10): 99.6	-0,39	0,27
Ncam1	P13595	GVTASSSSPASAPK	T(3): 0.0; S(5): 0.0; S(6): 0.3; S(7): 5.5; S(8): 94.1; S(11): 0.0	-0,39	0,07
C2cd5	Q7TPS5	SQSESSDEVTELDLSHGK	S(1): 0.6; S(3): 98.9; S(5): 0.6; S(6): 0.0; T(10): 0.0; S(15): 0.0	-0,39	0,22
Pkp4	Q68FH0	VASPSQGQVGSSSPK	S(3): 0.0; S(5): 0.0; S(11): 0.0; S(12): 5.3; S(13): 94.7	-0,40	-0,13
Mpdz	Q8VBX6-2	NVQGLGITIAGYIGDK	T(8): 98.6; Y(12): 1.4	-0,40	-0,13
Vill	D3Z669	TVVSVFPGNNK	T(1): 93.3; S(4): 6.7	-0,40	-0,13
Whsc1l1	Q6P2L6-2	SEKPAQSASSPEATSGSAGPVEK	S(1): 6.0; S(7): 6.0; S(9): 6.0; S(10): 35.1; T(14): 35.1; S(15): 6.0; S(17): 6.0	-0,40	-0,31
Zdhhc13	D3YY07	LLEAGSSLDIRNAK	S(6): 99.4; S(7): 0.6	-0,40	-0,23
Rps6kc1	Q8BLK9-2	GVDLLLEGVQGESSPTR	S(13): 49.9; S(14): 49.9; T(16): 0.2	-0,40	0,00
Cadm4	Q8R464	QKGSYLTHEASGLDEQGEAR	S(4): 0.0; Y(5): 0.0; T(7): 0.0; S(11): 100.0	-0,40	0,21
Phf3	B2RQG2	NTVDIVDKPENSPQR	T(2): 0.0; S(12): 100.0	-0,40	0,05
Rnmt	Q9D0L8	EFGEDLVEQNSSYVQDSPSK	S(11): 0.0; S(12): 0.0; Y(13): 0.0; S(17): 92.5; S(19): 7.5	-0,40	0,17
Pkp4	Q68FH0	AEQYPGSPDSWV	Y(4): 0.0; S(7): 100.0; S(10): 0.0	-0,40	0,42
Zfp185	A2BI37	RSSISGTEEEEVPFTPDEQK	S(2): 25.0; S(3): 25.0; S(5): 25.0; T(7): 25.0; T(15): 0.0	-0,40	-0,26
Gja1	P23242	SDPYHATTGPLSPSK	S(1): 0.0; Y(4): 0.0; T(7): 0.0; T(8): 0.0; S(12): 99.4; S(14): 0.6	-0,40	-0,30
Ppp3cb	E0CZ78	ICSFEEAK	S(3): 100.0	-0,40	0,33
Pianp	Q6P1B3	QEESQQPLTDLSPAGVTVLGAFGDSPTPTPDHEEPR	S(4): 0.0; T(9): 0.0; S(12): 0.0; T(17): 0.0; S(25): 99.5; T(27): 0.3; T(29): 0.3	-0,41	0,85
Slc4a4	E9Q8N8	MFSNPDNGSPAMTHR	S(3): 0.0; S(9): 95.2; T(13): 4.8	-0,41	-0,03
Transmembrane protein C15orf27 homolog	Q8BZB3	ALDPAPLAQPTPLGSVQTSPELEHR	T(11): 0.0; S(15): 0.0; T(18): 50.0; S(19): 50.0	-0,42	-0,19
Zc3h13	E9Q784	SLSPSHLTEDR	S(1): 99.7; S(3): 0.3; S(5): 0.0; T(8): 0.0	-0,42	0,24
Xirp2	Q4U4S6-2	FETQPLDMISDK	T(3): 100.0; S(10): 0.0	-0,42	0,06
Gtf3c1	Q8K284-2	VAASPRPGAEEQAEAQAPAQLAAPEDADAGGPR	S(4): 100.0	-0,42	-0,26
Edc4	G5E896	DSQDTSAEQSDHDDEVASLASASGGFGSK	S(2): 0.0; T(5): 0.0; S(6): 0.0; S(10): 100.0; S(18): 0.0; S(21): 0.0; S(23): 0.0; S(28): 0.0	-0,42	0,29

Disease Models & Mechanisms • Supplementary information

Hcn2	O88703	RAPPGPLPPAASPGPPAASPPAAPSSPR	S(12): 0.0; S(19): 0.0; S(25): 50.0; S(26): 50.0	-0,42	0,25
Gm2933	Q1LZI5	LMYVLALSSDLLVIQK	Y(3): 90.2; S(8): 9.7; S(9): 0.1	-0,42	0,22
Uty	D6RFT4	GSEVQSIIIK	S(2): 0.0; S(6): 100.0	-0,42	0,06
Trappc9	Q3U0M1-3	FNFESVPESPGEK	S(5): 0.0; S(9): 100.0	-0,43	0,12
Rtn4	Q99P72	ESAEFSVLEYSEMGSSFNGSPK	S(2): 0.0; S(6): 0.0; Y(10): 0.0; S(11): 0.0; S(15): 0.1; S(16): 0.9; S(20): 99.0	-0,43	0,01
Akt1	P31750	SGSPSDNSGAEEMEVSLAKPK	S(1): 0.0; S(3): 99.0; S(5): 0.9; S(8): 0.0; S(16): 0.0	-0,43	0,23
Col18a1	P39061-2	DGEPGDPGEDGRPGDTGPQGFPGTPGDVGPK	T(16): 1.3; T(24): 98.7	-0,43	0,05
Sfswap	Q3USH5	VKLDDDSEEDEESR	S(7): 100.0; S(13): 0.0	-0,43	-0,04
Golga4	Q91VW5	TSSFTDQLDDVTPNR	T(1): 33.3; S(2): 33.3; S(3): 33.3; T(5): 0.0; T(12): 0.0	-0,43	0,58
Vdac1	Q8K284-2	VAASPRPGAEEQAEAQAPAQLAAPEDADAGGPR	S(4): 100.0	-0,43	0,03
Mettl21e	Q8CDZ2	ELFDLEEISSFPSLNIK	S(9): 33.3; S(10): 33.3; S(13): 33.3	-0,44	-0,06
Ahnak	E9Q616	GGVTGSPEASISGSK	T(4): 0.0; S(6): 0.0; S(10): 0.1; S(12): 0.1; S(14): 99.8	-0,44	0,27
Wdr20a	Q3UWE6	SNSLPHSAVSNAASK	S(1): 0.0; S(3): 100.0; S(7): 0.0; S(10): 0.0; S(14): 0.0	-0,44	0,15
Usp47	Q8BY87-2	STETSDFENIESPLNER	S(1): 0.0; T(2): 0.0; T(4): 0.0; S(5): 0.0; S(12): 100.0	-0,44	0,25
Hepacam	Q640R3	STTEPGPPGYSVSPPVPGR	S(1): 0.0; T(2): 0.0; T(3): 0.0; Y(10): 9.5; S(11): 81.0; S(13): 9.5	-0,44	-0,36
Itih2	Q61703	SLPEESGEETDTVDPVTLYSYK	S(1): 1.1; S(6): 89.0; T(10): 9.8; T(12): 0.1; T(17): 0.0; Y(19): 0.0; S(20): 0.0; Y(21): 0.0	-0,44	0,21
Dock7	E9PX48	SLSNSNPDISGTPTSPDDEVR	S(1): 0.0; S(3): 0.1; S(5): 99.7; S(10): 0.1; T(12): 0.0; T(14): 0.0; S(15): 0.0	-0,44	0,12
Vdac1	Q60932-2	LTFDSSFSPNTGK	T(2): 0.0; S(5): 0.5; S(6): 0.5; S(8): 98.5; T(11): 0.5	-0,44	1,55
Mobp	Q9D2P8	ATSPQRPK	T(2): 50.0; S(3): 50.0	-0,44	0,54
Arpp21	E9Q4A0	AWSSTDSDSSNR	S(3): 6.6; S(4): 92.9; T(5): 0.5; S(7): 0.0; S(9): 0.0; S(10): 0.0	-0,44	-0,22
Prdm13	E9PZZ1-2	YPESISYLSGPAAAAAAAAAAAAAAAAAIGPAELGSLASIDR	Y(1): 0.1; S(4): 0.2; S(6): 0.2; Y(7): 0.3; S(9): 0.3; S(35): 49.5; S(38): 49.5	-0,45	0,34
Maoa	Q64133	VLGSQEALSPVHYEEK	S(4): 0.0; S(9): 100.0; Y(13): 0.0	-0,45	0,03
Prkce	P16054	KLAAGAESPQPASGNSPSEDDR	S(8): 0.0; S(13): 5.5; S(16): 89.0; S(18): 5.5	-0,45	0,38
Bcl2l13	P59017	TSPTPSVFVELGEEELEAVTARPEAVER	T(1): 97.6; S(2): 1.2; T(4): 1.2; S(6): 0.0; T(20): 0.0	-0,45	-0,40
Kcnk1	O08581	QSEPFVASQSPPYEDGSADH	S(2): 0.0; S(8): 50.0; S(10): 50.0; Y(13): 0.0; S(17): 0.0	-0,45	-0,41
Cdkn1b	P46414	TEENVSDGSPNAGTVEQTPK	T(1): 0.0; S(6): 0.0; S(9): 100.0; T(14): 0.0; T(18): 0.0	-0,45	0,58
Scn2a1	B1AWN6	AMSMASILTNTMEELEESR	S(3): 0.0; S(6): 0.0; T(9): 2.0; T(11): 12.5; S(18): 85.4	-0,46	0,40
Svop	Q8BFT9	TGESARSEDDAASGEHDIQIEGVR	T(1): 1.6; S(4): 49.2; S(7): 49.2; S(13): 0.1	-0,46	-0,27
Caskin2	Q8VHK1	VGLSPDSPAGDRNSVGSEGSVGSIR	S(4): 99.0; S(7): 1.0; S(14): 0.0; S(17): 0.0; S(20): 0.0; S(23): 0.0	-0,46	-0,10
Phkb	Q7TSH2	SGSVYEPLK	S(1): 5.8; S(3): 93.8; Y(5): 0.4	-0,46	-0,04
Map1b	P14873	ASLSPMDEPVPDSESPVEK	S(2): 50.0; S(4): 50.0; S(13): 0.0; S(15): 0.0	-0,46	0,66
Bclaf1	Q99JP4	QKEDVEGVGTSDGEGAAGLSSDPK	T(10): 8.1; S(11): 91.9; S(20): 0.0; S(21): 0.0	-0,46	0,76
Tprg1l	Q9DBS2	DTVDSAGTSPTAVLAAGEDAGAGRPGAGTPLR	T(2): 0.0; S(5): 0.0; T(8): 0.0; S(9): 0.0; T(11): 0.0; T(29): 100.0	-0,46	-0,05
Col3a1	P08121	GENGSPGAPGAPGHPGPPGPVGPSGK	S(5): 100.0; S(24): 0.0	-0,46	0,08
Vamp4	O70480	NLLEDDSDEEEDFFLR	S(7): 100.0	-0,47	0,11
Nfix	P70257-1	SPAAGSSQSSGWPNDVDAGPASLK	S(1): 93.5; S(6): 2.7; S(7): 2.7; S(9): 0.5; S(10): 0.5; S(22): 0.0	-0,47	0,10
Bsn	O88737	SADCSVQTDDEDNADWEQPVR	S(1): 0.0; S(5): 0.0; T(8): 100.0	-0,47	0,59
Kndc1	Q0KK55	VVNGPASPSESTSEEPGSQPEHSPSR	S(7): 0.0; S(9): 0.0; S(11): 0.0; T(12): 0.0; S(13): 0.0; S(18): 0.1; S(23): 50.0; S(25): 50.0	-0,47	-0,38
Prpf40b	D3Z4N6	TGWDTSESELSEGELER	T(1): 0.0; T(5): 0.0; S(6): 0.0; S(8): 0.0; S(11): 100.0	-0,47	-0,37
Lmnb2	P21619	ATSSSSSSSGVGMSVGQGR	T(2): 9.0; S(3): 79.6; S(4): 9.0; S(5): 1.1; S(6): 1.1; S(7): 0.1; S(8): 0.0; S(9): 0.1; S(14): 0.0	-0,47	-0,03
Map1a	Q9QYR6	WLAESPVGLPPEEEDK	S(5): 100.0	-0,47	0,11
Pfas	Q5SUR0	TGQGDAPLTPPTPVDLDLDWVLGK	T(1): 0.0; T(9): 87.2; T(12): 12.8	-0,47	0,66

Disease Models & Mechanisms • Supplementary information

Gramd1b	Q80TI0	SRSPTPQNQDGDTMVEK	S(1): 1.5; S(3): 96.9; T(5): 1.5; T(13): 0.0	-0,48	-0,41
Taf3	A2ASY0	GDSLDVVLLEAR	S(3): 100.0	-0,48	0,49
F2	H7BX99	TTDAEFHTFFNEK	T(1): 0.2; T(2): 1.5; T(8): 98.3	-0,48	-0,23
Rapgef2	E9QNQ4	SLGSLSQGSANATVLDVAQTGGHK	S(1): 97.9; S(4): 2.0; S(6): 0.1; S(9): 0.1; T(13): 0.0; T(20): 0.0	-0,48	-0,08
Gap43	P06837	KEGDGSATTDAAPATSPK	S(6): 0.0; T(8): 0.0; T(9): 0.0; T(15): 50.0; S(16): 50.0	-0,48	-0,36
Nav1	Q8CH77-2	SASSYSDIEEIATPDSSAPSSPK	S(1): 0.0; S(3): 0.0; S(4): 0.0; Y(5): 0.0; S(6): 0.0; T(13): 0.0; S(16): 0.0; S(17): 0.0; S(20): 50.0; S(21): 50.0	-0,48	0,30
Sdad1	Q80UZ2-2	YLEIDSDEESR	Y(1): 0.0; S(6): 99.9; S(10): 0.1	-0,48	0,04
Mcf2l	E9PXE2	ALEQSHSLPLPTPSSTSPTK	S(5): 0.0; S(7): 0.0; T(12): 0.2; S(14): 24.5; S(15): 24.5; T(16): 24.5; S(17): 24.5; T(19): 2.0	-0,48	0,00
Mroh2a	F5H8M8	CLGSLQGQVSSSAMAEGMEALTK	S(4): 0.0; S(10): 0.1; S(11): 3.5; S(12): 3.5; T(22): 93.0	-0,48	-0,36
Arhgap21	B7ZCJ1	DQGEPVSPSEDEPFSWPGPK	S(7): 100.0; S(9): 0.0; S(15): 0.0	-0,49	-0,04
Rims1	Q99NE5-5	STSVSGEIYTLEHNDGSQSDTAVGTVGAGGK	S(1): 0.0; T(2): 0.0; S(3): 0.0; S(5): 0.0; Y(9): 0.0; T(10): 0.3; S(17): 33.2; S(19): 33.2; T(21): 33.2; T(25): 0.3	-0,49	-0,10
Rims1	Q99NE5-5	ASQSSLESSSGPPCIR	S(2): 0.0; S(4): 0.6; S(5): 99.4; S(8): 0.0; S(9): 0.0; S(10): 0.0	-0,49	-0,40
Cdk11b	A2A9P6	DLLSDLQDISDSER	S(4): 0.0; S(10): 99.8; S(12): 0.2	-0,49	-0,09
Nucks1	Q80XU3	VVDYSQFQESDDADEDYGR	Y(4): 0.0; S(5): 0.0; S(10): 100.0; Y(17): 0.0	-0,50	0,30
Jph3	Q9ET77	EFSPSFQHR	S(3): 100.0; S(5): 0.0	-0,50	-0,20
Znf830	Q8R1N0	KEEENVDSDDEGELQDLLSQDWR	S(8): 100.0; S(19): 0.0	-0,50	0,37
Nebl	Q9DC07	GFSIVTDTPELQR	S(3): 0.0; T(6): 0.2; T(8): 99.8	-0,50	-0,39
Trim28	E9Q6J5	GSDDVLVSGEVPECEVGHMSPR	S(2): 0.0; S(8): 0.0; S(20): 100.0	-0,50	0,66
Pclo	Q9QYX7	LPTAVSLYSPTDEQSVMQK	T(3): 0.0; S(6): 0.0; Y(8): 0.4; S(9): 49.8; T(11): 49.8; S(15): 0.0	-0,50	0,28
Trim28	Q62318	RPAASSAAAASAAASSPAGGGGEAQELLEHCGVCR	S(5): 0.0; S(6): 0.0; S(11): 0.0; S(15): 50.0; S(16): 50.0	-0,50	0,73
Ptger4	Q6NS82-3	MASSGGGNTGAGGTSGLGLGLGLSLGMGEATGDAEEEAAAAEAVGR	S(3): 28.8; S(4): 28.8; T(9): 12.1; T(14): 12.1; S(15): 12.1; S(24): 5.6; T(31): 0.4	-0,50	0,39
Slc4a1	P04919	DHEEVLEIPDRDSEEELENIIGQIAYR	S(13): 100.0; Y(26): 0.0	-0,50	0,28
Akt2	Q91VE4	TSLGTEQHHAAAAAAVASVACR	T(1): 0.1; S(2): 0.5; T(5): 99.3; S(18): 0.0	-0,51	0,63
Otud4	Q60823	YFDDEFTAQSITITPPDRYDSLDPLELDQR	Y(1): 0.0; T(7): 0.0; S(10): 24.9; T(12): 24.9; T(14): 24.9; Y(19): 24.9; S(21): 0.2	-0,51	0,55
Slc4a8	B2RRE7	EESSEDENEVSNILR	S(3): 6.3; S(4): 93.7; S(11): 0.0	-0,51	0,36
Irf2bp1	Q8JZR6-2	DGQTVSPQSATNLEVK	T(4): 0.0; S(6): 100.0; S(9): 0.0; T(11): 0.0	-0,52	0,52
Skiv2l	Q6NZR5	LLEPLDLSGGDEDEGEAGGPR	S(8): 100.0	-0,52	0,20
Irf2bp1	Q8R3Y8-2	AGGASPAASSTTQPPAQHR	S(5): 91.5; S(9): 7.7; S(10): 0.1; T(11): 0.7; T(12): 0.1	-0,52	0,34
Akap12	Q9WTQ5	AEPEILELESK	S(10): 100.0	-0,52	0,39
Dlgap4	B1AZP2-2	SEVTSQSGLSNSSDSLDSSTRPPSVTR	S(1): 0.0; T(4): 0.0; S(5): 0.0; S(7): 0.0; S(10): 0.2; S(12): 0.2; S(13): 0.2; S(15): 0.0; S(18): 19.9; S(19): 19.9; T(20): 19.9; S(24): 19.9; T(26): 19.9	-0,52	-0,09
Cdc26	Q99JP4	QKEDVEGVGTSDGEGAAGLSSDPK	T(10): 9.0; S(11): 91.0; S(20): 0.0; S(21): 0.0	-0,52	0,80
Dlg1	H7BWY4	NLSEIENVHGFVSHSHISPIK	S(3): 0.0; S(13): 0.0; S(15): 50.0; S(18): 50.0	-0,52	0,25
Slc12a5	Q91V14	NKGPSPVSSEGIK	S(5): 100.0; S(8): 0.0; S(9): 0.0	-0,53	0,06
Slc12a5	Q91V14	ESSPFINSTDTEK	S(2): 50.0; S(3): 50.0; S(8): 0.0; T(9): 0.0; T(11): 0.0	-0,53	0,18
Pvrl1	Q9JKF6	AGIPQHHPPMAQNLQYPDDSDDEK	Y(16): 0.7; S(20): 99.3	-0,53	0,16
Purb	O35295	DSLGDFIEHYAQLGPSSPEQLAAGAEEGGGPR	S(2): 0.0; Y(10): 0.0; S(16): 8.3; S(17): 91.7	-0,53	0,24
Polr2a	Q8K019-2	ADGDWDDQEVLDYFSDKESAK	Y(13): 50.0; S(15): 50.0; S(19): 0.0	-0,55	0,55
Hook3	Q8BUK6	LNQSDSIEDPNSPAGR	S(4): 0.0; S(6): 0.0; S(12): 100.0	-0,55	-0,31
Polr2a	P08775	YSPTSPTYSPTSPK	Y(1): 0.5; S(2): 0.5; T(4): 98.6; S(5): 0.5; T(7): 0.0; Y(8): 0.0; S(9): 0.0; T(11): 0.0; S(12): 0.0	-0,55	0,34
Prdm2	A2A7B5	ETGSPPCFDEYK	T(2): 2.9; S(4): 97.1; Y(11): 0.0	-0,56	0,03
Ralgapa1	Q6GYP7	HFSQSEDTGNEVFGALHEEQPLPR	S(3): 99.4; S(5): 0.6; T(8): 0.0	-0,56	0,25

Disease Models & Mechanisms • Supplementary information

Pnn	O35691	EAGIVHSDAEK	S(7): 100.0	-0,56	-0,03
Cacna1c	Q01815-2	EAVSAASEDDIFR	S(4): 0.6; S(7): 99.4	-0,56	0,10
Pclo	Q9QYX7	DSFSQESSPSSPSDLAK	S(2): 0.0; S(4): 0.0; S(7): 0.0; S(8): 0.0; S(10): 5.0; S(11): 94.7; S(13): 0.3	-0,57	0,14
Marcks	P26645	VNGDASPAAAEPGAK	S(6): 100.0	-0,57	-0,11
Map1b	P14873	SPDTSAYCYETMEK	S(1): 95.0; T(4): 0.3; S(5): 4.7; Y(7): 0.0; Y(9): 0.0; T(11): 0.0	-0,57	0,01
Evc2	Q8K3H0	VNQSALEAVTPSPSFQQR	S(4): 0.0; T(10): 0.2; S(12): 89.4; S(14): 10.5	-0,57	1,60
Ciita	P79621-3	FTAAGAQQLASSLQK	T(2): 100.0; S(11): 0.0; S(12): 0.0	-0,57	0,19
Rrm1	Q8K1G2	VQGLLNTAATQLTSLIHK	T(7): 0.7; T(10): 7.3; T(13): 84.8; S(14): 7.3	-0,57	0,38
Pcm1	Q9R0L6	VTNAISPESSPGVGR	T(2): 0.0; S(6): 100.0; S(9): 0.0; S(10): 0.0	-0,58	-0,04
Map1a	P07742	ATGSYIAGTNGNSNGLVPMLRVYNNTAR	T(2): 33.3; S(4): 33.3; Y(5): 33.3; T(9): 0.0; S(13): 0.0; Y(23): 0.0; T(26): 0.0	-0,58	0,41
Caskin1	Q6P9K8-2	AQPGSPQALGGPHGPATAK	S(5): 100.0; T(17): 0.0	-0,58	-0,04
Kcnip4	Q6PHZ8	TSSPAIQNSVEDELEMATVR	T(1): 0.9; S(2): 49.6; S(3): 49.6; S(9): 0.0; T(18): 0.0	-0,59	-0,13
Serpinb6a	F8WIV2	LTIMDPLQEANGTFALNLLK	T(2): 50.0; T(13): 50.0	-0,59	-0,21
Fam169a	Q5XG69	SQSEEQSEASSEHLEQFTQSAEK	S(1): 0.0; S(3): 1.1; S(7): 9.1; S(10): 9.1; S(11): 80.5; T(18): 0.1; S(20): 0.0	-0,59	-0,39
Rmdn3	Q3UJU9	KDSLDLDVEAASSPAAAALEEDDSSGR	S(3): 100.0; S(12): 0.0; S(13): 0.0; S(24): 0.0; S(25): 0.0	-0,60	-0,21
Rb1cc1	Q9ESK9	VSTSQASPQSAASPR	S(2): 0.0; T(3): 0.0; S(4): 0.0; S(7): 100.0; S(10): 0.0; S(13): 0.0	-0,60	0,07
Bptf	E9Q6A7	SNGEVSESPGAGK	S(1): 0.0; S(6): 0.0; S(8): 100.0	-0,60	0,09
Arfgap1	Q9EPJ9-2	NSNSDGWESWEGASGEGR	S(2): 0.0; S(4): 100.0; S(9): 0.0; S(14): 0.0	-0,60	-0,41
Tle3	F6TS96	ESSTNNSVSPSESLR	S(2): 0.0; S(3): 0.0; T(4): 0.1; S(7): 7.1; S(9): 92.3; S(11): 0.6; S(13): 0.0	-0,61	-0,29
Map1a	Q9QYR6	EMTLDQKSPEK	T(3): 0.0; S(8): 100.0	-0,61	0,33
Aak1	Q3UHJ0-2	ILSDVTHSAVFGVPASK	S(3): 4.6; T(6): 47.7; S(8): 47.7; S(16): 0.0	-0,61	0,18
Tanc1	E9QAF9	QQGPPPAPANDSDNEEDAPASSLK	S(12): 100.0; S(21): 0.0; S(22): 0.0	-0,62	-0,13
Fxr1	Q61584-4	RGPNYTSGYGTNSELSNPSETESER	Y(5): 0.0; T(6): 0.0; S(7): 0.0; Y(9): 0.0; T(11): 0.0; S(13): 0.0; S(16): 0.2; S(19): 98.2; T(21): 1.5; S(23): 0.0	-0,63	-0,32
Svop	Q8BFT9	TGESARSEDDAASGEHDIQIEGVR	T(1): 0.0; S(4): 50.0; S(7): 50.0; S(13): 0.0	-0,64	-0,26
Vps9d1	D6RGB6	LQVAESQNSK	S(6): 100.0; S(9): 0.0	-0,64	0,23
Uncharacterized protein KIAA1143 homolog	Q99P72	LSASPQEVGKPYLESFQPNLHITK	S(2): 0.0; S(4): 100.0; Y(12): 0.0; S(15): 0.0; T(23): 0.0	-0,64	0,39
Dnajc6	Q80TZ3	GASSPDMEPSYGGGLFDMVK	S(3): 0.4; S(4): 99.6; S(10): 0.0; Y(11): 0.0	-0,64	0,27
Wipf2	Q8K039	NSSLLSFDSEDENE	S(2): 0.0; S(3): 0.0; S(6): 0.0; S(9): 100.0	-0,65	0,56
Muc5b	E9Q5I3	TSVSSPPTTPLTVTSVSTQHPK	T(1): 0.0; S(2): 0.0; S(4): 0.0; S(5): 0.0; T(8): 0.0; T(9): 0.1; T(12): 9.5; T(14): 90.3; S(15): 0.1; S(17): 0.0; T(18): 0.0	-0,66	-0,18
Krt19	Q6PEV3	QPPGVPNGPSSPTNESAPELPQR	S(10): 1.0; S(11): 49.4; T(13): 49.4; S(16): 0.1	-0,67	0,64
Smarcc2	P19001	GGSFSGTLAVSDGLLSGNEK	S(3): 0.0; S(5): 0.1; T(7): 0.0; S(11): 4.2; S(16): 95.7	-0,67	0,62
Kcnc1	E9PVV3	AALANEDCPHIDQALTPDEGLPFTR	T(16): 100.0; T(24): 0.0	-0,68	-0,06
Top2b	Q3UID0	DMDEPSPVPNVEEVTLPK	S(6): 100.0; T(15): 0.0	-0,69	0,41
Tspyl4	Q8VD63	GLEDAPSPSK	S(7): 95.3; S(9): 4.7	-0,69	0,11
Top2b	Q64511	FDSNEEDTASVFAPSFGLK	S(3): 100.0; T(8): 0.0; S(10): 0.0; S(15): 0.0	-0,69	0,60
Tiprl	Q5U3K5	EDLSDVTDEDTGPAQPPPPSK	S(4): 100.0; T(7): 0.0; T(11): 0.0; S(20): 0.0	-0,70	1,15
Fmn2	Q9JL04	SADWTEELGVR	S(1): 100.0; T(5): 0.0	-0,71	-0,15
Col18a1	Q8BH58	IDPNPVDSQSTPSE	S(8): 0.1; S(10): 0.1; T(11): 0.7; S(13): 99.1	-0,71	0,94
Col18a1	P39061-2	DGEPGDPGEDGRPGDTGPQGFPGTPGDVGPK	T(16): 1.3; T(24): 98.7	-0,72	0,42
Ckb	Q04447	RGTGGVDTAAVGGVFDVSNADR	T(3): 97.0; T(8): 3.0; S(18): 0.0	-0,73	-0,04
Trp53bp1	A2AU91	STPFIVPSSPTEQGGR	S(1): 0.0; T(2): 0.0; S(8): 49.9; S(9): 49.9; T(11): 0.2	-0,73	0,04
Cacna1g	Q5SUF8	SESEPDFFSPSVDGDGDR	S(1): 0.0; S(3): 100.0; S(9): 0.0; S(11): 0.0	-0,74	0,89
Kcnd2	Q9Z0V2	LQDDADTDNTGESALPTMTAR	T(7): 100.0; T(10): 0.0; S(13): 0.0; T(17): 0.0; T(19): 0.0	-0,74	0,24

Disease Models & Mechanisms • Supplementary information

Shisa6	F6VQZ6	QYNHPILSSATQTPTHEKPR	Y(2): 0.0; S(8): 0.0; S(9): 0.0; T(11): 0.0; T(13): 100.0; T(15): 0.0	-0,74	0,30
Dock4	P59764	ASPLLSDK	S(2): 100.0; S(6): 0.0	-0,76	0,25
Aak1	Q3UHJ0-2	EQGSSGLGSGSSGGGGSSSGLGSGYIGR	S(4): 0.0; S(5): 0.0; S(9): 47.3; S(11): 47.3; S(12): 5.5; S(17): 0.0; S(18): 0.0; S(19): 0.0; S(23): 0.0; Y(25): 0.0	-0,76	0,66
Plekha6	P16014	WASSREDAGAPVEDSQGQTK	S(3): 50.0; S(4): 50.0; S(15): 0.0; T(19): 0.0	-0,77	1,41
Ppp1r17	Q9Z2E4	LDPQCSPSDDLSDQFIK	S(6): 99.3; S(8): 0.7; S(12): 0.0	-0,77	-0,29
CR025	Q8BH50	RDSSESQLASTESDKPTTGR	S(3): 50.0; S(4): 50.0; S(6): 0.0; S(10): 0.0; T(11): 0.0; S(13): 0.0; T(17): 0.0; T(18): 0.0	-0,79	-0,24
Kat7	Q5SVQ0-4	SSGSETEQVVDFSDR	S(1): 0.6; S(2): 98.7; S(4): 0.6; T(6): 0.1; S(13): 0.0	-0,80	-0,35
Plcl2	Q8K394	EFQVSFQVQK	S(5): 100.0	-0,81	-0,06
Gas7	B1ATI9	STGDSQNLGSSSPGR	S(1): 0.0; T(2): 0.0; S(5): 0.0; S(10): 8.2; S(11): 8.2; S(12): 83.5	-0,81	-0,26
Pclo	Q9QYX7	GGSLGCQTETDPDTQSPPYMGATSPPKDK	S(3): 0.0; T(8): 0.0; T(10): 0.0; T(14): 20.0; S(16): 20.0; Y(19): 20.0; T(23): 20.0; S(24): 20.0	-0,81	0,30
Olfr777	Q8VFH3	QAFNDSVK	S(6): 100.0	-0,87	-0,08
Kif17	A2AM72	EYQEEIK	Y(2): 100.0	-0,87	-0,03
Plekha6	Q7TQG1	SIHEVDISNLEAALR	S(1): 100.0; S(8): 0.0	-0,87	0,61
N4bp3	A2A699	TGLCSPEDNSLTPLLDEVVAPEGR	T(1): 3.3; S(5): 92.8; S(10): 0.7; T(12): 3.3	-0,94	1,30
Dmxl2	Q8C7U1	AQLLHALSLDEGGPEPSLSDSSSGGSFGR	S(8): 0.8; S(17): 58.2; S(19): 12.6; S(21): 12.6; S(22): 12.6; S(23): 3.0; S(26): 0.2	-0,95	0,99
Fam131b	Q3TY60-2	KVSDVTSSGVQSFDEEEGDANN	S(3): 0.0; T(6): 0.0; S(7): 0.0; S(8): 0.0; S(12): 100.0	-1,01	-0,20
Dmxl2	Q8BPN8-2	ALLTPQDEECADGDTEVDVIAEQLK	T(4): 99.3; T(15): 0.7	-1,02	0,42
Scaf8	Q6DID3	ASEPVKEPVQTAQSPAPVEK	S(2): 0.0; T(11): 1.2; S(14): 98.8	-1,04	0,56
Sptbn2	Q68FG2	WDLPDSDWDNDSSSAR	S(6): 100.0; S(12): 0.0; S(13): 0.0; S(14): 0.0	-1,07	-0,37
Osbp	Q3B7Z2	GDMSDEDDENEFFDAPEIITMPENLGHK	S(4): 100.0; T(20): 0.0	-1,08	0,85
Pafah1b2	A2AHG0	GAAGGSSTPTPQHGEEK	S(6): 33.3; S(7): 33.3; T(8): 33.3; T(10): 0.0	-1,09	0,69
Sirt2	Q61206	ELFSPLHALNFGIGGDTTR	S(4): 100.0; T(17): 0.0; T(18): 0.0	-1,12	0,45
Slc6a11	P31650	GSETLGGGGGGAAGTR	S(2): 100.0; T(4): 0.0; T(15): 0.0	-1,12	-0,36
Sirt2	Q8VDQ8-2	REHANIDAQSGSQAPNPSTTISPGK	S(10): 0.0; S(12): 0.0; S(18): 47.9; T(19): 47.9; T(20): 3.9; S(22): 0.3	-1,17	0,77
Add1	Q9QYC0	SPPDQSAVPNTPPSTPVKLEEDLPQEPTSR	S(1): 0.0; S(6): 0.5; T(11): 33.2; S(14): 33.2; T(15): 33.2; T(28): 0.0; S(29): 0.0	-1,29	-0,16
Bsn	O88737	RPHSLSITPEAFDSDEELGDILEEDDSLAWGR	S(4): 0.0; S(6): 0.0; T(8): 0.0; S(14): 100.0; S(27): 0.0	-1,33	1,00
Cacna1g	F6RJ39	APVVLQPEQIVSEEETPPPLLTK	S(12): 0.0; T(16): 100.0; T(22): 0.0	-1,34	0,92
Ap3d1	O54774	VDIITEEMPENALPSDEDDKDPNDPYR	T(5): 0.0; S(15): 100.0; Y(26): 0.0	-1,60	-0,41
Il16	O54824	SASPETPASPGK	S(1): 0.9; S(3): 98.2; T(6): 0.9; S(9): 0.0	-1,72	0,19

Disease Models & Mechanisms • Supplementary information

Supplementary Table S4. Proteins up and down phosphorylated in the hippocampus of Ts65Dn mice and reverted by L41 treatment.

Gene name	Protein Group Accessions	Sequence	phosphoRS Site Probabilities	Ts65Dn vs WT	Ts65Dn +/-L41
Sart1	Q9Z315	ASSGDASSLSIEETNK	S(2): 20.0; S(3): 20.0; S(7): 20.0; S(8): 20.0; S(10): 20.0	4,08	-3,82
Synpr	D3Z5Q8	QASDWHSLSTLQLSLGLYK	S(3): 10.1; S(7): 89.9; S(9): 0.0; S(14): 0.0	2,79	-2,95
Eif2ak4	A2AUM0	EILDGLAYIHEK	Y(8): 100.0	2,74	-2,62
Fryl	F8VQ05	EDVLSGFVYFIVR	S(5): 0.0; Y(9): 100.0	2,68	-1,37
Specc1	Q5SXY1	GSPTGSSPNNASELSLASLTEK	S(2): 23.7; T(4): 23.7; S(6): 23.7; S(7): 23.7; S(12): 4.4; S(15): 0.9; S(18): 0.0; T(20): 0.0	2,59	-1,89
Tcp11l1	Q8BTG3	QEAENGALDISK	S(11): 100.0	2,48	-1,94
Myo1c	Q9WTI7-2	RDQAVMISGESGAGK	S(8): 92.4; S(11): 7.6	2,37	-2,01
Crocc	Q8CJ40	TADTSDGSLRGFSGQR	T(1): 0.1; T(4): 3.7; S(5): 66.8; S(8): 14.7; S(13): 14.7	2,12	-1,68
Clip2	Q9Z0H8	TGNESGSNLSDSGSVK	S(5): 0.0; S(7): 91.1; S(10): 8.9; S(12): 0.0; S(14): 0.0	2,08	-3,05
Cys1	Q8R4T1	DPRPATPSGGR	T(6): 91.3; S(8): 8.7	1,87	-2,15
Sipa1l1	Q8C0T5	RHQSDGNEIAHTR	S(4): 100.0; T(12): 0.0	1,84	-1,42
Klhl41	A2AUC9	LYQSTLLQDGLK	Y(2): 0.5; S(4): 6.3; T(5): 93.2	1,35	-1,11
Sirt2	Q8VDQ8	REHANIDAQSGSQAPNPSTTISPGKSPPPAK	S(10): 0.0; S(12): 0.0; S(18): 0.7; T(19): 0.7; T(20): 32.9; S(22): 32.9; S(26): 32.9	1,33	-0,84
Palmd	Q9JHU2	SNGPTHTSPTRPTPQPR	S(1): 0.0; T(5): 0.3; T(7): 49.7; S(8): 49.7; T(10): 0.3; T(13): 0.0	1,15	-1,30
Arhgef2	H3BJU7	LSPPHSPR	S(2): 100.0; S(6): 0.0	1,13	-1,17
Begain	F8WIG2	YYGGGGGGGAAGGGSPGDKAEGR	S(15): 100.0	1,13	0,53
Enah	Q03173-4	NSRPSSPVNTPSSQPPAAK	S(2): 0.0; S(5): 5.0; S(6): 95.0; T(10): 0.0; S(12): 0.0; S(13): 0.0	1,11	-0,42
Cdh10	P70408	RTPTAPDNTDVR	T(2): 100.0; T(4): 0.0; T(9): 0.0	1,10	-1,16
Map1b	P14873	SEQSSMSIEFGQESPEHSLAMDFSR	S(1): 0.0; S(4): 0.0; S(5): 0.4; S(7): 49.6; S(14): 49.6; S(18): 0.4; S(24): 0.0	1,06	-0,17
Apoa1bp	Q8K4Z3	RGSETMAGAAVK	S(3): 50.0; T(5): 50.0	1,03	0,44
C2cd2l	Q80X80	SLSPAATVTAELHYEQGSPR	S(1): 0.0; S(3): 0.0; T(7): 0.0; T(9): 0.0; Y(14): 0.0; S(18): 100.0	1,03	-0,82
Sipa1l3	G3X9J0	SSSEITLSECDVEEPGDPR	S(1): 8.5; S(2): 8.5; S(3): 83.1; T(6): 0.0; S(8): 0.0	0,97	-0,62
Col4a1	P02463	GFPGPPGPDGLPGSMGPPGTPSVDHGFLVTR	S(14): 98.9; T(20): 1.1; S(22): 0.0; T(30): 0.0	0,96	-0,64
Smg6	P61406	QVSLSSTDSLDEDR	S(3): 1.5; S(5): 10.8; S(6): 86.1; T(7): 1.5; S(9): 0.0	0,96	-0,55
Rltpr	Q3V3V9-2	LEAPPSPSLGSGLGSKPLPPYPTEPSSPER	S(6): 0.0; S(8): 0.0; S(11): 0.0; S(15): 0.0; S(26): 50.0; S(27): 50.0	0,94	-0,11
1700065D16Rik	Q7TSG0	QPPPAPGFSTAASGGEGGASTEPR	S(9): 80.9; T(10): 18.4; S(13): 0.3; S(20): 0.3; T(21): 0.0	0,93	-0,61
Hn1	P97825	SNSSEASSGDFLDLK	S(1): 0.0; S(3): 50.0; S(4): 50.0; S(7): 0.0; S(8): 0.0	0,92	-0,66
Gbf1	Q6A099	ADAPDAGAQSDSELPSYHQNDVSLDR	S(10): 49.7; S(12): 49.7; S(16): 0.3; Y(17): 0.3; S(23): 0.0	0,89	-0,60
Stk32c	Q8QZV4	RGSSAAAPPSSPPPGR	S(3): 0.0; S(4): 0.0; S(10): 0.0; S(11): 100.0	0,88	-1,06
Grin2b	G3X9V4	RQHSYDTFVDLQK	S(4): 48.4; Y(5): 48.4; T(7): 3.2	0,88	-0,69
Cask	O70589	TQSSSCERDSPSTSR	T(1): 0.8; S(3): 0.8; S(4): 0.0; S(5): 0.0; S(10): 97.7; S(12): 0.0; T(13): 0.8; S(14): 0.0	0,86	-0,71
Syk	E9PWE9	ELNGTYAISGGR	T(5): 50.0; Y(6): 50.0; S(9): 0.0	0,84	-0,52
Lgalsl	Q8VED9	LDDGHLNNSLGSPVQADVYFPR	S(9): 0.0; S(12): 100.0; Y(19): 0.0	0,83	0,17
Gphn	A0JNY3	DTASLSTTPSESPR	S(4): 0.0; S(6): 0.0; S(10): 0.9; S(12): 99.0	0,79	-1,00
Tnks1bp1	P58871	SAEEGEVTESK	S(1): 0.0; S(10): 100.0	0,78	-0,51
Prrc2c	Q3TLH4-5	ATSTSPNSQSSK	T(2): 0.0; S(3): 0.0; T(4): 4.6; S(5): 95.4; S(8): 0.0; S(10): 0.0; S(11): 0.0	0,76	-1,08
R3hdm2	Q80TM6-2	SLEPRPWSSTDSDGSVR	S(1): 0.0; S(8): 33.3; S(9): 33.3; T(10): 33.3; S(12): 0.2; S(15): 0.0	0,73	-0,45
Slc7a14	Q8BXR1	HKQNSEALIANDELDCSPE	S(5): 0.0; S(17): 100.0	0,72	-0,14
Map1a	Q9QYR6	SPESLSSPAMEDLAMEWGGK	S(1): 0.0; S(4): 0.3; S(6): 5.0; S(7): 94.7	0,72	-0,03
Ube2w	D6RCG4	ELLALQNDPPPGMTLNEK	T(14): 100.0	0,71	-0,52
Cacnb2	Q8CC27	TLASSTLPLSPTLASNSQGSQGDQRPDR	S(4): 85.0; S(5): 14.4; S(10): 0.5; S(15): 0.0; S(17): 0.0; S(20): 0.0	0,70	-0,76
Wipf2	Q6PEV3	HSSSAPPPPPPGR	S(2): 0.3; S(3): 0.3; S(4): 99.3	0,70	-0,68
Cep170b	Q80U49	HEDGTQSDSEDPLAK	T(5): 33.3; S(7): 33.3; S(9): 33.3	0,69	-1,01

Disease Models & Mechanisms • Supplementary information

Atad3a	H3BKE0	TPTLVDSSSETDLR	T(1): 2.2; T(3): 10.4; S(7): 10.4; S(8): 10.4; S(9): 10.4; T(11): 56.0	0,69	-0,21
Prkcb	P68404	QPVELTPTDK	T(6): 100.0; T(8): 0.0	0,68	-1,25
Dnaic2	R4GML5	ISEPIEVVIMDISR	S(2): 100.0; S(13): 0.0	0,68	-0,43
Cep170b	Q80U49	HEDGTQSDSEDPLAK	T(5): 0.8; S(7): 98.5; S(9): 0.8	0,67	-0,88
Tubb6	Q922F4	LTTPTYGDLNHLVSATMSGVTTSLR	T(2): 0.0; T(3): 0.0; T(5): 0.0; Y(6): 0.0; S(14): 1.7; T(16): 10.2; S(18): 66.1; T(21): 10.2; T(22): 10.2; S(23): 1.7	0,67	-1,09
Bsn	O88737	SPQVLYSPVSPLSPHR	S(1): 0.0; S(7): 0.0; S(10): 0.2; S(13): 99.8	0,67	-0,19
Pfas	Q5SUR0	TGQGDAPLTPPTPVDLDLDWVLGK	T(1): 0.0; T(9): 1.1; T(12): 98.9	0,67	0,12
Irf2bpl	Q8K3X4	NSSSPVSPASVPGQR	S(2): 0.1; S(3): 0.3; S(4): 0.3; S(7): 3.6; S(10): 95.8	0,67	-1,18
Nudt3	Q9JI46	SESEEEVLLVSSSR	S(1): 0.0; S(3): 100.0; S(11): 0.0; S(12): 0.0; S(13): 0.0	0,65	0,34
Tbc1d22a	Q8R5A6	SVSESHTPCPSESTGDTVPLQR	S(1): 1.6; S(3): 85.6; S(5): 11.2; T(7): 1.6; S(11): 0.0; S(13): 0.0; T(14): 0.0; T(17): 0.0	0,65	-0,43
Ace	P09470	GPQFGSEVELR	S(6): 100.0	0,65	-0,44
Nktr	P30415	SRASSSSSHHSSK	S(1): 0.0; S(4): 0.0; S(5): 0.0; S(6): 0.0; S(7): 1.2; S(8): 1.2; S(11): 87.9; S(12): 9.7	0,64	-0,64
Arhgef28	G5E8P2	LSLPMAAGHGGDAGDGAEENILYL	S(2): 96.9; Y(23): 3.1	0,64	-0,23
Scg2	Q03517	EHLGPGSSQEMER	S(7): 2.8; S(8): 97.2	0,62	0,26
Prkce	P16054	KLAAGAESPQPASGNSPSEDDR	S(8): 100.0; S(13): 0.0; S(16): 0.0; S(18): 0.0	0,62	0,23
Vps35	Q9EQH3	RESPESEGPIYEGLIL	S(3): 93.6; S(6): 6.4; Y(11): 0.0	0,62	-0,45
Dmtn	Q9WV69	KGAEEEEEEEDDDSEEEIK	S(14): 100.0	0,62	-1,21
Fam171a2	A2A699	SVEGPGGLEPSLDEYR	S(1): 100.0; S(11): 0.0; Y(15): 0.0	0,60	-0,15
Sept2	P42128	EGSPIPHDPDLGSK	S(3): 100.0; S(13): 0.0	0,60	-0,36
Polk	Q9QUG2-2	TRASTVPAAISTAEEIFAIAK	T(1): 80.3; S(4): 9.8; T(5): 9.8; S(11): 0.0; T(12): 0.0	0,60	-0,78
Rrp12	Q6P5B0	AMDLAQAGSTVESK	S(9): 0.0; S(13): 100.0	0,60	-0,32
Aak1	Q3UHJ0-2	ILSDVTHSAVFGVPASK	S(3): 0.4; T(6): 93.9; S(8): 5.7; S(16): 0.0	0,59	-0,02
Kcnab2	P62482	MYPESTTGSPAR	S(5): 0.1; S(9): 99.9	0,58	0,23
Aldh1a2	Q62148	LAFSLGSVWR	S(4): 100.0; S(7): 0.0	0,58	-0,19
Pvrl1	Q9JKF6	AGIPQHHPPMAQNLQYPDDSDDEK	Y(16): 0.0; S(20): 100.0	0,58	-0,74
Lmna	P48678	LRLSPSPTSQR		0,58	-0,14
Ap3d1	O54774	HSSLPTESDEDIAPAQR	S(2): 0.0; S(3): 0.0; T(6): 0.0; S(8): 100.0	0,58	-0,79
Sept9	A2A6U3	SFEVEEIEPPNSTPPR	S(1): 100.0; S(12): 0.0; T(13): 0.0	0,58	-0,58
Hcn2	O88703	RDSASPGAASGLDPLDSAR	S(3): 8.9; S(5): 91.1; S(10): 0.0; S(17): 0.0	0,58	-0,19
Ppp1r18	Q8BQ30	EEAEAQSRPLR		0,56	-0,38
Utp20	Q5XG71	EVMEANLPSILLK	S(9): 100.0	0,56	-0,75
Gm14597	B1AXE0	VLAFVEEDPLSHELK		0,55	-0,69
Map1b	P14873	TTRSPDTSAYCYETMEK	T(1): 0.2; T(2): 0.2; S(4): 99.5; T(7): 0.0; S(8): 0.0; Y(10): 0.0; Y(12): 0.0; T(14): 0.0	0,55	-0,56
Eno1	P17182	YDLDFKSPDDPSR		0,55	-0,26
Nap1l4	Q78ZA7	ASGDGESLDEDSEFTLASDFEIGHFFR	S(2): 0.0; S(7): 100.0; S(12): 0.0; T(15): 0.0; S(18): 0.0	0,55	-0,34
Arhgef12	F8VQN6	SEGVQDAQEPQSLVGSPSTR	S(1): 0.0; S(12): 49.0; S(16): 49.0; S(18): 1.0; T(19): 1.0	0,55	0,02
Sept2	P42208	IYHLPDAESDEDEDFKEQTR	S(9): 100.0	0,54	-0,42
Prkce	P16054	LAAGAESPQPASGNSPSEDDR	S(7): 100.0; S(12): 0.0; S(15): 0.0; S(17): 0.0	0,54	-0,02
Sdc4	O35988	VSMSSTAQGSNIFER	S(2): 0.7; S(4): 0.7; S(5): 5.4; T(6): 46.6; S(10): 46.6	0,54	-0,21
Aff4	Q9ESC8	LSPGNSGSYSSGGSSASASGSSVTIPQK	S(2): 0.7; S(6): 0.1; S(8): 2.9; S(10): 12.6; S(11): 58.7; S(14): 12.6; S(15): 2.9; S(17): 2.9; S(19): 2.9; S(21): 2.9; S(22): 0.7	0,53	-0,55
Ap2a1	P17426-2	RDTSSNDINGGVEPTPSTVSTPSPSADLLGLR	T(3): 0.0; S(4): 0.0; S(5): 0.0; T(15): 0.0; S(17): 1.5; T(18): 1.5; S(20): 1.5; T(21): 10.7; S(23): 83.5; S(25): 1.5	0,53	-0,21
Slc2a13	Q3UHK1	GSNYHLSDNDASDVE	S(2): 0.0; Y(4): 0.0; S(7): 0.0; S(12): 100.0	0,52	-0,60
Lppr4	Q7TME0	MSLQVMDTEPEGQSPPR	S(2): 0.0; T(8): 0.0; S(14): 100.0	0,51	-0,06
Zfp36l1	P23950	AVGTPAGGGFPR	T(4): 100.0	0,50	-0,67
Tsg101	Q61187	QILNLYAEENAIEDTIFYLGEALR	Y(6): 50.0; T(15): 50.0; Y(18): 0.0	0,50	-0,39
Slc1a4	O35874	SEEETSPLVTHQNPAGPVAIAPELESK	S(1): 0.0; T(5): 98.9; S(6): 1.1; T(10): 0.0; S(26): 0.0	0,50	-0,41
Hepacam	Q640R3	STTEPGPPGYSVSPPVPGRSPGLPIR	S(1): 0.0; S(11): 0.0; S(13): 0.0; S(20): 100.0	0,49	-0,07

Disease Models & Mechanisms • Supplementary information

Sirt2	Q8VDQ8	EHANIDAQSGSQAPNPSTTISPGKSPPPAK	S(9): 0.0; S(11): 0.0; S(17): 42.8; T(18): 42.8; T(19): 6.6; S(21): 1.1; S(25): 6.6	0,49	-0,17
Slc17a6	Q8BLE7	YASILMGISDGVGTLSGMVCPIIVGAMTK		0,49	-0,46
Nsmf	A2AJ96	MYSVDGVSDDVPIR	Y(2): 0.0; S(3): 100.0; S(8): 0.0	0,48	0,23
Ppme1	Q8BVQ5	QCEGITSPEGSK	T(6): 4.7; S(7): 95.3; S(11): 0.0	0,47	-0,24
CF057	Q8BTE0	FDSLEDSPEEREPLQK	S(3): 100.0; S(7): 0.0	0,47	0,27
Aldoa	P05064	GILAADESTGSIAK	S(8): 0.3; T(9): 5.5; S(11): 94.2	0,47	-0,01
Gpr123	Q8C4G9	ALPCPGADQPPYPK	Y(12): 100.0	0,45	0,22
Atcay	Q8BHE3	LGGAVEDSSSPPSTLNLSGAHR	S(8): 0.5; S(9): 0.5; S(10): 99.1; S(13): 0.0; T(14): 0.0; S(18): 0.0	0,44	-0,12
Vps13d	B1ART1	EKDDVSPQPLMTDLER	S(6): 100.0; T(12): 0.0	0,44	-0,30
Hnrnpa0	Q9CX86	LFIGGLNVQTSESGLR		0,44	-0,25
Sbf1	Q6ZPE2	ALYLEPSDGVSPTQETGEAQSQDDER	Y(3): 0.1; S(7): 82.3; S(11): 8.8; T(13): 8.8; T(16): 0.0; S(21): 0.0	0,44	-0,13
Kcna2	P63141	ETEGEEQAQYLQVTSCPK	T(2): 0.0; Y(10): 0.0; T(14): 0.0; S(15): 100.0	0,43	-0,27
Gm15800	E9Q2E4	TEGTPPPPGQPAK	T(1): 0.8; T(4): 99.2	0,43	-0,74
Wwp1	Q8BZZ3	SSSPPIEIQQNGDALHENGDPATR	S(1): 0.4; S(2): 49.8; S(3): 49.8; T(23): 0.0	0,43	-0,38
Vmn1r84	Q8R284	HLTLANSLVIISR		0,43	-0,34
Nlrp5	Q9R1M5-7	LLFSNQNLK	S(4): 100.0	0,42	-0,13
Pigx	F6WL03	LAADFSDAPFSAVCPQQLLTR	S(6): 49.7; S(11): 49.7; T(20): 0.7	0,42	-0,17
Rgs14	P97492	EPGSSHLGSPDTAR	S(4): 0.0; S(5): 0.7; S(9): 99.2; T(12): 0.0	0,42	-0,11
Rab12	P35283	RPAGGSLGAVSPALSGGQAR	S(6): 100.0; S(11): 0.0; S(15): 0.0	0,42	-0,02
Apoe	P08226	NEVHTMLGQSTEEIR	T(5): 0.0; S(10): 50.0; T(11): 50.0	0,42	-0,21
Mtdh	Q80WJ7	SETNWESPK	S(1): 0.0; S(7): 100.0	0,41	0,03
Arfip2	Q8K221	HPSHSTSPSGPGDEVAR	S(3): 0.0; S(5): 0.4; T(6): 49.8; S(7): 49.8; S(9): 0.0	0,41	-0,44
Iqsec2	A4GZ26	SSSPGAGGGHSTSTSTSPATTLQR	S(1): 7.9; S(2): 46.1; S(3): 46.1; S(11): 0.0; T(12): 0.0; S(13): 0.0; T(14): 0.0; S(15): 0.0; T(16): 0.0; S(17): 0.0; T(20): 0.0; T(21): 0.0	0,40	0,17
Pclo	Q9QYX7	TIELNSTVTDK	S(6): 100.0	0,40	0,37
Stim1	P70302	LIEGVHPGSLVEK		0,39	-0,16
Gprin1	Q3UNH4	SVATGPMTPQAAAPPAVPPVFPEVR	S(1): 0.2; T(4): 10.3; T(8): 89.6	0,39	-0,27
Krt26	Q3TRJ4	EALSEELTYLQK	S(4): 0.0; T(8): 50.0; Y(9): 50.0	0,39	0,20
Dmtn	Q9WV69	LQSTEFSPSGSEAGSPGLQNGEGQR	S(3): 0.1; T(4): 0.8; S(7): 99.1; S(9): 0.1; S(11): 0.0; S(15): 0.0	0,39	-0,27
Abi2	P62484	ALFDSYTNLER	S(5): 33.3; Y(6): 33.3; T(7): 33.3	0,39	0,08
Rasal1	Q9Z268	LLEDSSLDTEVDPGR	S(5): 7.6; S(6): 92.4; T(9): 0.1	0,39	-0,78
Uncharacterized protein C4orf21 homolog	Q0VGT4	SSSELSEVINNMSLLK	S(1): 0.5; S(2): 2.3; S(3): 12.9; S(6): 0.5; S(13): 83.9	0,38	-0,06
Stx1a	O35526	TTTSEELEDMLESGNPAIFASGIIMDSSISK	T(1): 42.2; T(2): 42.2; T(3): 7.1; S(4): 7.1; S(13): 1.3; S(21): 0.0; S(27): 0.0; S(28): 0.0; S(30): 0.0	0,38	0,08
Fam122a	Q9DB52	SNSAPLIHGLSDSSPVFQAEAPSAR	S(1): 9.9; S(3): 90.1; S(11): 0.0; S(13): 0.0; S(14): 0.0; S(23): 0.0	0,38	-0,54
Sptbn1	Q62261	ESSPVPSPTLDR	S(2): 93.7; S(3): 6.3; S(7): 0.0; T(9): 0.0	0,38	-0,22
Rps6kc1	Q8BLK9-2	SSQESLDIK	S(1): 0.0; S(2): 5.7; S(5): 94.3	0,38	-0,11
Mycbpap	Q5SUV2-2	LAAHEAITIAQSVLQDLLR	T(8): 50.0; S(12): 50.0	0,38	-0,02
Hecw2	Q6I6G8	SNSIQQMEQLNR	S(1): 0.1; S(3): 99.9	0,38	-0,24
Diras2	Q5PR73	CDESPNREVQSSEAEALAR	S(4): 100.0; S(11): 0.0; S(12): 0.0	0,38	0,09
Atp8a1	P70704	TDDVSEKTSLADQEEVR	T(1): 0.0; S(5): 33.3; T(8): 33.3; S(9): 33.3	0,37	-0,16
Uba1	Q02053	IHVSDQELQSANASVDDSR	S(4): 99.4; S(10): 0.6; S(14): 0.0; S(18): 0.0	0,37	-0,48
Pgbd5	D3YZI9	ELLGLEDSSPAH	S(8): 4.5; S(9): 95.5	0,37	-0,10
Acot7	Q91V12-2	MTFTSNK	T(2): 100.0; T(4): 0.0; S(5): 0.0	0,37	-0,03
Mybl1	E9QLX9	SCKQEHSASVK	S(1): 0.0; S(7): 7.8; S(9): 92.2	0,37	-0,37
Pacsin1	Q61644	STSGPGMPMNWPQFEEWNPDLPHTTAK	S(1): 0.0; T(2): 0.0; S(3): 1.1; T(24): 97.9; T(25): 1.1	0,36	-0,18
Enah	Q03173-4	NSRPSSPVNTPSSQPPAAK	S(2): 3.1; S(5): 48.5; S(6): 48.5; T(10): 0.0; S(12): 0.0; S(13): 0.0	0,36	-0,44
Wdr44	Q6NVE8	EYVSNDATQSDDEEKLQSQQTDTDGGR	Y(2): 0.0; S(4): 0.4; T(8): 49.8; S(10): 49.8; S(18): 0.0; T(21): 0.0; T(23): 0.0	0,36	-0,08
Gga3	Q8BMI3	TLIPSPPPRPK		0,36	-0,15
Itm2c	Q91VK4	AAASGPASASAPAAEILLTPAR	S(4): 0.0; S(8): 0.0; S(10): 0.0; T(19): 100.0	0,36	0,14

Disease Models & Mechanisms • Supplementary information

Ncor2	E9Q9V3	EGTPPPPPPPR	T(3): 100.0	0,36	-0,57
Abcf1	Q6P542	SKPAAADSEGEEEEEDTAK	S(1): 0.0; S(8): 100.0; T(17): 0.0	0,36	0,32
Fam117b	Q3U3E2	TSPTVATQTGASVTSTR	T(1): 1.2; S(2): 98.6; T(4): 0.2; T(7): 0.0; T(9): 0.0; S(12): 0.0; T(14): 0.0; S(15): 0.0; T(16): 0.0	0,35	0,27
Slc4a4	E9Q8N8	MFSNPDNGSPAMTHR	S(3): 0.0; S(9): 100.0; T(13): 0.0	0,35	-0,28
Slc9a6	A1L3P4	LVLPMDDSEPALNSLDDTRHSPA	S(8): 0.0; S(14): 0.0; T(18): 0.7; S(21): 99.3	0,35	0,37
Pacsin1	Q61644	GRLDSGQLGLYPANYVEAI	S(5): 100.0; Y(11): 0.0; Y(15): 0.0	0,35	0,07
Mug2	P28666	FDLAFSSEVSGTLQK	S(6): 49.8; S(7): 49.8; S(10): 0.3; T(12): 0.0	0,34	-0,02
Rph3a	P47708	WHQLQNENHVSSD	S(11): 50.0; S(12): 50.0	0,34	-0,13
Son	Q9QX47-3	ESAQAVAVALSPK	S(2): 0.0; S(11): 100.0	0,34	-0,10
Map1b	P14873	SQGSTSNSDWMK	S(1): 0.0; S(4): 93.3; T(5): 5.9; S(6): 0.4; S(8): 0.4	0,34	0,13
Rab3a	P63011	MSESLDTADPAVTGAK	S(2): 100.0; S(4): 0.0; T(7): 0.0; T(13): 0.0	0,34	0,06
Srrm2	Q8BTI8-3	SGAGSPPGK	S(1): 0.0; S(5): 100.0	-0,67	0,12
Commd7	A2AU52	FGVTSGSSELEK	T(4): 0.0; S(5): 0.0; S(7): 95.0; S(8): 5.0	-0,67	0,37
Ablim3	Q69ZX8	YTSDEMLER	Y(1): 3.9; T(2): 92.2; S(3): 3.9	-0,67	0,28
Khsrp	Q3U0V1	VQISPDSGGLPER	S(4): 100.0; S(7): 0.0	-0,67	0,30
Lrig3	Q6P1C6	NVDGLTFQGLGALK	T(6): 100.0	-0,67	0,05
Cacna1a	P97445	EMGTDGYSDSEHYLPMEGQTR	T(4): 24.9; Y(7): 24.9; S(8): 24.9; S(10): 24.9; Y(13): 0.3; T(20): 0.0	-0,68	0,27
Fam21	Q6PGL7	LAAQESSEAEDVTVDR	S(6): 50.0; S(7): 50.0; T(13): 0.0	-0,68	0,15
Pgk1	P09411	ALESPERPFLAILGGAK	S(4): 100.0	-0,68	0,33
Sparcl1	P70663	AAGSKEHIPHTEQQDQEGK	S(4): 100.0; T(11): 0.0	-0,68	-0,05
Sparcl1	P70663	GKESQEQPVSDSHQQPNESSK	S(4): 24.9; S(10): 24.9; S(12): 24.9; S(19): 24.9; S(20): 0.2	-0,68	0,06
Habp4	Q9JKS5	SLAASGAQQPDSPGGPQPPGQK	S(1): 0.0; S(5): 0.0; S(12): 100.0	-0,69	0,74
Pabpc1l	A2A5N3	EVFSTYGVITSAK	S(4): 0.0; T(5): 0.0; Y(6): 0.0; T(10): 100.0; S(11): 0.0	-0,69	0,15
Chgb	P16014	SSYEGHPLSEER	S(1): 0.0; S(2): 0.0; Y(3): 0.0; S(9): 100.0	-0,69	0,62
Tppp	Q7TQD2	RLSLESEGANEGATAAPELSALEEAFR	S(3): 99.6; S(6): 0.4; T(14): 0.0; S(20): 0.0	-0,69	0,16
Ppm1g	Q61074	KLEEALSTEGAEDTGNSDK	S(7): 50.0; T(8): 50.0; T(14): 0.0; S(17): 0.0	-0,69	0,59
Lrrc16a	F7AI27	TASRPEDTPDSPSGPSSPK	T(1): 0.0; S(3): 0.0; T(8): 0.0; S(11): 0.0; S(13): 0.0; S(16): 7.8; S(17): 92.2	-0,70	0,49
Unc13a	Q4KUS2	ESYSDSMHSYEEFSEPR	S(2): 0.0; Y(3): 0.0; S(4): 0.0; S(6): 0.0; S(9): 93.7; Y(10): 6.3; S(14): 0.0	-0,70	0,21
Hn1	P97825	SNSSEASSGDFLDLK	S(1): 33.3; S(3): 33.3; S(4): 33.3; S(7): 0.0; S(8): 0.0	-0,70	0,52
Smaka	Q9CPS8	TPSPGEQQQVQEVK	T(1): 0.0; S(3): 100.0	-0,70	0,17
Aak1	Q3UHJ0-2	VQTTPPPTIQGQK	T(3): 0.0; T(4): 100.0; T(8): 0.0	-0,70	0,27
Ttyh3	Q6P5F7	ESPPPSYTSSMR	S(2): 100.0; S(6): 0.0; Y(7): 0.0; T(8): 0.0; S(9): 0.0; S(10): 0.0	-0,71	0,25
Tcea1	P10711	EPAISSQNSPEAR	S(5): 5.1; S(6): 47.5; S(9): 47.5	-0,71	0,59
Map1a	Q9QYR6	SHWDDGTNDSDLEK	S(1): 0.0; T(7): 0.0; S(10): 100.0	-0,71	0,78
Map1a	Q9QYR6	VPSAPGQESPVPDTK	S(3): 0.4; S(9): 99.6; T(14): 0.0	-0,71	0,82
Npnt	D3YTX1	VTGLHSGTLQVFVRK	T(2): 50.0; S(6): 50.0; T(8): 0.1	-0,71	0,45
Pex19	Q8VCI5	AKPSPEHAPTISAPDASGPQK	S(4): 100.0; T(10): 0.0; S(12): 0.0; S(17): 0.0	-0,72	0,41
Stxbp5	D3Z2Q2	RPVSVSPSSSQEISENQYAVICSEK	S(4): 0.1; S(6): 0.5; S(8): 4.9; S(9): 47.0; S(10): 47.0; S(14): 0.5; S(23): 0.0	-0,72	0,71
Map1a	Q9QYR6	SHWDDGTNDSDLEK	S(1): 0.0; T(7): 0.0; S(10): 100.0	-0,72	0,28
Aatk	Q80YE4-4	DDCSSLEQTPR	S(4): 0.4; S(5): 99.6; T(9): 0.0	-0,72	0,09
Basp1	Q91XV3	KAEGAGTEEEGTPK	T(7): 0.0; T(12): 100.0	-0,73	0,36
Pclo	Q9QYX7	ENPELVDDLSPR	S(10): 100.0	-0,73	0,37
Ncl	P09405	GLSEDTTEETLK	S(3): 100.0; T(6): 0.0; T(7): 0.0; T(10): 0.0	-0,73	-0,30
Map1b	P14873	TPEEGGYSYEISEK	T(1): 100.0; Y(7): 0.0; S(8): 0.0; Y(9): 0.0; S(12): 0.0	-0,74	0,61
Smap	Q9R0P4	SASPDDDLGSSNWEAADLGNEER	S(1): 6.1; S(3): 93.9; S(10): 0.0; S(11): 0.0	-0,74	-0,05
Dmtn	Q9WV69	DSSVPGSPSSIVAK	S(2): 0.0; S(3): 0.0; S(7): 87.7; S(9): 6.1; S(10): 6.1	-0,74	0,13
Map1a	Q9QYR6	SPFEIISPPASPPEMTGQR	S(1): 0.0; S(7): 100.0; S(11): 0.0; T(16): 0.0	-0,74	0,02
Tmem161a	M0QWV0	YIHPSEEELR	Y(1): 50.0; S(5): 50.0	-0,74	0,46

Disease Models & Mechanisms • Supplementary information

Hnrnpa3	A2AL12	SSGSPYGGGYGSGGGSGGYGSR	S(1): 0.0; S(2): 0.1; S(4): 99.9; Y(6): 0.1; Y(10): 0.0; S(12): 0.0; S(16): 0.0; Y(19): 0.0; S(21): 0.0	-0,74	0,19
Rbbp8	Q80YR6	MDVTVIDTK	T(4): 100.0; T(8): 0.0	-0,75	0,47
Tmem161a	M0QWV0	YIHPSEEELR	Y(1): 50.0; S(5): 50.0	-0,75	0,41
D430041D05Rik	D3YZ21	GHSETSTLSSQPSIDEVR	S(3): 0.5; T(5): 97.7; S(6): 0.5; T(7): 0.5; S(9): 0.5; S(10): 0.5; S(13): 0.0	-0,76	0,71
Slc1a3	P56564	DVEMGNSVIEENEMK	S(7): 100.0	-0,76	0,34
Grin2a	P35436	QDSLNQNPVSQR	S(3): 100.0; S(10): 0.0	-0,77	0,66
Pacsin1	Q61644	KAEGATLSNATGAVESTSQAGDRGSVSSYDR	T(6): 0.0; S(8): 0.0; T(11): 0.0; S(16): 2.9; T(17): 2.9; S(18): 2.9; S(25): 87.7; S(27): 2.9; S(28): 0.6; Y(29): 0.1	-0,77	1,17
Nefm	P08553	QASHAQLGDAYDQEIR	S(3): 100.0; Y(11): 0.0	-0,77	0,32
Chgb	P16014	EGVDDQESLRPSNQQASK		-0,77	-0,26
Fam169a	Q5XG69	GHTEENLSPVSK		-0,77	0,19
Huwe1	Q7TMY8	DLSMSEEDQMMR	S(3): 91.9; S(5): 8.1	-0,77	0,74
Clns1a	Q923F1	LGEESKEPLSDEDEEDNDDVEPISEFR	S(5): 50.0; S(10): 50.0; S(24): 0.0	-0,77	0,73
Arhgap39	P59281-2	AFSEDEALAQQDSK	S(3): 100.0; S(13): 0.0	-0,78	0,66
Map2	P20357	GSAQESLDTISPKNQHDEK	S(2): 0.0; S(6): 0.3; T(9): 49.8; S(11): 49.8	-0,78	-0,09
Nefh	P19246	SPGEAKSPSEAK	S(1): 0.0; S(7): 99.6; S(9): 0.4	-0,78	0,02
Map1a	Q9QYR6	GEPVPAWEGKSPSEQEVR	S(11): 100.0	-0,79	0,40
Pcdh11x	B1AZR7	GDSPIIEEHPL	S(3): 100.0	-0,79	0,14
Rtn4	Q99P72	LSASPQEVGKPYLESFQPNLHITK	S(2): 0.0; S(4): 100.0; Y(12): 0.0; S(15): 0.0; T(23): 0.0	-0,80	0,75
Pclo	Q9QYX7	TSIGSSSSEEYKQEDSQGSGEDEDFIR	T(1): 0.0; S(2): 0.0; S(5): 0.0; S(6): 0.0; S(7): 0.0; S(8): 0.0; Y(11): 0.0; S(16): 0.7; S(19): 99.3	-0,80	0,44
Fam21	Q6PGL7	VSPEVGSADVASIAQK	S(2): 100.0; S(7): 0.0; S(12): 0.0	-0,80	0,37
Cep170b	Q80U49	QESFTKEPTSGPPAPGK	S(3): 93.6; T(5): 6.4; T(9): 0.0; S(10): 0.0	-0,81	0,27
Prrt2	E9PUL5	ASPSPSPSEAR	S(2): 99.3; S(4): 0.1; S(6): 0.7; S(8): 0.0	-0,81	0,50
Reps2	A2AFI8	APSQAAESSPTK	S(3): 0.0; S(8): 0.3; S(9): 99.7	-0,81	0,05
Marcksl1	P28667	GDVTAEEAAGASPAK	T(4): 0.0; S(12): 100.0	-0,83	0,21
Marcksl1	P28667	AAATPESQEPQAK	T(4): 50.0; S(7): 50.0	-0,83	0,60
UPF0690 protein C1orf52 homolog	Q9CWU4	LLPEGEETVESDDDKDER	T(8): 0.0; S(11): 100.0	-0,83	0,34
Zdhhc5	Q8VDZ4	GDSLKEPTSIADSSR		-0,83	0,60
Abcb5	B5X0E4	FNTLVGEK	T(3): 100.0	-0,84	0,48
Mdc1	Q5PSV9	VLLAADSEEEGDFPSGR	S(7): 100.0; S(15): 0.0	-0,84	0,30
Map1a	Q9QYR6	AELEEMEEVHPSDEEEEETKAESFYQK	S(12): 33.3; T(19): 33.3; S(23): 33.3; Y(25): 0.2	-0,84	0,27
Caskin1	Q6P9K8-2	AAASVVSGPPVASDCASPGDSAR	S(4): 0.0; S(7): 0.0; S(13): 2.5; S(17): 97.0; S(21): 0.4	-0,84	0,58
Akap5	D3YVF0	GPVAASPQK	S(6): 100.0	-0,85	0,25
Anp32a	O35381	NRTPSDVK	T(3): 100.0; S(5): 0.0	-0,85	0,50
Akap12	Q9WTQ5-2	CQETESNEEQSISPEKR	T(4): 0.0; S(6): 0.6; S(11): 0.6; S(13): 98.8	-0,85	0,52
Dbnl	Q62418-3	AMSTTSVTSSQPGK	S(3): 5.8; T(4): 87.9; T(5): 5.8; S(6): 0.4; T(8): 0.0; S(9): 0.0; S(10): 0.0	-0,85	0,51
Palmd	Q9JHU2	RSNGPTHTSPTRPTPQPR	S(2): 0.0; T(6): 0.0; T(8): 50.0; S(9): 50.0; T(11): 0.0; T(14): 0.0	-0,85	-0,07
Basp1	Q91XV3	KAEGAGTEEEGTPKESEPQAAADATEVK	T(7): 0.3; T(12): 86.6; S(16): 13.1; T(25): 0.0	-0,85	0,73
Sfr1	Q8BP27-2	ENPPSPPTSPAAPQPR	S(5): 99.6; T(8): 0.4; S(9): 0.0	-0,85	0,57
Kiaa1045	Q80TL4	RGTVEGSVQEVQEEK	T(3): 100.0; S(7): 0.0	-0,85	0,33
Irs2	P81122	ASSPAESSPEDSGYMR	S(2): 1.0; S(3): 1.0; S(7): 48.9; S(8): 48.9; S(12): 0.0; Y(14): 0.0	-0,86	0,40
Shank1	D3YZU5	GSSTEDGPGVPPPSPR	S(2): 13.5; S(3): 86.5; S(14): 0.0	-0,86	0,45
Cntnap1	O54991	DQNLPQILEESRSE	S(11): 10.3; S(13): 89.7	-0,86	0,43
Tmf1	B9EKI3	SVSEINSDDELPGK	S(1): 0.0; S(3): 0.1; S(7): 99.9	-0,86	0,17
Ppp6r3	Q922D4-2	IQQFDDGGSDEEDIWEEK	S(9): 100.0	-0,87	0,90
Pacsin1	Q61644	KAEGATLSNATGAVESTSQAGDRGSVSSYDR	T(6): 0.0; S(8): 0.0; T(11): 0.1; S(16): 78.2; T(17): 16.7; S(18): 3.8; S(25): 0.9; S(27): 0.2; S(28): 0.1; Y(29): 0.1	-0,87	0,22
Ppp1r9b	Q6R891	ASSLNENVDHSALLK	S(2): 0.4; S(3): 99.6; S(11): 0.0	-0,87	0,73
Npy	P57774	SSPETLISDLLMKESTENAPR	S(1): 0.0; S(2): 0.0; T(5): 0.0; S(8): 0.0; S(15): 99.5; T(16): 0.5	-0,88	0,76
Arfgef2	A2A5R2	QSLSSADNLEPDVQGHQVAAR	S(2): 8.7; S(4): 8.7; S(5): 82.5	-0,88	0,03

Disease Models & Mechanisms • Supplementary information

Dbnl	Q62418-3	QLTQPETSYGR	T(3): 100.0; T(7): 0.0; S(8): 0.0; Y(9): 0.0	-0,88	0,55
Fbxl20	Q9CZV8-4	VHAYFAPVTPPPSVGGSR	S(13): 99.1; S(17): 0.9	-0,88	0,66
Efhd2	Q8C845	ADLNQGIGEPQSPSR	S(12): 95.8; S(14): 4.2	-0,89	0,85
Pak3	Q61036-2	IGQGASGTVYTALDIATGQEVAIK	S(6): 0.0; T(8): 0.0; Y(10): 0.0; T(11): 0.0; T(17): 100.0	-0,90	0,29
Ywhaz	P63101	TAFDEAIAELDTLSEESYK	T(1): 0.0; T(12): 0.0; S(14): 100.0; S(17): 0.0; Y(18): 0.0	-0,90	0,12
Map1a	Q9QYR6	APISLSQDPSPPLNGSTTSCGPDR	S(4): 0.0; S(6): 0.0; S(10): 100.0; S(16): 0.0; T(17): 0.0; T(18): 0.0; S(19): 0.0	-0,90	0,49
Zswim8	Q3UHH1-5	RTFANGSADEFQR	T(2): 91.1; S(7): 8.9	-0,90	0,23
Wfs1	Q3UN10	LNATASLEQDK	T(4): 6.4; S(6): 93.6	-0,90	0,18
Rnf20	Q5DTM8	ALVVPEPEPDSDSNQER	S(11): 6.2; S(13): 93.8	-0,91	0,04
Fasn	P19096	TDSATDTTAPK	T(1): 0.1; S(3): 91.6; T(5): 7.5; T(7): 0.7; T(8): 0.1	-0,92	0,52
Marcks	P26645	GEATAERPGEAAVASSPSK	T(4): 0.0; S(15): 0.8; S(16): 98.3; S(18): 0.8	-0,92	0,45
Tom1	O88746	GLEFPMTDLDMLSPIHTPQR	T(7): 0.0; S(13): 100.0; T(17): 0.0	-0,92	0,34
Atp2b1	G5E829	IEDSEPHIPLIDDTDAEDDAPTK	S(4): 100.0; T(14): 0.0; T(22): 0.0	-0,93	0,26
Bcmo1	E9Q321	YLQSDTYIANIEANR	Y(1): 0.0; S(4): 0.1; T(6): 7.2; Y(7): 92.8	-0,93	0,73
Sh3bp5l	Q99LH9	CDSVEHLR	S(3): 100.0	-0,93	0,24
Ranbp3	Q9CT10	SAGSSSPEAGEDSDHEDGNYCPPVK	S(1): 0.0; S(4): 0.0; S(5): 0.0; S(6): 0.0; S(13): 100.0; Y(20): 0.0	-0,93	0,46
Dnah7b	L7N1Y0	SSDEVVNEVAGDILSK	S(1): 7.5; S(2): 92.5; S(15): 0.0	-0,94	-0,22
Bsn	O88737	DYLSDSELNQLR	Y(2): 0.0; S(4): 99.9; S(6): 0.0	-0,94	0,54
Slc7a14	Q8BXR1	ETCSPVSEGEEFSSPATNTCGAK	T(2): 33.3; S(4): 33.3; S(7): 33.3; S(13): 0.0; S(14): 0.0; T(17): 0.0; T(19): 0.0	-0,94	0,98
Arhgap35	Q91YM2	NEEENIYSVPHDSTQGK	Y(7): 99.5; S(8): 0.5; S(13): 0.0; T(14): 0.0	-0,94	0,21
Sparcl1	P70663	AEKPSALNSEEETHEQSTEQDK	S(5): 33.1; S(9): 33.1; T(13): 33.1; S(17): 0.3; T(18): 0.3	-0,95	0,56
Bsn	O88737	DACEPESGPDPSTVR	S(7): 100.0; S(12): 0.0; T(13): 0.0	-0,95	0,44
Clip2	Q9Z0H8	QASGPSSSGAATTVSEKPGPK	S(3): 82.9; S(6): 8.5; S(7): 8.5; S(8): 0.1; T(12): 0.0; T(13): 0.0; S(15): 0.0	-0,95	0,81
Mical3	Q8CJ19	GTSQVSSPSQPPEK	T(2): 0.0; S(3): 0.0; S(6): 0.4; S(7): 93.4; S(9): 6.2	-0,95	0,12
Map4	P27546	DMSPSAETEAPLAK	S(3): 50.0; S(5): 50.0; T(8): 0.0	-0,95	0,11
Park2	Q3UVA0	NLTETLVGGSGGR	T(3): 1.4; T(5): 97.3; S(10): 1.4	-0,96	0,38
Caskin1	Q6P9K8-2	RGSGEPAEQSHFMEDGTAR	S(3): 100.0; S(10): 0.0; T(17): 0.0	-0,96	0,17
Ptges3	Q9R0Q7	DWEDDSDEDMSNFDR	S(6): 100.0; S(11): 0.0	-0,98	0,55
Map1a	Q9QYR6	SEPQDFQEDSWGDTK	S(1): 0.0; S(10): 5.4; T(14): 94.6	-0,98	0,56
Lmtk2	Q3TYD6	GQSPPESVVPEESSDVR	S(3): 100.0; S(7): 0.0; S(13): 0.0; S(14): 0.0	-0,99	0,32
Map1a	Q9QYR6	TEATQGLDYVPSAGTISPTSSLEEDKGFK	T(1): 0.0; T(4): 0.0; Y(9): 0.0; S(12): 0.0; T(15): 0.2; S(17): 24.9; T(19): 24.9; S(20): 24.9; S(21): 24.9	-0,99	0,89
Map1b	P14873	ASLSPMDEPVPDSESPVEK	S(2): 0.0; S(4): 0.0; S(13): 0.0; S(15): 100.0	-1,00	0,29
Apba1	B2RUJ5	MDSYEQEEDIDQIVAEVK	S(3): 50.0; Y(4): 50.0	-1,01	1,13
Arpp21	E9Q4A0	TGSESSSSAGSSGSLSR	T(1): 1.0; S(3): 97.9; S(5): 1.0; S(6): 0.1; S(7): 0.0; S(8): 0.0; S(11): 0.0; S(12): 0.0; S(14): 0.0; S(16): 0.0	-1,02	1,03
Pp2d1	Q8BVT6	ECIISNLLR	S(5): 100.0	-1,02	0,91
Myo18a	B2RRE2	EPADLDPEAASPAYSQAK	S(11): 100.0; Y(14): 0.0; S(15): 0.0	-1,03	0,39
Chgb	P16014	ADEFPDFYDSEEQMGPHQEANDEK	Y(8): 1.0; S(10): 99.0	-1,03	0,47
Basp1	Q91XV3	ETPAASEAPSSAAK	T(2): 0.0; S(6): 0.0; S(10): 5.8; S(11): 94.2	-1,05	0,68
Syn1	O88935	QASISGPAPTK	S(3): 100.0; S(5): 0.0; T(10): 0.0	-1,05	0,85
Arpp21	E9Q4A0	SESLDEEEKLELQR	S(1): 0.0; S(3): 100.0	-1,05	0,68
Chgb	P16014	WWQQEEQLGPEESREEVR	S(13): 100.0	-1,06	0,48
Me1	P06801	LFYSVLMSDVEK	Y(3): 47.9; S(4): 47.9; S(8): 4.3	-1,09	0,45
Bsn	O88737	EPELEMESLTGSPEDR	S(8): 0.0; T(10): 93.4; S(12): 6.5	-1,09	0,05
Rapgef2	E9QNQ4	SLGSLSQGSANATVLDVAQTGGHK	S(1): 91.1; S(4): 8.9; S(6): 0.0; S(9): 0.0	-1,10	1,18
Tmem44	E9Q4M0	RASLSPVDLASDD	S(3): 96.4; S(5): 3.6; S(11): 0.0	-1,11	0,89
Pan3	Q640Q5	SVLVVTYSDLK	S(1): 0.0; S(8): 100.0	-1,12	0,60
Stxbp1	O08599-2	ESSRVSFEDQAPTME	S(2): 0.0; S(3): 0.0; S(6): 100.0; T(13): 0.0	-1,12	0,78
Srr	Q9QZX7-2	GLIPDTPEEKPK	T(6): 100.0	-1,15	0,00

Disease Models & Mechanisms • Supplementary information

Chgb	P16014	ESKEADVATVR	S(2): 100.0; T(9): 0.0	-1,18	0,45
Cplx1	P63040	EAEAQAAMEANSEGSLTRPK	S(12): 0.8; S(15): 98.3; T(17): 0.8	-1,21	0,79
Npy	P57774	SSPETLISDLLMKESTENAPR	S(1): 0.0; S(2): 0.0; T(5): 0.0; S(8): 0.0; S(15): 99.3; T(16): 0.7	-1,21	1,10
Tnrc6c	Q3UHC0	DSSEATGWEEPSPPSIR	S(2): 0.0; S(3): 0.0; T(6): 0.0; S(12): 99.9; S(15): 0.1	-1,23	0,82
Mccc2	Q3ULD5	AYHGDSVARLGTQPDSASSTYQENYEQMK	S(6): 0.0; S(16): 9.7; S(18): 45.1; S(19): 45.1	-1,27	0,70
Scg2	Q03517	RVPSPVSSEDDLQEEEQLEQAIK	S(4): 0.0; S(7): 99.5; S(8): 0.5	-1,28	0,62
Prkcb	P68404-2	NIDQSEFEGFSFVNSEFLKPEVK	S(5): 0.5; S(11): 49.8; S(15): 49.8	-1,31	0,23
Dgkg	Q91WG7	EIEQSPLVMLDR	S(5): 100.0	-1,35	0,08
Cttn	Q60598	QTPPASPSPQPIEDRPPSSPIYEDAAPFK	T(2): 0.0; S(6): 0.0; S(8): 0.0; S(18): 88.9; S(19): 9.9; Y(22): 1.2	-1,38	0,98
Col3a1	P08121	GFPGNPGPPGSPGAAGHQGAIGSPGPAGPR	S(11): 50.0; S(23): 50.0	-1,52	0,06
Arhgef11	Q68FM7	TCEGRLSVDSQEADSGLDSGTER	T(1): 0.6; S(7): 96.5; S(10): 3.0; S(15): 0.0; S(19): 0.0; T(21): 0.0	-1,52	0,50
Hsp90aa1	P07901	ESDDKPEIEDVGSDEEEEEK	S(2): 0.0; S(13): 100.0	-1,53	0,99
Map2	P20357	LASVSADAEVAR	S(3): 100.0; S(5): 0.0	-1,56	0,77
Trim28	Q62318	RPAASSAAAASAAASSPAGGGGEAQELLEHCGVCR	S(5): 0.0; S(6): 0.0; S(11): 0.0; S(15): 50.0; S(16): 50.0	-1,66	0,54
Slc12a5	Q91V14	ETDPEVHLTWTK	T(2): 0.0; T(9): 99.6; T(11): 0.4	-1,67	1,01
Smarcc2	Q3UID0	DMDEPSPVPNVEEVTLPK	S(6): 100.0; T(15): 0.0	-1,79	2,05
Camkv	Q3UHL1	SATPATDGRATPATEESTVPATQSSALPAAK	S(1): 0.0; T(3): 0.0; T(6): 0.0; T(11): 98.3; T(14): 1.7; S(17): 0.0; T(18): 0.0; T(22): 0.0; S(24): 0.0; S(25): 0.0	-1,81	1,54
Tsc22d2	E9Q7M2	TEDVSSEIFDVSR	T(1): 0.0; S(5): 50.0; S(6): 50.0; S(12): 0.0	-1,91	1,05
Camkv	Q3UHL1	SATPATDGRATPATEESTVPATQSSALPAAK	S(1): 2.4; T(3): 2.4; T(6): 2.4; T(11): 44.8; T(14): 44.8; S(17): 2.4; T(18): 0.6; T(22): 0.0; S(24): 0.0; S(25): 0.0	-1,97	1,17
Camkv	Q3UHL1	ATPATEESTVPATQSSALPAAK	T(2): 98.0; T(5): 1.9; S(8): 0.1; T(9): 0.1; T(13): 0.0; S(15): 0.0; S(16): 0.0	-2,18	1,91

Disease Models & Mechanisms • Supplementary information

Supplementary Table S5. Proteins up and down phosphorylated in the cortex of Ts65Dn mice and reverted by L41 treatment.

Gene name	Protein Group Accessions	Sequence	phosphoRS Site Probabilities	Ts65Dn vs WT	Ts65Dn +/-L41
Lppr4	Q7TME0	QTYELNDLNR	T(2): 50.0; Y(3): 50.0	5,46	-6,56
Khdrbs1	Q60604-2	ASQVAIGIRDNER	S(2): 100.0	4,82	-3,08
Napa	Q9DAV8	NYQFLAIFNLSHCLR	Y(2): 100.0; S(11): 0.0	3,44	-3,06
Akap12	Q9WTP7	VYNIEFNPPK	Y(2): 100.0	2,88	-1,42
Arhgap39	P59281-2	EDTSSCSPPGVLLEK	T(3): 33.3; S(4): 33.3; S(5): 33.3; S(7): 0.0	2,52	-2,93
Kif5b	Q61738-4	GANVQKESK	S(8): 100.0	2,20	-2,11
Sipa1l1	Q8C0T5	QDPVVHLSPNK	S(8): 100.0	2,16	-2,31
Dhx16	Q04690-2	SSSGLATYSPPMGAVSER	S(1): 0.0; S(2): 0.0; S(3): 0.0; T(7): 0.0; Y(8): 6.8; S(9): 93.2; S(16): 0.0	2,15	-1,77
Mark1	Q8VHG2	DTTVISHSPNTSYDTALEAR	T(2): 0.0; T(3): 0.0; S(6): 0.0; S(8): 100.0; T(11): 0.0; S(12): 0.0; Y(13): 0.0; T(15): 0.0	1,65	-1,70
Asap1	H3BL41	GVFPVSFVHILSD	S(6): 0.0; S(12): 100.0	1,58	-1,07
Pgm3	Q9CXF4	DDSPTQTLASPNACR	S(3): 99.6; T(5): 0.4; T(7): 0.0; S(10): 0.0	1,53	-1,40
Osbp	Q2WF71	STPHLDGAGGGAAGEDGDLGLGSAR	S(1): 50.0; T(2): 50.0; S(23): 0.0	1,51	-1,52
Bsn	O88737	GEHSSTLPASTPSYTSGTSPTSLSSLEEDSDSSPSR	S(4): 0.0; S(5): 0.0; T(6): 0.0; S(10): 0.0; T(11): 0.0; S(13): 0.0; Y(14): 0.0; T(15): 0.0; S(16): 0.0; T(18): 0.1; S(19): 0.1; T(21): 0.3; S(22): 0.8; S(24): 0.8; S(25): 0.8; S(30): 15.7; S(32): 15.7; S(33): 15.7; S(35): 49.9	1,51	-1,24
Tsc1	Q9DCS2	ISPQSNVDFDLTLR	S(2): 90.5; S(5): 9.5; T(12): 0.0	1,39	-1,79
Ehd3	Q9QXY6	DKPMYDEIFYTLSPVDGK	Y(5): 0.0; Y(10): 0.1; T(11): 8.1; S(13): 91.9	1,38	-1,10
Agap2	Q3UHD9	AISAFGPSASINGLVK	S(3): 0.0; S(8): 50.0; S(10): 50.0	1,38	-0,04
Sin3a	Q5XJV6	AQRPPEGMSPELPPR	S(9): 100.0	1,38	-1,52
Phkb	Q7TQH0-2	EVDGLLTSDPMGSPVSSK	T(7): 0.0; S(8): 0.0; S(13): 99.8; S(16): 0.2; S(17): 0.0	1,35	-0,93
Sgip1	Q8VCY8-2	LSPWEDLSQAPTMDSPLEK	S(2): 0.0; S(8): 0.0; T(12): 0.4; S(15): 99.6	1,34	-1,54
Ptpn23	Q6PAR5-6	RPGNEELPPAAATGATSLVAAPHSSSSSPSK	T(13): 0.0; T(16): 0.0; S(17): 0.1; S(24): 26.5; S(25): 6.8; S(26): 6.8; S(27): 26.5; S(28): 26.5; S(30): 6.8	1,33	-0,26
Lamtor1	Q99PV8-2	ELAGNSSTPPPVSPGR	S(6): 0.1; S(7): 0.0; T(8): 0.0; S(13): 99.9	1,29	-0,63
Map1b	P14873	TPEEGGYSYEISEK	T(1): 100.0; Y(7): 0.0; S(8): 0.0; Y(9): 0.0; S(12): 0.0	1,27	-1,03
Grin2b	G3X9V4	RQHSYDTFVDLQK	S(4): 99.6; Y(5): 0.4; T(7): 0.0	1,26	-1,39
Rab11fip2	G3XA57	SGTNDTYTIIQLGK	S(1): 0.1; T(3): 8.3; T(6): 83.2; Y(7): 8.3; T(8): 0.1	1,18	-0,90
Arhgap21	B7ZCJ1	DQGEPVSPSEDEPFSWPGPK	S(7): 91.8; S(9): 8.2; S(15): 0.0	1,16	-1,15
Bod1l	E9Q6J5	GSDDVLVSGEVPECEVGHMSPR	S(2): 0.0; S(8): 0.0; S(20): 100.0	1,15	-0,65
Gsk3b	Q9WV34	TYETPPPSPGLDPTFSNQPVPPDAVR	T(1): 49.8; Y(2): 49.8; T(4): 0.5; S(8): 0.0; T(14): 0.0; S(16): 0.0	1,10	0,00
Sec22b	O08547	NLGSINTELQDVQR	S(4): 93.0; T(7): 7.0	1,10	-0,76
Ppp1r1b	Q60829	RPNPCAYTPPSLK	Y(7): 0.0; T(8): 100.0; S(11): 0.0	1,04	-1,14
Usp45	E9Q4D8	ISLSSAPQLEPLVVELSSPGPLTSALFL	S(2): 69.4; S(4): 15.3; S(5): 15.3; S(17): 0.0; S(18): 0.0; T(23): 0.0; S(24): 0.0	1,04	0,07
Palmd	Q9JHU2	SNGPTHTSPTRPTPQPR	S(1): 0.0; T(5): 0.6; T(7): 0.6; S(8): 98.7; T(10): 0.0; T(13): 0.0	1,00	-1,26
Rps3	P62908	DEILPTTPISEQK	T(6): 4.8; T(7): 95.2; S(10): 0.0	0,98	-0,57
Exo1	Q9QZ06	LGYAVYETPTAHNGAK	Y(3): 0.0; Y(6): 99.3; T(8): 0.7; T(10): 0.0	0,98	-1,17
Avl9	Q80U56	DSLPSDSPPITVQPQANNR	S(2): 0.0; S(5): 0.7; S(7): 99.3; T(11): 0.0	0,97	-0,44
Chgb	P16014	EGVDDQESLRPSNQQASK	S(8): 0.3; S(12): 11.8; S(17): 88.0	0,97	-0,53
Kcna2	P63141	ETEGEEQAQYLQVTSCPK	T(2): 0.0; Y(10): 0.0; T(14): 0.0; S(15): 100.0	0,97	-0,48
Psd	Q5DP50	MGSQSLNSTFLK	S(3): 0.1; S(5): 7.9; S(8): 91.2; T(9): 0.8	0,96	-0,90
Abcc1	O35379	GSSQLDVNEEVEALIVK	S(2): 50.0; S(3): 50.0	0,94	-0,26
Setd1a	E9PYH6	SGSPAPETTNESVPFAQHSSLDSR	S(1): 0.6; S(3): 99.3; T(8): 0.0; T(9): 0.0; S(12): 0.0; S(19): 0.0; S(20): 0.0; S(23): 0.0	0,91	-0,79
Nup210l	Q9D219	SSTPSHGQTPAPEPISAQK	S(1): 1.2; S(2): 1.2; T(3): 95.4; S(5): 1.2; T(9): 1.2; S(16): 0.0	0,91	-0,45
Ppp1r1b	Q60823	YFDDEFTAQSITITPPDR	Y(1): 0.0; T(7): 0.0; S(10): 0.0; T(12): 0.2; T(14): 99.8	0,90	-0,06
Begain	F8WIG2	RPSVDTPVTDVGFLR	S(3): 6.4; T(6): 93.6; T(9): 0.0	0,88	-0,15

Disease Models & Mechanisms • Supplementary information

Pde1b	Q01065	SPPEMLESDCPSPLELK	S(1): 100.0; S(8): 0.0; S(12): 0.0	0,87	-0,53
Uncharacterized protein C15orf39 homolog	Q3TEI4	GAAYQAGGLGSPYLR	Y(4): 83.6; S(11): 13.8; Y(13): 2.6	0,86	-1,65
Ywhaz	P63101	TAFDEAIAELDTLSEESYK	T(1): 0.0; T(12): 0.0; S(14): 100.0; S(17): 0.0; Y(18): 0.0	0,86	-1,02
Ctps1	P70698	SGSSSPDSEITELK	S(1): 0.0; S(3): 0.0; S(4): 0.0; S(5): 0.2; S(8): 99.8; T(11): 0.0	0,85	-0,11
Wrnip1	Q91XQ0-2	NSDILSSTEGK	S(2): 0.0; S(6): 9.1; S(7): 1.0; T(8): 89.8	0,85	-0,84
Lpin2	Q99P72	ESAEFSVLEYSEMGSSFNGSPK	S(2): 0.0; S(6): 0.0; Y(10): 0.0; S(11): 0.0; S(15): 0.0; S(16): 6.7; S(20): 93.3	0,85	-1,05
Camk2d	E9Q1T1	ENFSGGTSLWQNI	S(4): 88.3; T(7): 5.8; S(8): 5.8	0,84	-0,14
Prkab2	Q6PAM0	DLSSSPPGPYGQEMYVFR	S(3): 0.0; S(4): 0.0; S(5): 100.0; Y(10): 0.0; Y(15): 0.0	0,83	-0,92
Tbc1d15	Q9CXF4	DDSPTQTLASPNACR	S(3): 92.4; T(5): 7.5; T(7): 0.1; S(10): 0.0	0,82	-0,79
Eml2	E9QK48	VGGYATSPSSPK	Y(4): 0.0; T(6): 0.3; S(7): 33.2; S(9): 33.2; S(10): 33.2	0,81	-0,76
Epn1	Q80V94	IIEQPECSSPVIETER	S(8): 50.0; S(9): 50.0; T(14): 0.0	0,81	-0,44
Map1a	Q9QYR6-2	VAELEEEQSQGSSSYSDWVK	S(9): 100.0; S(12): 0.0; S(13): 0.0; S(14): 0.0; Y(15): 0.0; S(16): 0.0	0,81	-0,36
Usp10	P52479-2	TCDSPQNPVDFISGPVPDSPFPR	T(1): 11.1; S(4): 88.9; S(13): 0.0; S(19): 0.0	0,80	0,04
Kcnd1	Q03717	STSSLPPEPMEIVR	S(1): 0.0; T(2): 0.2; S(3): 9.9; S(4): 90.0	0,74	-0,34
Gng3	P63216	GETPVNSTMSIGQAR	T(3): 0.0; S(7): 0.1; T(8): 0.1; S(10): 99.9	0,71	-0,83
Tppp	Q7TQ95	ADSVPNLEPSEESLVTK	S(3): 100.0; S(10): 0.0; S(13): 0.0; T(16): 0.0	0,71	-0,42
Lmtk3	Q5XJV6	ALVNGEPMSPEAGEK	S(9): 100.0	0,70	-0,64
Usp5	Q3U3J1	IGHHSTSDDSSAYR	S(5): 100.0; T(6): 0.0; S(7): 0.0; S(10): 0.0; S(11): 0.0; Y(13): 0.0	0,70	-0,39
Arfgef1	G3X9K3	VETEKQSPPHGEAK	T(3): 0.4; S(7): 99.6	0,69	-0,43
Hnrnpc	Q9Z1P7	SEGALQVLDPGSRTPDGEPR	S(1): 0.0; S(12): 99.4; T(14): 0.6	0,69	-0,64
Comt	O88587-2	AVYQGPGSSPVK	Y(3): 0.0; S(8): 50.0; S(9): 50.0	0,68	0,24
Arhgef2	H3BJU7	SVSTTNIAGHFNDESPLGLR	S(1): 0.0; S(3): 0.0; T(4): 0.0; T(5): 0.0; S(15): 100.0	0,68	-0,85
Grin2a	P35436	GSLIVDMVSDK	S(2): 100.0; S(9): 0.0	0,68	-0,80
Gm15800	E9Q2E4	SMSAPSDLEMIGNEDLEFTR	S(1): 47.3; S(3): 47.3; S(6): 5.4; T(19): 0.0	0,67	-0,87
Klc2	D3YXZ3	VDSPTVNTTLR	S(3): 100.0; T(5): 0.0; T(8): 0.0; T(9): 0.0	0,67	-0,40
Yap1	P46938-2	GDSETDLEALFNAVMNPK	S(3): 50.0; T(5): 50.0	0,67	-0,41
Gripap1	A2AEW8	TGLEELVLSEMNSPSR	T(1): 0.0; S(9): 0.0; S(13): 50.0; S(15): 50.0	0,66	-0,98
Kif17	A2AM72	EYQEEIK	Y(2): 100.0	0,65	-0,51
Apc	B2RUG9	SPTGNTPPVIDSVSEK	S(1): 99.9; T(3): 0.1; T(6): 0.0; S(12): 0.0; S(14): 0.0	0,64	-0,29
Pkdrej	F8VQF3	YSRFFYNVRLAQK	Y(1): 100.0; S(2): 0.0; Y(6): 0.0	0,64	-0,53
Aldoc	P05063	GILAADESVGSMAK	S(8): 100.0; S(11): 0.0	0,64	-0,48
Rtn1	Q8K0T0	GSVSEDELIAAIK	S(2): 3.4; S(4): 96.6	0,64	-0,72
Add2	Q9QY81	ELASPLALTPR	S(4): 0.8; T(9): 99.2	0,64	0,36
Pdpk1	F2Z3X6	ANSFVGTAQYVSPELLTEK	S(3): 99.7; T(7): 0.3; Y(10): 0.0; S(12): 0.0; T(17): 0.0	0,64	-0,93
Slc43a2	Q8CFT2-2	ISSPSSSSSSDK	S(2): 48.9; S(3): 48.9; S(5): 1.3; S(6): 0.1; S(7): 0.1; S(8): 0.3; S(9): 0.3; S(10): 0.3	0,63	-0,43
Chga	P26339	GELEHSQQEEDGEEAMVGTPQGLFPQGGK	S(6): 100.0; T(19): 0.0	0,62	-0,36
Map1b	P14873	TTRSPDTSAYCYETMEK	T(1): 0.0; T(2): 6.8; S(4): 93.1; T(7): 0.0; S(8): 0.0; Y(10): 0.0; Y(12): 0.0; T(14): 0.0	0,61	-0,65
Map1a	Q9QYR6-2	ELSSAVSPPNLHSDTPTFSYASLAGPTIPPR	S(3): 0.0; S(4): 0.8; S(7): 99.2; S(13): 0.0; T(15): 0.0; T(17): 0.0; S(19): 0.0; Y(20): 0.0; S(22): 0.0; T(27): 0.0	0,61	-0,29
Arhgef7	Q9ES00	LAGGQTSQPTTPLTSPQR	T(6): 0.0; S(7): 0.0; T(10): 0.0; T(11): 0.0; T(14): 9.5; S(15): 90.5	0,61	0,22
Xrn2	Q9DB05	AIDIYEQVGTSAMDSPLLK	Y(5): 0.0; T(10): 0.0; S(11): 0.0; S(15): 100.0	0,60	-1,65
Atp4a	Q91WG5	SSSKESSPNSNPSTSPGGIR	S(1): 0.4; S(2): 49.3; S(3): 49.3; S(6): 0.4; S(7): 0.4; S(10): 0.0; S(13): 0.0; T(14): 0.0; S(15): 0.0	0,59	-0,98
Yap1	P46938-2	QSSFEIPDDVPLPAGWEMAK	S(2): 0.5; S(3): 99.5	0,59	0,30
Kif2a	E0CZ72	ARPSQLPEQSSSAQQNGSVSDISPVQAAK	S(4): 0.0; S(10): 0.0; S(11): 0.0; S(12): 0.0; S(18): 0.0; S(20): 0.0; S(23): 100.0	0,59	-0,28
Cacna1e	Q61206	ELFSPLHALNFGIGGDTTR	S(4): 100.0; T(17): 0.0; T(18): 0.0	0,59	-0,54
Gprin1	Q3UNH4	AETVSPGEVDAMTLGK	T(3): 7.0; S(5): 93.0; T(13): 0.0	0,58	-0,62

Gbf1	Q69ZX8	TSETSISPPGSSIGSPNR	T(1): 0.0; S(2): 0.0; T(4): 0.0; S(5): 0.0; S(7): 100.0; S(11): 0.0; S(12): 0.0; S(15): 0.0	0,57	-0,14
Ap2a1	P17426-2	RDTSSNDINGGVEPTPSTVSTPSPSADLLGLR	T(3): 0.0; S(4): 0.0; S(5): 0.0; T(15): 0.0; S(17): 0.0; T(18): 0.0; S(20): 0.6; T(21): 0.6; S(23): 98.3; S(25): 0.6	0,56	0,15
Otud7a	Q8R554	AAPGTGGPTPGRSPPAPAR	T(5): 0.0; T(9): 0.0; S(13): 100.0	0,55	0,00
Pclo	Q9QYX7	KIVDSGVQTDDEETADR	S(5): 0.1; T(9): 99.9; T(14): 0.0	0,55	0,03
Ankrd63	A2ARS0	GSNSDSPPGHPAPAPSPER	S(2): 33.3; S(4): 33.3; S(6): 33.3; S(16): 0.0	0,55	-0,35
Ppp1r9b	Q6R5C6	NQALEMLSR	S(8): 100.0	0,55	0,44
Prkcb	P68404-2	NIDQSEFEGFSFVNSEFLKPEVK	S(5): 0.0; S(11): 50.0; S(15): 50.0	0,54	-0,23
Rims1	Q99NE5-5	SASTNCLRPDTSLHSPER	S(1): 0.0; S(3): 0.0; T(4): 0.0; T(11): 0.0; S(12): 0.0; S(15): 100.0	0,53	-0,47
Bora	Q8BRF7	VNLEESTGVENSPAGARPK	S(6): 0.0; T(7): 0.0; S(12): 100.0	0,53	-0,27
Aak1	Q3UHJ0-2	REQGSSGLGSGSSGGGGSSSGLGSGYIGR	S(5): 0.0; S(6): 0.0; S(10): 0.1; S(12): 13.4; S(13): 86.5; S(18): 0.0; S(19): 0.0; S(20): 0.0; S(24): 0.0; Y(26): 0.0	0,52	-0,20
Sfr1	Q8BP27-2	ENPPSPPTSPAAPQPR	S(5): 100.0; T(8): 0.0; S(9): 0.0	0,52	-0,60
Phyhipl	Q8BGR1	HRTVLAEEFK	T(3): 100.0	0,52	-0,20
Rtn4	Q99P31	LLQTCFSSPTDDSMDR	T(4): 0.0; S(7): 49.9; S(8): 49.9; T(10): 0.2; S(13): 0.0	0,52	-0,29
Ankrd34a	B2RW11	APSLPAPPPSGAPGSPR	S(3): 0.0; S(10): 0.0; S(15): 100.0	0,51	-0,57
Gm15800	E9Q2E4	TEGTPPPPGQPAK	T(1): 0.0; T(4): 100.0	0,51	-0,58
Map1b	P14873	DYNASASTISPPSSMEEDK	Y(2): 0.0; S(5): 0.0; S(7): 0.2; T(8): 1.2; S(10): 87.6; S(13): 9.9; S(14): 1.2	0,51	0,03
Dchs1	F6TP58	ITFNLLAGGDGLFTVDPTTGHVR	T(2): 0.1; T(14): 43.8; T(18): 12.3; T(19): 43.8	0,51	-0,19
Foxk1	P42128	SAPASPTHPGLMSPR	S(1): 0.0; S(5): 100.0; T(7): 0.0; S(13): 0.0	0,51	-0,20
Trappc8	E9PWG2	NSDSNLLSLDGLDNEVK	S(2): 50.0; S(4): 50.0; S(8): 0.1	0,50	-0,58
Dbr1	Q922Y1	SSPPATDPGPVPSSPSQEPPTK	S(1): 47.0; S(2): 47.0; T(6): 6.0; S(13): 0.0; S(14): 0.0; S(16): 0.0; T(21): 0.0	0,50	0,39
Ncoa7	Q6A0A2	SPSPVHLPEDPK	S(1): 0.0; S(3): 100.0	0,50	-0,59
Syn1	O88935	LPSPTAAPQQSASQATPVTQGQGR	S(3): 99.4; T(5): 0.6; S(11): 0.0; S(13): 0.0; T(16): 0.0; T(19): 0.0	0,50	-0,13
Rgs7bp	Q8BQC3-2	YTLLPK	Y(1): 100.0; T(2): 0.0	0,50	-0,46
Wdr7	Q920I9	TYQVPPVQPASPGSHNALK	T(1): 0.0; Y(2): 0.0; S(11): 99.4; S(14): 0.6	0,49	-0,32
Cplx1	P63040	EAEAQAAMEANSEGSLTRPK	S(12): 1.7; S(15): 86.8; T(17): 11.5	0,49	-0,40
Specc1	Q5SXY1	LGSSPTSSCNPTPTK	S(3): 7.3; S(4): 7.3; T(6): 0.8; S(7): 75.9; S(8): 7.3; T(12): 0.8; T(14): 0.8	0,49	-0,05
Reps1	E9Q632	RQSSSYEDPWK	S(3): 98.9; S(4): 0.4; S(5): 0.4; Y(6): 0.4	0,49	-0,15
Rtn4	Q99P72	RGSGSVDETLFALPAASEPVIPSSAEK	S(3): 98.0; S(5): 1.0; T(9): 1.0; S(17): 0.0; S(23): 0.0; S(24): 0.0	0,48	0,26
Aak1	Q3UHJ0-2	EQGSSGLGSGSSGGGGSSSGLGSGYIGR	S(4): 0.0; S(5): 0.0; S(9): 0.1; S(11): 9.9; S(12): 90.0; S(17): 0.0; S(18): 0.0; S(19): 0.0; S(23): 0.0; Y(25): 0.0	0,48	-0,40
Apc	B2RUG9	QSVGSGSPVQTVGLETR	S(2): 0.0; S(5): 0.4; S(7): 99.5; T(11): 0.0; T(16): 0.0	0,48	-0,62
Rims1	Q99NE5-5	RASQSSLESSSGPPCIR	S(3): 100.0; S(5): 0.0; S(6): 0.0; S(9): 0.0; S(10): 0.0; S(11): 0.0	0,48	0,13
Tbc1d5	Q80XQ2	EFTGSPPPSATK	T(3): 6.6; S(5): 93.3; S(9): 0.0; T(11): 0.0	0,48	-0,33
Tiam1	G3UWG2	ANSLGDLYAQK	S(3): 100.0; Y(8): 0.0	0,48	0,31
Synj1	E9Q7S0	TPGPPSSQGSPVDTQPAAQK	T(1): 0.0; S(6): 1.3; S(7): 10.2; S(10): 87.2; T(14): 1.3	0,47	-0,19
Krt15	Q61361	GAIYSIPISEDGGGGSSTPEDPAEAPR	Y(4): 0.0; S(5): 0.0; S(9): 0.0; S(16): 7.7; S(17): 91.6; T(18): 0.7	0,47	-0,17
Prl8a1	Q9DA80	VSSQPAPSQER	S(2): 50.0; S(3): 50.0; S(8): 0.0	0,47	-0,50
Mff	Q6PB44-2	GAAAADLLSSSPESQHGGTQPPGGGQPLLQPTK	S(9): 0.1; S(10): 25.0; S(11): 25.0; S(14): 25.0; T(19): 25.0; T(32): 0.0	0,47	0,03
Ubqln2	Q9QZD4	QTSSSPEGCGVEIK	T(2): 0.0; S(3): 0.0; S(4): 5.1; S(5): 94.9	0,46	0,03
Ssuh2	Q8C1E1	AGSLNLEITGQLNSK	S(3): 0.0; T(9): 13.7; S(14): 86.3	0,46	-0,15
Trank1	Q8BTI8	SGTPPRPGSVTNMQADECTATPQR	S(1): 0.0; T(3): 100.0; S(9): 0.0; T(11): 0.0; T(19): 0.0; T(21): 0.0	0,45	-0,13
Tacc1	F8VQ95	APVSVACGGESPLDGICLSEADK	S(4): 0.0; S(11): 100.0; S(19): 0.0	0,45	0,21
Gpr162	Q3UMB5	VASPANVGTLHTLSR	S(3): 100.0; T(9): 0.0; T(12): 0.0; S(14): 0.0	0,45	-0,36
Ankrd50	F7BE84	SALQSAAWQGHVK	S(1): 100.0; S(5): 0.0	0,45	-0,29
Uqcrh	P99028	SQTEEDCTEELFDFLHAR	S(1): 8.2; T(3): 91.8; T(8): 0.0	-0,50	0,55
Abi1	Q8CBH5-2	VAILTDDEEEQKR	T(5): 100.0	-0,50	0,20
Zswim8	Q3UHH1-2	KQSAGPNSPTGGGGGGGSGGTR	S(3): 0.0; S(8): 99.9; T(10): 0.1; S(18): 0.0; T(21): 0.0	-0,51	0,05
Tns1	E9Q0S6	ETTSDPSRTPEEEPLNLEGLVAHR	T(2): 0.0; T(3): 0.0; S(4): 0.0; S(7): 12.8; T(9): 87.2	-0,51	0,16

Disease Models & Mechanisms • Supplementary information

Epb4.1l1	A2AUK5	SLPELDRDKSDSETEGLVFAR	S(1): 0.8; S(10): 99.2; S(12): 0.0; T(14): 0.0	-0,51	-0,03
Hcn1	O88704	RLGTPPGGGAAGK	T(4): 100.0	-0,52	0,34
Klc2	D3YXZ3	DSLDDLFPNEDEQSPAPSPGGGDVAAQHGGYEIPAR	S(2): 0.0; S(14): 100.0; S(18): 0.0; Y(31): 0.0	-0,52	0,73
Mcm6	P97311	DEEQTAESIK	T(5): 99.4; S(8): 0.6	-0,52	0,48
Tbc1d10b	Q8BHI9	ALESVPIMILPEPK	S(4): 100.0	-0,52	0,45
Ncam1	P13595	NPPEAATAPASPK	T(7): 0.0; S(11): 100.0	-0,52	0,40
Gpkow	Q53YU8	VEDLNTFGPK	T(6): 100.0	-0,53	-0,21
Gng3	P63216	GETPVNSTMSIGQAR	T(3): 100.0; S(7): 0.0; T(8): 0.0; S(10): 0.0	-0,53	0,48
Zc2hc1a	Q8BJ42	TTSKPLISVTAQSSTESTQDAYQDSR	T(1): 0.0; T(2): 0.0; S(3): 0.0; S(8): 0.0; T(10): 0.0; S(13): 9.5; S(14): 80.6; T(15): 9.5; S(17): 0.2; T(18): 0.2; Y(22): 0.0; S(25): 0.0	-0,53	0,73
Sept5	Q9Z2Q6	FGIHVYQFPECDSDEDEDFKQQDR	Y(6): 0.0; S(13): 100.0	-0,54	0,25
Ahi1	E9QP54	ERSPPLTPK	S(3): 100.0; T(7): 0.0	-0,54	0,19
Syt1	P46096	TLVMAVYDFDR	T(1): 0.0; Y(7): 100.0	-0,54	0,56
Gm4951	Q3U4W8	AVDWIFSHIDDLDAEAAMDISEGR	S(7): 0.0; S(21): 100.0	-0,54	0,47
Map2	P20357	YTVPLPSPVQDSENLSGESGSFYEGTDDK	Y(1): 0.0; T(2): 0.0; S(7): 99.7; S(12): 0.2; S(16): 0.0; S(19): 0.0; S(21): 0.0; Y(23): 0.0; T(26): 0.0	-0,54	0,75
Bai3	Q80ZD8	RMSDPESVSSVFSDTPIVV	S(3): 100.0; S(7): 0.0; S(9): 0.0; S(10): 0.0; S(13): 0.0; T(15): 0.0	-0,55	0,44
Map1a	Q9QYR6-2	ELALSSPEDLTQDFEELK	S(5): 4.0; S(6): 96.0; T(11): 0.0	-0,55	0,40
Iqsec2	E9QAD8	AQNPAYFEGKPASLDEGAMAGAR	Y(6): 0.0; S(13): 100.0	-0,56	0,04
Ppp1r1b	Q60829	AVQHLQTISNLSENQASEEEDELGELR	T(7): 0.0; S(9): 0.0; S(12): 0.0; S(17): 100.0	-0,56	0,91
Psma5	Q9Z2U1	ITSPLMEPSSIEK	T(2): 96.0; S(3): 4.0; S(9): 0.0; S(10): 0.0	-0,56	0,38
Rtn4	Q99P72	ESAEFSVLEYSEMGSSFNGSPK	S(2): 0.0; S(6): 0.0; Y(10): 0.0; S(11): 0.0; S(15): 0.3; S(16): 2.0; S(20): 97.6	-0,58	0,27
Ctnnd2	E9QKH8	TGSQHGPQNAAAATFQR	T(1): 8.4; S(3): 91.6; T(14): 0.0	-0,58	0,01
Map1b	P14873	SPPLLGSESPYEDFLSADSK	S(1): 100.0; S(7): 0.0; S(9): 0.0; Y(11): 0.0; S(16): 0.0; S(19): 0.0	-0,58	0,26
Syn2	Q64332	SQSLTNAFSFSESSFFR	S(1): 0.0; S(3): 100.0; T(5): 0.0; S(9): 0.0; S(11): 0.0; S(13): 0.0; S(14): 0.0	-0,58	0,05
Ube4b	Q9ERZ4	EPVANQDPVSPSLVQGR	S(10): 11.0; S(12): 89.0	-0,58	0,28
Ppme1	Q8BVL3	VTSSVPLPSGGTSSPSR	T(2): 0.0; S(3): 0.0; S(4): 0.0; S(9): 0.5; T(12): 6.3; S(13): 80.5; S(14): 6.3; S(16): 6.3	-0,58	0,31
Ccdc92	Q8VDN2	VDNSSLTGESEPQTRSPDFTNENPLETR	S(4): 0.0; S(5): 0.0; T(7): 0.7; S(10): 97.8; T(14): 0.7; S(16): 0.7; T(20): 0.0; T(27): 0.0	-0,59	0,16
Arhgef10	F7BQE4	FIVSATAFQNK	S(4): 93.3; T(6): 6.7	-0,60	0,90
Tp53bp1	P70399	GREDAVAEDVCIDLTCDSGSQAVPSPATR	T(15): 0.0; S(18): 0.0; S(20): 0.0; S(25): 99.3; T(28): 0.7	-0,60	0,71
Nup37	Q9CWE0	ASSFADMMGILK	S(2): 3.8; S(3): 96.2	-0,60	0,47
Arfgap1	Q9EPJ9-2	RSSDSWDVWGSGSASNNK	S(2): 7.1; S(3): 85.9; S(5): 7.1; S(11): 0.0; S(13): 0.0; S(15): 0.0	-0,60	-0,06
Rgs12	E9Q1K7	TLPDSQQVPSSPASK	T(1): 0.0; S(5): 0.0; S(10): 6.6; S(11): 92.9; S(14): 0.5	-0,60	0,56
Marcks	P26645	AEDGAAPSPSSETPKK	S(8): 0.0; S(10): 33.3; S(11): 33.3; T(13): 33.3	-0,60	-0,37
Pcsk1n	Q9QXS6	MAPTPIPTRSPSDSSTASTPIAEQIER	T(4): 33.2; T(8): 33.2; S(10): 33.2; S(12): 0.3; S(14): 0.0; S(15): 0.0; T(16): 0.0; S(18): 0.0; T(19): 0.0	-0,60	-0,11
Ntng1	F8WJ50	GTANTCDLTILTVPTSPPK	T(2): 0.0; T(5): 0.0; T(9): 0.0; T(12): 1.7; T(15): 11.7; S(16): 86.6	-0,60	0,33
Atp8a1	P70704	SESLQQNLLHGYAFSQDENGIVSQSEVIR	S(1): 0.0; S(3): 100.0; Y(12): 0.0; S(15): 0.0; S(23): 0.0; S(25): 0.0	-0,61	2,83
Mag	P20917	GESPELDLSYSHSDLGK	S(3): 100.0; S(9): 0.0; Y(10): 0.0; S(11): 0.0; S(13): 0.0	-0,61	0,12
Slc39a10	Q6P5F6	LNETELTDLEAQQESPPKNYLGVEEEK	T(4): 0.0; T(7): 0.0; S(15): 10.3; Y(20): 89.7	-0,62	0,47
Syngap1	F6SEU4	DLNSSIDLQSFMAR	S(4): 0.3; S(5): 99.7; S(10): 0.0	-0,62	-0,27
D430041D05Rik	D3YZ21	QSLNSPSPGETEMDLLVTR	S(2): 0.0; S(5): 100.0; S(7): 0.0; T(11): 0.0; T(18): 0.0	-0,62	0,14
Chga	P26339	GELEHSQQEEDGEEAMVGTPQGLFPQGGK	S(6): 100.0; T(19): 0.0	-0,63	0,55
Tmsb4x	P20065-2	TETQEKNPLPSK	T(1): 0.0; T(3): 100.0; S(11): 0.0	-0,63	0,15
Slc1a2	P43006-2	ESNSNQCVYAAHNSVVIDECK	S(2): 50.0; S(4): 50.0; Y(9): 0.0; S(14): 0.0	-0,63	0,36
Begain	F8WIG2	ADSFSEGDDLSQGHLAEPCFLR	S(3): 100.0; S(5): 0.0; S(11): 0.0	-0,63	0,05
Twf1	Q91YM2	AGSPLCNSNLQDSEEDVEPPSYHLFR	S(3): 100.0; S(8): 0.0; S(13): 0.0; S(21): 0.0; Y(22): 0.0	-0,64	0,32
Sh3gl1	Q62418-3	QLTQPETSYGR	T(3): 100.0; T(7): 0.0; S(8): 0.0; Y(9): 0.0	-0,64	0,28
Camsap2	Q8C0T7	SEGTLQGAATTSTARR	S(1): 0.0; T(4): 0.1; T(10): 98.7; T(11): 1.1; S(12): 0.0; T(13): 0.0	-0,64	0,50

Disease Models & Mechanisms • Supplementary information

Chrm2	Q9ERT9	RPTPATLVLTSDQSSPEIDEDR	T(3): 0.0; T(6): 0.0; T(10): 0.0; S(11): 0.0; S(14): 0.3; S(15): 99.7	-0,65	0,59
Lppr2	Q8VCY8-2	LSPWEDLSQAPTMDSPLEK	S(2): 0.0; S(8): 0.0; T(12): 0.0; S(15): 100.0	-0,66	0,38
Gm996	A2AJA9	QRSLEQLDELITDLVIDSR	S(3): 100.0; T(12): 0.0; S(18): 0.0	-0,66	0,43
Garnl3	A2AUQ6	GCSFESPSTAPQPPGPLMDPLTK	S(3): 78.3; S(6): 10.2; S(8): 10.2; T(9): 1.4; T(22): 0.0	-0,67	0,02
Larp4b	Q6A099	FQLEMYIK	Y(6): 100.0	-0,68	0,21
Kbtbd11	Q8BNN1	SGDLAPPESPSSPGQASPR	S(1): 0.0; S(9): 33.3; S(11): 33.3; S(12): 33.3; S(17): 0.0	-0,68	-0,18
Lppr4	Q7TME0	MSLQVMDTEPEGQSPPR	S(2): 0.0; T(8): 0.0; S(14): 100.0	-0,69	0,19
Apoa1bp	Q8K3I9	VPCNVEGISPELEK	S(9): 100.0	-0,69	0,74
Fam195b	Q3UED7	LYFLDMVTEDAKTLLK	Y(2): 100.0; T(8): 0.0; T(13): 0.0	-0,69	0,23
Cep170b	Q80U49	SQGPRDTDDDEEEPDPYGFIVQTAEIAEIAR	S(1): 0.2; T(7): 99.6; Y(17): 0.2; T(23): 0.0	-0,70	0,51
Brsk1	Q5PR73	CDESPNREVQSSEAEALAR	S(4): 100.0; S(11): 0.0; S(12): 0.0	-0,70	0,76
Echdc1	Q9D8U8	SVSVDLNVDPSLQIDIPDALSER	S(1): 12.1; S(3): 87.9; S(11): 0.0; S(21): 0.0	-0,70	-0,37
Gprin1	Q3UNH4	DLAAAAAQKSPSAEAAAPPPGPR	S(10): 100.0; S(12): 0.0	-0,71	0,33
Cldn11	Q60771	FYYSSGSSSPTHAK	Y(2): 0.0; Y(3): 0.0; S(4): 0.0; S(5): 0.0; S(7): 0.0; S(8): 0.5; S(9): 99.5; T(11): 0.0	-0,73	0,31
Slc1a3	P56564	DVEMGNSVIEENEMK	S(7): 100.0	-0,73	0,92
Coro1a	O89053	RATPEPSGTPSSDTVSR	T(3): 100.0; S(7): 0.0; T(9): 0.0; S(11): 0.0; S(12): 0.0; T(14): 0.0; S(16): 0.0	-0,73	-0,28
Adcy5	P84309	SVSPPGYAAQTAASPAPR	S(1): 0.0; S(3): 100.0; Y(7): 0.0; T(11): 0.0; S(14): 0.0	-0,75	0,01
Slc39a10	Q6P5B8	ANSSGGGGGGGSVGSGSSK	S(3): 0.0; S(4): 0.0; S(12): 0.0; S(15): 0.8; S(17): 49.6; S(18): 49.6	-0,75	0,06
Pcdh1	F7BJK1	IHLPLNYPPGSPDLGR	Y(7): 0.0; S(11): 100.0	-0,75	0,31
Acap2	Q6ZQ18	SIGETISLQVEVESR	S(1): 100.0; T(5): 0.0; S(7): 0.0; S(14): 0.0	-0,76	0,66
Gng3	P63216	MKGETPVNSTMSIGQAR	T(5): 4.8; S(9): 47.4; T(10): 47.4; S(12): 0.5	-0,76	0,81
Map6d1	Q14BB9	SDVAVPLTLHGYSDPGSEESGADCSVSR	S(1): 0.0; T(8): 0.0; Y(12): 0.4; S(13): 0.4; S(17): 49.2; S(20): 49.2; S(25): 0.4; S(27): 0.4	-0,76	-0,19
Clasp2	E9Q8N5	NTGNGTQSSMGSPLTRPTPR	T(2): 0.0; T(6): 0.0; S(8): 0.0; S(9): 0.6; S(12): 99.4; T(15): 0.0; T(18): 0.0	-0,78	0,25
Dlg4	Q62073	SIQDLTVTGTEPGQVSSR	S(1): 100.0; T(6): 0.0; T(8): 0.0; T(10): 0.0; S(16): 0.0; S(17): 0.0	-0,78	0,33
Homer2	Q9JME5	AFYGSEEDEAKGPGSEEAATAALPAR	Y(3): 0.6; S(5): 99.4; S(15): 0.0; T(20): 0.0	-0,79	0,09
Pvrl1	Q9JKC6	ADPVLLNNHSNLKPAPTVPAAPSSPDATSEPK	S(10): 0.0; T(17): 0.0; S(23): 48.6; S(24): 48.6; T(28): 1.4; S(29): 1.4	-0,79	0,53
Pnpo	Q91XF0	GLATGDSPLGPMTHHGEEDWVYER	T(4): 0.3; S(7): 99.7; T(13): 0.0; Y(22): 0.0	-0,79	0,34
Apc	B2RUG9	TPASKSPSEGPGATTSPR	T(1): 0.0; S(4): 49.9; S(6): 49.9; S(8): 0.2; T(14): 0.0; T(15): 0.0; S(16): 0.0	-0,80	-0,03
Begain	F8WIG2	GDIYCSDPALYCPDER	Y(4): 0.0; S(6): 100.0; Y(11): 0.0	-0,81	0,71
Gas7	B1ATI9	KSTGDSQNLGSSSPGR	S(2): 0.0; T(3): 0.0; S(6): 0.1; S(11): 33.3; S(12): 33.3; S(13): 33.3	-0,82	0,32
Gprin1	Q3UN16	RLSLGSPDSR	S(3): 100.0; S(6): 0.0; S(9): 0.0	-0,83	0,68
Map6d1	Q149S1	LLSEVEELNMSLR	S(3): 2.5; S(11): 97.5	-0,83	0,09
Cacna1b	A2AIR9	EVAEVSPMSAANISIAAR	S(6): 99.9; S(9): 0.1; S(14): 0.0	-0,83	0,45
Atp13a1	Q9EPB4	EIHPYLVMDLEQS	Y(5): 0.0; S(13): 100.0	-0,84	0,19
Col3a1	P08121	GENGSPGAPGAPGHPGPPGPVGPSGK	S(5): 100.0; S(24): 0.0	-0,85	-0,17
Tbc1d22a	Q8R574	LGIAVIHGEAQDAESDLVDGRHSPPMVR	S(15): 0.0; S(23): 100.0	-0,86	0,19
Trim3	Q3B7Z2	GATVLPANPPGSAGSGK	T(3): 100.0; S(12): 0.0; S(15): 0.0	-0,87	0,71
Kcnma1	J3QN27	RLEDEQPPTLSPK	T(9): 0.3; S(11): 99.7	-0,87	0,41
Atp1a3	Q6PIC6	VDNSSLTGESEPQTRSPDCTHDNPLETR	S(4): 16.7; S(5): 16.7; T(7): 16.7; S(10): 16.7; T(14): 16.7; S(16): 16.7; T(20): 0.0; T(27): 0.0	-0,88	0,70
Map1a	Q9QYR6-2	MASPPPSGPPSAAHTPFHQSPVEEK	S(3): 98.9; S(7): 1.1; S(11): 0.0; T(15): 0.0; S(20): 0.0	-0,88	0,77
Ncor2	F8VQL9	TSVLGSSEDAIEPVSPPEGMTEPGHAR	T(1): 0.0; S(2): 0.0; S(6): 0.0; S(7): 0.0; S(15): 100.0; T(21): 0.0	-0,90	0,55
Map3k7	Q61937	CGSGPVHISGQHLVAVEEDAESEDEDEEDVK	S(3): 0.0; S(9): 0.0; S(22): 100.0	-0,91	0,58
Dzank1	Q8BYW1-2	DAPISPPAQK	S(5): 100.0	-0,94	-0,06
Cacng8	F7CZ64	DASPGGPGGPGFASTDISMYTLSR	S(3): 100.0; S(14): 0.0; T(15): 0.0; S(18): 0.0; Y(20): 0.0; T(21): 0.0; S(23): 0.0	-0,94	0,66
Col1a1	P11087-2	GDAGPAGPKGEPGSPGENGAPGQMGPR	S(14): 100.0	-0,94	-0,06
Caskin1	Q6P9K8-2	KTPQSLEMMAIESPPPSEPAAAECQSPK	T(2): 0.0; S(5): 0.0; S(13): 0.0; S(17): 9.8; S(26): 90.2	-0,95	1,34

Disease Models & Mechanisms • Supplementary information

Akt2	Q60771	FYYSSGSSSPTHAK	Y(2): 0.0; Y(3): 0.0; S(4): 0.0; S(5): 1.9; S(7): 32.0; S(8): 32.0; S(9): 32.0; T(11): 1.9	-0,95	0,30
Syngap1	F6SEU4	EGSIGGSGGSGGGGGGGLKPSITK	S(3): 0.0; S(7): 33.2; S(10): 33.2; S(21): 33.2; T(23): 0.3	-0,96	0,32
Sirt2	Q8VDQ8-2	EHANIDAQSGSQAPNPSTTISPGKSPPPAK	S(9): 0.0; S(11): 0.0; S(17): 0.0; T(18): 0.0; T(19): 0.0; S(21): 0.0; S(25): 100.0	-0,96	0,51
Pclo	Q9QYX7	GGSLGCQTETDPDTQSPPYMGATSPPKDK	S(3): 0.0; T(8): 0.0; T(10): 0.0; T(14): 20.0; S(16): 20.0; Y(19): 20.0; T(23): 20.0; S(24): 20.0	-0,97	0,52
Lrrc8e	Q64514-2	VNESSHYDLAFTDVHFKPGQIR	S(4): 25.0; S(5): 25.0; Y(7): 25.0; T(12): 25.0	-0,99	1,16
Col3a1	P08121	GFPGNPGPPGSPGAAGHQGAIGSPGPAGPR	S(11): 50.0; S(23): 50.0	-1,00	-0,07
Nbea	Q9EPJ9-2	EWSLESSPAQNWTPPQPK	S(3): 0.0; S(6): 0.0; S(7): 0.0; T(13): 100.0	-1,00	0,57
Rims1	Q99MX7	SSYGAPPSAPPPPPPPPPQGGSQR	S(1): 2.2; S(2): 48.9; Y(3): 48.9; S(8): 0.0; S(22): 0.0	-1,01	0,58
Rmdn3	Q3UID0	DMDEPSPVPNVEEVTLPK	S(6): 100.0; T(15): 0.0	-1,02	0,57
Il1a	P01582	ESRVTVSATSSNGK	S(2): 0.0; T(5): 0.0; S(7): 3.6; T(9): 46.4; S(10): 46.4; S(11): 3.6	-1,03	-0,10
Mtfr1l	Q9CW07	SFSLPADPILQAAK	S(1): 3.8; S(3): 96.2	-1,06	0,17
Pnpo	Q91WH7	VDNSSLTGESEPQTRSPECTHESPLETR	S(4): 0.0; S(5): 0.0; T(7): 0.2; S(10): 0.7; T(14): 77.4; S(16): 15.3; T(20): 3.2; S(23): 3.2; T(27): 0.0	-1,07	0,21
Col4a1	P02463	VVPLPGPPGAAGLPGSPGFPGPQGDR	S(16): 100.0	-1,10	0,71
Dopey2	Q3UHL1	SATPATDGRATPATEESTVPATQSSALPAAK	S(1): 0.0; T(3): 0.0; T(6): 1.1; T(11): 98.9; T(14): 0.0; S(17): 0.0; T(18): 0.0; T(22): 0.0; S(24): 0.0; S(25): 0.0	-1,10	0,17
Cep170b	Q80U49	GASPVTPSTTPPPPTDPQLTK	S(3): 0.0; T(6): 0.0; S(8): 0.0; T(9): 0.0; T(10): 0.4; T(15): 99.6; T(20): 0.0	-1,15	0,17
Braf	F6SZ47	SSSAPNVHINTIEPVNIDDLIR	S(1): 47.2; S(2): 47.2; S(3): 5.6; T(11): 0.0	-1,19	1,04
Strn4	P58404-2	SLELNGAGEPVEGAPR	S(1): 100.0	-1,23	-0,41
Slc8a2	Q8K4Z3	RGSETMAGAAVK	S(3): 100.0; T(5): 0.0	-1,25	0,42
Cecr6	Q99MV7-3	SAVSINDQLVK	S(1): 100.0; S(4): 0.0	-1,26	1,22
Cplx2	P84086	AALEQPCEGSLTRPK	S(10): 99.6; T(12): 0.4	-1,29	0,07
Syap1	Q9D2P8	HQPAASPVVVR	S(6): 100.0	-1,34	0,36
Scn11a	Q9QZS8-2	VHAPSATPSTSAQPASPVAR	S(5): 0.0; T(7): 0.0; S(9): 0.0; T(10): 0.0; S(11): 0.0; S(16): 100.0	-1,36	-0,18
Tmem163	Q8C996	GHAPSTAAPAPSPAPMSSSVQSDEER	S(5): 0.0; T(6): 0.0; S(12): 0.0; S(17): 84.4; S(18): 7.5; S(19): 0.7; S(22): 7.5	-1,36	0,86
Ehd3	Q9QXW9	NHPGSDTSPEAEASSGGGGVALK	S(5): 6.3; T(7): 6.3; S(8): 87.5; S(14): 0.0; S(15): 0.0	-1,39	1,64
Kif1b	Q60520	VEDALSYLDQVK	S(6): 0.1; Y(7): 99.9	-1,57	1,20
Nedd4l	E9PXB7	SLSSPTVTLSAPLEGAK	S(1): 1.4; S(3): 76.0; S(4): 9.9; T(6): 9.9; T(8): 1.4; S(10): 1.4	-1,57	0,96
Vcpip1	Q8CCF0-2	FPELESLVPNALDYIRTVK	S(6): 83.0; Y(14): 14.3; T(17): 2.7	-1,66	0,43
Slc2a13	Q3UHJ0-2	EQGSSGLGSGSSGGGGSSSGLGSGYIGR	S(4): 0.3; S(5): 0.3; S(9): 3.9; S(11): 16.4; S(12): 74.0; S(17): 3.9; S(18): 1.0; S(19): 0.3; S(23): 0.0; Y(25): 0.0	-1,67	0,69
Maoa	Q640R3	QNSLEYMDQNDDRLK	S(3): 99.7; Y(6): 0.3	-1,70	0,81
Hspa12a	Q8K0T0	EQDSPPMKPGALDAIR	S(4): 100.0	-2,16	0,21

Supplementary Table S6. Proteins up and down phosphorylated in the cerebellum of Ts65Dn mice and reverted by L41 treatment.

Gene name	Protein Group Accessions	Sequence	phosphoRS Site Probabilities	Ts65Dn vs WT	Ts65Dn +/-L41
Aqr	A2AQA7	IFTQLEEFR	T(3): 100.0	3,79	-3,42
C130026I21Rik	Q3KNJ6	GSAWANLSELVQGSR	S(2): 99.0; S(8): 0.5; S(14): 0.5	3,30	-2,67
Robo2	E9PYK2	SVVIGGLFPGIQYR	S(1): 0.1; Y(13): 99.9	2,50	-2,14
Tmbim1	D3Z0A5	AGSDSFRPGEWDDR	S(3): 100.0; S(5): 0.0	2,25	-2,47
Madd	A2AGR0	ATLSDSEIETNSATSAIFGK	T(2): 6.4; S(4): 6.4; S(6): 87.2; T(10): 0.0; S(12): 0.0; T(14): 0.0; S(15): 0.0	1,88	-0,18
Map1b	P14873	TTRSPDTSAYCYETMEK	T(1): 0.0; T(2): 0.2; S(4): 99.8; T(7): 0.0; S(8): 0.0; Y(10): 0.0; Y(12): 0.0; T(14): 0.0	1,70	-1,15
Ubap2l	Q80X50	RYPSSISSSPQK	Y(2): 0.0; S(4): 0.0; S(5): 0.0; S(7): 0.0; S(8): 0.0; S(9): 100.0	1,58	-1,56
Psmd4	O35226	AAAASAAEAGIATPGTEDSDDALLK	S(5): 0.0; T(13): 100.0; T(16): 0.0; S(19): 0.0	1,58	-0,54
Efhd2	Q8C845	ADLNQGIGEPQSPSRR	S(12): 98.8; S(14): 1.2	1,57	-1,47
Gabbr2	Q80T41	DLEEVTMQLQDTPEK	T(6): 0.0; T(12): 100.0	1,57	-1,30
Slc43a2	Q8CGA3	LCLSTVDLEVK	S(4): 50.0; T(5): 50.0	1,41	-0,29
Map1b	P14873	DYNASASTISPPSSMEEDK	Y(2): 0.0; S(5): 0.0; S(7): 0.0; T(8): 0.2; S(10): 99.7; S(13): 0.0; S(14): 0.0	1,39	-1,48
Htt	P42859	ASDPSPATPDSESVIVAMER	S(2): 0.0; S(5): 0.3; T(8): 97.7; S(11): 1.8; S(13): 0.3	1,39	-0,60
Pkp4	Q68FH0	TEPEQGTLYSPEQTSLHESEGSLGNSR	T(1): 0.0; T(7): 0.0; Y(9): 0.0; S(10): 0.4; T(14): 49.8; S(15): 49.8; S(19): 0.0; S(22): 0.0; S(26): 0.0	1,38	-1,42
Impact	O55091	KTEEVEVESEEDPILEHPPENPVK	T(2): 0.0; S(9): 100.0	1,36	-0,08
Ctage5	H3BK44	AFLSPPTLLEGPLR	S(4): 99.3; T(7): 0.7	1,34	-0,37
Pacs2	Q3V3Q7	EPSGQPEDSPEAETSTLDVFTEK	S(3): 0.0; S(9): 100.0; T(14): 0.0; S(15): 0.0; T(16): 0.0; T(21): 0.0	1,33	-0,11
Mpdz	Q8VBX6-2	NVQGLGITIAGYIGDK	T(8): 98.6; Y(12): 1.4	1,31	-0,50
Epn2	Q5NCM5	AGGSPASYHGSPEASLCPQHR	S(4): 0.0; S(7): 0.0; Y(8): 0.0; S(11): 50.0; S(15): 50.0	1,31	-1,46
Eif4b	Q8BGD9	SPPYTAFLGNLPYDVTEDSIK	S(1): 33.3; Y(4): 33.3; T(5): 33.3; Y(13): 0.0; T(16): 0.0; S(19): 0.0	1,28	-0,92
Bcas3	Q0VF62	EGSIETLSNSSGSTSGSIPR	S(3): 100.0; T(6): 0.0; S(8): 0.0; S(10): 0.0; S(11): 0.0; S(13): 0.0; T(14): 0.0; S(15): 0.0; S(17): 0.0	1,27	-1,17
Arhgef2	H3BJU7	LSPPHSPR	S(2): 100.0; S(6): 0.0	1,23	-1,04
Coro1a	O89053	RATPEPSGTPSSDTVSR	T(3): 99.8; S(7): 0.1; T(9): 0.0; S(11): 0.0; S(12): 0.0; T(14): 0.0; S(16): 0.0	1,14	-1,00
Tom1l2	Q5SRX1	GIEFPMADLDALSPIHTPQR	S(13): 100.0; T(17): 0.0	1,06	0,01
Slc1a4	O35874	SEEETSPLVTHQNPAGPVAIAPELESK	S(1): 0.0; T(5): 1.0; S(6): 99.0; T(10): 0.0; S(26): 0.0	1,06	-0,43
Cacna1g	P39061-2	QTVSVPGPPGPPGPPGPPGAMGASAG QVR	T(2): 6.0; S(4): 93.5; S(24): 0.5	1,06	-0,23
Ank1	Q02357-5	EYSLSPSQVNGQQR	Y(2): 0.0; S(3): 0.0; S(5): 99.5; S(7): 0.5	1,04	-1,08
Dmxl2	Q8BPN8-2	RQSESIAAPPVASEDMDK	S(3): 50.0; S(5): 50.0; S(13): 0.0	1,00	-1,10
Clns1a	Q923F1	LGEESKEPLSDEDEEDNDDVEPISEFR	S(5): 0.0; S(10): 100.0; S(24): 0.0	0,99	-0,29
Add2	Q9QYB8	SPSTESQLMSK	S(1): 0.0; S(3): 95.1; T(4): 4.8; S(6): 0.0; S(10): 0.0	0,99	-0,26
Upf1	Q9EPU0-2	AGLSQSLFER	S(4): 10.2; S(6): 89.8	0,99	-0,55
Epb4.1l1	A2AUK8	GPSSQEDESGGLEDSPDRGACSTPEMP QFESVK	S(3): 0.0; S(4): 0.0; S(9): 3.0; S(15): 14.9; S(22): 79.2; T(23): 3.0; S(31): 0.0	0,98	-0,65
Palmd	Q3UVT7	SNGPTHTSPTRPTPQPR	S(1): 0.0; T(5): 0.3; T(7): 49.9; S(8): 49.9; T(10): 0.0; T(13): 0.0	0,96	-0,79
Kif17	A2AM72	EYQEEIK	Y(2): 100.0	0,94	-0,75
Ppig	A2AR02	SETPPHWR	S(1): 0.0; T(3): 100.0	0,94	-0,84
Plekha6	P16014	WASSREDAGAPVEDSQGQTK	S(3): 50.0; S(4): 50.0; S(15): 0.0; T(19): 0.0	0,93	-0,09

Disease Models & Mechanisms • Supplementary information

K1967	Q8VDP4	SVASNQSEMEYSSLQDMPK	S(1): 0.0; S(4): 99.7; S(7): 0.3; Y(11): 0.0; S(12): 0.0; S(13): 0.0	0,93	-1,59
Osbp	Q3B7Z2	GDMSDEDDENEFFDAPEIITMPENLGHK	S(4): 100.0; T(20): 0.0	0,92	0,09
Dpysl5	Q9EQF6	EMGTPLADTPTRPVTR	T(4): 100.0; T(9): 0.0; T(11): 0.0; T(15): 0.0	0,91	-0,70
Shank3	Q4ACU6	LGAEEERPGTPELAPAPMQAAAVAEPM PSPR	T(10): 100.0; S(29): 0.0	0,90	-0,81
Phkb	Q7TSH2	EGPNFITMEGTVSDHIER	T(7): 10.0; T(11): 10.0; S(13): 80.1	0,89	-0,42
Ryr2	E9Q401	RISQTSQVSIDAAHGYSPR	S(3): 100.0; T(5): 0.0; S(6): 0.0; S(9): 0.0; Y(16): 0.0; S(17): 0.0	0,88	-0,88
Acin1	O88737	SQGSFEYQDTQDHDYGGR	S(1): 0.0; S(4): 100.0; Y(7): 0.0; T(10): 0.0; Y(15): 0.0	0,87	-0,73
Nfix	Q3TYK3	SPAAGSSQSSGWPNDVDAGSPR	S(1): 0.0; S(6): 0.0; S(7): 0.0; S(9): 0.0; S(10): 0.0; S(20): 100.0	0,87	-0,38
Arfip2	Q8K221	HPSHSTSPSGPGDEVAR	S(3): 0.0; S(5): 0.0; T(6): 0.4; S(7): 99.6; S(9): 0.0	0,86	-0,47
Ralgps1	F6WZ83	LVSSKEDLAGPSAGSSSAR	S(3): 50.0; S(4): 50.0; S(12): 0.0; S(15): 0.0; S(16): 0.0; S(17): 0.0	0,86	-0,69
Sh3bp5l	Q99LH9	EAQQETQK	T(6): 100.0	0,85	-0,38
Slc38a10	Q5I012-4	QDVFGEGSEER	S(8): 100.0	0,85	-0,67
Rtn1	Q8K0T0	SPPVAMETASTGMAAVPDALDHSPSSTL K	S(1): 0.0; T(8): 97.2; S(10): 0.0; T(11): 0.0; S(23): 0.9; S(25): 0.9; S(26): 0.9; T(27): 0.0	0,84	-1,35
Ebag9	Q9D0V7	KLSGDQITLPTTVDYSSVPK	S(3): 99.9; T(8): 0.1; T(11): 0.0; T(12): 0.0; Y(15): 0.0; S(16): 0.0; S(17): 0.0	0,83	-0,39
Slc9a1	Q61165	CLSDPGPHPEPGEGEPFIPK	S(3): 100.0	0,82	-0,23
Arid1b	E9Q6R4	SSPALAAPDASVDPK	S(1): 5.9; S(2): 94.1; S(11): 0.0	0,82	-0,74
Kif2a	P28740-1	EFGISPSDIPFSQGGGSRPDLSPSYDYD DFSPSITR	S(5): 0.3; S(7): 0.3; S(12): 32.9; S(17): 32.9; S(22): 32.9; S(24): 0.3; Y(25): 0.3; Y(27): 0.0; S(31): 0.0; S(33): 0.0; T(35): 0.0	0,81	-0,62
Pkp4	Q68FH0	AQSPSYVTSTGVSPSR	S(3): 0.0; S(5): 0.0; Y(6): 0.0; T(8): 0.0; S(9): 0.1; T(10): 0.1; S(13): 9.5; S(15): 90.2	0,80	-0,75
Oxct1	Q9D0K2	HHTKFYTDPVEAVK	T(3): 100.0; Y(6): 0.0; T(7): 0.0	0,77	-1,00
Aak1	Q3UHJ0-2	ILSDVTHSAVFGVPASK	S(3): 4.6; T(6): 47.7; S(8): 47.7; S(16): 0.0	0,77	-0,03
Phyhipl	Q8BGT8	NLSLEAIQLCDR	S(3): 100.0	0,77	0,30
Fbxl20	Q9CZV8-4	VHAYFAPVTPPPSVGGSR	Y(4): 0.0; T(9): 100.0; S(13): 0.0; S(17): 0.0	0,77	-0,64
Gm996	A2AJA9	SLEQLDELITDLVIDSR	S(1): 100.0; T(10): 0.0; S(16): 0.0	0,76	-0,35
Ckb	Q04447	RGTGGVDTAAVGGVFDVSNADR	T(3): 100.0; T(8): 0.0; S(18): 0.0	0,75	-0,41
Tom1l2	Q5SRX1	GIEFPMADLDALSPIHTPQR	S(13): 100.0; T(17): 0.0	0,74	-0,43
Sptbn4	E9PX29	QESADHEGPHSLTLGR	S(3): 100.0; S(11): 0.0; T(13): 0.0	0,73	-0,82
Usp5	Q3U4W8	SAAESISESVPVGPK	S(1): 0.0; S(5): 98.9; S(7): 1.0; S(9): 0.1	0,73	-0,45
Rapgef2	E9QNQ4	SLGSLSQGSANATVLDVAQTGGHK	S(1): 97.9; S(4): 2.0; S(6): 0.1; S(9): 0.1; T(13): 0.0; T(20): 0.0	0,72	-0,24
Ptges3	Q9R0Q7	DWEDDSDEDMSNFDR	S(6): 100.0; S(11): 0.0	0,72	-0,24
Fxr1	Q61584-4	RGPNYTSGYGTNSELSNPSETESER	Y(5): 0.0; T(6): 0.0; S(7): 0.0; Y(9): 0.0; T(11): 0.0; S(13): 0.0; S(16): 0.2; S(19): 98.2; T(21): 1.5; S(23): 0.0	0,71	0,00
Tcp11l2	Q8K1H7	NIRPHIQHHLVEYERNK	Y(13): 100.0	0,70	-0,12
Tanc1	E9QAF9	ADNCSPVAEEETTGSAESVLPK	S(5): 100.0; T(12): 0.0; T(13): 0.0; S(15): 0.0; S(18): 0.0	0,70	0,19
Dmxl2	Q8C7U1	AQLLHALSLDEGGPEPSLSDSSSGGSFG R	S(8): 0.8; S(17): 58.2; S(19): 12.6; S(21): 12.6; S(22): 12.6; S(23): 3.0; S(26): 0.2	0,69	-0,93
Tln2	E9PUM4	FGLEGDEESTMLEESVSPK	S(9): 0.0; T(10): 0.0; S(15): 0.2; S(17): 99.8	0,69	-0,41
Nup85	Q8R480	QVIFSAEQTYELMR	S(5): 47.3; T(9): 47.3; Y(10): 5.4	0,69	-0,15
Tiam1	G3UWG2	SNATNSSYSPPTGR	S(1): 0.0; T(4): 0.0; S(6): 1.3; S(7): 0.2; Y(8): 1.3; S(9): 87.3; T(12): 10.0	0,69	-0,26
Trim3	Q3TDT0	ALRPGDLPPSPDDVK	S(10): 100.0	0,68	-0,73
Mllt6	B1AR09	HSSGGGGGAGGGSSGGGGGGSSSAS GGGGGTGGGSGNSFLSGR	S(2): 0.0; S(3): 0.0; S(13): 0.0; S(14): 0.0; S(21): 0.0; S(22): 0.3; S(23): 0.8; S(25): 0.3; T(31): 70.7; S(35): 21.1; S(38): 6.6; S(41): 0.3	0,68	-0,18

Disease Models & Mechanisms • Supplementary information

Gapvd1	Q6PAR5-6	EAPWPSAQSEIRTISAYK	S(6): 100.0; S(9): 0.0; T(13): 0.0; S(15): 0.0; Y(17): 0.0	0,68	-0,81
Ube4b	Q9ES00	LAGGQTSQPTTPLTSPQR	T(6): 0.0; S(7): 0.0; T(10): 0.0; T(11): 0.0; T(14): 50.0; S(15): 50.0	0,68	-0,09
Ldhc	D3YVR7	SLNPAIGTDSDK	S(1): 100.0; T(8): 0.0; S(10): 0.0	0,67	-0,17
Elmo3	Q8BYZ7-2	LLESVTLSSPALGQLVK	S(4): 4.5; T(6): 45.5; S(8): 45.5; S(9): 4.5	0,67	-0,88
Ckb	Q04447	RGTGGVDTAAVGGVFDVSNADR	T(3): 97.0; T(8): 3.0; S(18): 0.0	0,67	-0,27
Dmwd	Q08274	LGDPDGAGEPPSTPSGLGAGGDR	S(12): 7.5; T(13): 85.1; S(15): 7.5	0,66	-0,20
Atp6v0a2	P15920-2	KDSEEEVSLLGNQDIEEGNSR	S(3): 100.0; S(8): 0.0; S(20): 0.0	0,66	-0,27
Hspa5	P20029	LYGSGGPPPTGEEDTSEKDEL	Y(2): 0.0; S(4): 0.0; T(10): 0.0; T(15): 50.0; S(16): 50.0	0,66	-0,01
Pcp2	P12660	RNSSPQPQTQAP	S(3): 94.4; S(4): 5.6; T(9): 0.0	0,66	-0,40
Arhgap21	B7ZCJ1	RNSEGSEASCTEGSLTPSLDSR	S(3): 91.9; S(6): 8.1; S(9): 0.0; T(11): 0.0; S(14): 0.0; T(16): 0.0; S(18): 0.0; S(21): 0.0	0,65	-0,38
Prune2	Q52KR3-5	VAISTVNMTLEVRK	S(4): 0.0; T(5): 0.0; T(9): 100.0	0,65	-0,37
Uty	D6RFT4	GSEVQSIIIK	S(2): 0.0; S(6): 100.0	0,64	-0,71
Madd	A2AGR0	ATLSDSEIETNSATSAIFGK	T(2): 0.1; S(4): 92.1; S(6): 7.8; T(10): 0.0; S(12): 0.0; T(14): 0.0; S(15): 0.0	0,63	-0,67
Ryr2	E9Q401	RISQTSQVSIDAAHGYSPR	S(3): 99.6; T(5): 0.4; S(6): 0.0; S(9): 0.0; Y(16): 0.0; S(17): 0.0	0,63	-0,64
Syn1	O88935	QTSQQPAGPPAQQRPPPQGGPPQPGPGPQR	T(2): 15.6; S(3): 84.4	0,62	-1,67
Rtn4	Q99P72	DDSPKEYTDLEVSNK	S(3): 100.0; Y(7): 0.0; T(8): 0.0; S(13): 0.0	0,62	-0,44
Gprin1	Q3UNH4	AETVSPGEVDAMTLGK	T(3): 4.6; S(5): 95.4; T(13): 0.0	0,61	-0,41
Tiam1	G3UWG2	SNATNSSYSPPTGR	S(1): 0.0; T(4): 0.0; S(6): 0.9; S(7): 0.1; Y(8): 0.9; S(9): 97.1; T(12): 0.9	0,60	-0,20
Pvrl1	Q9JKF6	AGIPQHHPPMAQNLQYPDDSDDEK	Y(16): 0.7; S(20): 99.3	0,60	-0,69
Sp100	E9Q4Y0	GGDTSDTESSIIIR	T(4): 0.8; S(5): 96.8; T(7): 0.8; S(9): 0.8; S(10): 0.8	0,60	-0,21
Sltm	Q8CH25-2	DVQDAIAQSPEK	S(9): 100.0	0,59	-0,20
Lmtk3	Q5XJV6	ALVNGEPMSPEAGEK	S(9): 100.0	0,59	-0,53
Map2	P20357	ASQPSPPAQEAGYSTLAQSYTPDHPSELPEEPSSPQER	S(2): 0.0; S(5): 0.0; Y(13): 0.0; S(14): 0.0; T(15): 0.0; S(19): 0.0; Y(20): 0.0; T(21): 0.0; S(26): 0.0; S(33): 12.1; S(34): 87.9	0,58	0,09
Pabpc4	Q6PHQ9	LAMDFGGAGAAQQGLTDSCQSGGVPTAVPNLAPR	T(16): 13.6; S(18): 28.8; S(21): 28.8; T(26): 28.8	0,58	-0,59
Pitpnm3	Q3UHE1-2	FPAQGSIEIHEDGEEGCSQR	S(6): 100.0; S(18): 0.0	0,58	-0,40
Ahdc1	Q6PAL7	ANSEASSSEGQSSLSSLEK	S(3): 91.1; S(6): 8.8; S(7): 0.1; S(8): 0.0; S(12): 0.0; S(13): 0.0; S(15): 0.0; S(16): 0.0	0,58	0,23
Gpsm1	Q6IR34-3	AAQSSVTASPQTEEFFDLIASSQSR	S(4): 0.0; S(5): 0.0; T(7): 8.5; S(9): 83.0; T(12): 8.5; S(21): 0.0; S(22): 0.0; S(24): 0.0	0,57	-0,02
Sept_4	P28661-2	LTRESGTDFPIPAVPPGTDPETEK	T(2): 11.1; S(5): 11.1; T(7): 77.9; T(18): 0.0; T(22): 0.0	0,57	-0,22
Speg	E9QQ25	MPSIPEEPEHGDLER	S(3): 100.0	0,57	-0,19
Apba1	B2RUJ5	APTPGGGHPDSPGLPAPAGQQQR	T(3): 0.0; S(11): 100.0	0,56	-0,53
Elmo1	Q8BPU7	IAFDAESEPNNSSGSMEK	S(7): 0.0; S(12): 0.0; S(13): 5.8; S(15): 94.2	0,56	0,01
Mobp	Q9D2P8	ATSPQRPK	T(2): 50.0; S(3): 50.0	0,56	-0,13
Dmxl1	Q6PNC0	ISEASWLPEEHYSSSPEK	S(2): 0.0; S(5): 0.0; Y(12): 0.0; S(13): 0.6; S(14): 0.6; S(15): 98.9	0,55	-0,42
Slc7a5	Q9Z127	AVATPAAAAAEEER	T(4): 100.0	0,55	-0,68
Pclo	Q9QYX7	SVDTSVQTDDEDQDEWDMPSR	S(1): 0.0; T(4): 0.6; S(5): 0.6; T(8): 98.8; S(20): 0.0	0,55	-0,50
Akt2	Q60823	YFDDEFTAQSITITPPDRYDSLDPLELDQR	Y(1): 0.0; T(7): 0.8; S(10): 96.6; T(12): 0.8; T(14): 0.8; Y(19): 0.8; S(21): 0.0	0,54	-0,15
Bsn	O88737	SPQVLYSPVSPLSPHR	S(1): 0.0; Y(6): 0.0; S(7): 0.0; S(10): 5.6; S(13): 94.4	0,54	-0,93
Rab12	P35283	RPAGGSLGAVSPALSGGQAR	S(6): 46.5; S(11): 46.5; S(15): 7.1	0,54	-0,13
Hcn2	O88703	DSASPGAASGLDPLDSAR	S(2): 7.6; S(4): 92.4; S(9): 0.0; S(16): 0.0	0,54	-0,47
Rabl6	Q64511	KTSFDQDSDVDIFPSDFTSEPPALPR	T(2): 0.0; S(3): 0.0; S(8): 100.0; S(15): 0.0; T(18): 0.0; S(19): 0.0	0,54	0,03

Disease Models & Mechanisms • Supplementary information

Epn1	Q80VP1	SPGAFDMSGVGGSLAESVGSPPPAATPTPTPPTR	S(1): 0.0; S(8): 0.2; S(13): 4.0; S(17): 4.0; S(20): 91.5; T(26): 0.2; T(28): 0.0; T(30): 0.0; T(33): 0.0	0,54	-0,17
Pml	F7BTZ2	ATSPPHLDGTSNPESTVPEK	T(2): 50.0; S(3): 50.0; T(10): 0.0; S(11): 0.0; S(15): 0.0; T(16): 0.0	0,52	-0,64
Dpysl2	O08553	NLHQSGFSLSGAQIDDNIPR	S(5): 49.9; S(8): 49.9; S(10): 0.2	0,52	-0,43
Prex1	Q69ZK0	HPDHTAVQSALQAMK	T(5): 0.1; S(9): 99.9	0,52	-0,41
Tom1l2	Q5SRX1	AAETVPDLPSPPTEAPAPASNTSTR	T(4): 0.0; S(10): 50.0; T(13): 50.0; S(20): 0.0; T(22): 0.0; S(23): 0.0; T(24): 0.0	0,52	-0,86
Shisa6	F6VQZ6	QYNHPILSSATQTPTHEKPR	Y(2): 0.0; S(8): 0.0; S(9): 0.0; T(11): 0.0; T(13): 100.0; T(15): 0.0	0,52	-0,95
Ranbp2	Q9ERU9	NRPGYVSEEEEDDEDYEMAVK	Y(5): 0.0; S(7): 100.0; Y(16): 0.0	0,52	-0,37
Csnk1a1	E9Q4G7	AAQQAASSSGQGQQAQTPTGK	S(7): 0.0; S(8): 0.0; S(9): 0.0; T(17): 100.0; T(19): 0.0	0,52	-0,40
Fbxo16	Q9QZM9	QSPSLAFRSSSSLR	S(2): 0.0; S(4): 0.0; S(9): 2.7; S(10): 32.4; S(11): 32.4; S(12): 32.4	0,51	-0,72
Ace	P09470	GPQFGSEVELR	S(6): 100.0	0,51	-0,77
Scap	Q6GQT6	RDSCGGGAFETQENWER	S(3): 100.0; T(11): 0.0	0,51	-0,81
Armc10	Q9D0L7	SAEDLTDGSYDDILNAEQLK	S(1): 49.4; T(6): 49.4; S(9): 0.6; Y(10): 0.6	0,51	-0,28
Syn1	O88935	SQSLTNAFNLPEPAPPRPSLSQDEVK	S(1): 0.0; S(3): 0.0; T(5): 0.0; S(19): 50.0; S(21): 50.0	0,51	-0,14
Phkb	Q7TSH2	SGSVYEPLK	S(1): 5.8; S(3): 93.8; Y(5): 0.4	0,51	-0,84
Tmem108	Q8BHE4	EIQSLETSEDQLSEPR	S(4): 0.0; T(7): 0.0; S(8): 0.1; S(13): 99.9	0,50	-0,64
Tnks1bp1	P58871	SSGSLSPGLETEDPLEAR	S(1): 0.0; S(2): 0.0; S(4): 0.0; S(6): 100.0; T(11): 0.0	0,50	0,02
Mink1	Q9JM52-2	QNSDPTSEGPGPSPNPPSWVRPDNEAPPK	S(3): 95.3; T(6): 2.3; S(7): 2.3; S(13): 0.0; S(18): 0.0	0,49	-0,54
Arfgef3	Q3UGY8	VAAPSSSPSAEAEYWR	S(5): 0.2; S(6): 0.2; S(7): 49.8; S(9): 49.8; Y(14): 0.0	0,49	-0,40
Lgalsl	Q8VED9	LDDGHLNNSLGSPVQADVYFPR	S(9): 0.0; S(12): 100.0; Y(19): 0.0	0,49	0,18
Amph	Q7TQF7	TPSPPEEPSPLPSPTASPNHTLAPASPAPVRPR	T(1): 2.9; S(3): 95.8; S(9): 0.6; S(13): 0.6; T(15): 0.1; S(17): 0.0; T(21): 0.0; S(26): 0.0	0,49	-0,29
Ppp1r14a	Q91VC7	QPGFPQPSPSDDPSLSPR	S(8): 0.0; S(10): 0.0; S(14): 0.6; S(16): 99.4	0,49	0,03
Tmem163	Q8C996	GHAPSTAAPAPSPAPMSSSVQSDEER	S(5): 0.0; T(6): 0.0; S(12): 0.0; S(17): 0.0; S(18): 0.0; S(19): 0.0; S(22): 100.0	0,48	0,15
Rab3a	P63011	MSESLDTADPAVTGAK	S(2): 33.3; S(4): 33.3; T(7): 33.3; T(13): 0.0	0,47	-0,41
Prpf40b	D3Z4N6	TGWDTSESELSEGELER	T(1): 0.0; T(5): 0.0; S(6): 0.0; S(8): 0.0; S(11): 100.0	0,46	-0,01
Sec16a	A2AIX1	FTGSFDDDAEIHR	T(2): 0.2; S(4): 99.8	0,46	-0,63
Wdr47	Q8CGF6	TSPMSHSFANFHYPGVQNLSR	T(1): 98.9; S(2): 1.1; S(5): 0.0; S(7): 0.0; Y(13): 0.0; S(20): 0.0	0,46	-0,28
Bod1l	E9Q6J5	TSTPVILEGAQEETDTR	T(1): 0.1; S(2): 0.7; T(3): 99.2; T(14): 0.0; T(16): 0.0	0,46	-0,22
Rps28	G3UYV7	TGSQGQCTQVR	T(1): 0.5; S(3): 99.5; T(8): 0.0	0,46	-0,47
Rps3	P62908	DEILPTTPISEQK	T(6): 3.8; T(7): 96.2; S(10): 0.0	0,46	-0,33
Dbi	Q4VWZ5	TQPTDEEMLFIYSHFK	T(1): 0.0; T(4): 0.0; Y(12): 95.8; S(13): 4.2	0,45	-0,23
Cep76	Q0VEJ0	ELNFVTDSVDQELPSSPK	T(6): 0.0; S(8): 0.0; S(15): 7.8; S(16): 92.2	0,45	-0,37
Ybx1	P62960	NYQQNYQNSESGEK	Y(2): 0.0; Y(6): 0.0; S(9): 99.4; S(11): 0.6	0,45	-0,60
N4bp3	A2A699	TGLCSPEDNSLTPLLDEVVAPEGR	T(1): 3.3; S(5): 92.8; S(10): 0.7; T(12): 3.3	0,45	-0,16
Prrc2c	Q3TLH4	ATSTSPNSQSSK	T(2): 0.0; S(3): 0.0; T(4): 0.2; S(5): 99.8; S(8): 0.0; S(10): 0.0; S(11): 0.0	0,45	-0,26
Egfr	Q01279	ELVEPLTPSGEAPNQAHLR	T(7): 100.0; S(9): 0.0	0,45	-0,18
Tns1	E9Q0S6	WDSYDNFSGHR	S(3): 99.7; Y(4): 0.3; S(8): 0.0	0,45	-0,25
Dmtn	Q9WV69	LQSTEFSPSGSEAGSPGLQNGEGQR	S(3): 46.9; T(4): 46.9; S(7): 6.1; S(9): 0.1; S(11): 0.0; S(15): 0.0	0,45	-0,27
Arhgef12	F8VQN6	SEGVQDAQEPQSLVGSPSTR	S(1): 0.0; S(12): 10.9; S(16): 67.4; S(18): 10.9; T(19): 10.9	0,45	-0,31
Myo5a	D3YZ62	RTSSIADEGTYTLDSILR	T(2): 48.1; S(3): 48.1; S(4): 3.8; T(10): 0.0; Y(11): 0.0; T(12): 0.0; S(15): 0.0	0,44	-0,18
Znfx1	Q8R151	IQGSAGEISTSQEQLK	S(4): 0.1; S(9): 33.3; T(10): 33.3; S(11): 33.3	0,44	0,01
Gm15800	E9Q2E4	TEGTPPPPGQPAK	T(1): 0.8; T(4): 99.2	0,44	-0,25
Sptbn2	Q68FG2	SSEAAHGATLPTR	S(1): 100.0; S(2): 0.0; T(9): 0.0; T(12): 0.0	0,44	-0,63

Disease Models & Mechanisms • Supplementary information

Slc25a46	Q9CQS4	SFGSGTELGHWVTTPPDIPGSR	S(1): 0.0; S(4): 0.0; T(6): 0.0; T(13): 0.7; T(14): 99.3; S(21): 0.0	0,44	-0,33
Astn1	Q61137-2	QLTTISLIIR	T(3): 33.3; T(4): 33.3; S(6): 33.3	0,44	-0,14
Sphkap	E9PUC4	QSSTESITEEFYR	S(2): 0.0; S(3): 99.7; T(4): 0.3; S(6): 0.0; T(8): 0.0; Y(12): 0.0	0,44	-0,09
Nefh	P19246	EGAKSPAEAK	S(5): 100.0	0,44	-0,27
Shank1	D3YZU5	HYTVGSYDSFDAPSDYIIK	Y(2): 0.1; T(3): 0.7; S(6): 7.4; Y(7): 7.4; S(9): 84.4; S(14): 0.0; Y(16): 0.0	0,44	-0,77
Ifi44	Q8BV66	DMLILSALK	S(6): 100.0	0,44	-0,27
Arhgef12	Q8R4H2	SEGVQDAEPQSLVGSPSTR	S(1): 0.0; S(11): 0.0; S(15): 83.9; S(17): 8.0; T(18): 8.0	0,44	0,03
Purb	O35295	DSLGDFIEHYAQLGPSSPEQLAAGAEEGGGPR	S(2): 0.0; Y(10): 0.0; S(16): 8.3; S(17): 91.7	0,43	-0,05
Hspa12a	Q8K0U4	ETAPTSTYSSPAR	T(2): 0.0; T(5): 0.0; S(6): 0.0; T(7): 0.0; Y(8): 0.6; S(9): 92.5; S(10): 6.8	0,43	-0,42
Tgfb1i1	Q62219-7	EGCPSPPGQTSK	S(5): 88.1; T(10): 6.0; S(11): 6.0	0,43	-0,18
Eif3b	Q8JZQ9	AKPAAQSEEETATSPAASPTPQSAER	S(7): 0.0; T(11): 0.0; T(13): 8.7; S(14): 91.3; S(18): 0.0; T(20): 0.0; S(23): 0.0	0,43	0,21
Tpr	Q7M739	QTPQAPQSPR	T(2): 0.0; S(8): 100.0	0,42	-0,48
Kcnc1	E9PVV3	AALANEDCPHIDQALTPDEGLPFTR	T(16): 100.0; T(24): 0.0	0,42	-0,13
Akap1	O08715	SESSGNLPSVADTR	S(1): 33.3; S(3): 33.3; S(4): 33.3; S(9): 0.1; T(13): 0.0	0,42	-0,29
Dclk1	Q9JLM8	REESEEGFQIPATITER	S(4): 100.0; T(13): 0.0; T(15): 0.0	0,42	-0,10
Sod1	P08228	DGVANVSIEDR	S(7): 100.0	0,42	-0,05
Sipa1l2	Q80TE4-2	EYGSTSSIDR	Y(2): 0.5; S(4): 32.0; T(5): 32.0; S(6): 32.0; S(7): 3.6	0,41	-0,38
Reps2	A2AFI8	SFSVEREPQENNSNYPDEPWR	S(1): 0.8; S(3): 99.2; S(13): 0.0; Y(15): 0.0	0,41	0,21
Tmem163	Q8C996	GHAPSTAAPAPSPAPMSSSVQSDEER	S(5): 0.0; T(6): 0.0; S(12): 0.0; S(17): 0.0; S(18): 0.0; S(19): 0.0; S(22): 100.0	0,41	0,23
Epb4.1l1	A2AUK8	GPSSQEDESGGLEDSPDRGACSTPEMPQFESVK	S(3): 0.0; S(4): 0.0; S(9): 13.1; S(15): 84.3; S(22): 2.2; T(23): 0.4; S(31): 0.0	0,41	-0,42
Pianp	Q6P1B3	QEESQQPLTDLSPAGVTVLGAFGDSPTPTPDHEEPR	S(4): 0.0; T(9): 0.0; S(12): 0.0; T(17): 0.0; S(25): 99.5; T(27): 0.3; T(29): 0.3	0,41	-0,24
Atp1a3	Q6PIC6	DVAGDASESALLK	S(7): 100.0; S(9): 0.0	0,41	0,24
Rgl3	Q3UYI5	EPPPPGSPPASPGPQSPSTK	S(7): 100.0; S(11): 0.0; S(16): 0.0; S(18): 0.0; T(19): 0.0	0,41	-0,16
Rab11fip5	Q8R361	GSPSLGASPHHSSTGEEK	S(2): 0.0; S(4): 0.0; S(8): 100.0; S(12): 0.0; S(13): 0.0; T(14): 0.0	0,41	-0,57
Prkar2a	Q8K1M3	RVSVCAETFNPDEEEEDNDPR	S(3): 100.0; T(8): 0.0	0,41	-0,22
Srrm1	E9QKA4	RESPSPAPKPR	S(3): 100.0; S(5): 0.0	0,41	0,16
Ksr2	M0QW59	LKPPGTPPPSSR	T(6): 100.0; S(10): 0.0; S(11): 0.0	0,41	-0,60
Dsg2	O55111	LDLSIIVTNK	S(4): 100.0; T(8): 0.0	0,41	0,00
Ywhae	P62259	AAFDDAIAELDTLSEESYK	T(12): 0.0; S(14): 100.0; S(17): 0.0; Y(18): 0.0	0,41	0,01
Zfp292	Q9Z2U2-2	LESTENTEVK	S(3): 0.0; T(4): 0.1; T(7): 99.9	0,41	-0,16
Ppapdc3	Q91WB2	KASGPSTQPPPSSDGAR	S(3): 99.3; S(6): 0.7; T(7): 0.0; S(12): 0.0; S(13): 0.0	0,40	-0,28
Ep300	E9PYJ8	TDGKEEEEQPSTSATQSSPAPGQSK	T(1): 0.0; S(11): 0.0; T(12): 0.0; S(13): 0.2; T(15): 0.0; S(17): 49.4; S(18): 49.4; S(24): 1.0	0,40	-0,52
Kmt2b	Q64514-2	LGFTVTVGNNRGIYLR	T(4): 50.0; T(6): 50.0; Y(14): 0.0	0,40	0,03
Atp1a3	Q6PIC6	SPDCTHDNPLETR	S(1): 100.0; T(5): 0.0; T(12): 0.0	0,40	-0,21
Srrm2	Q8BTI8	SGTPPRPGSVTNMQADECTATPQR	S(1): 0.6; T(3): 99.4; S(9): 0.0; T(11): 0.0; T(19): 0.0; T(21): 0.0	0,40	-0,02
Gpbp1	Q6NXH3-3	SSTFPQTDVLSSSLEAEHR	S(1): 0.0; S(2): 0.0; T(3): 0.0; T(7): 0.0; S(11): 0.6; S(12): 7.4; S(13): 92.0	0,40	-0,45
Map4	P27546	DVAPPMEEEIVPGNDTTSPK	T(16): 4.6; T(17): 47.7; S(18): 47.7	0,40	-0,38
Sfr1	Q8BP27-2	ENPPSPHSNSSGK	S(5): 100.0; S(8): 0.0; S(10): 0.0; S(11): 0.0	0,40	-0,22
Pik3c2g	D3Z6L6	FLGHAQTFGGIK	T(7): 100.0	0,39	-0,05
Golga3	P55937-1	SSTSVVSEVSPSSETDNR	S(1): 0.0; S(2): 0.0; T(3): 0.0; S(4): 0.0; S(7): 0.0; S(10): 49.8; S(12): 49.8; S(13): 0.3; T(15): 0.0	0,39	-0,09

Disease Models & Mechanisms • Supplementary information

Park2	Q9WVS6	SHETNASGGDEPQSTSEGSIWESR	S(1): 10.2; T(4): 89.6; S(7): 0.2; S(14): 0.0; T(15): 0.0; S(16): 0.0; S(19): 0.0; S(23): 0.0	0,39	-0,33
Wipf3	E9Q2D0	AQLVSPPAPPTK	S(5): 100.0; T(11): 0.0	0,39	-0,44
Raly	Q64012-2	LPAPQEDTASEAGTPQGEVQTR	T(8): 0.0; S(10): 0.0; T(14): 100.0; T(21): 0.0	0,39	0,12
Rph3a	P47708	WHQLQNENHVSSD	S(11): 0.3; S(12): 99.7	0,39	-0,14
Tanc1	E9QAF9	QQGPPPAPANDSDNEEDAPASSLK	S(12): 100.0; S(21): 0.0; S(22): 0.0	0,39	-0,31
Gucy1b2	E9Q6H0	VGIHTGPVLAGVVGDK	T(5): 100.0	0,39	-0,46
Ctnnd2	E9QKH8	DGWSQYHFVASSSTIER	S(4): 0.0; Y(6): 0.0; S(11): 25.0; S(12): 25.0; S(13): 25.0; T(14): 25.0	0,39	-0,20
Psmd4	O35226-2	AAAASAAEAGIATPGTEGER	S(5): 0.0; T(13): 100.0; T(16): 0.0	0,39	-0,07
ptplad1	Q8K2C9	EGSPETLTNLK	S(3): 0.0; T(6): 0.7; T(8): 99.3	0,39	-0,24
Unc13c	Q8K0T7	NAWLSPNDSDRELESDLSR	S(5): 0.0; S(9): 100.0; S(15): 0.0; S(18): 0.0	0,39	-0,32
Gsk3a	D3Z7E5	TSSFAEPGGGGGGGGGGPGGSASGPGGTGGGK	T(1): 5.2; S(2): 47.4; S(3): 47.4; S(21): 0.0; S(23): 0.0; T(28): 0.0	0,39	-0,49
Lhx9	Q9WUH2-2	SYFAINHNPDAK	S(1): 8.8; Y(2): 91.2	-0,35	0,46
Sp9	Q64HY3	MATSILGEEPR	T(3): 50.0; S(4): 50.0	-0,35	0,31
THMS1	Q8BGW0-4	EFPTAYDLQIAK	T(4): 0.4; Y(6): 99.6	-0,35	0,34
Stub1	Q9WUD1	LGTGGGGSPDKSPSAQELK	T(3): 0.0; S(8): 100.0; S(12): 0.0; S(14): 0.0	-0,35	0,32
Kcnrg	Q2TUM3-3	DGALFSFILDFLR	S(6): 100.0	-0,35	0,57
Ppfia3	B8QI35	RGSALGPDEAGGELER	S(3): 100.0	-0,35	0,48
Farp1	F8VPU2	LGAPENSGISTLER	S(7): 0.0; S(10): 94.5; T(11): 5.5	-0,35	0,15
Camsap2	Q8C1B1	LNQSSPDNLTDTK	S(4): 94.4; S(5): 5.6; T(10): 0.0; T(12): 0.0	-0,35	0,16
Lnp	Q7TQ95	ADSVPNLEPSEESLVTK	S(3): 100.0; S(10): 0.0; S(13): 0.0; T(16): 0.0	-0,35	0,09
Plcl2	Q8K394	EFQVSFQVQK	S(5): 100.0	-0,35	0,26
Ap3d1	O54774	VDIITEEMPENALPSDEDDKDPNDPYR	T(5): 0.0; S(15): 100.0; Y(26): 0.0	-0,35	0,71
Nin	E9Q4S3	DLYVENAQLLK	Y(3): 100.0	-0,35	0,32
Sptbn2	Q68FG2	LEQSNVPEGPGSGTGDESSGPR	S(4): 0.0; S(12): 99.6; T(14): 0.4; S(18): 0.0; S(19): 0.0	-0,35	-0,10
Slc25a31	Q3V132	SDGLIGLYQGFGVSVQGIIVYR	S(1): 0.0; Y(8): 99.8; S(14): 0.2; Y(21): 0.0	-0,35	0,07
Rims3	Q80U57	SSSISGEICGSQQAGGGAGTTTAK	S(1): 0.4; S(2): 47.6; S(3): 4.4; S(5): 47.6; S(11): 0.0; T(20): 0.0; T(21): 0.0; T(22): 0.0	-0,36	0,37
Sde2	Q8K1J5	EDGIDAVEVAADRPGSPR	S(16): 100.0	-0,36	0,25
Rtn4	Q99P72	LSASPQEVGKPYLESFQPNLHITK	S(2): 0.0; S(4): 100.0; Y(12): 0.0; S(15): 0.0; T(23): 0.0	-0,36	-0,03
Il16	O54824	SASPETPASPGK	S(1): 0.9; S(3): 98.2; T(6): 0.9; S(9): 0.0	-0,36	2,20
Map4	P27546	DMSPSAETEAPLAK	S(3): 100.0; S(5): 0.0; T(8): 0.0	-0,36	0,33
Nlgn2	Q69ZK9	RLSPPGGSGSGVPGGGPLLPTAGR	S(3): 100.0; S(8): 0.0; S(10): 0.0; T(21): 0.0	-0,36	0,24
Sparcl1	P70663	AAGSKEHIPHTEQQDQEGK	S(4): 100.0; T(11): 0.0	-0,36	0,30
Slc1a3	P56564	DVEMGNSVIEENEMK	S(7): 100.0	-0,36	-0,02
Nfia	Q02780-2	SVEDEMDSPGEEPFYTGQGR	S(1): 0.0; S(8): 100.0; Y(15): 0.0; T(16): 0.0	-0,36	0,22
Marcks	P26645	VNGDASPAAAEPGAK	S(6): 100.0	-0,36	0,79
Hnrnpa3	A2AL12	SSGSPYGGGYGSGGGSGGYGSR	S(1): 49.8; S(2): 49.8; S(4): 0.3; Y(6): 0.0; Y(10): 0.0; S(12): 0.0; S(16): 0.0; Y(19): 0.0; S(21): 0.0	-0,36	0,20
Tut1	Q8R3F9	LGGQEKSFEEGK	S(7): 100.0	-0,36	0,35
Tjp2	Q9Z0U1	SYHEAYEPDYGGGYSPSYDR	S(1): 0.0; Y(2): 0.0; Y(6): 0.0; Y(10): 0.0; Y(14): 0.6; S(15): 98.8; S(17): 0.6; Y(18): 0.0	-0,37	-0,01
Hist1h4a	P62806	TVTAMDVVYALK	T(1): 0.0; T(3): 0.0; Y(9): 100.0	-0,37	-0,27
Cd209c	D6RJ18	VQQLGSLDEEHLIPSGTR	S(6): 100.0; S(15): 0.0; T(17): 0.0	-0,37	0,19
Gabbr2	Q80T41	TCKDPIEDINSPEHIQR	T(1): 0.0; S(11): 100.0	-0,37	-0,01
Dagla	Q6WQJ1	LLSPVAAASAAR	S(3): 100.0; S(9): 0.0	-0,37	-0,12
Arhgap35	Q91YM2	TSFSVGSDDELGPIR	T(1): 0.0; S(2): 0.0; S(4): 0.0; S(7): 100.0	-0,37	0,56
Izumo4	D3Z690	TTPAFLSQNIK	T(1): 50.0; T(2): 50.0; S(7): 0.0	-0,37	-0,05
Sash1	F8VQK5	SCETLEGPEPVESWPR	S(1): 100.0; T(4): 0.0; S(13): 0.0	-0,37	0,30
Gm2933	Q1LZI5	LMYVLALSSDLLVIQK	Y(3): 90.2; S(8): 9.7; S(9): 0.1	-0,37	0,43
Trim28	Q62318	RPAASSAAAASAAASSPAGGGGEAQELLEHCGVCR	S(5): 0.0; S(6): 0.0; S(11): 0.0; S(15): 50.0; S(16): 50.0	-0,37	0,77

Disease Models & Mechanisms • Supplementary information

Rplp2	P99027	KEESEESDDDMGFGLFD	S(4): 50.0; S(7): 50.0	-0,37	0,18
Pnpo	Q91XF0	GLATGDSPLGPMTHHGEEDWVYER	T(4): 50.0; S(7): 50.0; T(13): 0.0; Y(22): 0.0	-0,38	0,28
Trim28	E9Q6J5	GSDDVLVSGEVPECEVGHMSPR	S(2): 0.0; S(8): 0.0; S(20): 100.0	-0,38	-0,07
Acot11	Q8VHQ9	KSISHPESGDPPTMAEGEGYR	S(2): 50.0; S(4): 50.0; S(8): 0.0; T(13): 0.0; Y(20): 0.0	-0,38	-0,01
Unc13c	Q8K0T7	RPSQPPASSTPEK	S(3): 0.0; S(8): 0.0; S(9): 0.6; T(10): 99.4	-0,38	0,35
Pkp4	Q68FH0	DGWNQNHFITPVSTLER	T(10): 100.0; S(13): 0.0; T(14): 0.0	-0,38	0,36
Rnf20	Q5DTM8	ALVVPEPEPDSDSNQER	S(11): 0.5; S(13): 99.5	-0,38	0,20
Npm1	Q61937	CGSGPVHISGQHLVAVEEDAESEDEDE EDVK	S(3): 0.0; S(9): 0.0; S(22): 100.0	-0,38	-0,08
Pde4b	B1AWC9	QNDVEIPSPTQK	S(8): 96.0; T(10): 4.0	-0,38	0,10
Park2	Q9WVS6	SHETNASGGDEPQSTSEGSIWESR	S(1): 1.6; T(4): 96.8; S(7): 1.6; S(14): 0.0; T(15): 0.0; S(16): 0.0; S(19): 0.0; S(23): 0.0	-0,38	0,10
Oxr1	Q4KMM3	ESLPIELDQLSGR	S(2): 0.0; S(11): 100.0	-0,38	-0,10
Pkp4	Q68FH0	AEQYPGSPDSWV	Y(4): 0.0; S(7): 100.0; S(10): 0.0	-0,39	-0,23
Map2	P20357	DGSPDAPATPEKEEVAFSEYK	S(3): 100.0; T(9): 0.0; S(18): 0.0; Y(20): 0.0	-0,39	0,54
Plekha6	Q7TQG1	SIHEVDISNLEAALR	S(1): 100.0; S(8): 0.0	-0,39	-0,07
Mgrn1	Q9D074	ESSSPESFGTEEGDEPSLK	S(2): 1.2; S(3): 9.6; S(4): 79.4; S(7): 9.6; T(10): 0.2; S(17): 0.0	-0,39	0,26
Mink1	Q9JM52-2	QNSDPTSEGPGPSPNPPSWVRPDNEAP PK	S(3): 99.7; T(6): 0.3; S(7): 0.0; S(13): 0.0; S(18): 0.0	-0,39	0,50
Gja1	P23242	MGQAGSTISNSHAQPFDFPDDSQNAK	S(6): 25.0; T(7): 25.0; S(9): 25.0; S(11): 25.0; S(22): 0.0	-0,39	0,16
Trappc10	F8VQF9	RQESGSSLEPPSGLALEDGAHVLR	S(4): 33.3; S(6): 33.3; S(7): 33.3; S(12): 0.0	-0,39	0,07
Pcif1	P59114	QLSEEQPSGNGVK	S(3): 100.0; S(8): 0.0	-0,40	0,08
Aatk	B1AZF9	AVSFFDDVTVYLFDQESPTR	S(3): 0.0; T(9): 0.0; Y(11): 0.0; S(17): 50.0; T(19): 50.0	-0,40	0,06
Atp1a2	Q6PIE5	CIELSCGSVR	S(5): 0.0; S(8): 100.0	-0,40	0,07
Bag6	Q3UF95	ENASPAPGTTAEEAMSR	S(4): 100.0; T(9): 0.0; T(10): 0.0; S(16): 0.0	-0,40	0,30
Mobp	Q9D2P8	HQPAASPVVVR	S(6): 100.0	-0,40	0,08
Ctnnd2	E9QKH8	TSTAPSSPGVDSVPLQR	T(1): 0.0; S(2): 0.0; T(3): 0.0; S(6): 50.0; S(7): 50.0; S(12): 0.0	-0,40	0,16
Mllt6	B1AQX9	EVSGPNETSSPGSEKPSGSR	S(3): 0.0; T(8): 33.2; S(9): 33.2; S(10): 33.2; S(13): 0.3; S(17): 0.0; S(19): 0.0	-0,40	0,44
Fam195b	Q3UGS4	SPTNSSEIFTPAHEENVR	S(1): 100.0; T(3): 0.0; S(5): 0.0; S(6): 0.0; T(10): 0.0	-0,40	0,25
Gdf7	P43029-2	TALAGTRGAQGSGGGGGGGGGGGGG GGGGGGGAGR	T(1): 9.5; T(6): 3.5; S(12): 87.0	-0,40	0,22
Bsn	O88737	TLPSPPPEEAHLPLAGQVPSQLYAASLL QR	T(1): 0.9; S(4): 99.1; S(20): 0.0; Y(23): 0.0; S(26): 0.0	-0,40	0,31
H2afy2	Q8CCK0	EGTSNSTSEDGPGDGFTILSSK	T(3): 20.0; S(4): 20.0; S(6): 20.0; T(7): 20.0; S(8): 20.0; T(17): 0.0; S(20): 0.0; S(21): 0.0	-0,41	0,44
Naf1	Q3UMQ8	NDQEPPPEVLDFSDDEKEK	S(13): 100.0	-0,41	0,41
Nck1	Q99M51	KPSVPDTASPADDSFVDPGER	S(3): 0.0; T(7): 0.1; S(9): 99.9; S(14): 0.0	-0,41	0,23
Hnrnpc	Q9Z204-2	SAAEMYGSSFDLDYDFQR	S(1): 0.0; Y(6): 0.0; S(8): 0.9; S(9): 99.1; Y(14): 0.0	-0,41	0,57
Lppr4	Q7TME0	MSLQVMDTEPEGQSPPR	S(2): 0.0; T(8): 0.0; S(14): 100.0	-0,41	0,11
Bsn	O88737	LYSSMSDTNLAEAGLNYHAQR	Y(2): 48.0; S(3): 48.0; S(4): 0.3; S(6): 3.7; T(8): 0.0; Y(17): 0.0	-0,41	-0,14
Cstf2t	Q8C7E9	QGGGQPSSFSPGQSQVTPQDQEK	S(7): 0.0; S(8): 0.0; S(10): 99.9; S(14): 0.0; T(17): 0.0	-0,41	1,02
Gtf3c1	Q8K284-2	VAASPRPGAEEQAEAQAPAQLAAPEDA DAGGPR	S(4): 100.0	-0,41	0,63
Top2b	Q64511	FDSNEEDTASVFAPSFGLK	S(3): 100.0; T(8): 0.0; S(10): 0.0; S(15): 0.0	-0,42	0,44
Srrm2	Q8BTI8	SPVPSAFSDQSR	S(1): 100.0; S(5): 0.0; S(8): 0.0; S(11): 0.0	-0,42	0,23
Inpp5f	Q8CDA1	SPSADSIHTR	S(1): 100.0; S(3): 0.0; S(6): 0.0; T(9): 0.0	-0,42	0,33
Zfpm2	Q8CCH7	SSPEVSFER	S(1): 0.4; S(2): 99.6; S(6): 0.0	-0,42	0,16
Caskin2	Q8VHK1	VGLSPDSPAGDRNSVGSEGSVGSIR	S(4): 99.0; S(7): 1.0; S(14): 0.0; S(17): 0.0; S(20): 0.0; S(23): 0.0	-0,42	0,26
Brsk1	Q5RJI5	SSGGTPLHSPLHTPR	S(1): 0.0; S(2): 0.0; T(5): 0.0; S(9): 100.0; T(13): 0.0	-0,42	0,17
Cacna1a	P97445	EMGTDGYSDSEHYLPMEGQTR	T(4): 0.3; Y(7): 49.7; S(8): 49.7; S(10): 0.3; Y(13): 0.0; T(20): 0.0	-0,42	0,42

Disease Models & Mechanisms • Supplementary information

Phf3	B2RQG2	NTVDIVDKPENSPQR	T(2): 0.0; S(12): 100.0	-0,42	0,49
Arhgap21	B7ZCJ1	DQGEPVSPSEDEPFSWPGPK	S(7): 100.0; S(9): 0.0; S(15): 0.0	-0,42	-0,07
Bag6	Q3UF95	ENASPAPGTTAEEAMSR	S(4): 99.9; T(9): 0.1; T(10): 0.0; S(16): 0.0	-0,43	-0,15
Trank1	Q8BV79	AEQAGSPLLSK	S(6): 98.4; S(10): 1.6	-0,43	0,12
Arfgap1	Q9EPJ9-2	RSSDSWDVWGSGSASNNK	S(2): 33.3; S(3): 33.3; S(5): 33.3; S(11): 0.0; S(13): 0.0; S(15): 0.0	-0,43	0,93
Epn2	Q8CHU3	AGGSPASYHGSTSPR	S(4): 100.0; S(7): 0.0; Y(8): 0.0; S(11): 0.0; T(12): 0.0; S(13): 0.0	-0,43	0,00
Kcnj13	P86046	DGHSTLQMDGAQR	S(4): 50.0; T(5): 50.0	-0,44	-0,18
Mier1	Q5UAK0-4	APSPPPTASNSSNSQSEK	S(3): 0.8; T(7): 90.7; S(9): 8.5; S(11): 0.0; S(12): 0.0; S(14): 0.0; S(16): 0.0	-0,44	0,89
Vat1l	Q80TB8	TPTPLMANDSTETSEAGEEEEDHEGDSENK	T(1): 0.0; T(3): 2.0; S(10): 2.0; T(11): 2.0; T(13): 12.5; S(14): 81.4; S(27): 0.0	-0,44	0,09
Fam169a	Q5XG69	SQSEEQSEASSEHLEQFTQSAEK	S(1): 0.0; S(3): 1.1; S(7): 9.1; S(10): 9.1; S(11): 80.5; T(18): 0.1; S(20): 0.0	-0,44	0,64
Krt15	Q61414	EVASNTEMIQTSK	S(4): 0.0; T(6): 0.0; T(11): 1.0; S(12): 99.0	-0,44	0,61
Rps6kc1	Q8BLK9-2	GVDLLLEGVQGESSPTR	S(13): 49.9; S(14): 49.9; T(16): 0.2	-0,44	0,35
Spag7	Q7TNE3	EFAPSDEELDSYR	S(5): 100.0; S(11): 0.0; Y(12): 0.0	-0,45	0,37
Mroh2a	F5H8M8	CLGSLQGQVSSSAMAEGMEALTK	S(4): 0.0; S(10): 0.1; S(11): 3.5; S(12): 3.5; T(22): 93.0	-0,45	0,42
CR025	Q8BH50	RDSSESQLASTESDKPTTGR	S(3): 50.0; S(4): 50.0; S(6): 0.0; S(10): 0.0; T(11): 0.0; S(13): 0.0; T(17): 0.0; T(18): 0.0	-0,45	0,92
Slc12a4	Q9JIS8	LESLYSDEEEESVAGADK	S(3): 3.5; Y(5): 48.3; S(6): 48.3; S(12): 0.0	-0,45	0,15
Dclk1	Q9JLM8	SKSPASTSSVNGTPGSQLSTPR	S(1): 49.2; S(3): 49.2; S(6): 0.5; T(7): 0.5; S(8): 0.5; S(9): 0.0; T(13): 0.0; S(16): 0.0; S(19): 0.0; T(20): 0.0	-0,46	-0,06
Sparcl1	P70663	AEKPSALNSEEETHEQSTEQDK	S(5): 33.3; S(9): 33.3; T(13): 33.3; S(17): 0.0; T(18): 0.0	-0,46	0,39
Abca8b	Q8K440-2	ELTESHAEIIR	T(3): 15.1; S(5): 84.9	-0,46	0,66
Dnajc6	Q80TZ3	GASSPDMEPSYGGGLFDMVK	S(3): 0.4; S(4): 99.6; S(10): 0.0; Y(11): 0.0	-0,46	-0,04
Akt1	P31750	SGSPSDNSGAEEMEVSLAKPK	S(1): 0.0; S(3): 99.0; S(5): 0.9; S(8): 0.0; S(16): 0.0	-0,46	0,13
Caskin1	Q6P9K8-2	SVSESSPGDSPVKPPEGSSGAAR	S(1): 0.0; S(3): 0.0; S(5): 0.0; S(6): 0.0; S(10): 100.0; S(18): 0.0; S(19): 0.0	-0,46	0,29
Arhgap12	B2RUJ8	IQQDSESGDELSSSSTEQMR	S(5): 0.0; S(7): 100.0; S(12): 0.0; S(13): 0.0; S(14): 0.0; S(15): 0.0; T(16): 0.0	-0,46	0,33
Serpinb6a	F8WIV2	LTIMDPLQEANGTFALNLLK	T(2): 50.0; T(13): 50.0	-0,46	-0,01
Prkar2b	P31324	RASVCAEAYNPDEEEDDAESR	S(3): 95.2; Y(9): 4.8; S(20): 0.0	-0,46	0,81
Pkp4	Q68FH0	VGSPLTLTDAQTR	S(3): 100.0; T(6): 0.0; T(8): 0.0; T(12): 0.0	-0,46	0,05
Vmn2r3	E9Q107	YLIGIIGAGGSTMSLAGAR	Y(1): 0.6; S(11): 73.5; T(12): 13.0; S(14): 13.0	-0,46	-0,04
Mtmr2	Q9Z2D1	ASSPAQCVTPVQTVV	S(2): 50.0; S(3): 50.0; T(9): 0.0; T(13): 0.0	-0,47	0,13
Sparcl1	P70663	AAGSKEHIPHTEQQDQEGK	S(4): 100.0; T(11): 0.0	-0,47	0,34
Polr2a	P08775	YSPTSPTYSPTSPK	Y(1): 0.5; S(2): 0.5; T(4): 98.6; S(5): 0.5; T(7): 0.0; Y(8): 0.0; S(9): 0.0; T(11): 0.0; S(12): 0.0	-0,47	0,30
Anks1b	Q8BIZ1	NSSTGPTPDCSPPSPDTALK	S(2): 0.0; S(3): 0.0; T(4): 0.0; T(7): 0.0; S(11): 0.0; S(14): 100.0; T(17): 0.0	-0,48	0,18
Znf598	Q80YR4-2	YLDNDELLK	Y(1): 100.0	-0,48	0,48
Map1a	Q9QYR6	MASPPPSGPPSAAHTPFHQSPVEEK	S(3): 11.2; S(7): 11.2; S(11): 77.5; T(15): 0.0; S(20): 0.0	-0,48	0,22
Fam131b	Q3TY60-2	KVSDVTSSGVQSFDEEEGDANN	S(3): 0.0; T(6): 0.0; S(7): 0.0; S(8): 0.0; S(12): 100.0	-0,48	0,72
Nfia	Q02780-2	LKSVEDEMDSPGEEPFYTGQGR	S(3): 100.0; S(10): 0.0; Y(17): 0.0; T(18): 0.0	-0,48	-0,32
Skiv2l	Q6NZR5	LLEPLDLSGGDEDEGEAAGGPR	S(8): 100.0	-0,49	-0,08
Pbxip1	Q3TVI8	GREPSSSQPVVPVDVEDQAK	S(5): 91.8; S(6): 7.6; S(7): 0.7	-0,49	0,33
Atp1a1	Q8VDN2	VDNSSLTGESEPQTRSPDFTNENPLETR	S(4): 0.0; S(5): 0.0; T(7): 0.0; S(10): 0.0; T(14): 0.1; S(16): 99.7; T(20): 0.1; T(27): 0.0	-0,49	0,85
Cnksr3	Q8BMA3	KGSESPNSFLDQESQR	S(3): 0.0; S(5): 100.0; S(8): 0.0; S(14): 0.0	-0,49	0,15
Pds5b	Q4VA53-3	LDSSEMDHSENEDYTMSSPLPGK	S(3): 0.1; S(4): 0.7; S(9): 99.2; Y(14): 0.0; T(15): 0.0; S(17): 0.0; S(18): 0.0	-0,50	0,12

Disease Models & Mechanisms • Supplementary information

Lurap1	Q9D6I9	GSWDSLPDTSSTDR	S(2): 0.2; S(5): 99.7; T(9): 0.0; S(10): 0.0; S(11): 0.0; T(12): 0.0	-0,50	0,43
Gtpbp1	O08582	SRSPVDSPVPASMFAPEPSSPGAAR	S(1): 1.0; S(3): 99.0; S(7): 0.0; S(12): 0.0; S(19): 0.0; S(20): 0.0	-0,50	0,78
Ppp1r1b	Q60829	AVQHLQTISNLSENQASEEEDELGELR	T(7): 0.0; S(9): 0.0; S(12): 0.0; S(17): 100.0	-0,51	0,27
Ywhaz	P63101	TAFDEAIAELDTLSEESYK	T(1): 0.0; T(12): 0.1; S(14): 99.7; S(17): 0.1; Y(18): 0.1	-0,51	0,55
Sptbn1	Q62261	AQTLPTSVVTITSESSPGKR	T(3): 0.0; T(6): 0.0; S(7): 0.0; T(10): 0.0; T(12): 0.2; S(13): 33.3; S(15): 33.3; S(16): 33.3	-0,51	0,39
Srgap1	Q91Z69	RPGHGSLTNISR	S(6): 100.0; T(8): 0.0; S(11): 0.0	-0,52	-0,16
Psmd2	Q8VDM4	DKTPVQSQQPSATTPSGADEK	T(3): 0.0; S(7): 0.0; S(11): 0.0; T(13): 49.9; T(14): 49.9; S(16): 0.3	-0,52	0,24
Sp4	K4DI62	ENNVSQPASSSSSSSSSNNGSSSPTK	S(5): 0.0; S(9): 0.0; S(10): 0.0; S(11): 0.0; S(12): 0.0; S(13): 0.0; S(14): 0.0; S(15): 0.0; S(16): 0.0; S(17): 0.0; S(21): 0.4; S(22): 6.1; S(23): 87.3; T(25): 6.1	-0,53	0,53
Ppp1r12a	Q9DBR7	STGVSFWTQDSDENEQER	S(1): 0.0; T(2): 0.0; S(5): 0.0; T(8): 0.0; S(11): 100.0	-0,53	0,53
Map1b	P14873	SQGSTSNSDWMK	S(1): 0.0; S(4): 0.0; T(5): 0.1; S(6): 50.0; S(8): 50.0	-0,53	0,52
Sphkap	E9PUC4	NPHHTLNYDSNER	T(5): 1.4; Y(8): 97.2; S(10): 1.4	-0,53	0,23
Transmembrane protein C15orf27 homolog	Q8BZB3	ALDPAPLAQPTPLGSVQTSPELEHR	T(11): 0.0; S(15): 0.0; T(18): 50.0; S(19): 50.0	-0,54	0,00
Atn1	O35126	TEQELPRPQSPSDLDSLDGR	T(1): 0.0; S(10): 99.4; S(12): 0.6; S(16): 0.0	-0,54	0,16
Efr3a	Q8BG67	LTFYAVSAPEK	T(2): 0.3; Y(4): 99.7; S(7): 0.0	-0,55	0,44
Irf2bpl	Q8K3X4	NSSSPVSPASVPGQR	S(2): 0.0; S(3): 0.6; S(4): 99.3; S(7): 0.1; S(10): 0.0	-0,55	0,46
Phkb	Q7TSH2	QSSTADAPEAQHEPGITITEWK	S(2): 47.7; S(3): 47.7; T(4): 4.7; T(17): 0.0; T(19): 0.0	-0,55	0,17
Serpinb6a	F8WIV2	LTIMDPLQEANGTFALNLLK	T(2): 50.0; T(13): 50.0	-0,55	0,21
Svop	Q8BFT9	TGESARSEDDAASGEHDIQIEGVR	T(1): 1.6; S(4): 49.2; S(7): 49.2; S(13): 0.1	-0,56	0,44
Zswim8	Q3UHH1-3	KQSAGPNSPTGGGGGGGGSGGTR	S(3): 0.0; S(8): 99.8; T(10): 0.2; S(18): 0.0; T(21): 0.0	-0,56	0,92
Vdac1	Q8K284-2	VAASPRPGAEEQAEAQAPAQLAAPEDADAGGPR	S(4): 100.0	-0,57	0,06
Gramd1b	Q80TI0	SRSPTPQNQDGDTMVEK	S(1): 0.0; S(3): 99.5; T(5): 0.5; T(13): 0.0	-0,57	0,05
Slc4a4	E9Q8N8	MFSNPDNGSPAMTHR	S(3): 0.0; S(9): 99.8; T(13): 0.2	-0,57	0,38
Pcsk1n	Q9QXT0	NGESSELDLQGIR	S(4): 100.0; S(5): 0.0	-0,57	0,20
Slc6a11	P31650	GSETLGGGGGGAAGTR	S(2): 100.0; T(4): 0.0; T(15): 0.0	-0,58	0,82
Tom1	O88746	GLEFPMTDLDMLSPIHTPQR	T(7): 0.0; S(13): 100.0; T(17): 0.0	-0,59	-0,31
Tnrc6c	Q3UHC0	DSSEATGWEEPSPPSIR	S(2): 0.0; S(3): 0.0; T(6): 0.0; S(12): 99.7; S(15): 0.3	-0,59	0,33
Itih2	Q61703	SLPEESGEETDTVDPVTLYSYK	S(1): 1.1; S(6): 89.0; T(10): 9.8; T(12): 0.1; T(17): 0.0; Y(19): 0.0; S(20): 0.0; Y(21): 0.0	-0,59	0,09
Top2b	Q64511	KTSFDQDSDVDIFPSDFTSEPPALPR	T(2): 0.0; S(3): 0.0; S(8): 100.0; S(15): 0.0; T(18): 0.0; S(19): 0.0	-0,59	0,03
Golga1	Q9CW79-2	ESVASMGADSGDDFASDGSSSR	S(2): 0.0; S(5): 0.0; S(10): 100.0; S(16): 0.0; S(19): 0.0; S(20): 0.0; S(21): 0.0	-0,59	0,60
Rcor1	Q8CFE3	EREESEDELEETNGSNPVDIEIDPNK	S(5): 87.4; T(12): 12.6; S(15): 0.1	-0,60	0,09
Ctnnd2	E9QKH8	TGSQHGPQNAAAATFQR	T(1): 0.0; S(3): 100.0; T(14): 0.0	-0,60	0,15
Rtn3	Q9ES97-2	SEMCENSEQPQAQPETPTQK	S(1): 0.0; S(7): 0.0; T(16): 89.8; T(18): 10.2	-0,60	0,86
Akap12	Q9WTQ5	QAQSSTEIPLQAESGQGTEEEAAKDGEENR	S(4): 33.3; S(5): 33.3; T(6): 33.3; S(14): 0.0; T(18): 0.0	-0,60	0,31
Raly	Q64012-2	LPAPQEDTASEAGTPQGEVQTR	T(8): 0.0; S(10): 0.0; T(14): 100.0; T(21): 0.0	-0,61	-0,09
Bsn	O88737	EPELEMESLTGSPEDR	S(8): 4.7; T(10): 47.6; S(12): 47.6	-0,62	0,49
Ctbs	Q8R242	NIIDPTFRASWIAQK	T(6): 91.2; S(10): 8.8	-0,62	0,31
Pde4d	E9PVU7	EWYQSTIPQSPSPAPDDQEEGR	Y(3): 0.0; S(5): 0.0; T(6): 0.0; S(10): 99.6; S(12): 0.4	-0,62	0,40
Radil	Q69Z89-4	GTPALTSEAAQSSPPTR	T(2): 0.0; T(6): 0.0; S(7): 0.0; S(12): 4.9; S(13): 47.5; T(16): 47.5	-0,62	0,27
Camk4	Q8BGR3	TVCGTPGYCAPEILR	T(1): 99.9; T(5): 0.1; Y(8): 0.0	-0,64	0,34
Slc12a5	Q91V14	NKGPSPVSSEGIK	S(5): 100.0; S(8): 0.0; S(9): 0.0	-0,64	0,27
Stxbp1	O08599-2	ESSRVSFEDQAPTME	S(2): 0.0; S(3): 0.0; S(6): 99.9; T(13): 0.0	-0,64	0,67

Disease Models & Mechanisms • Supplementary information

Tmem200b	D3Z0P8	SLDLGLGELLLGAPATR	S(1): 100.0; T(16): 0.0	-0,65	-0,20
Jph3	Q9ET77	EFSPSFQHR	S(3): 100.0; S(5): 0.0	-0,66	0,26
Atf7ip	Q7TT18	NSSTTAAPLGTTLAVQAVPTAHSIVQATR	S(2): 4.7; S(3): 4.7; T(4): 4.7; T(5): 11.2; T(11): 29.8; T(12): 11.2; T(20): 11.2; S(23): 11.2; T(28): 11.2	-0,66	0,64
Setd1a	E9PYH6	SGSPAPETTNESVPFAQHSSLDSR	S(1): 0.0; S(3): 100.0; T(8): 0.0; T(9): 0.0; S(12): 0.0; S(19): 0.0; S(20): 0.0; S(23): 0.0	-0,67	0,46
Svop	Q8BFT9	TGESARSEDDAASGEHDIQIEGVR	T(1): 0.0; S(4): 50.0; S(7): 50.0; S(13): 0.0	-0,67	0,49
Myo18a	B2RRE2	EPADLDPEAASPAYSQAK	S(11): 74.6; Y(14): 12.7; S(15): 12.7	-0,67	0,17
Cacna1g	F6RJ39	APVVLQPEQIVSEEETPPPLLTK	S(12): 0.0; T(16): 100.0; T(22): 0.0	-0,67	0,61
Bysl	O54825	LGPGLPQDGSDEEDEEWPTLEK	S(10): 100.0; T(19): 0.0	-0,68	0,38
Vill	D3Z669	TVVSVFPGNNK	T(1): 93.3; S(4): 6.7	-0,68	0,05
Samd14	Q8K070	DASPPEPASPTIGLDK	S(3): 100.0; S(9): 0.0; T(11): 0.0	-0,69	0,68
Hspa4l	P48722	TGSEHNGPMDGQSGSETSPDPPK	T(1): 0.0; S(3): 0.0; S(13): 6.6; S(15): 6.6; T(17): 43.4; S(18): 43.4	-0,69	0,03
Dock4	P59764	ASPLLSDK	S(2): 100.0; S(6): 0.0	-0,73	0,59
Pds5b	Q4VA53-3	AESPETSAVESTQSTPQK	S(3): 98.9; T(6): 1.0; S(7): 0.1; S(11): 0.0; T(12): 0.0; S(14): 0.0; T(15): 0.0	-0,74	-0,05
Vps35	Q9EQH3	RESPESEGPIYEGLIL	S(3): 100.0; S(6): 0.0; Y(11): 0.0	-0,81	0,00
Brd3	Q8K2F0	QASASYDSEEEEEGLPMSYDEK	S(3): 0.5; S(5): 4.6; Y(6): 47.4; S(8): 47.4; S(18): 0.0; Y(19): 0.0	-0,84	-0,04
Nucks1	Q80XU3	KVVDYSQFQESDDADEDYGR	Y(5): 0.0; S(6): 0.0; S(11): 100.0; Y(18): 0.0	-0,91	0,20
Rgl3	Q3UYI5	NREPPPPGSPPASPGPQSPSTK	S(9): 100.0; S(13): 0.0; S(18): 0.0; S(20): 0.0; T(21): 0.0	-0,94	0,18
Ccdc40	Q8BI79-3	ILQQWTTSLVGMK	T(6): 33.3; T(7): 33.3; S(8): 33.3	-0,95	-0,20
Efr3b	Q6ZQ18	AGIVEVLSEAAIIAATGSVGPTVLEMFNTLLR	S(8): 9.3; T(16): 29.2; S(18): 29.2; T(22): 29.2; T(29): 3.2	-0,96	0,57
Mtss1	G3X9H7	GSDYSWSYQTPPSSPSTTMSR	S(2): 0.0; Y(4): 0.0; S(5): 0.0; S(7): 0.0; Y(8): 1.4; T(10): 10.0; S(13): 78.3; S(14): 10.0; S(16): 0.2; T(17): 0.0; T(18): 0.0; S(20): 0.0	-0,96	0,87
Apex1	P28352	ALGSDHCPITLYLAL	S(4): 93.8; T(10): 3.1; Y(12): 3.1	-1,00	0,45
Htt	P42859	SLNPQKSGEEEDSGSAAQLGMCNR	S(1): 0.2; S(7): 99.7; S(13): 0.1; S(15): 0.1	-1,18	0,68
Dennd1a	Q8K382	TMPSPQAETQDPR	T(1): 0.1; S(4): 99.9; T(9): 0.0	-1,29	-0,22
Arvcf	P98203-2	SLAADDEGGPDLEPDYSTATR	S(1): 100.0; Y(16): 0.0; S(17): 0.0; T(18): 0.0; T(20): 0.0	-1,31	0,64
Ptchd3	Q0EEE2	GFIQIVDVSSSSNK	S(9): 25.0; S(10): 25.0; S(11): 25.0; S(12): 25.0	-1,32	0,55
Prkcb	P68404-2	NIDQSEFEGFSFVNSEFLKPEVK	S(5): 0.0; S(11): 50.0; S(15): 50.0	-1,33	0,17
Tbx18	G3X919	IVSSQGSFLGSSPSGTMTDR	S(3): 0.1; S(4): 0.1; S(7): 2.3; S(11): 43.5; S(12): 43.5; S(14): 9.5; T(16): 0.6; T(18): 0.6	-1,35	0,95
Dos	E9QP85	LAEPVASSQVLIVAAAAPTSPDHSPA	S(7): 0.0; S(8): 0.0; T(19): 50.0; S(20): 50.0; S(24): 0.1	-1,37	1,51
Fam131b	Q3TY60-2	KVSDVTSSGVQSFDEEEGDANN	S(3): 100.0; T(6): 0.0; S(7): 0.0; S(8): 0.0; S(12): 0.0	-1,38	0,87
Atp7b	B1AQ57	AIATQVGINK	T(4): 100.0	-1,38	1,02
Mprip	P97434-2	DFASEAPTAPLSDACPLSPHR	S(4): 0.0; T(8): 0.0; S(12): 0.0; S(18): 100.0	-1,62	0,43
Atp1a4	Q9WV27	ATTGDASESALLK	T(2): 0.0; T(3): 0.0; S(7): 92.5; S(9): 7.5	-1,94	0,42

Disease Models & Mechanisms • Supplementary information

Supplementary Table S7: Common proteins up and down phosphorylated in hippocampus of Tg(*Dyrk1a*) and Ts65Dn mice, and reverted by L41 treatment.

Gene name	Protein Group Accessions	Sequence	phosphoRS Site Probabilities	Ts65Dn vs WT	Ts65Dn +/- L41	TgDyrk1A vs WT	TgDyrk1A +/-L41
Aak1	Q3UHJ0-2	ILSDVTHSAVFGVPASK	S(3): 0.4; T(6): 93.9; S(8): 5.7; S(16): 0.0	0,59	-0,02	0,59	-0,63
Aak1	Q3UHJ0-2	VQTTPPPTIQGQK	T(3): 0.0; T(4): 100.0; T(8): 0.0	-0,70	0,27	0,42	-0,18
Ace	P09470	GPQFGSEVELR	S(6): 100.0	0,65	-0,44	-0,67	0,17
Aldoa	P05064	GILAADESTGSIAK	S(8): 0.3; T(9): 5.5; S(11): 94.2	0,47	-0,01	-0,73	0,34
Arhgef2	H3BJU7	LQDSSDPDTGSEEEVSSRLSPPHSPR	S(4): 14.3; S(5): 14.3; T(9): 14.3; S(11): 14.3; S(16): 14.3; S(17): 14.3; S(20): 14.3; S(24): 0.0	-0,38	1,25	-1,40	-0,59
Arhgef2	H3BJU7	LSPPHSPR	S(2): 100.0; S(6): 0.0	1,13	-1,17	0,46	-0,59
Arhgef28	G5E8P2	LSLPMAAGHGGDAGDGAEENILYL	S(2): 96.9; Y(23): 3.1	0,64	-0,23	-0,51	0,37
Basp1	Q91XV3	AEGAGTEEEGTPKESEPQAAADATEVK	T(6): 0.0; T(11): 100.0; S(15): 0.0; T(24): 0.0	-0,56	0,36	-0,29	0,83
Basp1	Q91XV3	AEGAGTEEEGTPKESEPQAAADATEVK	T(6): 1.4; T(11): 97.2; S(15): 1.4; T(24): 0.0	0,11	0,04	-0,63	0,57
Basp1	Q91XV3	ETPAASEAPSSAAK	T(2): 0.0; S(6): 0.0; S(10): 5.8; S(11): 94.2	-1,05	0,68	-0,86	0,60
Basp1	Q91XV3	KAEGAGTEEEGTPK	T(7): 0.0; T(12): 100.0	-0,73	0,36	-0,53	0,20
Basp1	Q91XV3	KAEGAGTEEEGTPKESEPQAAADATEVK	T(7): 0.3; T(12): 86.6; S(16): 13.1; T(25): 0.0	-0,85	0,73	-0,13	0,88
Basp1	Q91XV3	KAEGAGTEEEGTPKESEPQAAADATEVK	T(7): 0.1; T(12): 49.9; S(16): 49.9; T(25): 0.0	0,08	-0,18	-1,30	1,22
Basp1	Q91XV3	SDAAPAASDSKPSSAEPAPSSK	S(1): 0.0; S(8): 98.8; S(10): 0.6; S(13): 0.6; S(14): 0.0; S(20): 0.0; S(21): 0.0	0,31	-0,02	-0,48	0,54
Bcmo1	E9Q321	YLQSDTYIANIEANR	Y(1): 0.0; S(4): 0.1; T(6): 7.2; Y(7): 92.8	-0,93	0,73	0,56	-0,28
Bsn	O88737	DACEPESGPDPSTVR	S(7): 100.0; S(12): 0.0; T(13): 0.0	-0,95	0,44	0,02	0,13
Bsn	O88737	DYLSDSELNQLR	Y(2): 0.0; S(4): 100.0; S(6): 0.0	-0,31	0,03	-0,25	0,47
Bsn	O88737	DYLSDSELNQLR	Y(2): 0.0; S(4): 99.9; S(6): 0.0	-0,94	0,54	-0,30	0,53
Bsn	O88737	EPELEMESLTGSPEDR	S(8): 0.0; T(10): 93.4; S(12): 6.5	-1,09	0,05	0,05	-0,18
Bsn	O88737	HDYVEDSSEGGLSPLPPQPPAR	Y(3): 0.8; S(7): 91.2; S(8): 8.0; S(13): 0.0	-0,30	0,50	-0,25	-0,68
Bsn	O88737	SPQVLYSPVSPLSPHR	S(1): 0.0; S(7): 0.0; S(10): 0.2; S(13): 99.8	0,67	-0,19	-0,27	0,30
Bsn	O88737	SQGSFEYQDTQDHDYGGR	S(1): 0.0; S(4): 100.0; Y(7): 0.0; T(10): 0.0; Y(15): 0.0	-0,64	0,31	-0,20	-0,06
Bsn	O88737	TLPSPPPEEAHLPLAGQVPSQLYAASLLQR	T(1): 0.0; S(4): 13.3; S(20): 86.4; Y(23): 0.4; S(26): 0.0	-0,19	0,32	-0,55	-0,27
C2cd2l	Q80X80	EAGLSQSHDDLSNTTATPSVR		-0,23	0,00	-0,53	0,29
C2cd2l	Q80X80	SLSPAATVTAELHYEQGSPR	S(1): 0.0; S(3): 0.0; T(7): 0.0; T(9): 0.0; Y(14): 0.0; S(18): 100.0	1,03	-0,82	-0,46	0,59
Cacna1a	P97445	EMGTDGYSDSEHYLPMEGQTR	T(4): 24.9; Y(7): 24.9; S(8): 24.9; S(10): 24.9; Y(13): 0.3; T(20): 0.0	-0,68	0,27	0,27	-0,23
Cacna1a	P97445	EMGTDGYSDSEHYLPMEGQTR	T(4): 0.0; Y(7): 49.9; S(8): 49.9; S(10): 0.3; Y(13): 0.0; T(20): 0.0	0,03	0,08	-0,49	0,21
Camkv	Q3UHL1	ATPATEESTVPATQSSALPAAK	T(2): 98.0; T(5): 1.9; S(8): 0.1; T(9): 0.1; T(13): 0.0; S(15): 0.0; S(16): 0.0	-2,18	1,91	-0,29	-0,32
Camkv	Q3UHL1	SATPATDGRATPATEESTVPATQSSALPAAK	S(1): 2.4; T(3): 2.4; T(6): 2.4; T(11): 44.8; T(14): 44.8; S(17): 2.4; T(18): 0.6; T(22): 0.0; S(24): 0.0; S(25): 0.0	-1,97	1,17	0,85	-0,26
Camkv	Q3UHL1	SATPATDGRATPATEESTVPATQSSALPAAK	S(1): 0.0; T(3): 0.0; T(6): 0.0; T(11): 98.3; T(14): 1.7; S(17): 0.0; T(18): 0.0; T(22): 0.0; S(24): 0.0; S(25): 0.0	-1,81	1,54	0,41	0,07
Cep170b	Q80U49	ESPLSPPTVPDPGGATPGSAR	S(2): 0.6; S(5): 99.4; T(8): 0.0; T(16): 0.0; S(19): 0.0	-0,32	0,24	-0,46	0,36
Cep170b	Q80U49	GSLDWPEEER	S(2): 100.0	-0,58	0,00	-0,63	0,29
Cep170b	Q80U49	HEDGTQSDSEDPLAK	T(5): 33.3; S(7): 33.3; S(9): 33.3	0,69	-1,01	1,63	-0,59
Cep170b	Q80U49	HEDGTQSDSEDPLAK	T(5): 0.8; S(7): 98.5; S(9): 0.8	0,67	-0,88	1,37	-0,75
Cep170b	Q80U49	QESFTKEPTSGPPAPGK	S(3): 93.6; T(5): 6.4; T(9): 0.0; S(10): 0.0	-0,81	0,27	-0,75	0,63
Chgb	P16014	ADEFPDFYDSEEQMGPHQEANDEK	Y(8): 1.0; S(10): 99.0	-1,03	0,47	-0,39	0,74
Chgb	P16014	EGVDDQESLRPSNQQASK		-0,77	-0,26	-0,82	-0,01
Chgb	P16014	ESKEADVATVR	S(2): 100.0; T(9): 0.0	-1,18	0,45	0,19	0,05
Chgb	P16014	SSYEGHPLSEER	S(1): 0.0; S(2): 0.0; Y(3): 0.0; S(9): 100.0	-0,69	0,62	0,06	0,01
Chgb	P16014	WWQQEEQLGPEESREEVR	S(13): 100.0	-1,06	0,48	0,40	-0,09

Disease Models & Mechanisms • Supplementary information

Clip2	Q9Z0H8	QASGPSSSGAATTVSEKPGPK	S(3): 82.9; S(6): 8.5; S(7): 8.5; S(8): 0.1; T(12): 0.0; T(13): 0.0; S(15): 0.0	-0,95	0,81	-0,39	0,35
Clip2	Q9Z0H8	TGNESGSNLSDSGSVK	S(5): 0.0; S(7): 91.1; S(10): 8.9; S(12): 0.0; S(14): 0.0	2,08	-3,05	-0,69	-0,06
Clns1a	Q923F1	LGEESKEPLSDEDEEDNDDVEPISEFR	S(5): 50.0; S(10): 50.0; S(24): 0.0	-0,77	0,73	0,56	-0,28
Col3a1	P08121	GENGSPGAPGAPGHPGPPGPVGPSGK	S(5): 100.0; S(24): 0.0	-0,64	-0,40	-0,33	0,32
Col3a1	P08121	GFPGNPGPPGSPGAAGHQGAIGSPGPAGPR	S(11): 50.0; S(23): 50.0	-1,52	0,06	0,33	0,60
Col3a1	P08121	GPAGPPGPPGASGSPGLQGMPGER	S(12): 85.8; S(14): 14.2	0,00	-0,40	0,41	-0,92
Col3a1	P08121	GPAGPPGPPGASGSPGLQGMPGER	S(12): 50.0; S(14): 50.0	-0,64	0,59	-0,55	-0,15
Col4a1	P02463	GFPGPPGPDGLPGSMGPPGTPSVDHGFLVTR	S(14): 98.9; T(20): 1.1; S(22): 0.0; T(30): 0.0	0,96	-0,64	0,58	-0,29
D430041D05Rik	D3YZ21	GHSETSTLSSQPSIDEVR	S(3): 0.5; T(5): 97.7; S(6): 0.5; T(7): 0.5; S(9): 0.5; S(10): 0.5; S(13): 0.0	-0,76	0,71	0,61	-0,26
Dmtn	Q9WV69	DSSVPGSPSSIVAK	S(2): 0.0; S(3): 0.0; S(7): 87.7; S(9): 6.1; S(10): 6.1	-0,74	0,13	-0,12	0,04
Dmtn	Q9WV69	ESVGGSPQSK		-0,59	0,11	-0,58	0,41
Dmtn	Q9WV69	KGAEEEEEEEDDDSEEEIK	S(14): 100.0	0,62	-1,21	-0,64	0,90
Dmtn	Q9WV69	LQSTEFSPSGSEAGSPGLQNGEGQR	S(3): 0.1; T(4): 0.8; S(7): 99.1; S(9): 0.1; S(11): 0.0; S(15): 0.0	0,39	-0,27	0,43	-0,05
Dmtn	Q9WV69	QRESVGGSPQSK	S(4): 100.0; S(8): 0.0; S(11): 0.0	-0,45	0,08	-0,15	0,45
Dnah7b	L7N1Y0	SSDEVVNEVAGDILSK	S(1): 7.5; S(2): 92.5; S(15): 0.0	-0,94	-0,22	-0,67	-0,02
Eif2ak4	A2AUM0	EILDGLAYIHEK	Y(8): 100.0	2,74	-2,62	0,93	0,13
Fam169a	Q5XG69	GHTEENLSPVSK		-0,77	0,19	-0,27	-0,33
Fam169a	Q5XG69	HFQDSEFSSSQGEDENVAK	S(5): 0.0; S(8): 6.8; S(9): 86.3; S(10): 6.8	0,25	0,50	0,35	-0,71
Fam169a	Q5XG69	SQSEEQSEASSEHLEQFTQSAEK	S(1): 0.0; S(3): 0.0; S(7): 0.0; S(10): 33.2; S(11): 33.2; T(18): 33.2; S(20): 0.3	-0,59	0,15	-0,44	0,00
Fam171a2	A2A699	SVEGPGGLEPSLDEYR	S(1): 100.0; S(11): 0.0; Y(15): 0.0	0,60	-0,15	-0,75	0,71
Gbf1	Q6A099	ADAPDAGAQSDSELPSYHQNDVSLDR	S(10): 49.7; S(12): 49.7; S(16): 0.3; Y(17): 0.3; S(23): 0.0	0,89	-0,60	1,08	-0,40
Gm14597	B1AXE0	VLAFVEEDPLSHELK		0,55	-0,69	-0,54	-0,19
Gpr123	Q8C4G9	ALPCPGADQPPYPK	Y(12): 100.0	0,45	0,22	-0,96	-0,34
Grin2b	G3X9V4	QHSYDTFVDLQK	S(3): 2.9; Y(4): 97.1; T(6): 0.0	-0,25	0,02	0,47	-0,09
Grin2b	G3X9V4	RQHSYDTFVDLQK	S(4): 48.4; Y(5): 48.4; T(7): 3.2	0,88	-0,69	0,06	-0,02
Habp4	Q9JKS5	SLAASGAQQPDSPGGPQPPGQK	S(1): 0.0; S(5): 0.0; S(12): 100.0	-0,69	0,74	0,50	-0,63
Hcn2	O88703	RAPPGPLPPAASPGPPAASPPAAPSSPR	S(12): 0.0; S(19): 100.0; S(25): 0.0; S(26): 0.0	-0,20	0,21	-0,77	0,46
Hcn2	O88703	RDSASPGAASGLDPLDSAR	S(3): 8.9; S(5): 91.1; S(10): 0.0; S(17): 0.0	0,58	-0,19	-0,32	-0,16
Hepacam	Q640R3	STTEPGPPGYSVSPPVPGRSPGLPIR	S(1): 0.0; S(11): 0.0; S(13): 0.0; S(20): 100.0	0,49	-0,07	0,65	-0,36
Hn1	P97825	SNSSEASSGDFLDLK	S(1): 0.0; S(3): 50.0; S(4): 50.0; S(7): 0.0; S(8): 0.0	0,92	-0,66	0,46	-0,03
Hn1	P97825	SNSSEASSGDFLDLK	S(1): 33.3; S(3): 33.3; S(4): 33.3; S(7): 0.0; S(8): 0.0	-0,70	0,52	0,20	-0,17
Hnrnpa0	Q9CX86	LFIGGLNVQTSESGLR		0,44	-0,25	-0,69	0,20
Huwe1	Q7TMY8	DLSMSEEDQMMR	S(3): 91.9; S(5): 8.1	-0,77	0,74	0,89	-0,71
Krt26	Q3TRJ4	EALSEELTYLQK	S(4): 0.0; T(8): 50.0; Y(9): 50.0	0,39	0,20	-0,70	0,72
Lmna	P48678	ASSHSSQSQGGGSVTK	S(2): 6.8; S(3): 6.8; S(5): 0.6; S(6): 85.1; S(8): 0.6; S(13): 0.0	-0,64	0,14	-0,94	0,00
Lmna	P48678	LRLSPSPTSQR		0,58	-0,14	-0,39	-0,31
Map1a	Q9QYR6	AELEEMEEVHPSDEEEEETKAESFYQK	S(12): 33.3; T(19): 33.3; S(23): 33.3; Y(25): 0.2	-0,84	0,27	-0,72	0,55
Map1a	Q9QYR6	APISLSQDPSPPLNGSTTSCGPDR	S(4): 0.0; S(6): 0.0; S(10): 100.0; S(16): 0.0; T(17): 0.0; T(18): 0.0; S(19): 0.0	-0,90	0,49	0,13	-0,30
Map1a	Q9QYR6	GEPVPAWEGKSPEQEVR	S(11): 100.0	-0,79	0,40	-0,08	0,02
Map1a	Q9QYR6	GFKSPPCEDFSVTGESEK	S(4): 100.0; S(11): 0.0; S(16): 0.0	-0,15	-0,66	-0,59	0,18
Map1a	Q9QYR6	SEPQDFQEDSWGDTK	S(1): 0.0; S(10): 5.4; T(14): 94.6	-0,98	0,56	0,47	-0,05
Map1a	Q9QYR6	SHWDDGTNDSDLEK	S(1): 0.0; T(7): 0.0; S(10): 100.0	-0,71	0,78	0,48	-0,31
Map1a	Q9QYR6	SHWDDGTNDSDLEK	S(1): 0.0; T(7): 0.0; S(10): 100.0	-0,72	0,28	0,09	-0,05
Map1a	Q9QYR6	SPESLSSPAMEDLAMEWGGK	S(1): 0.0; S(4): 0.3; S(6): 5.0; S(7): 94.7	0,72	-0,03	0,33	-0,59
Map1a	Q9QYR6	SPFEIISPPASPPEMTGQR	S(1): 0.0; S(7): 100.0; S(11): 0.0; T(16): 0.0	-0,74	0,02	0,33	-0,30
Map1a	Q9QYR6	SPPCEDFSVTGESEK	S(1): 100.0; S(8): 0.0; T(10): 0.0; S(13): 0.0	-0,41	0,15	0,71	-0,21
Map1a	Q9QYR6	TEATQGLDYVPSAGTISPTSSLEEDKGFK	T(1): 0.0; T(4): 0.0; Y(9): 0.0; S(12): 0.0; T(15): 0.2; S(17): 24.9; T(19): 24.9; S(20): 24.9; S(21): 24.9	-0,99	0,89	0,30	0,45

Map1a	Q9QYR6	VPLSRSPQAQDTLGSLAGGQTGCTIQLLPEQDK	S(4): 47.2; S(6): 47.2; T(12): 2.3; S(15): 0.6; T(21): 2.3; T(24): 0.6	0,24	0,33	0,91	-0,56
Map1a	Q9QYR6	VPSAPGQESPVPDTK	S(3): 0.4; S(9): 99.6; T(14): 0.0	-0,71	0,82	0,32	0,01
Map1a	Q9QYR6	WLAESPVGLPPEEEDK	S(5): 100.0	-0,08	-0,09	-0,45	-0,58
Map1b	P14873	ASLSPMDEPVPDSESPVEK	S(2): 0.0; S(4): 0.0; S(13): 0.0; S(15): 100.0	-0,18	0,40	0,82	-0,09
Map1b	P14873	ASLSPMDEPVPDSESPVEK	S(2): 0.0; S(4): 0.0; S(13): 0.0; S(15): 100.0	-1,00	0,29	-0,19	0,17
Map1b	P14873	DLTTSSVEK	T(3): 0.0; T(4): 5.6; S(5): 88.8; S(6): 5.6	-0,37	0,04	3,35	-2,96
Map1b	P14873	DYNASASTISPPSSMEEDK	Y(2): 0.0; S(5): 0.0; S(7): 0.0; T(8): 0.0; S(10): 100.0; S(13): 0.0; S(14): 0.0	-0,58	0,11	0,24	-0,59
Map1b	P14873	DYNASASTISPPSSMEEDK	Y(2): 0.0; S(5): 0.0; S(7): 0.0; T(8): 0.0; S(10): 100.0; S(13): 0.0; S(14): 0.0	-0,34	-0,44	0,06	0,44
Map1b	P14873	DYNASASTISPPSSMEEDK	Y(2): 0.0; S(5): 0.0; S(7): 0.0; T(8): 0.0; S(10): 99.6; S(13): 0.2; S(14): 0.2	-0,54	0,33	-0,61	-0,25
Map1b	P14873	DYNASASTISPPSSMEEDKFSK	Y(2): 0.0; S(5): 0.3; S(7): 11.2; T(8): 11.2; S(10): 77.2; S(13): 0.0; S(14): 0.0; S(21): 0.0	-0,14	-0,57	-0,04	-0,45
Map1b	P14873	EEQSPVKAEVAEK	S(4): 100.0	-0,04	-0,08	-0,65	-0,10
Map1b	P14873	ETAAAHQASSSPPIDAATAEPYGFR	T(2): 0.0; S(9): 33.3; S(10): 33.3; S(11): 33.3; T(18): 0.0; Y(22): 0.0	-0,28	0,04	0,33	-0,74
Map1b	P14873	KLGGDVSPTQIDVSQFGSFK	S(7): 99.9; T(9): 0.1; S(14): 0.0; S(18): 0.0	-0,12	0,42	-0,37	0,58
Map1b	P14873	SEQSSMSIEFGQESPEHSLAMDFSR	S(1): 0.0; S(4): 0.0; S(5): 0.4; S(7): 49.6; S(14): 49.6; S(18): 0.4; S(24): 0.0	1,06	-0,17	0,20	-0,04
Map1b	P14873	SQGSTSNSDWMK	S(1): 0.0; S(4): 93.3; T(5): 5.9; S(6): 0.4; S(8): 0.4	0,34	0,13	-0,30	-0,17
Map1b	P14873	SVSPGVTQAVVEEHCASPEEK	S(1): 0.0; S(3): 0.0; T(7): 0.0; S(17): 100.0	-0,35	0,18	0,09	-1,19
Map1b	P14873	TPEEGGYSYEISEK	T(1): 100.0; Y(7): 0.0; S(8): 0.0; Y(9): 0.0; S(12): 0.0	-0,74	0,61	-0,03	0,11
Map1b	P14873	TTRSPDTSAYCYETMEK	T(1): 0.2; T(2): 0.2; S(4): 99.5; T(7): 0.0; S(8): 0.0; Y(10): 0.0; Y(12): 0.0; T(14): 0.0	0,55	-0,56	0,05	0,40
Map2	P20357	GSAQESLDTISPKNQHDEK	S(2): 0.0; S(6): 0.3; T(9): 49.8; S(11): 49.8	-0,78	-0,09	-0,26	0,24
Map2	P20357	LASVSADAEVAR	S(3): 100.0; S(5): 0.0	-1,56	0,77	0,98	-0,50
Map2	P20357	STELGSDYYELSDSR	S(1): 0.0; T(2): 0.0; S(6): 0.0; Y(8): 0.0; Y(9): 0.0; S(12): 0.0; S(14): 100.0	-0,64	0,72	-0,47	0,25
Map2	P20357	VGSLDNAHHVPGGGNVK	S(3): 100.0	-0,46	0,33	-0,44	0,08
Marcksl1	P28667	AAATPESQEPQAK	T(4): 50.0; S(7): 50.0	-0,83	0,60	-0,56	0,32
Marcksl1	P28667	AAATPESQEPQAK	T(4): 99.5; S(7): 0.5	-0,58	0,55	-0,67	0,41
Marcksl1	P28667	AAATPESQEPQAK	T(4): 50.0; S(7): 50.0	-0,83	0,60	-0,56	0,32
Marcksl1	P28667	AAATPESQEPQAK	T(4): 99.5; S(7): 0.5	-0,58	0,55	-0,67	0,41
Marcksl1	P28667	GDVTAEEAAGASPAK	T(4): 0.0; S(12): 100.0	-0,83	0,21	-0,77	0,41
Marcksl1	P28667	GDVTAEEAAGASPAK	T(4): 0.0; S(12): 100.0	-0,83	0,21	-0,77	0,41
Mccc2	Q3ULD5	AYHGDSVARLGTQPDSASSTYQENYEQMK	S(6): 0.0; S(16): 9.7; S(18): 45.1; S(19): 45.1	-1,27	0,70	-0,85	0,47
Me1	P06801	LFYSVLMSDVEK	Y(3): 47.9; S(4): 47.9; S(8): 4.3	-1,09	0,45	0,76	-0,07
Mtdh	Q80WJ7	SETNWESPK	S(1): 0.0; S(7): 100.0	0,41	0,03	0,50	-0,39
Mug2	P28666	FDLAFSSEVSGTLQK	S(6): 49.8; S(7): 49.8; S(10): 0.3; T(12): 0.0	0,34	-0,02	1,23	-0,46
Myo18a	B2RRE2	EPADLDPEAASPAYSQAK	S(11): 100.0; Y(14): 0.0; S(15): 0.0	-1,03	0,39	1,09	-0,22
Nap1l4	Q78ZA7	ASGDGESLDEDSEFTLASDFEIGHFFR	S(2): 0.0; S(7): 100.0; S(12): 0.0; T(15): 0.0; S(18): 0.0	0,55	-0,34	-0,81	0,49
Nap1l4	Q78ZA7	EFITGDVEPTDAESAWHSENEEEDK	T(4): 0.0; T(10): 0.2; S(14): 1.3; S(18): 98.6	-0,29	-0,03	-0,52	0,39
Ncl	P09405	GLSEDTTEETLK	S(3): 100.0; T(6): 0.0; T(7): 0.0; T(10): 0.0	-0,73	-0,30	0,16	-0,07
Ncl	P09405	NLSFNITEDELK	S(3): 100.0; T(7): 0.0	-0,42	0,50	0,78	-0,52
Nefh	P19246	SPAAVKSPAEAK	S(1): 0.0; S(7): 100.0	-0,38	-0,09	0,46	-0,04
Nefh	P19246	SPAEAKSPAEAK	S(1): 0.0; S(7): 100.0	-0,66	-0,08	0,52	0,52
Nefh	P19246	SPAEPKSPAEAK	S(1): 0.0; S(7): 100.0	-0,38	-0,12	0,71	-0,03
Nefh	P19246	SPGEAKSPSEAK	S(1): 0.0; S(7): 99.6; S(9): 0.4	-0,78	0,02	0,40	0,34
Nefm	P08553	QASHAQLGDAYDQEIR	S(3): 100.0; Y(11): 0.0	-0,77	0,32	0,51	0,03
Nktr	P30415	SRASSSSSHHSSK	S(1): 0.0; S(4): 0.0; S(5): 0.0; S(6): 0.0; S(7): 1.2; S(8): 1.2; S(11): 87.9; S(12): 9.7	0,64	-0,64	-0,43	1,35
Npnt	D3YTX1	VTGLHSGTLQVFVRK	T(2): 50.0; S(6): 50.0; T(8): 0.1	-0,71	0,45	-0,43	0,41

Disease Models & Mechanisms • Supplementary information

Nsmf	A2AJ96	MYSVDGVSDDVPIR	Y(2): 0.0; S(3): 100.0; S(8): 0.0	0,48	0,23	-0,72	0,29
Nudt3	Q9JI46	SESEEEVLLVSSSR	S(1): 0.0; S(3): 100.0; S(11): 0.0; S(12): 0.0; S(13): 0.0	0,65	0,34	-0,70	0,61
Pacsin1	Q61644	GRLDSGQLGLYPANYVEAI	S(5): 100.0; Y(11): 0.0; Y(15): 0.0	0,35	0,07	0,57	-0,05
Pacsin1	Q61644	KAEGATLSNATGAVESTSQAGDR	T(6): 0.0; S(8): 0.0; T(11): 0.0; S(16): 6.2; T(17): 46.9; S(18): 46.9	0,08	-0,21	0,29	-0,11
Pacsin1	Q61644	KAEGATLSNATGAVESTSQAGDRGSVSSYDR	T(6): 0.0; S(8): 0.0; T(11): 0.1; S(16): 78.2; T(17): 16.7; S(18): 3.8; S(25): 0.9; S(27): 0.2; S(28): 0.1; Y(29): 0.1	-0,87	0,22	-0,06	0,63
Pacsin1	Q61644	KAEGATLSNATGAVESTSQAGDRGSVSSYDR	T(6): 0.0; S(8): 0.0; T(11): 0.0; S(16): 2.9; T(17): 2.9; S(18): 2.9; S(25): 87.7; S(27): 2.9; S(28): 0.6; Y(29): 0.1	-0,77	1,17	-0,53	0,28
Pacsin1	Q61644	STSGPGMPMNWPQFEEWNPDLPHTTAK	S(1): 0.0; T(2): 0.0; S(3): 1.1; T(24): 97.9; T(25): 1.1	0,36	-0,18	-0,61	0,41
Pak3	Q61036-2	IGQGASGTVYTALDIATGQEVAIK	S(6): 0.0; T(8): 0.0; Y(10): 0.0; T(11): 0.0; T(17): 100.0	-0,90	0,29	1,31	-0,19
Palmd	Q9JHU2	RSNGPTHTSPTRPTPQPR	S(2): 0.0; T(6): 0.0; T(8): 50.0; S(9): 50.0; T(11): 0.0; T(14): 0.0	-0,85	-0,07	-0,55	0,70
Palmd	Q9JHU2	SGPQCSSPTCQEETEDVR	S(1): 0.0; S(6): 50.0; S(7): 50.0; T(9): 0.0; T(14): 0.0	-0,66	0,49	-0,64	0,37
Palmd	Q9JHU2	SNGPTHTSPTRPTPQPR	S(1): 0.0; T(5): 0.3; T(7): 49.7; S(8): 49.7; T(10): 0.3; T(13): 0.0	1,15	-1,30	0,15	0,00
Pcdh11x	B1AZR7	GDSPIIEEHPL	S(3): 100.0	-0,79	0,14	1,31	-0,93
Pclo	Q9QYX7	ENPELVDDLSPR	S(10): 100.0	-0,73	0,37	-0,17	-0,07
Pclo	Q9QYX7	GSVPAAAAESPSMHR	S(2): 0.0; S(10): 94.2; S(12): 5.8	-0,35	0,28	0,79	-0,74
Pclo	Q9QYX7	KDSFSQESSPSSPSDLAK	S(3): 0.0; S(5): 0.0; S(8): 0.0; S(9): 0.0; S(11): 94.1; S(12): 5.9; S(14): 0.0	-0,17	0,22	-0,19	1,16
Pclo	Q9QYX7	RSQEVTDFLAPLQTSSR	S(2): 100.0; S(15): 0.0; S(16): 0.0	-0,12	0,53	-1,02	0,58
Pclo	Q9QYX7	TIELNSTVTDK	S(6): 100.0	0,40	0,37	0,27	0,06
Pclo	Q9QYX7	TSIGSSSSEEYKQEDSQGSGEDEDFIR	T(1): 0.0; S(2): 0.0; S(5): 0.0; S(6): 0.0; S(7): 0.0; S(8): 0.0; Y(11): 0.0; S(16): 0.7; S(19): 99.3	-0,80	0,44	-0,14	0,18
Pp2d1	Q8BVT6	ECIISNLLR	S(5): 100.0	-1,02	0,91	1,62	-0,97
Ppp1r18	Q8BQ30	EEAEAQSRPLR		0,56	-0,38	-0,90	0,41
Ppp6r3	Q922D4-2	IQQFDDGGSDEEDIWEEK	S(9): 100.0	-0,87	0,90	0,71	-0,13
Prkcb	P68404-2	NIDQSEFEGFSFVNSEFLKPEVK	S(5): 0.5; S(11): 49.8; S(15): 49.8	-1,31	0,23	1,24	-1,29
Prkcb	P68404	QPVELTPTDK	T(6): 100.0; T(8): 0.0	0,68	-1,25	-0,02	0,13
Prrc2c	Q3TLH4-5	AFGSGIDIKPGTPPIGGR		-0,18	0,20	-0,50	0,49
Prrc2c	Q3TLH4-5	ATSTSPNSQSSK	T(2): 0.0; S(3): 0.0; T(4): 4.6; S(5): 95.4; S(8): 0.0; S(10): 0.0; S(11): 0.0	0,76	-1,08	-0,40	0,18
Prrt2	E9PUL5	ASPSPSPSEAR	S(2): 99.3; S(4): 0.1; S(6): 0.7; S(8): 0.0	-0,81	0,50	-0,71	0,37
Prrt2	E9PUL5	QENGAVVPLQAGDGEEGPAPQPHSPPSTK	S(24): 100.0; S(27): 0.0; T(28): 0.0	0,18	0,18	-0,44	0,20
Rab3a	P63011	MSESLDTADPAVTGAK	S(2): 100.0; S(4): 0.0; T(7): 0.0; T(13): 0.0	0,34	0,06	-0,49	-0,26
Rbbp8	Q80YR6	MDVTVIDTK	T(4): 100.0; T(8): 0.0	-0,75	0,47	0,46	-0,72
Reps2	A2AFI8	APSQAAESSPTK	S(3): 0.0; S(8): 0.3; S(9): 99.7	-0,81	0,05	-1,14	0,51
Reps2	A2AFI8	SFSVEREPQENNSNYPDEPWR	S(1): 10.6; S(3): 89.4; S(13): 0.0; Y(15): 0.0	-0,23	0,68	-0,10	-0,09
Rgs14	P97492	EPGSSHLGSPDTAR	S(4): 0.0; S(5): 0.7; S(9): 99.2; T(12): 0.0	0,42	-0,11	0,28	-0,30
Rgs14	P97492	SLGSGESESESRPGK		-0,01	0,19	-0,47	0,33
Rltpr	Q3V3V9-2	LEAPPSPSLGSGLGSKPLPPYPTEPSSPER	S(6): 0.0; S(8): 0.0; S(11): 0.0; S(15): 0.0; S(26): 50.0; S(27): 50.0	0,94	-0,11	-1,26	0,34
Rnf20	Q5DTM8	ALVVPEPEPDSDSNQER	S(11): 6.2; S(13): 93.8	-0,91	0,04	0,56	-0,52
Scg2	Q03517	EHLGPGSSQEMER	S(7): 2.8; S(8): 97.2	0,62	0,26	0,33	0,06
Scg2	Q03517	RVPSPVSSEDDLQEEEQLEQAIK	S(4): 0.0; S(7): 99.5; S(8): 0.5	-1,28	0,62	0,05	0,09
Scg2	Q03517	VPSPVSSEDDLQEEEQLEQAIK	S(3): 0.0; S(6): 50.0; S(7): 50.0	-0,40	0,58	1,10	-0,48
Sdc4	O35988	VSMSSTAQGSNIFER	S(2): 0.7; S(4): 0.7; S(5): 5.4; T(6): 46.6; S(10): 46.6	0,54	-0,21	0,64	-0,17
Sept2	P42128	EGSPIPHDPDLGSK	S(3): 100.0; S(13): 0.0	0,60	-0,36	1,09	-0,59
Sept2	P42208	IYHLPDAESDEDEDFKEQTR	S(9): 100.0	0,54	-0,42	-0,34	0,40
Sipa1l3	G3X9J0	SSSEITLSECDVEEPGDPR	S(1): 8.5; S(2): 8.5; S(3): 83.1; T(6): 0.0; S(8): 0.0	0,97	-0,62	-1,57	0,30
Sirt2	Q8VDQ8	EHANIDAQSGSQAPNPSTTISPGKSPPPAK	S(9): 0.0; S(11): 0.0; S(17): 42.8; T(18): 42.8; T(19): 6.6; S(21): 1.1; S(25): 6.6	0,49	-0,17	-0,81	0,24
Sirt2	Q8VDQ8	REHANIDAQSGSQAPNPSTTISPGKSPPPAK	S(10): 0.0; S(12): 0.0; S(18): 0.7; T(19): 0.7; T(20): 32.9; S(22): 32.9; S(26): 32.9	1,33	-0,84	-0,62	0,21

Disease Models & Mechanisms • Supplementary information

Slc12a5	Q91V14	ETDPEVHLTWTK	T(2): 0.0; T(9): 99.6; T(11): 0.4	-1,67	1,01	0,52	-0,59
Slc1a3	P56564	DVEMGNSVIEENEMK	S(7): 100.0	-0,52	0,55	1,07	-0,75
Slc1a3	P56564	DVEMGNSVIEENEMK	S(7): 100.0	-0,76	0,34	0,21	-0,28
Slc1a4	O35874	SEEETSPLVTHQNPAGPVAIAPELESK	S(1): 0.0; T(5): 98.9; S(6): 1.1; T(10): 0.0; S(26): 0.0	0,50	-0,41	0,47	-0,81
Slc7a14	Q8BXR1	ETCSPVSEGEEFSSPATNTCGAK	T(2): 33.3; S(4): 33.3; S(7): 33.3; S(13): 0.0; S(14): 0.0; T(17): 0.0; T(19): 0.0	-0,94	0,98	0,48	-0,88
Slc7a14	Q8BXR1	HKQNSEALIANDELDCSPE	S(5): 0.0; S(17): 100.0	0,72	-0,14	-0,03	0,19
Slc7a14	Q8BXR1	HKQNSEALIANDELDCSPE	S(5): 0.0; S(17): 100.0	-0,06	0,09	-0,83	0,24
Slc9a6	A1L3P4	LVLPMDDSEPALNSLDDTRHSPA	S(8): 0.0; S(14): 0.0; T(18): 6.0; S(21): 94.0	-0,09	-0,19	-0,55	-0,02
Slc9a6	A1L3P4	LVLPMDDSEPALNSLDDTRHSPA	S(8): 0.0; S(14): 0.0; T(18): 0.7; S(21): 99.3	0,35	0,37	-0,59	0,25
Sparcl1	P70663	AAGSKEHIPHTEQQDQEGK	S(4): 100.0; T(11): 0.0	-0,68	-0,05	0,11	0,11
Sparcl1	P70663	AEKPSALNSEEETHEQSTEQDK	S(5): 33.1; S(9): 33.1; T(13): 33.1; S(17): 0.3; T(18): 0.3	-0,95	0,56	0,54	0,10
Sparcl1	P70663	GKESQEQPVSDSHQQPNESSK	S(4): 24.9; S(10): 24.9; S(12): 24.9; S(19): 24.9; S(20): 0.2	-0,68	0,06	-0,16	0,35
Sparcl1	P70663	KAESSPNAEPSDEGNSR	S(4): 0.0; S(5): 0.0; S(11): 100.0; S(16): 0.0	-0,29	-0,58	0,04	0,08
Specc1	Q5SXY1	ASSEDTLNKPGSASSGVAR	S(2): 50.0; S(3): 50.0; T(6): 0.0; S(12): 0.0; S(14): 0.0; S(15): 0.0	-0,12	0,07	-0,54	0,38
Specc1	Q5SXY1	GSPTGSSPNNASELSLASLTEK	S(2): 23.7; T(4): 23.7; S(6): 23.7; S(7): 23.7; S(12): 4.4; S(15): 0.9; S(18): 0.0; T(20): 0.0	2,59	-1,89	-0,17	0,51
Specc1	Q5SXY1	GSPTGSSPNNASELSLASLTEK	S(2): 31.6; T(4): 31.6; S(6): 31.6; S(7): 5.0; S(12): 0.2; S(15): 0.0; S(18): 0.0; T(20): 0.0	-0,15	0,11	-0,46	-0,05
Sptbn1	Q62261	ESSPVPSPTLDR	S(2): 93.7; S(3): 6.3; S(7): 0.0; T(9): 0.0	0,38	-0,22	2,11	-1,83
Sptbn1	Q62261	RPPSPDPNTK	S(4): 100.0; T(9): 0.0	0,04	-0,15	0,29	0,50
Srrm2	Q8BTI8-3	ELSHSPPRENSFESSLEFK	S(3): 12.7; S(5): 86.9; S(11): 0.1; S(14): 0.1; S(15): 0.3	-0,38	0,43	0,60	-0,35
Srrm2	Q8BTI8-3	SGAGSPPGK	S(1): 0.0; S(5): 100.0	-0,67	0,12	0,19	0,02
Srrm2	Q8BTI8-3	SLLPNSSQDELMEVEK	S(1): 0.2; S(6): 49.9; S(7): 49.9	-0,24	0,09	-0,48	0,30
Stim1	P70302	LIEGVHPGSLVEK		0,39	-0,16	0,53	-0,21
Syk	E9PWE9	ELNGTYAISGGR	T(5): 50.0; Y(6): 50.0; S(9): 0.0	0,84	-0,52	-0,64	0,17
Syn1	O88935	QASISGPAPTK	S(3): 100.0; S(5): 0.0; T(10): 0.0	-1,05	0,85	0,21	-0,02
Syn1	O88935	QSRPVAGGPGAPPAARPPASPSPQR	S(2): 0.0; S(20): 0.3; S(22): 99.7	0,25	0,42	0,70	0,30
Syn1	O88935	SQSLTNAFNLPEPAPPRPSLSQDEVK	S(1): 0.0; S(3): 0.0; T(5): 0.0; S(19): 50.0; S(21): 50.0	-0,46	0,61	-0,18	0,47
Synpr	D3Z5Q8	QASDWHSLSTLQLSLGLYK	S(3): 10.1; S(7): 89.9; S(9): 0.0; S(14): 0.0	2,79	-2,95	-0,98	0,05
Tcp11l1	Q8BTG3	QEAENGALDISK	S(11): 100.0	2,48	-1,94	0,57	-0,16
Tmem161a	M0QWV0	YIHPSEEELR	Y(1): 50.0; S(5): 50.0	-0,74	0,46	0,54	-0,83
Tmem161a	M0QWV0	YIHPSEEELR	Y(1): 50.0; S(5): 50.0	-0,75	0,41	0,21	-0,12
Tom1	O88746	GLEFPMTDLDMLSPIHTPQR	T(7): 0.0; S(13): 100.0; T(17): 0.0	-0,92	0,34	0,53	-1,27
Tppp	Q7TQD2	AANKTPPKSPGDPAR	T(5): 100.0; S(9): 0.0	-0,24	0,19	-0,63	0,67
Tppp	Q7TQD2	RLSLESEGANEGATAAPELSALEEAFR	S(3): 99.6; S(6): 0.4; T(14): 0.0; S(20): 0.0	-0,69	0,16	-0,16	-0,47
Tsc22d2	E9Q7M2	TEDVSSEIFDVSR	T(1): 0.0; S(5): 50.0; S(6): 50.0; S(12): 0.0	-1,91	1,05	1,42	0,06
Tsg101	Q61187	QILNLYAEENAIEDTIFYLGEALR	Y(6): 50.0; T(15): 50.0; Y(18): 0.0	0,50	-0,39	-0,58	0,48
Ube2w	D6RCG4	ELLALQNDPPPGMTLNEK	T(14): 100.0	0,71	-0,52	0,51	-0,60
Unc13a	Q4KUS2	ESYSDSMHSYEEFSEPR	S(2): 0.0; Y(3): 0.0; S(4): 0.0; S(6): 0.0; S(9): 93.7; Y(10): 6.3; S(14): 0.0	-0,70	0,21	0,59	-0,33
Uncharacterized protein C4orf21 homolog	Q0VGT4	SSSELSEVINNMSLLK	S(1): 0.5; S(2): 2.3; S(3): 12.9; S(6): 0.5; S(13): 83.9	0,38	-0,06	-0,43	0,53
Utp20	Q5XG71	EVMEANLPSILLK	S(9): 100.0	0,56	-0,75	-0,47	0,09
Wfs1	Q3UN10	LNATASLEQDK	T(4): 6.4; S(6): 93.6	-0,90	0,18	1,08	-0,38
Wfs1	Q3UN10	LNATASLEQDK	T(4): 0.0; S(6): 100.0	-0,09	-0,28	0,22	-0,55
Wwp1	Q8BZZ3	SSSPPIEIQQNGDALHENGDPATR	S(1): 0.4; S(2): 49.8; S(3): 49.8; T(23): 0.0	0,43	-0,38	0,49	0,17
Zswim8	Q3UHH1-5	RTFANGSADEFQR	T(2): 91.1; S(7): 8.9	-0,90	0,23	0,58	-0,08

Disease Models & Mechanisms • Supplementary information

Supplementary Table S8: Common proteins up and down phosphorylated in cortex of Tg(*Dyrk1a*) and Ts65Dn mice, and reverted by L41 treatment.

Gene name	Protein Group Accessions	Sequence	phosphoRS Site Probabilities	Ts65Dn vs WT	Ts65Dn +/-L41	TgDyrk1A vs WT	TgDyrk1A +/-L41
Adcy5	P84309	APPAGGSGSSAAAAAAAGGTEVRPR	S(7): 0.7; S(9): 49.7; S(10): 49.7; T(20): 0.0	0,17	0,03	0,51	-0,33
Adcy5	P84309	SVSPPGYAAQTAASPAPR	S(1): 0.0; S(3): 100.0; Y(7): 0.0; T(11): 0.0; S(14): 0.0	-0,75	0,01	0,29	-0,05
Add2	Q9QYB8	DIATEKPGSPVK	T(4): 0.0; S(9): 100.0	-0,28	-0,09	0,20	0,31
Add2	Q9QY81	ELASPLALTPR	S(4): 0.8; T(9): 99.2	0,64	0,36	-0,63	0,29
Agap2	Q3UHD9	AISAFGPSASINGLVK	S(3): 0.0; S(8): 50.0; S(10): 50.0	1,38	-0,04	0,70	-0,23
Aldoc	P05063	RLSQIGVENTEENR	S(3): 100.0; T(10): 0.0	-0,13	0,15	0,15	-0,61
Aldoc	P05063	GILAADESVGSMAK	S(8): 100.0; S(11): 0.0	-0,18	-0,22	0,07	0,31
Aldoc	P05063	GILAADESVGSMAK	S(8): 100.0; S(11): 0.0	0,64	-0,48	-0,49	0,08
Ap2a1	P17426-2	RDTSSNDINGGVEPTPSTVSTPSPSADLLGLR	T(3): 0.0; S(4): 0.0; S(5): 0.0; T(15): 0.0; S(17): 0.0; T(18): 0.0; S(20): 0.6; T(21): 0.6; S(23): 98.3; S(25): 0.6	0,56	0,15	1,37	-0,78
Apc	B2RUG9	QSVGSGSPVQTVGLETR	S(2): 0.0; S(5): 0.4; S(7): 99.5; T(11): 0.0; T(16): 0.0	0,48	-0,62	0,66	-0,39
Apc	B2RUG9	TPASKSPSEGPGATTSPR	T(1): 0.0; S(4): 49.9; S(6): 49.9; S(8): 0.2; T(14): 0.0; T(15): 0.0; S(16): 0.0	-0,80	-0,03	0,30	-0,18
Apc	B2RUG9	SPTGNTPPVIDSVSEK	S(1): 99.9; T(3): 0.1; T(6): 0.0; S(12): 0.0; S(14): 0.0	0,64	-0,29	-0,17	0,37
Arhgap21	B7ZCJ1	DQGEPVSPSEDEPFSWPGPK	S(7): 91.8; S(9): 8.2; S(15): 0.0	1,16	-1,15	0,53	-0,67
Arhgef7	Q9ES00	LAGGQTSQPTTPLTSPQR	T(6): 0.0; S(7): 0.0; T(10): 0.0; T(11): 0.0; T(14): 9.5; S(15): 90.5	0,61	0,22	-0,78	0,00
Asap1	H3BL41	QASEDSNDLTPTLPETPVPLPR	S(3): 87.1; S(6): 12.9; T(10): 0.0; T(12): 0.0; T(16): 0.0	0,22	-0,37	3,10	-1,42
Asap1	H3BL41	TLSDPPSPLPHGPPNK	T(1): 0.0; S(3): 100.0; S(7): 0.0	0,41	-0,73	-0,08	0,21
Asap1	H3BL41	GVFPVSFVHILSD	S(6): 0.0; S(12): 100.0	1,58	-1,07	-0,30	0,28
Atp13a1	Q9EPB4	EIHPYLVMDLEQS	Y(5): 0.0; S(13): 100.0	-0,84	0,19	1,31	-0,30
Atp1a3	Q6PIC6	VDNSSLTGESEPQTRSPDCTHDNPLETR	S(4): 16.7; S(5): 16.7; T(7): 16.7; S(10): 16.7; T(14): 16.7; S(16): 16.7; T(20): 0.0; T(27): 0.0	-0,88	0,70	1,07	-0,01
Avl9	Q80U56	DSLPSDSPPITVQPQANNR	S(2): 0.0; S(5): 0.7; S(7): 99.3; T(11): 0.0	0,97	-0,44	2,25	-1,20
Avl9	Q80U49	GASPVTPSTTPPPPTDPQLTK	S(3): 0.0; T(6): 1.1; S(8): 6.7; T(9): 45.5; T(10): 45.5; T(15): 1.1; T(20): 0.0	0,72	3,19	-2,00	-2,09
Begain	F8WIG2	GDIYCSDPALYCPDER	Y(4): 0.0; S(6): 100.0; Y(11): 0.0	-0,81	0,71	0,52	0,26
Begain	F8WIG2	RPSVDTPVTDVGFLR	S(3): 6.4; T(6): 93.6; T(9): 0.0	0,88	-0,15	0,41	0,34
Begain	F8WIG2	ADSFSEGDDLSQGHLAEPCFLR	S(3): 100.0; S(5): 0.0; S(11): 0.0	-0,63	0,05	0,09	0,28
Bod1l	E9Q6J5	GSDDVLVSGEVPECEVGHMSPR	S(2): 0.0; S(8): 0.0; S(20): 100.0	1,15	-0,65	0,86	-0,91
Bora	Q8BRF7	VNLEESTGVENSPAGARPK	S(6): 0.0; T(7): 0.0; S(12): 100.0	0,53	-0,27	-0,53	1,06
Braf	F6SZ47	SSSAPNVHINTIEPVNIDDLIR	S(1): 47.2; S(2): 47.2; S(3): 5.6; T(11): 0.0	-1,19	1,04	-0,65	-0,10
Brsk1	Q5RJI5	MQVPTAEEMSSLTPESSPELAK	T(5): 0.0; S(10): 0.0; S(11): 0.0; T(13): 0.0; S(16): 50.0; S(17): 50.0	-0,28	0,10	0,51	-0,09
Brsk1	Q5RJI5	SPVFSFSPEPGAGDEAR	S(1): 99.9; S(5): 0.1; S(7): 0.0	-0,23	0,44	-0,64	-0,08
Brsk1	Q5PR73	CDESPNREVQSSEAEALAR	S(4): 100.0; S(11): 0.0; S(12): 0.0	-0,70	0,76	0,40	-0,26
Cacna1b	A2AIR9	EVAEVSPMSAANISIAAR	S(6): 99.9; S(9): 0.1; S(14): 0.0	-0,83	0,45	0,71	-0,18
Camk2d	E9Q1T1	ENFSGGTSLWQNI	S(4): 88.3; T(7): 5.8; S(8): 5.8	0,84	-0,14	0,57	0,12
Camsap2	Q8C1B1	RFSPSQVPIQTR	S(3): 100.0; S(5): 0.0; T(11): 0.0	0,37	-0,11	0,50	-0,32
Camsap2	Q8C0T7	SEGTLQGAATTSTARR	S(1): 0.0; T(4): 0.1; T(10): 98.7; T(11): 1.1; S(12): 0.0; T(13): 0.0	-0,64	0,50	-0,58	0,15
Cep170b	Q80U49	GASPVTPSTTPPPPTDPQLTK	S(3): 0.0; T(6): 0.0; S(8): 0.0; T(9): 0.0; T(10): 0.4; T(15): 99.6; T(20): 0.0	-1,15	0,17	0,54	0,18
Cep170b	Q80U49	SQGPRDTDDDEEEPDPYGFIVQTAEIAEIAR	S(1): 0.2; T(7): 99.6; Y(17): 0.2; T(23): 0.0	-0,70	0,51	0,23	-0,11
Cep170b	Q80U49	SQGPRDTDDDEEEPDPYGFIVQTAEIAEIAR	S(1): 49.4; T(7): 49.4; Y(17): 1.3; T(23): 0.0	0,50	0,71	0,08	0,47

Disease Models & Mechanisms • Supplementary information

Cep170b	Q80TZ3	GASSPDMEPSYGGGLFDMVK	S(3): 6.9; S(4): 93.1; S(10): 0.0; Y(11): 0.0	-0,01	0,61	-0,58	0,39
Chga	P26339	GELEHSQQEEDGEEAMVGTPQGLFPQGGK	S(6): 100.0; T(19): 0.0	-0,63	0,55	0,73	-0,52
Chga	P26339	GELEHSQQEEDGEEAMVGTPQGLFPQGGK	S(6): 100.0; T(19): 0.0	0,62	-0,36	0,61	0,40
Col3a1	P08121	GENGSPGAPGAPGHPGPPGPVGPSGK	S(5): 100.0; S(24): 0.0	-0,85	-0,17	0,60	-0,19
Col3a1	P08121	GFPGNPGPPGSPGAAGHQGAIGSPGPAGPR	S(11): 50.0; S(23): 50.0	-1,00	-0,07	0,35	0,41
Comt	O88587-2	AVYQGPGSSPVKS	Y(3): 0.0; S(8): 0.5; S(9): 99.5; S(13): 0.0	0,17	-0,06	-0,25	-0,01
Comt	O88587-2	AVYQGPGSSPVK	Y(3): 0.0; S(8): 50.0; S(9): 50.0	0,68	0,24	-0,70	0,39
Ctnnd2	E9QKH8	TGSQHGPQNAAAATFQR	T(1): 8.4; S(3): 91.6; T(14): 0.0	-0,58	0,01	0,55	-0,97
Ctnnd2	E9QKH8	TGSQHGPQNAAAATFQR	T(1): 0.0; S(3): 100.0; T(14): 0.0	-0,38	0,05	0,12	-0,79
Ctps1	P70698	SGSSSPDSEITELK	S(1): 0.0; S(3): 0.0; S(4): 0.0; S(5): 0.2; S(8): 99.8; T(11): 0.0	0,85	-0,11	0,82	-1,17
D430041D05Rik	D3YZ21	QSLNSPSPGETEMDLLVTR	S(2): 0.0; S(5): 100.0; S(7): 0.0; T(11): 0.0; T(18): 0.0	-0,62	0,14	0,58	-0,10
Dchs1	F6TP58	ITFNLLAGGDGLFTVDPTTGHVR	T(2): 0.1; T(14): 43.8; T(18): 12.3; T(19): 43.8	0,51	-0,19	-1,01	-0,28
Dhx16	Q04690-2	SSSGLATYSPPMGAVSER	S(1): 0.0; S(2): 0.0; S(3): 0.0; T(7): 0.0; Y(8): 6.8; S(9): 93.2; S(16): 0.0	2,15	-1,77	1,04	-1,81
Dlg4	Q62073	SIQDLTVTGTEPGQVSSR	S(1): 100.0; T(6): 0.0; T(8): 0.0; T(10): 0.0; S(16): 0.0; S(17): 0.0	-0,78	0,33	-0,45	-0,15
Echdc1	Q9D8U8	SVSVDLNVDPSLQIDIPDALSER	S(1): 12.1; S(3): 87.9; S(11): 0.0; S(21): 0.0	-0,70	-0,37	-0,38	1,04
Ehd3	Q9QXY6	DKPMYDEIFYTLSPVDGK	Y(5): 0.0; Y(10): 0.1; T(11): 8.1; S(13): 91.9	1,38	-1,10	-0,62	-0,42
Ehd3	Q9QXW9	NHPGSDTSPEAEASSGGGGVALK	S(5): 6.3; T(7): 6.3; S(8): 87.5; S(14): 0.0; S(15): 0.0	-1,39	1,64	-2,43	0,58
Eml2	E9QK48	VGGYATSPSSPK	Y(4): 0.0; T(6): 0.3; S(7): 33.2; S(9): 33.2; S(10): 33.2	0,81	-0,76	-0,44	2,07
Epn1	Q80V94	IIEQPECSSPVIETER	S(8): 50.0; S(9): 50.0; T(14): 0.0	0,81	-0,44	0,48	-0,42
Fam195b	Q3UED7	LYFLDMVTEDAKTLLK	Y(2): 100.0; T(8): 0.0; T(13): 0.0	-0,69	0,23	-0,76	0,57
Gm15800	E9Q2E4	SMSAPSDLEMIGNEDLEFTR	S(1): 47.3; S(3): 47.3; S(6): 5.4; T(19): 0.0	0,67	-0,87	0,54	-1,11
Gm15800	E9Q2E4	SMSAPSDLEMIGNEDLEFTR	S(1): 49.9; S(3): 49.9; S(6): 0.1; T(19): 0.0	-0,19	0,27	-0,59	0,39
Gm15800	E9Q2E4	TEGTPPPPGQPAK	T(1): 0.0; T(4): 100.0	0,51	-0,58	-0,62	0,10
Gm996	A2AJA9	ASQGLATAPASPPVLQR	S(2): 0.0; T(7): 0.0; S(11): 100.0	0,18	-0,26	1,38	-0,52
Gm996	A2AJA9	QRSLEQLDELITDLVIDSR	S(3): 100.0; T(12): 0.0; S(18): 0.0	-0,66	0,43	0,26	-0,70
Gpkow	Q53YU8	VEDLNTFGPK	T(6): 100.0	-0,53	-0,21	1,66	-1,43
Gpr162	Q3UMB5	VASPANVGTLHTLSR	S(3): 100.0; T(9): 0.0; T(12): 0.0; S(14): 0.0	0,45	-0,36	-0,54	-0,06
Gprin1	Q3UNH4	AETVSPGEVDAMTLGK	T(3): 0.3; S(5): 99.7; T(13): 0.0	0,24	-0,04	0,49	0,00
Gprin1	Q3UN16	RLSLGSPDSR	S(3): 100.0; S(6): 0.0; S(9): 0.0	-0,83	0,68	0,19	-0,41
Gprin1	Q3UNH4	DLAAAAAQKSPSAEAAAPPPGPR	S(10): 100.0; S(12): 0.0	-0,71	0,33	0,17	-0,21
Gprin1	Q3UNH4	AETVSPGEVDAMTLGK	T(3): 7.0; S(5): 93.0; T(13): 0.0	0,58	-0,62	-0,04	0,88
Gprin1	Q3UNH4	SVATGPMTPQAAAPPAVPPVFPEVR	S(1): 0.0; T(4): 0.1; T(8): 99.9	0,12	-0,46	-0,23	0,36
Iqsec2	E9Q8N8	MFSNPDNGSPAMTHR	S(3): 0.0; S(9): 100.0; T(13): 0.0	-0,09	0,01	0,61	-0,74
Iqsec2	E9QAD8	AQNPAYFEGKPASLDEGAMAGAR	Y(6): 0.0; S(13): 100.0	-0,56	0,04	-0,04	0,51
Iqsec2	E9QAD8	SSSPGAGGGHSTSTSTSPATTLQR	S(1): 0.4; S(2): 0.4; S(3): 99.1; S(11): 0.0; T(12): 0.0; S(13): 0.0; T(14): 0.0; S(15): 0.0; T(16): 0.0; S(17): 0.0; T(20): 0.0; T(21): 0.0	-0,01	-0,31	-0,33	0,56
Kcnd1	Q03717	STSSLPPEPMEIVR	S(1): 0.0; T(2): 0.2; S(3): 9.9; S(4): 90.0	0,74	-0,34	0,67	-1,04
Kif1b	Q60575-2	SHAVFTIVFTQK	S(1): 100.0; T(6): 0.0; T(10): 0.0	-0,09	-0,45	-0,40	0,46
Kif1b	Q60520	VEDALSYLDQVK	S(6): 0.1; Y(7): 99.9	-1,57	1,20	-2,20	0,33
Klc2	D3YXZ3	VDSPTVNTTLR	S(3): 100.0; T(5): 0.0; T(8): 0.0; T(9): 0.0	0,67	-0,40	0,17	-0,02
Klc2	D3YXZ3	DSLDDLFPNEDEQSPAPSPGGGDVAAQHGGYEIPAR	S(2): 0.0; S(14): 100.0; S(18): 0.0; Y(31): 0.0	-0,52	0,73	-0,85	0,42
Lmtk3	Q5XJV6	ALVNGEPMSPEAGEK	S(9): 100.0	0,11	-0,23	1,08	0,12
Lmtk3	Q5XJV6	ALVNGEPMSPEAGEK	S(9): 100.0	0,70	-0,64	0,45	0,23
Lmtk3	Q5U4C3	QPPASVATLASVAAPAAPPASAPR	S(5): 87.2; T(8): 12.8; S(11): 0.0; S(21): 0.0	0,09	-0,43	-0,06	0,43
Lpin2	Q99P72	ESAEFSVLEYSEMGSSFNGSPK	S(2): 0.0; S(6): 0.0; Y(10): 0.0; S(11): 0.0; S(15): 0.0; S(16): 6.7; S(20): 93.3	0,85	-1,05	0,67	-0,31

Disease Models & Mechanisms • Supplementary information

Lppr2	Q8VCY8-2	LSPWEDLSQAPTMDSPLEK	S(2): 0.0; S(8): 0.0; T(12): 0.0; S(15): 100.0	-0,66	0,38	0,50	-0,12
Lpin2	Q99P72	ESAEFSVLEYSEMGSSFNGSPK	S(2): 0.0; S(6): 0.0; Y(10): 0.0; S(11): 0.0; S(15): 0.0; S(16): 6.7; S(20): 93.3	0,85	-1,05	0,67	-0,31
Lppr4	Q7TME0	QTYELNDLNR	T(2): 50.0; Y(3): 50.0	5,46	-6,56	0,63	-0,33
Lppr4	Q7TME0	MSLQVMDTEPEGQSPPR	S(2): 0.0; T(8): 0.0; S(14): 100.0	-0,69	0,19	0,49	-0,45
Mag	P20917	GESPELDLSYSHSDLGK	S(3): 100.0; S(9): 0.0; Y(10): 0.0; S(11): 0.0; S(13): 0.0	-0,61	0,12	0,51	0,19
Maoa	Q640R3	QNSLEYMDQNDDRLK	S(3): 99.7; Y(6): 0.3	-1,70	0,81	0,70	-0,26
Map1a	Q9QYR6-2	ELSSAVSPPNLHSDTPTFSYASLAGPTIPPR	S(3): 0.0; S(4): 0.8; S(7): 99.2; S(13): 0.0; T(15): 0.0; T(17): 0.0; S(19): 0.0; Y(20): 0.0; S(22): 0.0; T(27): 0.0	0,61	-0,29	0,18	0,12
Map1a	Q9QYR6-2	SPESLSSPAMEDLAMEWGGK	S(1): 0.0; S(4): 0.0; S(6): 5.6; S(7): 94.4	0,06	-0,97	-0,28	0,14
Map1a	Q9QYR6-2	MASPPPSGPPSAAHTPFHQSPVEEK	S(3): 98.9; S(7): 1.1; S(11): 0.0; T(15): 0.0; S(20): 0.0	-0,88	0,77	-0,30	-0,31
Map1a	Q9QYR6-2	MASPPPSGPPSAAHTPFHQSPVEEK	S(3): 95.4; S(7): 2.3; S(11): 2.3; T(15): 0.0; S(20): 0.0	-0,46	-0,01	-0,36	0,22
Map1a	Q9QYR6-2	EMTLDQKSPEK	T(3): 1.2; S(8): 98.8	-0,09	0,23	-0,39	0,17
Map1a	Q9QYR6-2	MASPPPSGPPSAAHTPFHQSPVEEK	S(3): 100.0; S(7): 0.0; S(11): 0.0; T(15): 0.0; S(20): 0.0	-0,44	0,10	-0,41	0,46
Map1a	Q9QYR6-2	APISLSQDPSPPLNGSTTSCGPDR	S(4): 0.0; S(6): 0.0; S(10): 100.0; S(16): 0.0; T(17): 0.0; T(18): 0.0; S(19): 0.0	0,00	0,02	-0,42	0,16
Map1a	Q9QYR6-2	EMTLDQKSPEK	T(3): 0.1; S(8): 99.9	-0,10	0,29	-0,42	0,14
Map1b	P14873	TPEEGGYSYEISEK	T(1): 100.0; Y(7): 0.0; S(8): 0.0; Y(9): 0.0; S(12): 0.0	1,27	-1,03	0,56	-0,01
Map1b	P14873	SPPLLGSESPYEDFLSADSK	S(1): 100.0; S(7): 0.0; S(9): 0.0; Y(11): 0.0; S(16): 0.0; S(19): 0.0	-0,58	0,26	0,45	-0,28
Map1b	P14873	TTRSPDTSAYCYETMEK	T(1): 0.0; T(2): 6.8; S(4): 93.1; T(7): 0.0; S(8): 0.0; Y(10): 0.0; Y(12): 0.0; T(14): 0.0	0,61	-0,65	0,35	0,20
Map1b	P14873	DYNASASTISPPSSMEEDK	Y(2): 0.0; S(5): 0.0; S(7): 0.2; T(8): 1.2; S(10): 87.6; S(13): 9.9; S(14): 1.2	0,51	0,03	0,25	-0,40
Map1b	P14873	TTRSPDTSAYCYETMEK	T(1): 0.0; T(2): 0.0; S(4): 100.0; T(7): 0.0; S(8): 0.0; Y(10): 0.0; Y(12): 0.0; T(14): 0.0	0,36	-0,57	0,10	0,17
Map1b	P14873	QGFPDRESPVSDLTSTGLYQDK	S(8): 100.0; S(11): 0.0; T(14): 0.0; S(15): 0.0; T(16): 0.0; Y(19): 0.0	-0,09	0,04	-0,14	0,46
Map1b	P14873	TLEVVSPSQSVTGSAGHTPYYQSPTDEK	T(1): 0.0; S(6): 99.3; S(8): 0.7; S(10): 0.0; T(12): 0.0; S(14): 0.0; T(18): 0.0; Y(20): 0.0; Y(21): 0.0; S(23): 0.0; T(25): 0.0	-0,19	0,57	-0,30	0,09
Map1b	P14873	TLEVVSPSQSVTGSAGHTPYYQSPTDEK	T(1): 0.0; S(6): 94.1; S(8): 0.8; S(10): 0.8; T(12): 0.8; S(14): 0.8; T(18): 0.8; Y(20): 0.8; Y(21): 0.8; S(23): 0.0; T(25): 0.0	-0,02	-0,23	-0,39	0,08
Map1b	P14873	DYNASASTISPPSSMEEDKFSK	Y(2): 0.0; S(5): 0.0; S(7): 0.6; T(8): 0.6; S(10): 98.8; S(13): 0.0; S(14): 0.0; S(21): 0.0	0,05	-0,38	-0,43	-0,24
Map1b	P14873	LGGDVSPTQIDVSQFGSFK	S(6): 50.0; T(8): 50.0; S(13): 0.0; S(17): 0.0	0,31	0,12	-0,45	0,04
Map1b	P14873	DYNASASTISPPSSMEEDK	Y(2): 0.0; S(5): 0.0; S(7): 0.0; T(8): 0.0; S(10): 100.0; S(13): 0.0; S(14): 0.0	-0,36	0,12	-0,61	0,68
Map1b	P14873	VLSPLRSPPLLGSESPYEDFLSADSK	S(3): 0.8; S(7): 96.9; S(13): 0.8; S(15): 0.8; Y(17): 0.8; S(22): 0.0; S(25): 0.0	0,44	0,29	-0,81	0,34
Map2	P20357	ETSPETSLIQDEVALK	T(2): 47.5; S(3): 47.5; T(6): 2.5; S(7): 2.5	0,09	-0,15	0,87	-1,31
Map2	P20357	RLSNVSSSGSINLLESPQLATLAEDVTAALAK	S(3): 10.4; S(6): 76.0; S(7): 10.4; S(8): 1.5; S(10): 0.2; S(16): 1.5; T(21): 0.0; T(27): 0.0	0,34	-0,77	-0,35	0,44
Map2	P20357	YTVPLPSPVQDSENLSGESGSFYEGTDDK	Y(1): 0.0; T(2): 0.0; S(7): 99.7; S(12): 0.2; S(16): 0.0; S(19): 0.0; S(21): 0.0; Y(23): 0.0; T(26): 0.0	-0,54	0,75	-0,62	0,10
Map6d1	Q149S1	LLSEVEELNMSLR	S(3): 2.5; S(11): 97.5	-0,83	0,09	0,47	-0,73

Map6d1	Q14BB9	SDVAVPLTLHGYSDPGSEESGADCSVSR	S(1): 0.0; T(8): 0.0; Y(12): 0.4; S(13): 0.4; S(17): 49.2; S(20): 49.2; S(25): 0.4; S(27): 0.4	-0,76	-0,19	0,01	0,32
Mark1	Q8VHG2	DTTVISHSPNTSYDTALEAR	T(2): 0.0; T(3): 0.0; S(6): 0.0; S(8): 100.0; T(11): 0.0; S(12): 0.0; Y(13): 0.0; T(15): 0.0	1,65	-1,70	0,48	-0,49
Mark1	Q8VHJ5	KPPEFEGGESLSSGSLCQR	S(10): 0.3; S(12): 48.1; S(13): 48.1; S(15): 3.6	0,26	-0,20	-0,04	0,55
Mtfr1l	Q9CW07	SFSLPADPILQAAK	S(1): 3.8; S(3): 96.2	-1,06	0,17	-0,56	0,09
Otud7a	Q8R554	AAPGTGGPTPGRSPPAPAR	T(5): 0.0; T(9): 0.0; S(13): 100.0	0,55	0,00	0,29	-0,46
Otud7a	Q8R554	TVNTVESLAPGGADAPGPAEHK	T(1): 50.0; T(4): 50.0; S(7): 0.0	0,04	0,17	0,03	0,31
Otud7a	Q8R550	SIEVENDFLPVEK	S(1): 100.0	-0,02	-0,45	-0,44	0,44
Pcdh1	F7BJK1	SNSPLPSIQLQPQSPSASK	S(1): 0.0; S(3): 0.0; S(7): 0.0; S(14): 47.4; S(16): 47.4; S(18): 5.2	-0,12	-0,13	0,47	-0,20
Pcdh1	F7BJK1	IHLPLNYPPGSPDLGR	Y(7): 0.0; S(11): 100.0	-0,75	0,31	-0,16	-0,19
Pclo	Q9QYX7	SEGITKPSLQQPSPK	S(1): 0.0; T(5): 0.0; S(8): 0.0; S(13): 100.0	-0,30	0,27	0,74	-0,80
Pclo	Q9QYX7	SHSSQNSQQSPKPSVIK	S(1): 0.0; S(3): 0.0; S(4): 0.0; S(7): 0.5; S(10): 99.5; S(14): 0.0	-0,04	0,01	0,24	0,34
Pclo	Q9QYX7	KIVDSGVQTDDEETADR	S(5): 0.1; T(9): 99.9; T(14): 0.0	0,55	0,03	0,22	-0,04
Pclo	Q9QYX7	KDSFSQESSPSSPSDLAK	S(3): 0.0; S(5): 0.0; S(8): 0.0; S(9): 0.0; S(11): 6.7; S(12): 86.5; S(14): 6.7	0,08	0,11	0,14	0,44
Pclo	Q9QYX7	SMSDPKPLSPTADESSR	S(1): 0.5; S(3): 99.5; S(9): 0.0; T(11): 0.0; S(15): 0.0; S(16): 0.0	0,40	-0,48	0,07	0,07
Pclo	Q9QYX7	LPTAVSLYSPTDEQSVMQK	T(3): 0.0; S(6): 0.0; Y(8): 0.0; S(9): 50.0; T(11): 50.0; S(15): 0.0	0,17	-0,59	0,06	-0,24
Pclo	Q9QYX7	GGSLGCQTETDPDTQSPPYMGATSPPKDK	S(3): 0.0; T(8): 0.0; T(10): 0.0; T(14): 20.0; S(16): 20.0; Y(19): 20.0; T(23): 20.0; S(24): 20.0	-0,97	0,52	-0,10	0,16
Pclo	Q9QYX7	GAHAHSGPTSAGSSSVPSPGQPGSPSVSK	S(6): 0.0; T(9): 0.7; S(10): 0.0; S(13): 0.0; S(14): 0.0; S(15): 0.0; S(18): 0.0; S(24): 98.6; S(26): 0.7; S(28): 0.0	-0,03	0,19	-0,11	0,44
Pclo	Q9QYX7	SPVPSQQASPK	S(1): 0.0; S(5): 0.0; S(9): 100.0	0,38	-0,48	-0,15	0,26
Pclo	Q9QYX7	TQPQKVSPEQPQDQQK	T(1): 0.2; S(7): 99.8	-0,05	0,31	-0,20	0,47
Phkb	Q7TQH0-2	EVDGLLTSDPMGSPVSSK	T(7): 0.0; S(8): 0.0; S(13): 99.8; S(16): 0.2; S(17): 0.0	1,35	-0,93	-0,38	0,27
Pnpo	Q91WH7	VDNSSLTGESEPQTRSPECTHESPLETR	S(4): 0.0; S(5): 0.0; T(7): 0.2; S(10): 0.7; T(14): 77.4; S(16): 15.3; T(20): 3.2; S(23): 3.2; T(27): 0.0	-1,07	0,21	0,56	-0,12
Pnpo	Q91XF0	GLATGDSPLGPMTHHGEEDWVYER	T(4): 0.3; S(7): 99.7; T(13): 0.0; Y(22): 0.0	-0,79	0,34	0,42	-0,63
Ppme1	Q8BVL3	VTSSVPLPSGGTSSPSR	T(2): 0.0; S(3): 0.0; S(4): 0.0; S(9): 0.5; T(12): 6.3; S(13): 80.5; S(14): 6.3; S(16): 6.3	-0,58	0,31	-0,38	0,38
Ppp1r1b	Q60829	AVQHLQTISNLSENQASEEEDELGELR	T(7): 0.0; S(9): 0.0; S(12): 0.0; S(17): 100.0	-0,56	0,91	0,65	-0,01
Ppp1r1b	Q60829	RPNPCAYTPPSLK	Y(7): 0.0; T(8): 100.0; S(11): 0.0	1,04	-1,14	0,04	-0,38
Ppp1r1b	Q60823	YFDDEFTAQSITITPPDR	Y(1): 0.0; T(7): 0.0; S(10): 0.0; T(12): 0.2; T(14): 99.8	0,90	-0,06	-0,66	0,54
Ppp1r9b	Q6R5C6	NQALEMLSR	S(8): 100.0	0,55	0,44	0,66	-0,42
Ppp1r9b	Q6R891	ASSLNENVDHSALLK	S(2): 0.3; S(3): 99.7; S(11): 0.0	-0,18	0,12	-0,01	-0,06
Prl8a1	Q9DA80	VSSQPAPSQER	S(2): 50.0; S(3): 50.0; S(8): 0.0	0,47	-0,50	0,48	-0,01
Rmdn3	Q3UJU9	SHSLPNSLDYAQASER	S(1): 0.0; S(3): 100.0; S(7): 0.0; Y(10): 0.0; S(14): 0.0	0,00	0,07	0,68	-0,58
Rmdn3	Q3UID0	DMDEPSPVPNVEEVTLPK	S(6): 100.0; T(15): 0.0	-1,02	0,57	0,30	-0,54
Rtn1	Q8K0T0	GSVSEDELIAAIK	S(2): 3.4; S(4): 96.6	0,64	-0,72	0,48	-0,53
Sec22b	O08547	NLGSINTELQDVQR	S(4): 93.0; T(7): 7.0	1,10	-0,76	0,87	0,02
Sgip1	Q8VD37	LPSISDLDSIFGPVLSPK	S(3): 0.0; S(5): 0.0; S(9): 0.0; S(16): 100.0	0,00	0,05	0,76	-0,15
Sgip1	Q8VCY8-2	LSPWEDLSQAPTMDSPLEK	S(2): 0.0; S(8): 0.0; T(12): 0.4; S(15): 99.6	1,34	-1,54	-0,70	0,03
Sipa1l1	Q8C0T5	LIDLESPTPESQK	S(6): 99.7; T(8): 0.3; S(11): 0.0	-0,33	0,11	1,30	-1,29
Sipa1l1	Q8C0T5	QDPVVHLSPNK	S(8): 100.0	2,16	-2,31	-0,04	-0,49
Sirt2	Q8VDQ8-2	EHANIDAQSGSQAPNPSTTISPGK	S(9): 0.0; S(11): 0.0; S(17): 0.0; T(18): 0.0; T(19): 0.0; S(21): 100.0	-0,43	0,43	1,09	-0,55

Disease Models & Mechanisms • Supplementary information

Sirt2	Q8VDQ8-2	EHANIDAQSGSQAPNPSTTISPGKSPPPAK	S(9): 0.0; S(11): 0.0; S(17): 0.0; T(18): 0.0; T(19): 0.0; S(21): 0.0; S(25): 100.0	-0,96	0,51	0,22	0,17
Syn1	O88935	QSRPVAGGPGAPPAARPPASPSPQR	S(2): 0.0; S(20): 50.0; S(22): 50.0	-0,34	-0,11	0,47	-0,29
Syn1	O88935	QSRPVAGGPGAPPAARPPASPSPQR	S(2): 0.0; S(20): 1.3; S(22): 98.7	-0,27	-0,14	0,46	-0,16
Syn1	O88935	LPSPTAAPQQSASQATPVTQGQGR	S(3): 99.4; T(5): 0.6; S(11): 0.0; S(13): 0.0; T(16): 0.0; T(19): 0.0	0,50	-0,13	0,35	-0,84
Syn1	O88935	DASPGRGSHSQSSSPGALTLGR	S(3): 100.0; S(8): 0.0; S(10): 0.0; S(12): 0.0; S(13): 0.0; S(14): 0.0; T(19): 0.0	0,21	0,10	0,28	0,33
Syngap1	F6SEU4	DLNSSIDLQSFMAR	S(4): 0.3; S(5): 99.7; S(10): 0.0	-0,62	-0,27	0,61	-0,13
Syngap1	F6SEU4	SSPAYCTSSSDITEPEQK	S(1): 0.1; S(2): 0.4; Y(5): 3.5; T(7): 30.9; S(8): 3.5; S(9): 30.9; S(10): 30.9; T(13): 0.0	0,13	-0,52	0,39	-0,39
Syngap1	F6SEU4	EGSIGGSGGSGGGGGGGLKPSITK	S(3): 0.0; S(7): 33.2; S(10): 33.2; S(21): 33.2; T(23): 0.3	-0,96	0,32	0,22	0,03
Syngap1	F6SEU4	GPSAEMQGYMMR	S(3): 100.0; Y(9): 0.0	-0,25	-0,05	-0,03	0,36
Synj1	E9Q7S0	TPGPPSSQGSPVDTQPAAQK	T(1): 0.0; S(6): 0.7; S(7): 7.6; S(10): 91.7; T(14): 0.0	0,28	0,00	0,65	-0,42
Synj1	E9Q7S0	TPGPPSSQGSPVDTQPAAQK	T(1): 0.0; S(6): 1.3; S(7): 10.2; S(10): 87.2; T(14): 1.3	0,47	-0,19	0,54	-0,78
Tmem163	Q8C996	GHAPSTAAPAPSPAPMSSSVQSDEER	S(5): 0.0; T(6): 0.0; S(12): 0.0; S(17): 0.0; S(18): 0.0; S(19): 0.0; S(22): 100.0	-0,02	0,24	0,56	-0,35
Tmem163	Q8C996	GHAPSTAAPAPSPAPMSSSVQSDEER	S(5): 0.0; T(6): 0.0; S(12): 0.0; S(17): 84.4; S(18): 7.5; S(19): 0.7; S(22): 7.5	-1,36	0,86	-0,23	-0,22
Tmsb4x	P20065-2	TETQEKNPLPSK	T(1): 0.0; T(3): 100.0; S(11): 0.0	-0,63	0,15	-0,37	-0,36
Tp53bp1	P70399	GREDAVAEDVCIDLTCDSGSQAVPSPATR	T(15): 0.0; S(18): 0.0; S(20): 0.0; S(25): 99.3; T(28): 0.7	-0,60	0,71	0,15	-0,73
Tp53bp1	P70399	ASQEPFSPAEDVMETDLLEGLAANQDRPSK	S(2): 0.0; S(7): 1.4; T(15): 98.6; S(29): 0.0	0,14	0,10	-0,38	0,23
Trank1	Q8BTI8	SGTPPRPGSVTNMQADECTATPQR	S(1): 0.0; T(3): 100.0; S(9): 0.0; T(11): 0.0; T(19): 0.0; T(21): 0.0	0,45	-0,13	-0,54	-0,01
Trappc8	E9PWG2	NSDSNLLSLDGLDNEVK	S(2): 50.0; S(4): 50.0; S(8): 0.1	0,50	-0,58	-0,43	0,26
Trim3	Q3B7Z2	GATVLPANPPGSAGSGK	T(3): 100.0; S(12): 0.0; S(15): 0.0	-0,87	0,71	-0,39	-0,08
Twf1	Q91YM2	AGSPLCNSNLQDSEEDVEPPSYHLFR	S(3): 100.0; S(8): 0.0; S(13): 0.0; S(21): 0.0; Y(22): 0.0	-0,64	0,32	-0,55	-0,08
Uqcrh	P99028	SQTEEDCTEELFDFLHAR	S(1): 8.2; T(3): 91.8; T(8): 0.0	-0,50	0,55	0,57	0,07
Usp10	P52479-2	TCDSPQNPVDFISGPVPDSPFPR	T(1): 11.1; S(4): 88.9; S(13): 0.0; S(19): 0.0	0,80	0,04	-0,39	0,41
Usp45	E9Q4D8	ISLSSAPQLEPLVVELSSPGPLTSALFL	S(2): 69.4; S(4): 15.3; S(5): 15.3; S(17): 0.0; S(18): 0.0; T(23): 0.0; S(24): 0.0	1,04	0,07	-0,53	0,31
Xrn2	Q9DB05	AIDIYEQVGTSAMDSPLLK	Y(5): 0.0; T(10): 0.0; S(11): 0.0; S(15): 100.0	0,60	-1,65	-1,33	0,96
Yap1	P46938-2	QSSFEIPDDVPLPAGWEMAK	S(2): 0.5; S(3): 99.5	0,59	0,30	0,16	0,19
Yap1	P46938-2	GDSETDLEALFNAVMNPK	S(3): 50.0; T(5): 50.0	0,67	-0,41	-0,57	0,25
Ywhaz	P63101	TAFDEAIAELDTLSEESYK	T(1): 0.0; T(12): 0.0; S(14): 100.0; S(17): 0.0; Y(18): 0.0	0,86	-1,02	1,19	-1,35

Disease Models & Mechanisms • Supplementary information

Supplementary Table S9: Common proteins up and down phosphorylated in cerebellum of Tg(*Dyrk1a*) and Ts65Dn mice, and reverted by L41 treatment.

Gene name	Protein Group Accessions	Sequence	phosphoRS Site Probabilities	Ts65Dn vs WT	Ts65Dn +/-L41	TgDyrk1A vs WT	TgDyrk1A +/-L41
Aak1	Q3UHJ0-2	STQLLQAAAAEASLNK	S(1): 50.0; T(2): 50.0; S(13): 0.0	0,00	1,00	-0,32	0,66
Aak1	Q3UHJ0-2	ILSDVTHSAVFGVPASK	S(3): 4.6; T(6): 47.7; S(8): 47.7; S(16): 0.0	0,77	-0,03	-0,61	0,18
Aak1	Q3UHJ0-2	EQGSSGLGSGSSGGGGSSSGLGSGYIGR	S(4): 0.0; S(5): 0.0; S(9): 47.3; S(11): 47.3; S(12): 5.5; S(17): 0.0; S(18): 0.0; S(19): 0.0; S(23): 0.0; Y(25): 0.0	-0,32	0,08	-0,76	0,66
Acot11	Q8VHQ9	SISHPESGDPPTMAEGEGYR	S(1): 100.0; S(3): 0.0; S(7): 0.0; T(12): 0.0; Y(19): 0.0	-0,02	-0,30	2,75	-2,76
Acot11	Q8VHQ9	KSISHPESGDPPTMAEGEGYR	S(2): 50.0; S(4): 50.0; S(8): 0.0; T(13): 0.0; Y(20): 0.0	-0,38	-0,01	-0,03	0,12
Akap12	Q9WTQ5	QSQVTEEEAAAAQTEGPSTPSSFPAQEEHR	S(2): 0.0; T(5): 0.0; T(14): 0.0; S(18): 92.3; T(19): 7.7; S(21): 0.0; S(22): 0.0	-0,15	0,05	0,52	-1,14
Akap12	Q9WTQ5	QAQSSTEIPLQAESGQGTEEEAAKDGEENR	S(4): 33.3; S(5): 33.3; T(6): 33.3; S(14): 0.0; T(18): 0.0	-0,60	0,31	0,43	-0,04
Akap12	Q9WTQ5	SATLSSTESTASGMQDEVR	S(1): 0.0; T(3): 0.0; S(5): 0.0; S(6): 0.0; T(7): 0.0; S(9): 0.0; T(10): 0.0; S(12): 100.0	0,10	-0,12	0,39	-0,09
Akap12	Q9WTQ5	AEPEILELESK	S(10): 100.0	-0,07	-0,20	-0,52	0,39
Akt1	P31750	SGSPSDNSGAEEMEVSLAKPK	S(1): 0.0; S(3): 99.0; S(5): 0.9; S(8): 0.0; S(16): 0.0	-0,46	0,13	-0,43	0,23
Akt2	Q60823	YFDDEFTAQSITITPPDRYDSLDPLELDQR	Y(1): 0.0; T(7): 0.8; S(10): 96.6; T(12): 0.8; T(14): 0.8; Y(19): 0.8; S(21): 0.0	0,54	-0,15	0,30	-0,07
Akt2	Q60823	YFDDEFTAQSITITPPDR	Y(1): 0.0; T(7): 0.0; S(10): 0.0; T(12): 0.1; T(14): 99.9	0,31	0,01	-0,37	0,14
Akt2	Q91VE4	TSLGTEQHHAAAAAAVASVACR	T(1): 0.1; S(2): 0.5; T(5): 99.3; S(18): 0.0	0,28	0,08	-0,51	0,63
Amph	Q7TQF7	TPSPPEEPSPLPSPTASPNHTLAPASPAPVRPR	T(1): 2.9; S(3): 95.8; S(9): 0.6; S(13): 0.6; T(15): 0.1; S(17): 0.0; T(21): 0.0; S(26): 0.0	0,49	-0,29	0,31	0,33
Amph	Q7TQF7	AFSIQGAPSDSGPLR	S(3): 0.0; S(9): 7.2; S(11): 92.8	0,20	-0,32	-0,37	0,45
Ap3d1	O54774	VDIITEEMPENALPSDEDDKDPNDPYR	T(5): 0.0; S(15): 100.0; Y(26): 0.0	0,07	0,15	-0,18	-0,32
Ap3d1	O54774	HSSLPTESDEDIAPAQR	S(2): 0.0; S(3): 0.0; T(6): 0.0; S(8): 100.0	-0,01	0,41	-0,24	-1,08
Ap3d1	O54774	VDIITEEMPENALPSDEDDKDPNDPYR	T(5): 0.0; S(15): 100.0; Y(26): 0.0	0,32	0,29	-0,36	0,33
Ap3d1	O54774	VDIITEEMPENALPSDEDDKDPNDPYR	T(5): 0.0; S(15): 100.0; Y(26): 0.0	-0,35	0,71	-1,60	-0,41
Arfgap1	Q9EPJ9-2	NSNSDGWESWEGASGEGR	S(2): 0.1; S(4): 99.9; S(9): 0.0; S(14): 0.0	-0,25	0,80	0,07	-0,64
Arfgap1	Q9EPJ9-2	AKSPSSDSWTCADASTGR	S(3): 100.0; S(5): 0.0; S(6): 0.0; S(8): 0.0; T(10): 0.0; S(15): 0.0; T(16): 0.0	0,02	0,09	0,06	0,69
Arfgap1	Q9EPJ9-2	RSSDSWDVWGSGSASNNK	S(2): 33.3; S(3): 33.3; S(5): 33.3; S(11): 0.0; S(13): 0.0; S(15): 0.0	-0,43	0,93	-0,10	0,11
Arfgap1	Q9EPJ9-2	RSSDSWDVWGSGSASNNK	S(2): 5.7; S(3): 88.5; S(5): 5.7; S(11): 0.0; S(13): 0.0; S(15): 0.0	-0,23	0,02	-0,13	0,01
Arfgap1	Q9EPJ9-2	NSNSDGWESWEGASGEGR	S(2): 0.0; S(4): 100.0; S(9): 0.0; S(14): 0.0	-0,15	0,38	-0,60	-0,41
Arhgap21	B7ZCJ1	RNSEGSEASCTEGSLTPSLDSR	S(3): 91.9; S(6): 8.1; S(9): 0.0; T(11): 0.0; S(14): 0.0; T(16): 0.0; S(18): 0.0; S(21): 0.0	0,65	-0,38	0,13	-0,70
Arhgap21	B7ZCJ1	TRSWDYIEGQTEATATVNSESQIPDSNGER	T(1): 49.8; S(3): 49.8; Y(6): 0.4; T(11): 0.0; T(14): 0.0; T(16): 0.0; S(19): 0.0; S(21): 0.0; S(26): 0.0	0,08	-0,28	0,04	-0,52
Arhgap21	B7ZCJ1	DQGEPVSPSEDEPFSWPGPK	S(7): 100.0; S(9): 0.0; S(15): 0.0	-0,42	-0,07	-0,49	-0,04
Arhgef12	Q8R4H2	SEGVQDAEPQSLVGSPSTR	S(1): 0.0; S(11): 0.0; S(15): 83.9; S(17): 8.0; T(18): 8.0	0,44	0,03	0,40	-0,16
Arhgef12	F8VQN6	SEGVQDAQEPQSLVGSPSTR	S(1): 0.0; S(12): 10.9; S(16): 67.4; S(18): 10.9; T(19): 10.9	0,45	-0,31	-0,05	-0,26
Arvcf	P98203-2	SLAADDEGGPDLEPDYSTATR	S(1): 100.0; Y(16): 0.0; S(17): 0.0; T(18): 0.0; T(20): 0.0	-1,31	0,64	-0,38	-0,34
Atp1a3	Q6PIC6	CIELSSGSVK	S(5): 0.2; S(6): 49.9; S(8): 49.9	-0,19	0,13	0,41	-0,26
Atp1a3	Q6PIC6	SPDCTHDNPLETR	S(1): 100.0; T(5): 0.0; T(12): 0.0	0,40	-0,21	0,25	-0,31
Atp1a3	Q6PIC6	DVAGDASESALLK	S(7): 100.0; S(9): 0.0	0,41	0,24	-0,08	0,49
Brsk1	Q5RJI5	SPVFSFSPEPGAGDEAR	S(1): 100.0; S(5): 0.0; S(7): 0.0	-0,07	-0,03	0,47	-0,07
Brsk1	Q5RJI5	SSGGTPLHSPLHTPR	S(1): 0.0; S(2): 0.0; T(5): 0.0; S(9): 100.0; T(13): 0.0	-0,42	0,17	0,19	-0,18

Disease Models & Mechanisms • Supplementary information

Bsn	Q8VDQ8-2	EHANIDAQSGSQAPNPSTTISPGK	S(9): 0.0; S(11): 0.0; S(17): 0.0; T(18): 0.0; T(19): 0.0; S(21): 100.0	0,14	-0,42	0,27	-0,44
Bsn	O88737	SSVSQSPAPTYPSDSHYTSLEQNVPR	S(1): 0.0; S(2): 0.0; S(4): 1.2; S(6): 98.8; T(10): 0.0; Y(11): 0.0; S(13): 0.0; S(15): 0.0; Y(17): 0.0; T(18): 0.0; S(19): 0.0	-0,04	0,50	0,09	0,04
Bsn	O88737	SPQVLYSPVSPLSPHR	S(1): 0.0; Y(6): 0.0; S(7): 0.0; S(10): 5.6; S(13): 94.4	0,54	-0,93	0,01	-0,37
Bsn	O88737	TLPSPPPEEAHLPLAGQVPSQLYAASLLQR	T(1): 0.9; S(4): 99.1; S(20): 0.0; Y(23): 0.0; S(26): 0.0	-0,40	0,31	-0,15	0,08
Bsn	O88737	EPELEMESLTGSPEDR	S(8): 4.7; T(10): 47.6; S(12): 47.6	-0,62	0,49	-0,19	-0,21
Bsn	O88737	LYSSMSDTNLAEAGLNYHAQR	Y(2): 48.0; S(3): 48.0; S(4): 0.3; S(6): 3.7; T(8): 0.0; Y(17): 0.0	-0,41	-0,14	-0,22	0,39
Bsn	O88737	SADCSVQTDDEDNADWEQPVR	S(1): 0.0; S(5): 0.0; T(8): 100.0	-0,28	0,38	-0,47	0,59
Bsn	O88737	RPHSLSITPEAFDSDEELGDILEEDDSLAWGR	S(4): 0.0; S(6): 0.0; T(8): 0.0; S(14): 100.0; S(27): 0.0	0,19	0,32	-1,33	1,00
Cacna1g	P39061-2	QTVSVPGPPGPPGPPGPPGAMGASAGGQVR	T(2): 6.0; S(4): 93.5; S(24): 0.5	1,06	-0,23	-0,14	-0,13
Cacna1g	Q5SUF8	SESEPDFFSPSVDGDGDR	S(1): 0.0; S(3): 100.0; S(9): 0.0; S(11): 0.0	0,34	0,61	-0,74	0,89
Cacna1g	F6RJ39	APVVLQPEQIVSEEETPPPLLTK	S(12): 0.0; T(16): 100.0; T(22): 0.0	-0,67	0,61	-1,34	0,92
Camsap2	Q8C1B1	SESVEGFLSPSR	S(1): 0.0; S(3): 0.0; S(9): 99.7; S(11): 0.3	0,00	-0,23	0,67	-0,39
Camsap2	Q8C1B1	LNQSSPDNLTDTK	S(4): 94.4; S(5): 5.6; T(10): 0.0; T(12): 0.0	-0,35	0,16	0,30	-0,08
Camsap2	Q8C1B1	SVSNEGLTLNNSR	S(1): 0.0; S(3): 100.0; T(8): 0.0; S(12): 0.0	0,11	-0,39	0,00	0,25
Caskin1	Q6P9K8-2	HISSSQELLGDGPPGPGSPMSR	S(3): 6.2; S(4): 6.2; S(5): 87.6; S(18): 0.0; S(21): 0.0	-0,16	0,37	1,09	-0,91
Caskin1	Q6P9K8-2	SQEYLLDEGMAPGTPPKEVR	S(1): 0.0; Y(4): 0.0; T(14): 100.0	0,05	-0,43	-0,01	-0,40
Caskin1	Q6P9K8-2	SVSESSPGDSPVKPPEGSSGAAR	S(1): 0.0; S(3): 0.0; S(5): 0.0; S(6): 0.0; S(10): 100.0; S(18): 0.0; S(19): 0.0	-0,46	0,29	-0,14	-0,12
Caskin1	Q6P9K8-2	AQPGSPQALGGPHGPATAK	S(5): 100.0; T(17): 0.0	-0,02	0,14	-0,58	-0,04
Caskin2	Q8VHK1	VGLSPDSPAGDRNSVGSEGSVGSIR	S(4): 99.0; S(7): 1.0; S(14): 0.0; S(17): 0.0; S(20): 0.0; S(23): 0.0	-0,42	0,26	-0,46	-0,10
Ckb	Q04447	RGTGGVDTAAVGGVFDVSNADR	T(3): 100.0; T(8): 0.0; S(18): 0.0	0,75	-0,41	0,31	-0,27
Ckb	Q04447	RGTGGVDTAAVGGVFDVSNADR	T(3): 97.0; T(8): 3.0; S(18): 0.0	0,67	-0,27	-0,73	-0,04
Clns1a	Q923F1	LGEESKEPLSDEDEEDNDDVEPISEFR	S(5): 0.0; S(10): 100.0; S(24): 0.0	0,99	-0,29	0,50	0,29
Coro1a	O89053	RATPEPSGTPSSDTVSR	T(3): 99.8; S(7): 0.1; T(9): 0.0; S(11): 0.0; S(12): 0.0; T(14): 0.0; S(16): 0.0	1,14	-1,00	0,53	-0,03
CR025	Q8BH50	RDSSESQLASTESDKPTTGR	S(3): 50.0; S(4): 50.0; S(6): 0.0; S(10): 0.0; T(11): 0.0; S(13): 0.0; T(17): 0.0; T(18): 0.0	-0,45	0,92	-0,79	-0,24
Cstf2t	Q8C7E9	QGGGQPSSFSPGQSQVTPQDQEK	S(7): 0.0; S(8): 0.0; S(10): 99.9; S(14): 0.0; T(17): 0.0	-0,41	1,02	0,64	-0,46
Ctage5	H3BK44	AFLSPPTLLEGPLR	S(4): 99.3; T(7): 0.7	1,34	-0,37	1,18	-0,37
Dagla	Q6WQJ1	DEGHLFYIDPAIPEENPSLSSR	Y(7): 0.0; S(18): 33.3; S(20): 33.3; S(21): 33.3	-0,05	0,29	0,27	-0,45
Dagla	Q6WQJ1	LLSPVAAASAAR	S(3): 100.0; S(9): 0.0	-0,37	-0,12	-0,38	0,28
Dbi	Q4VWZ5	TQPTDEEMLFIYSHFK	T(1): 0.0; T(4): 0.0; Y(12): 95.8; S(13): 4.2	0,45	-0,23	-0,38	0,33
Dclk1	Q9JLM8	SKSPASTSSVNGTPGSQLSTPR	S(1): 49.2; S(3): 49.2; S(6): 0.5; T(7): 0.5; S(8): 0.5; S(9): 0.0; T(13): 0.0; S(16): 0.0; S(19): 0.0; T(20): 0.0	-0,46	-0,06	0,43	0,25
Dclk1	Q9JLM8	AQPAPPELNSESEDYSPSSSETVR	S(10): 0.0; S(12): 0.0; Y(15): 1.3; S(16): 94.8; S(18): 1.3; S(19): 1.3; S(20): 1.3; T(22): 0.2	-0,17	0,18	0,14	-0,78
Dclk1	Q9JLM8	REESEEGFQIPATITER	S(4): 100.0; T(13): 0.0; T(15): 0.0	0,42	-0,10	-0,11	-0,76
Dclk1	Q9JLM8	VCSSMDENDGPGEGDELGR	S(3): 50.0; S(4): 50.0	0,32	0,38	-0,56	-0,45
Dmtn	Q9WV69	LQSTEFSPSGSEAGSPGLQNGEGQR	S(3): 46.9; T(4): 46.9; S(7): 6.1; S(9): 0.1; S(11): 0.0; S(15): 0.0	0,45	-0,27	-0,37	-0,12
Dmxl2	Q8BPN8-2	RQSESIAAPPVASEDMDK	S(3): 92.1; S(5): 7.9; S(13): 0.0	0,01	-0,80	0,25	0,44
Dmxl2	Q8BPN8-2	RQSESIAAPPVASEDMDK	S(3): 50.0; S(5): 50.0; S(13): 0.0	1,00	-1,10	-0,10	0,43
Dmxl2	Q8BPN8-2	ALLTPQDEECADGDTEVDVIAEQLK	T(4): 99.3; T(15): 0.7	0,12	-0,55	-1,02	0,42
Dmxl2	Q8C7U1	AQLLHALSLDEGGPEPSLSDSSSGGSFGR	S(8): 0.8; S(17): 58.2; S(19): 12.6; S(21): 12.6; S(22): 12.6; S(23): 3.0; S(26): 0.2	0,69	-0,93	-0,95	0,99
Dnajc6	Q80TZ3	TATSASASPTLR	T(1): 0.0; T(3): 0.0; S(4): 0.0; S(6): 0.5; S(8): 99.5; T(10): 0.0	-0,06	-0,46	0,07	-0,01
Dnajc6	Q80TZ3	GASSPDMEPSYGGGLFDMVK	S(3): 0.4; S(4): 99.6; S(10): 0.0; Y(11): 0.0	-0,46	-0,04	-0,64	0,27
Dock4	P59764	NSAPASMSPDGTR	S(2): 0.0; S(6): 8.8; S(8): 91.2; T(12): 0.0	0,06	0,00	0,60	-0,59

Disease Models & Mechanisms • Supplementary information

Dock4	P59764	ASPLLSDK	S(2): 100.0; S(6): 0.0	-0,73	0,59	-0,76	0,25
Dsg2	O55111	LDLSIIVTNK	S(4): 100.0; T(8): 0.0	0,41	0,00	0,43	0,07
Efhd2	Q8C845	ADLNQGIGEPQSPSRR	S(12): 98.8; S(14): 1.2	1,57	-1,47	0,58	-0,51
Eif4b	Q8BGD9	ARPTTDSFDDYPPR	T(4): 0.0; T(5): 0.0; S(7): 100.0; Y(11): 0.0	0,24	-0,43	0,70	-0,71
Eif4b	Q8BGD9	SPPYTAFLGNLPYDVTEDSIK	S(1): 33.3; Y(4): 33.3; T(5): 33.3; Y(13): 0.0; T(16): 0.0; S(19): 0.0	1,28	-0,92	0,68	-0,50
Eif4b	Q8BGD9	SQSSDTEQPSPTSGGGK	S(1): 0.0; S(3): 92.9; S(4): 6.6; T(6): 0.5; S(10): 0.0; T(12): 0.0; S(13): 0.0	-0,30	-0,12	-0,01	-0,43
Eif4b	Q8BGD9	SQSSDTEQPSPTSGGGK	S(1): 11.4; S(3): 85.2; S(4): 1.7; T(6): 1.7; S(10): 0.0; T(12): 0.0; S(13): 0.0	-0,10	-0,71	-0,06	-0,11
Epn1	Q80VP1	SPGAFDMSGVGGSLAESVGSPPPAATPTPTPPTR	S(1): 0.0; S(8): 0.2; S(13): 4.0; S(17): 4.0; S(20): 91.5; T(26): 0.2; T(28): 0.0; T(30): 0.0; T(33): 0.0	0,54	-0,17	0,45	-0,03
Epn2	Q8CHU3	AGGSPASYHGSTSPR	S(4): 100.0; S(7): 0.0; Y(8): 0.0; S(11): 0.0; T(12): 0.0; S(13): 0.0	-0,43	0,00	0,64	0,21
Epn2	Q5NCM5	AGGSPASYHGSPEASLCPQHR	S(4): 0.0; S(7): 0.0; Y(8): 0.0; S(11): 50.0; S(15): 50.0	1,31	-1,46	0,25	-0,12
Fam131b	Q3TY60-2	KVSDVTSSGVQSFDEEEGDANN	S(3): 100.0; T(6): 0.0; S(7): 0.0; S(8): 0.0; S(12): 0.0	-1,38	0,87	0,12	-0,06
Fam131b	Q3TY60-2	KVSDVTSSGVQSFDEEEGDANN	S(3): 0.0; T(6): 0.0; S(7): 0.0; S(8): 0.0; S(12): 100.0	-0,48	0,72	-1,01	-0,20
Fam169a	Q5XG69	SQSEEQSEASSEHLEQFTQSAEK	S(1): 0.0; S(3): 0.0; S(7): 0.0; S(10): 50.0; S(11): 50.0; T(18): 0.0; S(20): 0.0	0,03	0,08	-0,41	-0,52
Fam169a	Q5XG69	SQSEEQSEASSEHLEQFTQSAEK	S(1): 0.0; S(3): 1.1; S(7): 9.1; S(10): 9.1; S(11): 80.5; T(18): 0.1; S(20): 0.0	-0,44	0,64	-0,59	-0,39
Fxr1	Q61584-4	RGPNYTSGYGTNSELSNPSETESER	Y(5): 0.0; T(6): 0.0; S(7): 0.0; Y(9): 0.0; T(11): 0.0; S(13): 0.0; S(16): 0.2; S(19): 98.2; T(21): 1.5; S(23): 0.0	0,71	0,00	-0,63	-0,32
Gja1	P23242	MGQAGSTISNSHAQPFDFPDDSQNAK	S(6): 25.0; T(7): 25.0; S(9): 25.0; S(11): 25.0; S(22): 0.0	-0,39	0,16	-0,24	-0,34
Gja1	P23242	SDPYHATTGPLSPSK	S(1): 0.0; Y(4): 0.0; T(7): 0.0; T(8): 0.0; S(12): 99.4; S(14): 0.6	0,06	0,31	-0,40	-0,30
Gm2933	Q1LZI5	LMYVLALSSDLLVIQK	Y(3): 90.2; S(8): 9.7; S(9): 0.1	-0,37	0,43	-0,42	0,22
Gm996	A2AJA9	SLEQLDELITDLVIDSR	S(1): 100.0; T(10): 0.0; S(16): 0.0	0,76	-0,35	0,74	-0,08
Gprin1	Q3UNH4	AETVSPGEVDAMTLGK	T(3): 4.6; S(5): 95.4; T(13): 0.0	0,61	-0,41	0,11	-0,03
Gprin1	Q3UNH4	DLAAAAAQKSPSAEAAAPPPGPR	S(10): 50.0; S(12): 50.0	0,10	-0,40	-0,15	-0,12
Gprin1	Q3UNH4	VDIVSPGGDNAGSLR	S(5): 0.0; S(13): 100.0	0,33	-0,42	-0,18	-0,26
Gprin1	Q3UNH4	AETVSPGEVDAMTLGK	T(3): 0.3; S(5): 99.7; T(13): 0.0	0,24	-0,03	-0,37	0,41
Gpsm1	Q6IR34-3	AAQSSVTASPQTEEFFDLIASSQSR	S(4): 0.0; S(5): 0.0; T(7): 8.5; S(9): 83.0; T(12): 8.5; S(21): 0.0; S(22): 0.0; S(24): 0.0	0,57	-0,02	0,57	-0,17
Gramd1b	Q80TI0	SRSPTPQNQDGDTMVEK	S(1): 0.0; S(3): 99.5; T(5): 0.5; T(13): 0.0	-0,57	0,05	-0,30	-0,38
Gramd1b	Q80TI0	SRSPTPQNQDGDTMVEK	S(1): 1.5; S(3): 96.9; T(5): 1.5; T(13): 0.0	-0,34	0,27	-0,48	-0,41
Gtf3c1	Q8K284-2	VAASPRPGAEEQAEAQAPAQLAAPEDADAGGPR	S(4): 100.0	-0,41	0,63	-0,42	-0,26
Hcn2	O88703	DSASPGAASGLDPLDSAR	S(2): 7.6; S(4): 92.4; S(9): 0.0; S(16): 0.0	0,54	-0,47	-0,34	0,26
Hcn2	O88703	RAPPGPLPPAASPGPPAASPPAAPSSPR	S(12): 0.0; S(19): 0.0; S(25): 50.0; S(26): 50.0	0,18	-0,66	-0,42	0,25
Htt	P42859	ASDPSPATPDSESVIVAMER	S(2): 0.0; S(5): 0.3; T(8): 97.7; S(11): 1.8; S(13): 0.3	1,39	-0,60	0,78	-1,61
Htt	P42859	SGSIVELLAGGGSSCSPVLSR	S(1): 0.0; S(3): 100.0; S(13): 0.0; S(14): 0.0; S(16): 0.0; S(20): 0.0	0,15	-0,23	0,13	-0,45
Htt	P42859	SLNPQKSGEEEDSGSAAQLGMCNR	S(1): 0.2; S(7): 99.7; S(13): 0.1; S(15): 0.1	-1,18	0,68	0,08	-1,06
Ifi44	Q8BV66	DMLILSALK	S(6): 100.0	0,44	-0,27	1,08	-0,49
Il16	O54824	DPGLPESPPPGQRPSTK	S(7): 100.0; S(15): 0.0; T(16): 0.0	0,00	-0,08	0,55	-0,15
Il16	O54824	SASPETPASPGK	S(1): 0.9; S(3): 98.2; T(6): 0.9; S(9): 0.0	-0,36	2,20	-1,72	0,19
Itih2	Q61703	SLPEESGEETDTVDPVTLYSYK	S(1): 1.1; S(6): 89.0; T(10): 9.8; T(12): 0.1; T(17): 0.0; Y(19): 0.0; S(20): 0.0; Y(21): 0.0	-0,59	0,09	-0,44	0,21
Jph3	Q9ET77	EFSPSFQHR	S(3): 100.0; S(5): 0.0	-0,66	0,26	-0,50	-0,20
Kif17	A2AM72	EYQEEIK	Y(2): 100.0	0,94	-0,75	-0,87	-0,03
Kif2a	P28740-1	ARPSQLPEQSSSAQQNGSVSDISPVQAAK	S(4): 0.0; S(10): 0.0; S(11): 0.0; S(12): 0.0; S(18): 0.0; S(20): 0.0; S(23): 100.0	-0,25	0,07	0,63	0,01

Disease Models & Mechanisms • Supplementary information

Kif2a	P28740-1	EFGISPSDIPFSQGGGSRPDLSPSYDYDDFSPSITR	S(5): 0.3; S(7): 0.3; S(12): 32.9; S(17): 32.9; S(22): 32.9; S(24): 0.3; Y(25): 0.3; Y(27): 0.0; S(31): 0.0; S(33): 0.0; T(35): 0.0	0,81	-0,62	0,47	-0,09
Lgalsl	Q8VED9	LDDGHLNNSLGSPVQADVYFPR	S(9): 0.0; S(12): 100.0; Y(19): 0.0	0,49	0,18	0,70	-0,52
Lgalsl	Q8VED9	LDDGHLNNSLGSPVQADVYFPR	S(9): 0.0; S(12): 100.0; Y(19): 0.0	0,14	-0,35	0,37	-0,32
Lnp	Q7TQ95	ADSVPNLEPSEESLVTK	S(3): 100.0; S(10): 0.0; S(13): 0.0; T(16): 0.0	-0,35	0,09	0,38	-0,39
Map1a	Q9QYR6	MASPPPSGPPSAAHTPFHQSPVEEK	S(3): 11.2; S(7): 11.2; S(11): 77.5; T(15): 0.0; S(20): 0.0	-0,48	0,22	0,79	-0,28
Map1a	Q9QYR6	ELSSEPRTPPAQK	S(3): 0.0; S(4): 0.0; T(8): 100.0	0,07	0,42	0,50	0,03
Map1a	Q9QYR6	ELSSAVSPPNLHSDTPTFSYASLAGPTIPPR	S(3): 0.0; S(4): 0.0; S(7): 98.9; S(13): 0.5; T(15): 0.5; T(17): 0.0; S(19): 0.0; Y(20): 0.0; S(22): 0.0; T(27): 0.0	0,09	-0,22	0,50	-0,47
Map1a	Q9QYR6	VAELEEEQSQGSSSYSDWVK	S(9): 100.0; S(12): 0.0; S(13): 0.0; S(14): 0.0; Y(15): 0.0; S(16): 0.0	-0,13	0,17	0,41	-0,66
Map1a	Q9QYR6	QLSPESLGTLQFGELSLGK	S(3): 100.0; S(6): 0.0; T(9): 0.0; S(16): 0.0	0,29	-0,20	0,40	-0,20
Map1a	Q9QYR6	WLAESPVGLPPEEEDKLTR	S(5): 100.0; T(18): 0.0	0,15	0,36	0,35	0,10
Map1a	Q9QYR6	MASPPPSGPPSAAHTPFHQSPVEEK	S(3): 94.7; S(7): 2.6; S(11): 2.6; T(15): 0.1; S(20): 0.0	0,36	0,00	-0,08	0,37
Map1a	Q9QYR6	ESTFLDEGPNEQEITPLQHTPR	S(2): 0.0; T(3): 0.0; T(15): 100.0; T(20): 0.0	0,33	-0,68	-0,24	-0,06
Map1a	Q9QYR6	WLAESPVGLPPEEEDK	S(5): 100.0	0,17	0,00	-0,47	0,11
Map1a	P07742	ATGSYIAGTNGNSNGLVPMLRVYNNTAR	T(2): 33.3; S(4): 33.3; Y(5): 33.3; T(9): 0.0; S(13): 0.0; Y(23): 0.0; T(26): 0.0	0,00	-0,11	-0,58	0,41
Map1a	Q9QYR6	EMTLDQKSPEK	T(3): 0.0; S(8): 100.0	-0,05	0,36	-0,61	0,33
Map1b	P14873	TTEAAATAVGTAATTAAVVAAAGIAASGPVK	T(1): 0.0; T(2): 0.2; T(7): 16.3; T(11): 83.3; T(14): 0.2; T(15): 0.0; S(27): 0.0	-0,09	-0,23	0,83	-0,66
Map1b	P14873	ETAAAHQASSSPPIDAATAEPYGFR	T(2): 0.0; S(9): 6.0; S(10): 47.0; S(11): 47.0; T(18): 0.0; Y(22): 0.0	-0,32	0,04	0,54	0,56
Map1b	P14873	QGFPDRESPVSDLTSTGLYQDKQEEK	S(8): 87.7; S(11): 12.0; T(14): 0.3; S(15): 0.0; T(16): 0.0; Y(19): 0.0	0,04	0,49	0,54	0,73
Map1b	P14873	DYNASASTISPPSSMEEDK	Y(2): 0.0; S(5): 0.0; S(7): 0.0; T(8): 0.2; S(10): 99.7; S(13): 0.0; S(14): 0.0	1,39	-1,48	0,41	0,31
Map1b	P14873	LGGDVSPTQIDVSQFGSFK	S(6): 5.6; T(8): 94.4; S(13): 0.0; S(17): 0.0	0,09	-0,05	0,32	0,47
Map1b	P14873	DLTTSSVEKDSGGK	T(3): 0.0; T(4): 0.0; S(5): 0.0; S(6): 0.0; S(11): 100.0	-0,17	0,41	0,13	-0,26
Map1b	P14873	TPEEGGYSYEISEK	T(1): 100.0; Y(7): 0.0; S(8): 0.0; Y(9): 0.0; S(12): 0.0	-0,20	-0,09	0,13	0,17
Map1b	P14873	SQGSTSNSDWMK	S(1): 0.0; S(4): 0.0; T(5): 0.1; S(6): 50.0; S(8): 50.0	-0,53	0,52	0,05	-0,04
Map1b	P14873	VLSPLRSPPLLGSESPYEDFLSADSK	S(3): 0.0; S(7): 100.0; S(13): 0.0; S(15): 0.0; Y(17): 0.0; S(22): 0.0; S(25): 0.0	-0,28	0,07	0,04	0,48
Map1b	P14873	SVSPGVTQAVVEEHCASPEEK	S(1): 0.0; S(3): 0.0; T(7): 0.0; S(17): 100.0	-0,30	0,43	0,00	0,06
Map1b	P14873	QGFPDRESPVSDLTSTGLYQDK	S(8): 100.0; S(11): 0.0; T(14): 0.0; S(15): 0.0; T(16): 0.0; Y(19): 0.0	0,06	0,50	-0,19	0,08
Map1b	P14873	TTRSPDTSAYCYETMEK	T(1): 0.0; T(2): 0.2; S(4): 99.8; T(7): 0.0; S(8): 0.0; Y(10): 0.0; Y(12): 0.0; T(14): 0.0	1,70	-1,15	-0,23	0,12
Map1b	P14873	ASLSPMDEPVPDSESPVEK	S(2): 50.0; S(4): 50.0; S(13): 0.0; S(15): 0.0	0,31	-0,27	-0,46	0,66
Map1b	P14873	SPDTSAYCYETMEK	S(1): 95.0; T(4): 0.3; S(5): 4.7; Y(7): 0.0; Y(9): 0.0; T(11): 0.0	0,17	0,10	-0,57	0,01
Map2	P20357	ETSPETSLIQDEVALK	T(2): 99.7; S(3): 0.2; T(6): 0.0; S(7): 0.0	-0,04	0,21	0,76	-0,66
Map2	P20357	LASVSADAEVAR	S(3): 94.4; S(5): 5.6	-0,33	-0,02	0,41	-0,22
Map2	P20357	VDHGAEIITQSPSR	T(9): 47.7; S(11): 47.7; S(13): 4.7	-0,32	0,46	0,33	0,15
Map2	P20357	GSAQESLDTISPK	S(2): 0.0; S(6): 0.0; T(9): 0.4; S(11): 99.5	-0,27	0,55	0,24	0,04
Map2	P20357	ASQPSPPAQEAGYSTLAQSYTPDHPSELPEEPSSPQER	S(2): 0.0; S(5): 0.0; Y(13): 0.0; S(14): 0.0; T(15): 0.0; S(19): 0.0; Y(20): 0.0; T(21): 0.0; S(26): 0.0; S(33): 12.1; S(34): 87.9	0,58	0,09	0,18	-0,34
Map2	P20357	ETSPETSLIQDEVALK	T(2): 7.3; S(3): 92.7; T(6): 0.0; S(7): 0.0	-0,22	0,49	0,04	0,39
Map2	P20357	GVVESVVTIEDDFITVVQTTTDEGESGSHSVR	S(5): 0.0; T(8): 0.1; T(15): 0.6; T(19): 12.1; T(20): 12.1; T(21): 12.1; S(26): 38.9; S(28): 12.1; S(30): 12.1	0,05	0,10	-0,06	-0,63
Map2	P20357	DGSPDAPATPEKEEVAFSEYK	S(3): 100.0; T(9): 0.0; S(18): 0.0; Y(20): 0.0	-0,39	0,54	-0,21	0,31
Marcks	P26645	GEATAERPGEAAVASSPSK	T(4): 0.0; S(15): 47.3; S(16): 47.3; S(18): 5.3	-0,13	0,04	0,04	-0,27
Marcks	P26645	VNGDASPAAAEPGAK	S(6): 100.0	-0,36	0,79	-0,57	-0,11

Disease Models & Mechanisms • Supplementary information

Mier1	Q5UAK0-4	APSPPPTASNSSNSQSEK	S(3): 0.8; T(7): 90.7; S(9): 8.5; S(11): 0.0; S(12): 0.0; S(14): 0.0; S(16): 0.0	-0,44	0,89	1,09	-1,33
Mobp	Q9D2P8	HQPAASPVVVR	S(6): 100.0	-0,40	0,08	-0,07	0,20
Mobp	Q9D2P8	ATSPQRPK	T(2): 50.0; S(3): 50.0	0,56	-0,13	-0,44	0,54
Mpdz	Q8VBX6-2	NVQGLGITIAGYIGDK	T(8): 98.6; Y(12): 1.4	1,31	-0,50	-0,40	-0,13
Mprip	P97434-2	DFASEAPTAPLSDACPLSPHR	S(4): 0.0; T(8): 0.0; S(12): 0.0; S(18): 100.0	-1,62	0,43	2,12	-0,71
Mroh2a	F5H8M8	CLGSLQGQVSSSAMAEGMEALTK	S(4): 0.0; S(10): 0.1; S(11): 3.5; S(12): 3.5; T(22): 93.0	-0,45	0,42	-0,48	-0,36
Myo5a	D3YZ62	IGELEVGQMENISPGQIIDEPIRPVNIPR	S(13): 100.0	-0,26	-0,16	0,49	-1,08
Myo5a	D3YZ62	IGELEVGQMENISPGQIIDEPIRPVNIPR	S(13): 100.0	-0,24	0,03	0,22	-0,53
Myo5a	D3YZ62	RTSSIADEGTYTLDSILR	T(2): 48.1; S(3): 48.1; S(4): 3.8; T(10): 0.0; Y(11): 0.0; T(12): 0.0; S(15): 0.0	0,44	-0,18	-0,01	0,13
N4bp3	A2A699	TGLCSPEDNSLTPLLDEVVAPEGR	T(1): 3.3; S(5): 92.8; S(10): 0.7; T(12): 3.3	0,45	-0,16	-0,94	1,30
Nfix	Q3TYK3	SPAAGSSQSSGWPNDVDAGSPR	S(1): 0.0; S(6): 0.0; S(7): 0.0; S(9): 0.0; S(10): 0.0; S(20): 100.0	0,87	-0,38	0,08	0,63
Nfix	P70257-1	SPAAGSSQSSGWPNDVDAGPASLK	S(1): 93.5; S(6): 2.7; S(7): 2.7; S(9): 0.5; S(10): 0.5; S(22): 0.0	0,06	-0,09	-0,47	0,10
Npm1	Q61937	CGSGPVHISGQHLVAVEEDAESEDEDEEDVK	S(3): 0.0; S(9): 0.0; S(22): 100.0	0,22	-0,07	0,12	0,44
Npm1	Q61937	CGSGPVHISGQHLVAVEEDAESEDEDEEDVK	S(3): 0.0; S(9): 0.0; S(22): 100.0	-0,38	-0,08	-0,38	0,24
Nucks1	Q80XU3	KVVDYSQFQESDDADEDYGR	Y(5): 0.0; S(6): 0.0; S(11): 100.0; Y(18): 0.0	-0,91	0,20	0,01	-0,03
Nucks1	Q80XU3	VVDYSQFQESDDADEDYGR	Y(4): 0.0; S(5): 0.0; S(10): 100.0; Y(17): 0.0	-0,12	0,07	-0,50	0,30
Oxct1	Q9D0K2	SARNFNLPMCK	S(1): 100.0	0,05	0,12	0,51	-0,45
Oxct1	Q9D0K2	HHTKFYTDPVEAVK	T(3): 100.0; Y(6): 0.0; T(7): 0.0	0,77	-1,00	-0,29	1,14
Oxr1	Q4KMM3	ESLPIELDQLSGR	S(2): 0.0; S(11): 100.0	-0,38	-0,10	0,36	-0,23
Oxr1	Q4KMM3	VVSSTSEEEEAFTEK	S(3): 0.0; S(4): 0.0; T(5): 0.3; S(6): 99.7; T(13): 0.0	-0,15	0,38	0,17	-0,07
Park2	Q9WVS6	SHETNASGGDEPQSTSEGSIWESR	S(1): 10.2; T(4): 89.6; S(7): 0.2; S(14): 0.0; T(15): 0.0; S(16): 0.0; S(19): 0.0; S(23): 0.0	0,39	-0,33	0,58	-0,30
Park2	Q9WVS6	SHETNASGGDEPQSTSEGSIWESR	S(1): 1.6; T(4): 96.8; S(7): 1.6; S(14): 0.0; T(15): 0.0; S(16): 0.0; S(19): 0.0; S(23): 0.0	-0,38	0,10	0,39	-0,30
Pclo	Q9QYX7	KDSFSQESSPSSPSDLAK	S(3): 0.0; S(5): 0.0; S(8): 0.0; S(9): 0.0; S(11): 48.1; S(12): 48.1; S(14): 3.8	-0,27	0,31	0,65	-0,42
Pclo	Q9QYX7	KDSFSQESSPSSPSDLAK	S(3): 0.0; S(5): 0.0; S(8): 0.0; S(9): 0.0; S(11): 94.8; S(12): 5.2; S(14): 0.0	-0,04	0,22	0,38	-0,25
Pclo	Q9QYX7	GSVPAAAAESPSMHR	S(2): 0.0; S(10): 100.0; S(12): 0.0	-0,12	0,40	0,16	-0,30
Pclo	Q9QYX7	SVDTSVQTDDEDQDEWDMPSR	S(1): 0.0; T(4): 0.6; S(5): 0.6; T(8): 98.8; S(20): 0.0	0,55	-0,50	-0,04	-0,05
Pclo	Q9QYX7	SMSDPKPLSPTADESSR	S(1): 0.0; S(3): 100.0; S(9): 0.0; T(11): 0.0; S(15): 0.0; S(16): 0.0	-0,07	0,36	-0,06	0,11
Pclo	Q9QYX7	LPTAVSLYSPTDEQSVMQK	T(3): 0.0; S(6): 0.0; Y(8): 0.4; S(9): 49.8; T(11): 49.8; S(15): 0.0	0,34	-0,27	-0,50	0,28
Pclo	Q9QYX7	DSFSQESSPSSPSDLAK	S(2): 0.0; S(4): 0.0; S(7): 0.0; S(8): 0.0; S(10): 5.0; S(11): 94.7; S(13): 0.3	0,25	0,19	-0,57	0,14
Pclo	Q9QYX7	GGSLGCQTETDPDTQSPPYMGATSPPKDK	S(3): 0.0; T(8): 0.0; T(10): 0.0; T(14): 20.0; S(16): 20.0; Y(19): 20.0; T(23): 20.0; S(24): 20.0	-0,15	-0,03	-0,81	0,30
Phf3	B2RQG2	NTVDIVDKPENSPQR	T(2): 0.0; S(12): 100.0	-0,42	0,49	-0,40	0,05
Phkb	Q7TSH2	QSSTADAPEAQHEPGITITEWK	S(2): 47.7; S(3): 47.7; T(4): 4.7; T(17): 0.0; T(19): 0.0	-0,55	0,17	0,08	-0,11
Phkb	Q7TSH2	EGPNFITMEGTVSDHIER	T(7): 10.0; T(11): 10.0; S(13): 80.1	0,89	-0,42	-0,38	-0,21
Phkb	Q7TSH2	SGSVYEPLK	S(1): 5.8; S(3): 93.8; Y(5): 0.4	0,51	-0,84	-0,46	-0,04
Pianp	Q6P1B3	QEESQQPLTDLSPAGVTVLGAFGDSPTPTPDHEEPR	S(4): 0.0; T(9): 0.0; S(12): 0.0; T(17): 0.0; S(25): 99.5; T(27): 0.3; T(29): 0.3	0,41	-0,24	-0,41	0,85
Pkp4	Q68FH0	TEPEQGTLYSPEQTSLHESEGSLGNSR	T(1): 0.0; T(7): 0.0; Y(9): 0.0; S(10): 0.4; T(14): 49.8; S(15): 49.8; S(19): 0.0; S(22): 0.0; S(26): 0.0	1,38	-1,42	0,82	-1,08
Pkp4	Q68FH0	DGWNQNHFITPVSTLER	T(10): 100.0; S(13): 0.0; T(14): 0.0	-0,38	0,36	0,12	0,04
Pkp4	Q68FH0	VGSPLTLTDAQTR	S(3): 100.0; T(6): 0.0; T(8): 0.0; T(12): 0.0	-0,46	0,05	-0,04	-0,14
Pkp4	Q68FH0	AQSPSYVTSTGVSPSR	S(3): 0.0; S(5): 0.0; Y(6): 0.0; T(8): 0.0; S(9): 0.1; T(10): 0.1; S(13): 9.5; S(15): 90.2	0,80	-0,75	-0,05	0,09

Disease Models & Mechanisms • Supplementary information

Pkp4	Q68FH0	VASPSQGQVGSSSPK	S(3): 0.0; S(5): 0.0; S(11): 0.0; S(12): 5.3; S(13): 94.7	-0,14	0,05	-0,40	-0,13
Pkp4	Q68FH0	AEQYPGSPDSWV	Y(4): 0.0; S(7): 100.0; S(10): 0.0	-0,39	-0,23	-0,40	0,42
Plcl2	Q8K394	EFQVSFQVQK	S(5): 100.0	-0,35	0,26	-0,81	-0,06
Plekha6	P16014	WASSREDAGAPVEDSQGQTK	S(3): 50.0; S(4): 50.0; S(15): 0.0; T(19): 0.0	0,93	-0,09	-0,77	1,41
Plekha6	Q7TQG1	SIHEVDISNLEAALR	S(1): 100.0; S(8): 0.0	-0,39	-0,07	-0,87	0,61
Polr2a	Q8K019-2	ADGDWDDQEVLDYFSDKESAK	Y(13): 50.0; S(15): 50.0; S(19): 0.0	0,00	0,00	-0,55	0,55
Polr2a	P08775	YSPTSPTYSPTSPK	Y(1): 0.5; S(2): 0.5; T(4): 98.6; S(5): 0.5; T(7): 0.0; Y(8): 0.0; S(9): 0.0; T(11): 0.0; S(12): 0.0	-0,47	0,30	-0,55	0,34
Ppfia3	B8QI35	RGSALGPDEAGGELER	S(3): 100.0	-0,35	0,48	0,01	0,10
Ppfia3	B8QI35	GEGPAVPGDTPPPTPR	T(10): 100.0; T(14): 0.0	-0,18	0,14	-0,26	0,24
Ppfia3	B8QI35	ESVSLAGTPSDETLATDPLGLAK	S(2): 0.0; S(4): 0.0; T(8): 0.6; S(10): 0.6; T(13): 98.7; T(16): 0.1	0,23	-0,08	-0,38	0,06
Prkcb	P68254	TAFDEAIAELDTLNEDSYK	T(1): 0.0; T(12): 0.0; S(17): 99.2; Y(18): 0.8	-0,22	0,47	0,27	-0,17
Prkcb	P68404-2	NIDQSEFEGFSFVNSEFLKPEVK	S(5): 0.0; S(11): 50.0; S(15): 50.0	-1,33	0,17	0,49	-0,53
Prpf40b	D3Z4N6	TGWDTSESELSEGELER	T(1): 0.0; T(5): 0.0; S(6): 0.0; S(8): 0.0; S(11): 100.0	0,46	-0,01	-0,47	-0,37
Ptchd3	Q0EEE2	GFIQIVDVSSSSNK	S(9): 25.0; S(10): 25.0; S(11): 25.0; S(12): 25.0	-1,32	0,55	0,45	-0,75
Purb	O35295	DSLGDFIEHYAQLGPSSPEQLAAGAEEGGGPR	S(2): 0.0; Y(10): 0.0; S(16): 8.3; S(17): 91.7	0,43	-0,05	-0,53	0,24
Pvrl1	Q9JKF6	AGIPQHHPPMAQNLQYPDDSDDEK	Y(16): 0.7; S(20): 99.3	0,60	-0,69	-0,53	0,16
Rapgef2	E9QNQ4	SLGSLSQGSANATVLDVAQTGGHK	S(1): 97.9; S(4): 2.0; S(6): 0.1; S(9): 0.1; T(13): 0.0; T(20): 0.0	0,72	-0,24	-0,48	-0,08
Rgl3	Q3UYI5	NREPPPPGSPPASPGPQSPSTK	S(9): 100.0; S(13): 0.0; S(18): 0.0; S(20): 0.0; T(21): 0.0	-0,94	0,18	1,44	-1,37
Rgl3	Q3UYI5	EPPPPGSPPASPGPQSPSTK	S(7): 100.0; S(11): 0.0; S(16): 0.0; S(18): 0.0; T(19): 0.0	0,41	-0,16	0,01	0,03
Rph3a	P47708	WHQLQNENHVSSD	S(11): 50.0; S(12): 50.0	-0,11	0,10	0,36	-0,25
Rph3a	P47708	TKPQQPAGEPATQEQPTPESR	T(1): 0.0; T(12): 0.0; T(17): 99.4; S(20): 0.6	0,31	-0,65	0,32	-0,48
Rph3a	P47708	WHQLQNENHVSSD	S(11): 0.3; S(12): 99.7	0,39	-0,14	0,31	-0,41
Rph3a	P47708	TKPQQPAGEPATQEQPTPESR	T(1): 0.0; T(12): 0.0; T(17): 99.6; S(20): 0.4	-0,04	-0,10	0,05	-0,45
Rps6kc1	Q8BLK9-2	GVDLLLEGVQGESSPTR	S(13): 49.9; S(14): 49.9; T(16): 0.2	-0,44	0,35	-0,40	0,00
Rtn4	Q99P72	DDSPKEYTDLEVSNK	S(3): 100.0; Y(7): 0.0; T(8): 0.0; S(13): 0.0	0,62	-0,44	0,00	-0,06
Rtn4	Q99P72	LSASPQEVGKPYLESFQPNLHITK	S(2): 0.0; S(4): 100.0; Y(12): 0.0; S(15): 0.0; T(23): 0.0	-0,36	-0,03	-0,09	-0,10
Rtn4	Q99P72	ESAEFSVLEYSEMGSSFNGSPK	S(2): 0.0; S(6): 0.0; Y(10): 0.0; S(11): 0.0; S(15): 0.1; S(16): 0.9; S(20): 99.0	-0,01	-0,48	-0,43	0,01
Rtn4	Q9QYR6	ETSPTRGEPVPAWEGK	T(2): 0.4; S(3): 99.6; T(5): 0.0	0,02	0,30	0,04	0,38
Ryr2	E9Q401	RISQTSQVSIDAAHGYSPR	S(3): 100.0; T(5): 0.0; S(6): 0.0; S(9): 0.0; Y(16): 0.0; S(17): 0.0	0,88	-0,88	0,40	-0,29
Ryr2	E9Q401	RISQTSQVSIDAAHGYSPR	S(3): 99.6; T(5): 0.4; S(6): 0.0; S(9): 0.0; Y(16): 0.0; S(17): 0.0	0,63	-0,64	0,25	-0,02
Ryr2	E9Q401	EAAVPEEEGGTPEKEISIEDAK	T(11): 100.0; S(17): 0.0	0,31	-0,77	0,17	-0,46
Scap	Q6GQT6	RDSCGGGAFETQENWER	S(3): 100.0; T(11): 0.0	0,51	-0,81	1,02	-0,71
Serpinb6a	F8WIV2	LTIMDPLQEANGTFALNLLK	T(2): 50.0; T(13): 50.0	-0,55	0,21	-0,21	0,19
Serpinb6a	F8WIV2	LTIMDPLQEANGTFALNLLK	T(2): 50.0; T(13): 50.0	-0,46	-0,01	-0,59	-0,21
Sfr1	Q8BP27-2	ENPPSPHSNSSGK	S(5): 100.0; S(8): 0.0; S(10): 0.0; S(11): 0.0	0,40	-0,22	0,66	-0,34
Shisa6	F6VQZ6	QYNHPILSSATQTPTHEKPR	Y(2): 0.0; S(8): 0.0; S(9): 0.0; T(11): 0.0; T(13): 100.0; T(15): 0.0	0,52	-0,95	-0,74	0,30
Skiv2l	Q6NZR5	LLEPLDLSGGDEDEGEAAGGPR	S(8): 100.0	-0,49	-0,08	-0,52	0,20
Slc12a5	Q91V14	ESSPFINSTDTEK	S(2): 7.1; S(3): 92.9; S(8): 0.0; T(9): 0.0; T(11): 0.0	-0,15	-0,10	-0,37	0,18
Slc12a5	Q91V14	GPSPVSSEGIK	S(3): 100.0; S(6): 0.0; S(7): 0.0	-0,07	-0,06	-0,38	-0,19
Slc12a5	Q91V14	NKGPSPVSSEGIK	S(5): 100.0; S(8): 0.0; S(9): 0.0	-0,64	0,27	-0,53	0,06
Slc12a5	Q91V14	ESSPFINSTDTEK	S(2): 50.0; S(3): 50.0; S(8): 0.0; T(9): 0.0; T(11): 0.0	-0,06	0,01	-0,53	0,18
Slc43a2	Q8CGA3	LCLSTVDLEVK	S(4): 50.0; T(5): 50.0	1,41	-0,29	0,77	-0,78
Slc4a4	E9Q8N8	MFSNPDNGSPAMTHR	S(3): 0.0; S(9): 99.8; T(13): 0.2	-0,57	0,38	-0,37	0,13
Slc4a4	E9Q8N8	MFSNPDNGSPAMTHR	S(3): 0.0; S(9): 95.2; T(13): 4.8	-0,04	0,01	-0,41	-0,03
Slc6a11	P31650	GSETLGGGGGGAAGTR	S(2): 100.0; T(4): 0.0; T(15): 0.0	-0,58	0,82	-1,12	-0,36
Sltm	Q8CH25-2	DVQDAIAQSPEK	S(9): 100.0	0,59	-0,20	0,64	-0,45

Disease Models & Mechanisms • Supplementary information

Sp4	K4DI62	ENNVSQPASSSSSSSSSNNGSSSPTK	S(5): 0.0; S(9): 0.0; S(10): 0.0; S(11): 0.0; S(12): 0.0; S(13): 0.0; S(14): 0.0; S(15): 0.0; S(16): 0.0; S(17): 0.0; S(21): 0.4; S(22): 6.1; S(23): 87.3; T(25): 6.1	-0,53	0,53	0,38	-0,39
Sptbn1	Q62261	TLETPAAQMEGFLNR	T(1): 0.0; T(4): 100.0	0,38	-0,26	0,50	-0,13
Sptbn1	Q62261	AQTLPTSVVTITSESSPGKR	T(3): 0.0; T(6): 0.0; S(7): 0.0; T(10): 0.0; T(12): 0.2; S(13): 33.3; S(15): 33.3; S(16): 33.3	-0,51	0,39	-0,02	-0,12
Sptbn2	Q68FG2	SSEAAHGATLPTR	S(1): 100.0; S(2): 0.0; T(9): 0.0; T(12): 0.0	0,44	-0,63	0,04	-0,47
Sptbn2	Q68FG2	LEQSNVPEGPGSGTGDESSGPR	S(4): 0.0; S(12): 99.6; T(14): 0.4; S(18): 0.0; S(19): 0.0	-0,35	-0,10	-0,03	-0,48
Sptbn2	Q68FG2	WDLPDSDWDNDSSSAR	S(6): 100.0; S(12): 0.0; S(13): 0.0; S(14): 0.0	-0,08	0,23	-1,07	-0,37
Sptbn4	E9PX29	QESADHEGPHSLTLGR	S(3): 100.0; S(11): 0.0; T(13): 0.0	0,73	-0,82	0,36	-0,18
Srgap1	Q91Z69	RPGHGSLTNISR	S(6): 100.0; T(8): 0.0; S(11): 0.0	-0,52	-0,16	0,47	-0,56
Srrm2	Q8BTI8	SRTPPSAPSQSR	S(1): 0.0; T(3): 100.0; S(6): 0.0; S(9): 0.0; S(11): 0.0	0,21	-0,40	-0,06	0,21
Srrm2	Q8BTI8	SPVPSAFSDQSR	S(1): 100.0; S(5): 0.0; S(8): 0.0; S(11): 0.0	-0,42	0,23	-0,19	0,12
Srrm2	Q8BTI8	MVQASSQSLLPPAQDRPRSPVPSAFSDQSR	S(5): 0.0; S(6): 0.0; S(8): 0.0; S(19): 100.0; S(23): 0.0; S(26): 0.0; S(29): 0.0	0,06	-0,40	-0,21	0,03
Srrm2	Q8BTI8	SGTPPRPGSVTNMQADECTATPQR	S(1): 0.6; T(3): 99.4; S(9): 0.0; T(11): 0.0; T(19): 0.0; T(21): 0.0	0,40	-0,02	-0,25	0,29
Srrm2	Q8BTI8	SGMSPEQSK	S(1): 0.0; S(4): 100.0; S(8): 0.0	-0,06	-0,02	-0,38	0,29
Stxbp1	O08599-2	ESSRVSFEDQAPTME	S(2): 0.0; S(3): 0.0; S(6): 99.9; T(13): 0.0	-0,64	0,67	0,73	-0,53
Svop	Q8BFT9	TGESARSEDDAASGEHDIQIEGVR	T(1): 1.6; S(4): 49.2; S(7): 49.2; S(13): 0.1	-0,56	0,44	-0,46	-0,27
Svop	Q8BFT9	TGESARSEDDAASGEHDIQIEGVR	T(1): 0.0; S(4): 50.0; S(7): 50.0; S(13): 0.0	-0,67	0,49	-0,64	-0,26
Syn1	O88935	QTSQQPAGPPAQQRPPPQGGPPQPGPGPQR	T(2): 15.6; S(3): 84.4	0,62	-1,67	0,80	-0,73
Syn1	O88935	QSRPVAGGPGAPPAARPPASPSPQR	S(2): 0.0; S(20): 1.2; S(22): 98.8	0,33	-0,08	0,49	-0,31
Syn1	O88935	SQSLTNAFNLPEPAPPRPSLSQDEVK	S(1): 0.0; S(3): 0.0; T(5): 0.0; S(19): 50.0; S(21): 50.0	0,51	-0,14	0,48	-0,27
Syn1	O88935	QASISGPAPTK	S(3): 100.0; S(5): 0.0; T(10): 0.0	0,18	-0,50	0,34	-0,47
Syn1	O88935	QSRPVAGGPGAPPAARPPASPSPQR	S(2): 0.0; S(20): 1.1; S(22): 98.9	0,30	-0,42	0,17	0,37
Tanc1	E9QAF9	RADNCSPVAEEETTGSAESVLPK	S(6): 100.0; T(13): 0.0; T(14): 0.0; S(16): 0.0; S(19): 0.0	0,09	0,11	0,46	-0,28
Tanc1	E9QAF9	ADNCSPVAEEETTGSAESVLPK	S(5): 100.0; T(12): 0.0; T(13): 0.0; S(15): 0.0; S(18): 0.0	0,70	0,19	0,28	-0,01
Tanc1	E9QAF9	QQGPPPAPANDSDNEEDAPASSLK	S(12): 100.0; S(21): 0.0; S(22): 0.0	0,39	-0,31	0,03	-0,15
Tanc1	E9QAF9	QQGPPPAPANDSDNEEDAPASSLK	S(12): 100.0; S(21): 0.0; S(22): 0.0	-0,15	0,55	-0,62	-0,13
Tom1l2	Q5SRX1	AAETVPDLPSPPTEAPAPASNTSTR	T(4): 0.0; S(10): 38.8; T(13): 38.8; S(20): 1.4; T(22): 7.0; S(23): 7.0; T(24): 7.0	0,23	0,79	0,95	-0,11
Tom1l2	Q5SRX1	GIEFPMADLDALSPIHTPQR	S(13): 100.0; T(17): 0.0	1,06	0,01	0,67	0,80
Tom1l2	Q5SRX1	AAETVPDLPSPPTEAPAPASNTSTR	T(4): 0.0; S(10): 50.0; T(13): 50.0; S(20): 0.0; T(22): 0.0; S(23): 0.0; T(24): 0.0	0,52	-0,86	0,17	0,24
Tom1l2	Q5SRX1	GIEFPMADLDALSPIHTPQR	S(13): 100.0; T(17): 0.0	0,74	-0,43	0,10	-0,63
Top2b	Q64511	KASGSENEGDYNPGR	S(3): 50.0; S(5): 50.0; Y(11): 0.0	-0,12	0,09	0,59	0,19
Top2b	Q64511	KTSFDQDSDVDIFPSDFTSEPPALPR	T(2): 0.0; S(3): 0.0; S(8): 100.0; S(15): 0.0; T(18): 0.0; S(19): 0.0	-0,59	0,03	-0,13	-0,17
Top2b	Q3UID0	DMDEPSPVPNVEEVTLPK	S(6): 100.0; T(15): 0.0	0,33	0,08	-0,69	0,41
Top2b	Q64511	FDSNEEDTASVFAPSFGLK	S(3): 100.0; T(8): 0.0; S(10): 0.0; S(15): 0.0	-0,42	0,44	-0,69	0,60
Transmembrane protein C15orf27 homolog	Q8BZB3	ALDPAPLAQPTPLGSVQTSPELEHR	T(11): 0.0; S(15): 0.0; T(18): 50.0; S(19): 50.0	-0,54	0,00	-0,42	-0,19
Trim28	E9Q6J5	GSDDVLVSGEVPECEVGHMSPR	S(2): 0.0; S(8): 0.0; S(20): 100.0	-0,38	-0,07	-0,50	0,66
Trim28	Q62318	RPAASSAAAASAAASSPAGGGGEAQELLEHCGVCR	S(5): 0.0; S(6): 0.0; S(11): 0.0; S(15): 50.0; S(16): 50.0	-0,37	0,77	-0,50	0,73
Ube4b	Q9ES00	LAGGQTSQPTTPLTSPQR	T(6): 0.0; S(7): 0.0; T(10): 0.0; T(11): 0.0; T(14): 50.0; S(15): 50.0	0,68	-0,09	0,75	-0,03
Uty	D6RFT4	GSEVQSIIIK	S(2): 0.0; S(6): 100.0	0,64	-0,71	-0,42	0,06
Vdac1	Q8K284-2	VAASPRPGAEEQAEAQAPAQLAAPEDADAGGPR	S(4): 100.0	-0,57	0,06	-0,43	0,03
Vdac1	Q60932-2	LTFDSSFSPNTGK	T(2): 0.0; S(5): 0.5; S(6): 0.5; S(8): 98.5; T(11): 0.5	0,25	0,01	-0,44	1,55
Vill	D3Z669	TVVSVFPGNNK	T(1): 93.3; S(4): 6.7	-0,68	0,05	-0,40	-0,13

Ywhaz	P63101	TAFDEAIAELDTLSEESYK	T(1): 0.0; T(12): 0.1; S(14): 99.7; S(17): 0.1; Y(18): 0.1	-0,51	0,55	0,02	-0,40
Ywhaz	P63101	TAFDEAIAELDTLSEESYKDSTLIMQLLR	T(1): 0.0; T(12): 49.9; S(14): 49.9; S(17): 0.1; Y(18): 0.1; S(21): 0.0; T(22): 0.0	0,09	0,30	-0,37	0,85
Zswim8	Q3UHH1-3	KQSAGPNSPTGGGGGGGGSGGTR	S(3): 0.0; S(8): 99.8; T(10): 0.2; S(18): 0.0; T(21): 0.0	-0,56	0,92	0,82	0,02

Disease Models & Mechanisms • Supplementary information

Supplementary Table S10: Phosphoproteins common to both models Tg(*Dyrk1a*) and Ts65Dn in each region of hippocampus, cortex and cerebellum.

Hippocampus	Cortex	Cerebellum
Aak1	Aak1	Aak1
Ace	Adcy5	Acot11
Akap5	Agap2	Akap12
Aldoa	Akt2	Akt1
Arhgef2	Aldoc	Akt2
Arhgef28	Amot	Amph
Basp1	Ap2a1	Ap3d1
Bcmo1	Ap4e1	Appl1
Bsn	Apc	Arfgap1
C2cd2l	Arhgap21	Arhgap21
Cacna1a	Arhgap35	Arhgef12
Camkv	Asap1	Arvcf
Caskin1	Atp1a1	Atp1a3
Cep170b	Atp1a3	Brsk1
Chgb	Atp4a	Bsn
Clip2	Atp8a1	Cacna1g
Clns1a	Atxn2l	Camsap2
Col3a1	Avl9	Caskin1
Col4a1	Begain	Caskin2
Dmtn	Bod1l	Ckb
Dnah7b	Braf	Clns1a
Eif2ak4	Cacna1b	Coro1a
Fam169a	Camk2d	Cstf2t
Fam171a2	Cep170b	Ctage5
Foxk1	Chga	Dagla
Gbf1	Col3a1	Dbi
Gm14597	Comt	Dclk1
Gpr123	Ctnnd2	Dmtn
Grin2b	Ctps1	Dmxl2
Hcn2	D430041D05Rik	Dnajc6
Hepacam	Dchs1	Dock4
Hn1	Ehd3	Dsg2
Hnrnpa0	Eml2	Efhd2
Huwe1	Fam208a	Eif4b
Krt26	Gkn1	Epn1
Lmna	Gm10277	Epn2
Map1a	Gm15800	Fam134a
Map1b	Gm4951	Fam169a
Map2	Gm996	Fam171a2
Marcks	Gprin1	Fxr1

Disease Models & Mechanisms • Supplementary information

Marcksl1	Hepacam	Gja1
Mccc2	Kcnb1	Gm2933
Me1	Klc2	Gm996
Mtdh	Lmtk3	Gprin1
Mug2	Lppr2	Gpsm1
Mycbpap	Lppr4	Gramd1b
Myo18a	Mag	Gtf3c1
Nap1l4	Map1a	Hcn2
Ncl	Map1b	Hist1h4a
Nefh	Map2	Htt
Nefm	Map3k7	Ifi44
Nktr	Mfsd9	Il16
Npnt	Napa	Itih2
Nsmf	Nf1	Jph3
Nudt3	Npm1	Kcnc1
Pacsin1	Nup210	Kif17
Pak3	Osbp	Kif2a
Palmd	Pcdh1	Lgalsl
Pcdh11x	Pclo	Lnp
Pclo	Ppp1r1b	Lzts3
Pp2d1	Ppp1r3g	Map1a
Ppm1g	Pycard	Map1b
Ppp1r18	Rsph3b	Map2
Ppp6r3	Rtn1	Marcks
Prkcb	Rtn4	Mier1
Prrc2c	Scfd1	Mobp
Prrt2	Sec22b	Mpdz
Rab3a	Sin3a	Mprip
Rbbp8	Sipa1l1	Mroh2a
Reps2	Sirt2	Myo5a
Rgs14	Slc7a8	N4bp3
Rltpr	Snx17	Npm1
Rnf20	Snx5	Nucks1
Scg2	Syn1	Oxct1
Sdc4	Syngap1	Oxr1
Shank1	Synj1	Park2
Sipa1l3	Tbc1d5	Pclo
Sirt2	Tekt4	Phf3
Slc12a5	Tmem163	Phkb
Slc1a3	Tmsb4x	Pianp
Slc1a4	Trappc8	Pkp4
Slc7a14	Ube4b	Plcl2
Slc9a6	Uncharacterized protein C15orf39 homolog	Plekha6
Sparcl1	Usp10	Ppfia3

Disease Models & Mechanisms • Supplementary information

Specc1	Usp45	Prkcb
Sptbn1	Yap1	Prpf40b
Srrm2	Ywhaz	Ptchd3
Stim1		Purb
Syk		Pvrl1
Syn1		Rabl6
Synpr		Rapgef2
Tcp11l1		Rgl3
Tmem161a		Rps6kc1
Tom1		Rtn4
Tppp		Ryr2
Tsc22d2		Scaf8
Tsg101		Scap
Ube2w		Serpinb6a
Unc13a		Sfr1
Utp20		Shisa6
Wfs1		Skiv2l
Wwp1		Slc12a5
Zdhhc5		Slc43a2
Zswim8		Slc4a4
		Slc6a11
		Slc9a1
		Sltm
		Sp4
		Sptbn1
		Sptbn2
		Sptbn4
		Srgap1
		Srrm1
		Srrm2
		Stxbp1
		Svop
		Syn1
		Tanc1
		Tom1l2
		Top2b
		Transmembrane protein C15orf27 homolog
		Trim28
		Ube4b
		Uncharacterized protein C18orf25 homolog
		Uty
		Vdac1
		Vill
		Ywhaz
		Zfp292
		Zswim8

Disease Models & Mechanisms • Supplementary information

Supplementary Table S11: Biological processes altered by the common proteins up- and down-phosphorylated in the hippocampus of Tg(*Dyrk1a*) and Ts65Dn mice, and reverted by L41 treatment.
ToppCluster analysis.

ID	GO : Biological Process	TgDyrk1a logP	Ts65Dn logP	TgDyrk1a	Ts65Dn
		pValues		Gene List	
GO:0007268	synaptic transmission	10.0000	10.0000	AKAP5 BSN CACNA1A EIF2AK4 GRIN2B HCN2 MYCBPAP NSMF PCLO PRKCB RAB3A RGS14 SHANK1 SLC12A5 SLC1A3 SLC1A4 SYN1 UNC13A	AP2A1 APBA1 APOE ATP1A2 BSN CACNB2 CASK EIF2AK4 GRIN2B GRIP1 HCN2 JPH3 KCNA2 KCNAB2 MYCBPAP NEFL NSMF PCLO PRKCB PRKCE RAB3A RGS14 SIPA1L1 SLC1A4 SLC6A11 STX1A
GO:0007010	cytoskeleton organization	10.0000	10.0000	ALDOA ARHGEF2 ARHGEF28 DMTN DNAH7 LMNA MAP1A MAP1B MAP2 MYO18A NEFH NEFM PACSIN1 PAK3 PCLO RGS14 SDC4 SHANK1 SIRT2 SPTBN1 TPPP	ABI2 ADD1 ALDOA APOE ARFIP2 ARHGEF2 ARHGEF28 CROCC DMTN DNAI2 ENAH FRYL IQSEC2 KLHL41 LMNA MAP1A MAP1B NEFL PACSIN1 PCLO PRKCE RGS14 SDC4 SIPA1L1 SIRT2 SON SORBS1 SPTBN1 WDR62 WIPF2
GO:0007267	cell-cell signaling	5.4650	10.0000	AKAP5 BSN C2CD2L CACNA1A EIF2AK4 GRIN2B HCN2 MARCKS MYCBPAP NSMF NUDT3 PCLO PRKCB RAB3A RGS14 SHANK1 SLC12A5 SLC1A3 SLC1A4 SYN1 UNC13A	AP2A1 APBA1 APOE ATP1A2 BSN C2CD2L CACNB2 CASK EIF2AK4 GAL GRIN2B GRIP1 HCN2 JPH3 KCNA2 KCNAB2 MYCBPAP NEFL NSMF NUDT3 PCLO PRKAR2A PRKAR2B PRKCB PRKCE RAB3A RGS14 SIPA1L1 SLC1A4 SLC6A11 STX1A VPS35
GO:0048812	neuron projection morphogenesis	5.8279	4.9981	AKAP5 ARHGEF28 CACNA1A COL3A1 COL4A1 EIF2AK4 GRIN2B MAP1B MAP2 NEFH NSMF PACSIN1 PAK3 RAB3A SHANK1 SLC9A6 SPTBN1 UNC13A	AP2A1 APOE ARHGAP35 ARHGEF12 ARHGEF28 CACNB2 COL3A1 COL4A1 EIF2AK4 ENAH GRIN2B MAP1B NEFL NSMF PACSIN1 PGRMC1 PVRL1 RAB3A RASAL1 SIPA1L1 SLC9A6 SPTBN1 USP9X VAV3
GO:0032940	secretion by cell	10.0000		AKAP5 ALDOA C2CD2L CACNA1A DMTN GRIN2B MARCKS MYO18A NCL PCLO PRKCB RAB3A RLTPR SCG2 SDC4 SLC1A3 SPTBN1 SYK SYN1 TSG101 UNC13A	
GO:0031175	neuron projection development		10.0000		ABI2 AP2A1 APOE ARHGAP35 ARHGEF12 ARHGEF28 ATCAY CACNB2 COL3A1 COL4A1 EIF2AK4 ENAH FRYL GPRIN1 GRIN2B GRIP1 ITM2C MAP1B NEFL NSMF PACSIN1 PGRMC1 PVRL1 RAB3A RASAL1 SEPT2 SIPA1L1 SLC9A6 SPTBN1 USP9X VAV3
GO:1903530	regulation of secretion by cell	10.0000		AKAP5 C2CD2L CACNA1A GRIN2B MARCKS MYO18A NCL PCLO PRKCB RAB3A RLTPR SDC4 SPTBN1 SYK SYN1 TSG101 UNC13A	

Disease Models & Mechanisms • Supplementary information

GO:0051046	regulation of secretion	10.0000		AKAP5 C2CD2L CACNA1A GRIN2B MARCKS MYO18A NCL PCLO PRKCB RAB3A RLTPR SDC4 SPTBN1 SYK SYN1 TSG101 UNC13A	
GO:0046903	secretion	10.0000		ACE AKAP5 ALDOA C2CD2L CACNA1A DMTN GRIN2B MARCKS MYO18A NCL PCLO PRKCB RAB3A RLTPR SCG2 SDC4 SLC1A3 SPTBN1 SYK SYN1 TSG101 UNC13A	
GO:0060341	regulation of cellular localization	10.0000		AKAP5 C2CD2L CACNA1A DMTN GBF1 GRIN2B HUWE1 LMNA MAP1B MARCKS MYO18A NCL NEFH PCLO PRKCB RAB3A RLTPR SDC4 SIRT2 SPTBN1 SYK SYN1 TSG101 UNC13A	
GO:0006865	amino acid transport		10.0000		APBA1 ATP1A2 GRIN2B RAB3A SEPT2 SLC17A6 SLC1A4 SLC6A11 SLC7A14 SLC7A8 STX1A
GO:0048666	neuron development		5.6658		ABI2 AP2A1 APOE ARHGAP35 ARHGEF12 ARHGEF28 ATCAY CACNB2 COL3A1 COL4A1 EIF2AK4 ENAH FRYL GPRIN1 GRIN2B GRIP1 ITM2C MAP1B NEFL NSMF PACSIN1 PGRMC1 PVRL1 RAB3A RASAL1 SDC4 SEPT2 SIPA1L1 SLC9A6 SPTBN1 USP9X VAV3
GO:0016358	dendrite development	5.5182		AKAP5 CACNA1A MAP1B MAP2 NSMF PACSIN1 PAK3 SHANK1 SLC9A6	
GO:0046942	carboxylic acid transport		5.5116		ACE APBA1 APOE ATP1A2 GRIN2B RAB3A SEPT2 SLC17A6 SLC1A4 SLC6A11 SLC7A14 SLC7A8 STX1A SYK
GO:0017157	regulation of exocytosis	5.4720		CACNA1A PCLO RAB3A SDC4 SYK SYN1 TSG101 UNC13A	
GO:0015849	organic acid transport		5.4651		ACE APBA1 APOE ATP1A2 GRIN2B RAB3A SEPT2 SLC17A6 SLC1A4 SLC6A11 SLC7A14 SLC7A8 STX1A SYK
GO:0007015	actin filament organization		5.2844		ABI2 ADD1 ALDOA ARFIP2 ARHGEF2 DMTN ENAH FRYL PACSIN1 PRKCE SDC4 SORBS1 SPTBN1 WIPF2
GO:0050808	synapse organization	5.2328		BSN CACNA1A COL4A1 PAK3 PCLO RAB3A SHANK1 SLC9A6 UNC13A	

Disease Models & Mechanisms • Supplementary information

GO:0006835	dicarboxylic acid transport		5.2268		APBA1 ATP1A2 GRIN2B RAB3A SEPT2 SLC17A6 SLC1A4 STX1A
GO:0015711	organic anion transport		5.1795		ACE APBA1 APOE ATP1A2 ATP8A1 GRIN2B RAB3A SEPT2 SLC17A6 SLC1A4 SLC4A4 SLC6A11 SLC7A14 SLC7A8 STX1A SYK
GO:1903532	positive regulation of secretion by cell	5.1216		C2CD2L GRIN2B MYO18A NCL RAB3A RLTPR SDC4 SPTBN1 SYK TSG101 UNC13A	
GO:0050804	modulation of synaptic transmission	5.0345		CACNA1A EIF2AK4 GRIN2B NSMF RAB3A RGS14 SHANK1 SLC1A3 SYN1 UNC13A	
GO:0050767	regulation of neurogenesis	5.0074		ACE AKAP5 ARHGEF2 CACNA1A COL3A1 EIF2AK4 MAP1B MAP2 NSMF PACSIN1 PAK3 RGS14 SHANK1 SIRT2 UNC13A	
GO:0071705	nitrogen compound transport		4.9578		APBA1 ATP1A2 C2CD2L GAL GRIN2B HNRNPA1 KCNA2 MYO1C PCLO PRKAR2A PRKAR2B PRKCB PRKCE RAB3A SEPT2 SLC17A6 SLC1A4 SLC6A11 SLC7A14 SLC7A8 SMG6 STX1A SYK
GO:0048858	cell projection morphogenesis	4.9237		AKAP5 ARHGEF28 CACNA1A COL3A1 COL4A1 DNAH7 EIF2AK4 GRIN2B MAP1B MAP2 NEFH NSMF PACSIN1 PAK3 RAB3A SHANK1 SLC9A6 SPTBN1 UNC13A	
GO:0050806	positive regulation of synaptic transmission	4.8784		EIF2AK4 GRIN2B RAB3A RGS14 SHANK1 SLC1A3 UNC13A	
GO:0032990	cell part morphogenesis	4.8217		AKAP5 ARHGEF28 CACNA1A COL3A1 COL4A1 DNAH7 EIF2AK4 GRIN2B MAP1B MAP2 NEFH NSMF PACSIN1 PAK3 RAB3A SHANK1 SLC9A6 SPTBN1 UNC13A	
GO:0051047	positive regulation of secretion	4.7964		C2CD2L GRIN2B MYO18A NCL RAB3A RLTPR SDC4 SPTBN1 SYK TSG101 UNC13A	
GO:0048667	cell morphogenesis involved in neuron differentiation	4.7810		AKAP5 ARHGEF28 CACNA1A COL3A1 COL4A1 GRIN2B MAP1B MAP2 NEFH NSMF PAK3 RAB3A SHANK1 SLC1A3 SLC9A6 SPTBN1	
GO:0060052	neurofilament cytoskeleton organization			ARHGEF28 NEFH NEFM	
		4.7808			

Disease Models & Mechanisms • Supplementary information

Supplementary Table S12: Biological processes altered by the common proteins up- and down-phosphorylated in the cortex of Tg(*Dyrk1a*) and Ts65Dn mice, and reverted by L41 treatment. ToppCluster analysis.

ID	GO: Biological Process	TgDyrk1a logP	Ts65Dn logP	TgDyrk1a	Ts65Dn
		PValues		Gene List	
GO:0032990	cell part morphogenesis	10.0000	10.0000	ABLIM3 AGAP2 AHI1 AP2A1 APC ARHGAP39 ASAP1 BRSK1 CAMK2D COL3A1 COL4A3BP CRMP1 CTNND2 DBN1 DBNL DPYSL5 GAP43 GDI1 IL17RD MAG MAP1B MAP1S MAP2 MAP2K1 MARK2 MYCBP2 MYO9B NEDD4L NF1 PSMD1 PSMD2 RAB3A RTN4 SIPA1L1 SLC9A6 SPTBN1 SRCIN1 SYNGAP1 TBC1D15 TNIK TOP2B	ABLIM3 AGAP2 AP2A1 APC ARHGAP39 ASAP1 BCL11B CAMK2D EHD3 GRIN2A GRIN2B HTT LRFN1 MAP1B MAP2 MARK3 NF1 NUMBL PTPN23 RIMS1 RTN4 SIPA1L1 SRC SYNGAP1 TBC1D15 TBC1D5 TIAM1
GO:0007268	synaptic transmission	10.0000	10.0000	ADCY5 ADCY9 AP2A1 APBA1 BRSK1 CACNA1B CACNA1E CAMK2D CSPG5 CTNNB1 CTNND2 DBN1 DNAJC5 HRH3 KCNB1 NF1 PCLO PDPK1 PPFIA4 RAB3A SIPA1L1 SLC6A11 SLC8A2 SYN1 SYN2 SYN3 SYNGAP1 SYNJ1	AMPH AP2A1 BCAN BSN CAMK2D COMT CPLX1 GNG3 GRIN2A GRIN2B HCN1 HTT KCNA2 KCNB1 NAPA NF1 PCLO PDPK1 PRKCB RIMS1 SIPA1L1 SYN1 SYNGAP1 SYNJ1
GO:0048858	cell projection morphogenesis	10.0000	10.0000	ABLIM3 AGAP2 AHI1 AP2A1 APC ARHGAP39 ASAP1 BRSK1 CAMK2D COL3A1 CRMP1 CTNND2 DBN1 DBNL DPYSL5 GAP43 GDI1 IL17RD MAG MAP1B MAP1S MAP2 MAP2K1 MARK2 MYCBP2 MYO9B NEDD4L NF1 PSMD1 PSMD2 RAB3A RTN4 SIPA1L1 SLC9A6 SPTBN1 SRCIN1 SYNGAP1 TBC1D15 TNIK TOP2B	ABLIM3 AGAP2 AP2A1 APC ARHGAP39 ASAP1 BCL11B CAMK2D EHD3 GRIN2A GRIN2B HTT LRFN1 MAP1B MAP2 MARK3 NF1 NUMBL PTPN23 RIMS1 RTN4 SIPA1L1 SRC SYNGAP1 TBC1D15 TBC1D5 TIAM1
GO:0007264	small GTPase mediated signal transduction	10.0000	10.0000	ABR AGAP2 AMOT ARHGAP33 ARHGAP39 ARHGEF10 ARHGEF2 CAMK2D COL3A1 CTNNB1 DBNL DOCK5 FMNL1 G3BP2 GARNL3 GDI1 IL17RD MAP2K1 MKL1 MYO9B NF1 PDCD10 PDPK1 PKN2 PSD3 PSMD1 PSMD2 RAB3A RALGAPA1 RALGAPB REM2 SIPA1L1 SPTBN1 STARD8 SYNGAP1 TAOK1 YWHAZ	AGAP2 AMOT ARFGEF1 ARHGAP39 ARHGEF2 CAMK2D FMNL1 GRIN2A GRIN2B IQSEC3 KIF2A KLC2 MARK3 NF1 PDPK1 PKN2 REM2 SIPA1L1 SRC SYNGAP1 TIAM1 YWHAZ
GO:0051640	organelle localization	10.0000	10.0000	AHI1 ARHGAP21 ARHGEF2 CHGA CTNNB1 DNAJC5 MAP1B MAP1S MAP2K1 MAP4 MARK2 NPM1 PCLO PDCD10 PDPK1 RAB3A SEC22B SEC24D SYN1 SYNJ1 YWHAZ	AMPH ARHGAP21 ARHGEF2 CHGA CPLX1 HTT MAP1B NAPA NPM1 PCLO PDPK1 RIMS1 SCFD1 SEC22B SYN1 SYNJ1 YWHAZ
GO:0051656	establishment of organelle localization	10.0000	10.0000	AHI1 ARHGAP21 ARHGEF2 CTNNB1 DNAJC5 MAP1B MAP1S MAP2K1 MAP4 NPM1 PCLO PDCD10 RAB3A SEC22B SEC24D SYN1 SYNJ1 YWHAZ	AMPH ARHGAP21 ARHGEF2 CPLX1 HTT MAP1B NAPA NPM1 PCLO RIMS1 SCFD1 SEC22B SYN1 SYNJ1 YWHAZ
GO:1902580	single-organism cellular localization	10.0000	10.0000	ABLIM3 ARHGAP21 ARHGEF2 ATP8A1 C2CD5 CHGA CTNNB1 DBN1 DNAJC5 G3BP2 GDI1 GPR158 KCNB1 MAP1B MAP1S MAP2K1 MAP4 MAP7 NF1 NPM1 PACS1 PCLO PDPK1 RAB3A SEC22B SEC24D SLC9A1 SPTBN1 SULF2 SYN1 SYNGAP1 SYNJ1 TNIK YWHAZ	ABLIM3 AKT2 AMPH ARHGAP21 ARHGEF2 CHGA CPLX1 EHD3 GRIN2B HTT KCNB1 MAP1B NAPA NF1 NPM1 PACS1 PCLO PDE2A PDPK1 RIMS1 RPS3 SCFD1 SEC22B SYN1 SYNGAP1 SYNJ1 TIAM1 TRAPPC8 YWHAZ
GO:0007612	learning	10.0000	10.0000	ATP1A3 ATP8A1 CACNA1E CTNND2 HRH3 NF1 PDE1B PPP1R1B SLC8A2 SYNGAP1 SYNJ1	AMPH COMT GRIN2A GRIN2B HTT NF1 PDE1B PPP1R1B SYNGAP1 SYNJ1

Disease Models & Mechanisms • Supplementary information

GO:0060341	regulation of cellular localization	10.0000	10.0000	ABLIM3 ABR ADCY5 AHI1 APC C2CD2L C2CD5 CACNA1B CACNA1E CAMK2D CHGA CTNNB1 DBN1 G3BP2 GDI1 HRH3 KCNB1 MAP1B MAP2K1 NEDD4L NF1 PCLO PDPK1 PIK3C3 PYCARD RAB3A RALGAPB RTN4 SIRT2 SLC9A1 SNX12 SPTBN1 SRCIN1 SULF2 SYN1 SYNJ1 YWHAZ	ABLIM3 AKT2 APC ASTN2 CAMK2D CHGA CPLX1 GRIN2B HTT KCNA2 KCNB1 MAP1B NAPA NF1 PCLO PDE2A PDPK1 PKDREJ PRKCB PTPN23 RIMS1 RTN4 SCFD1 SRC SYN1 SYNJ1 TIAM1 YWHAZ
GO:0007010	cytoskeleton organization	10.0000	10.0000	ABLIM3 ABR AGAP2 AMOT APC ARHGEF10 ARHGEF2 BRSK1 CAMSAP1 CAMSAP2 CRMP1 CSNK1A1 CTNNB1 DBN1 DBNL FMNL1 MAP1A MAP1B MAP1S MAP2 MAP4 MAP7 MARK2 MYCBP2 NF1 NPM1 PCLO PDCD10 PDPK1 PYCARD SIPA1L1 SIRT2 SLC9A1 SPTBN1 STRIP1 SYNPO TAOK1 TNIK	ABLIM3 AGAP2 AMOT APC ARFGEF1 ARHGEF2 CDC42BPB EML2 FHOD1 FMNL1 HTT IQSEC3 KANK3 KIF2A MAP1A MAP1B MAP2 NF1 NPM1 PCLO PDPK1 RPS3 SCIN SIPA1L1 SRC SYNPO
GO:0007409	axonogenesis	10.0000	5.9321	ABLIM3 AGAP2 AP2A1 APC ARHGAP39 BRSK1 CAMK2D COL3A1 CRMP1 DBN1 DPYSL5 GAP43 GDI1 IL17RD MAG MAP1B MAP2 MAP2K1 MARK2 MYCBP2 NF1 PSMD1 PSMD2 RAB3A RTN4 SIPA1L1 SLC9A6 SPTBN1 SYNGAP1 TOP2B	ABLIM3 AGAP2 AP2A1 APC ARHGAP39 BCL11B CAMK2D GRIN2A GRIN2B LRFN1 MAP1B MAP2 MARK3 NF1 NUMBL RTN4 SIPA1L1 SRC SYNGAP1 TIAM1
GO:0048666	neuron development	10.0000	5.8393	ABLIM3 AGAP2 AHI1 AP2A1 APC ARHGAP39 ASAP1 BRSK1 CAMK2D CAMSAP1 CAMSAP2 COL3A1 CRMP1 CTNND2 DBN1 DBNL DPYSL5 GAP43 GBA2 GDI1 GPRIN1 IL17RD LRRC7 MAG MAP1B MAP1S MAP2 MAP2K1 MAP4 MARK2 MYCBP2 NEDD4L NF1 PSMD1 PSMD2 RAB3A RTN4 SIPA1L1 SLC9A6 SPTBN1 SRCIN1 SYNGAP1 TNIK TOP2B	ABLIM3 AGAP2 AP2A1 APC ARFGEF1 ARHGAP39 ASAP1 BCL11B CAMK2D GPRIN1 GRIN2A GRIN2B HCN1 HTT LRFN1 MAP1B MAP2 MARK3 NF1 NUMBL RIMS1 RTN4 SIPA1L1 SRC SYNGAP1 TIAM1 UBE4B
GO:0031175	neuron projection development	10.0000	5.7384	ABLIM3 AGAP2 AHI1 AP2A1 APC ARHGAP39 ASAP1 BRSK1 CAMK2D CAMSAP1 CAMSAP2 COL3A1 CRMP1 CTNND2 DBN1 DBNL DPYSL5 GAP43 GDI1 GPRIN1 IL17RD LRRC7 MAG MAP1B MAP1S MAP2 MAP2K1 MAP4 MARK2 MYCBP2 NEDD4L NF1 PSMD1 PSMD2 RAB3A RTN4 SIPA1L1 SLC9A6 SPTBN1 SRCIN1 SYNGAP1 TNIK TOP2B	ABLIM3 AGAP2 AP2A1 APC ARFGEF1 ARHGAP39 ASAP1 BCL11B CAMK2D GPRIN1 GRIN2A GRIN2B LRFN1 MAP1B MAP2 MARK3 NF1 NUMBL RIMS1 RTN4 SIPA1L1 SRC SYNGAP1 TIAM1 UBE4B
GO:0016192	vesicle-mediated transport	5.7358	10.0000	AAK1 ABR AHI1 AP2A1 AP4E1 ARHGAP21 ATP6V1H ATP8A1 C2CD5 CHGA COL3A1 CSNK1A1 CTNNB1 DBNL DNAJC5 GAPVD1 KCNB1 MAP2K1 NEDD4L PACS1 PCLO PDPK1 PIK3C3 PUM1 PYCARD RAB3A SEC22B SEC24D SGIP1 SNX12 SPTBN1 SRCIN1 SYN1 SYNJ1 YWHAZ	AAK1 AKT2 AMPH AP2A1 AP4E1 ARFGEF1 ARHGAP21 CHGA CPLX1 EHD3 GAPVD1 HTT KCNB1 KIF17 NAPA PACS1 PCLO PDPK1 PKDREJ PTPN23 RAB11FIP2 RIMS1 SCFD1 SCIN SEC22B SRC SYN1 SYNJ1 TBC1D5 TRAPPC8 YWHAZ
GO:0051336	regulation of hydrolase activity	10.0000	5.7298	ABR ADCY5 ADCY9 AGAP2 AMOT ARHGAP21 ARHGAP33 ARHGEF10 ARHGEF2 ASAP1 CAMK2D DBN1 DOCK5 GAPVD1 GARNL3 GDI1 LMTK3 MAP2K1 MKL1 MYO9B NF1 NPM1 PDE1B PDPK1 PSD3 PYCARD RAB3A RALGAPA1 RALGAPB SIPA1L1 SLC39A10 STARD8 SYNGAP1 TBC1D15	AGAP2 AKT2 AMOT AMPH ARFGEF1 ARHGAP21 ARHGEF2 ASAP1 CAMK2D GAPVD1 HNRNPU HTT IQSEC3 LMTK3 NF1 NPM1 PDE1B PDPK1 RPS3 SIPA1L1 SRC SYNGAP1 TBC1D15 TBC1D5 TIAM1 UBXN1
GO:0061564	axon development	10.0000	5.6612	ABLIM3 AGAP2 AP2A1 APC ARHGAP39 BRSK1 CAMK2D COL3A1 CRMP1 DBN1 DPYSL5 GAP43 GDI1 IL17RD MAG MAP1B MAP2 MAP2K1 MARK2 MYCBP2 NF1 PSMD1 PSMD2 RAB3A RTN4 SIPA1L1 SLC9A6 SPTBN1 SYNGAP1 TOP2B	ABLIM3 AGAP2 AP2A1 APC ARHGAP39 BCL11B CAMK2D GRIN2A GRIN2B LRFN1 MAP1B MAP2 MARK3 NF1 NUMBL RTN4 SIPA1L1 SRC SYNGAP1 TIAM1
GO:0033043	regulation of organelle organization	5.5866	10.0000	ABLIM3 AMOT APC ARHGEF10 ARHGEF2 ATXN2L C2CD5 CAMK2D CAMSAP2 CTNNB1 DBN1 DBNL HNRNPC MAP1A MAP1B MAP1S MAP2K1 MARK2 MYCBP2 NPM1 PYCARD RAB3A SEC22B SIRT2 SLC9A1 SPTBN1 SYNPO TAOK1 TBC1D15	ABLIM3 AKT2 AMOT APC ARFGEF1 ARHGEF2 ATXN2L CAMK2D EML2 ERCC4 FHOD1 HNRNPU HTT KANK3 MAP1A MAP1B NPM1 RPS3 SCFD1 SCIN SEC22B SRC SYNPO TBC1D15 TBC1D5 YWHAZ

Disease Models & Mechanisms • Supplementary information

				YWHAZ	
GO:0048812	neuron projection morphogenesis	10.0000	5.4657	ABLIM3 AGAP2 AP2A1 APC ARHGAP39 BRSK1 CAMK2D COL3A1 CRMP1 CTNND2 DBN1 DBNL DPYSL5 GAP43 GDI1 IL17RD MAG MAP1B MAP1S MAP2 MAP2K1 MARK2 MYCBP2 NEDD4L NF1 PSMD1 PSMD2 RAB3A RTN4 SIPA1L1 SLC9A6 SPTBN1 SRCIN1 SYNGAP1 TNIK TOP2B	ABLIM3 AGAP2 AP2A1 APC ARHGAP39 BCL11B CAMK2D GRIN2A GRIN2B LRFN1 MAP1B MAP2 MARK3 NF1 NUMBL RIMS1 RTN4 SIPA1L1 SRC SYNGAP1 TIAM1
GO:0043087	regulation of GTPase activity	10.0000	5.4589	ABR AGAP2 AMOT ARHGAP21 ARHGAP33 ARHGEF10 ARHGEF2 ASAP1 DOCK5 GAPVD1 GARNL3 GDI1 MAP2K1 MYO9B NF1 PSD3 PYCARD RALGAPA1 RALGAPB SIPA1L1 STARD8 SYNGAP1 TBC1D15	AGAP2 AKT2 AMOT AMPH ARFGEF1 ARHGAP21 ARHGEF2 ASAP1 GAPVD1 IQSEC3 NF1 SIPA1L1 SYNGAP1 TBC1D15 TBC1D5 TIAM1
GO:0043547	positive regulation of GTPase activity	10.0000	5.3319	ABR AGAP2 ARHGAP21 ARHGAP33 ARHGEF10 ARHGEF2 ASAP1 DOCK5 GAPVD1 GARNL3 GDI1 MAP2K1 MYO9B NF1 PSD3 RALGAPA1 RALGAPB SIPA1L1 STARD8 SYNGAP1 TBC1D15	AGAP2 AKT2 AMPH ARFGEF1 ARHGAP21 ARHGEF2 ASAP1 GAPVD1 IQSEC3 NF1 SIPA1L1 SYNGAP1 TBC1D15 TBC1D5 TIAM1
GO:0048667	cell morphogenesis involved in neuron differentiation	10.0000	5.1540	ABLIM3 AGAP2 AP2A1 APC ARHGAP39 BRSK1 CAMK2D COL3A1 CRMP1 CTNND2 DBN1 DPYSL5 GAP43 GDI1 IL17RD MAG MAP1B MAP2 MAP2K1 MARK2 MYCBP2 NF1 PSMD1 PSMD2 RAB3A RTN4 SIPA1L1 SLC9A6 SPTBN1 SRCIN1 SYNGAP1 TNIK TOP2B	ABLIM3 AGAP2 AP2A1 APC ARHGAP39 BCL11B CAMK2D GRIN2A GRIN2B LRFN1 MAP1B MAP2 MARK3 NF1 NUMBL RTN4 SIPA1L1 SRC SYNGAP1 TIAM1
GO:0048011	neurotrophin TRK receptor signaling pathway	10.0000	5.1040	ABR ADCY5 ADCY9 AP2A1 ARHGEF2 CAMK2D IL17RD MAG MAP2K1 NF1 PDE1B PDPK1 PSMD1 PSMD2 RTN4 SLC9A6 SPTBN1 SYNGAP1	AP2A1 ARHGEF2 CAMK2D GRIN2A GRIN2B MARK3 NF1 PDE1B PDPK1 RTN4 SRC SYNGAP1 TIAM1
GO:0007267	cell-cell signaling	5.1035	10.0000	ADCY5 ADCY9 AP2A1 APBA1 BRSK1 C2CD2L CACNA1B CACNA1E CAMK2D CSPG5 CTNNB1 CTNND2 DBN1 DNAJC5 HRH3 KCNB1 NF1 PCDH1 PCLO PDPK1 PPFIA4 RAB3A SIPA1L1 SLC6A11 SLC8A2 SYN1 SYN2 SYN3 SYNGAP1 SYNJ1 TJP1 YWHAZ	AMPH AP2A1 BCAN BSN CAMK2D COMT CPLX1 GNG3 GRIN2A GRIN2B HCN1 HTT KCNA2 KCNB1 NAPA NF1 PCLO PDPK1 PRKCB RAB11FIP2 RIMS1 SIPA1L1 SYN1 SYNGAP1 SYNJ1 TIAM1 TOLLIP YWHAZ
GO:0030029	actin filament-based process	10.0000	5.0963	ABLIM3 ABR AGAP2 AMOT ARHGEF10 ARHGEF2 ATP1A1 CAMK2D DBN1 DBNL FMNL1 MYO9B NEDD4L NF1 PDCD10 PDPK1 PYCARD SIPA1L1 SLC9A1 SPTBN1 STRIP1 SYNPO TNIK	ABLIM3 AGAP2 AMOT ARFGEF1 ARHGEF2 CAMK2D CDC42BPB FHOD1 FMNL1 IQSEC3 KANK3 NF1 PDPK1 SCIN SIPA1L1 SRC SYNPO
GO:0038179	neurotrophin signaling pathway	10.0000	5.0696	ABR ADCY5 ADCY9 AP2A1 ARHGEF2 CAMK2D IL17RD MAG MAP2K1 NF1 PDE1B PDPK1 PSMD1 PSMD2 RTN4 SLC9A6 SPTBN1 SYNGAP1	AP2A1 ARHGEF2 CAMK2D GRIN2A GRIN2B MARK3 NF1 PDE1B PDPK1 RTN4 SRC SYNGAP1 TIAM1
GO:0051345	positive regulation of hydrolase activity	10.0000	4.9161	ABR ADCY5 ADCY9 AGAP2 ARHGAP21 ARHGAP33 ARHGEF10 ARHGEF2 ASAP1 DOCK5 GAPVD1 GARNL3 GDI1 MAP2K1 MYO9B NF1 PDE1B PDPK1 PSD3 PYCARD RAB3A RALGAPA1 RALGAPB SIPA1L1 SLC39A10 STARD8 SYNGAP1 TBC1D15	AGAP2 AKT2 AMPH ARFGEF1 ARHGAP21 ARHGEF2 ASAP1 GAPVD1 HNRNPU IQSEC3 NF1 PDE1B PDPK1 RPS3 SIPA1L1 SYNGAP1 TBC1D15 TBC1D5 TIAM1
GO:0001505	regulation of neurotransmitter levels	10.0000	4.8415	APBA1 BRSK1 CACNA1B DNAJC5 HRH3 NF1 PCLO PDE1B PPFIA4 RAB3A SLC6A11 SYN1 SYN2 SYN3 SYNJ1	COMT CPLX1 NAPA NF1 PCLO PDE1B RIMS1 SYN1 SYNJ1

Disease Models & Mechanisms • Supplementary information

GO:0032940	secretion by cell	5.1006	5.6649	ABR ADCY5 AHI1 APBA1 BRSK1 C2CD2L CACNA1B CACNA1E CHGA DNAJC5 HRH3 KCNB1 NF1 PCLO PDPK1 PIK3C3 PPFIA4 PYCARD RAB3A RTN4 SLC6A11 SPTBN1 SRCIN1 SYN1 SYN2 SYN3 SYNJ1 YWHAZ	ARFGEF1 CHGA CPLX1 GRIN2B HTT KCNA2 KCNB1 NAPA NF1 PCLO PDPK1 PKDREJ PRKCB RAB11FIP2 RIMS1 RTN4 SCFD1 SCIN SRC SYN1 SYNJ1 TIAM1 YWHAZ
GO:0007163	establishment or maintenance of cell polarity	5.4304	5.1746	AMOT APC ARHGEF2 BRSK1 MAP1B MAP2 MAP4 MAP7 MARK2 MYO9B SLC9A1	AMOT APC ARFGEF1 ARHGEF2 CDC42BPB HTT MAP1B MAP2 RAB11FIP2
GO:1901653	cellular response to peptide	4.8994	5.2205	ADCY5 ADCY9 ARHGEF2 ATP6V1H C2CD5 CAMK2D CTNNB1 IL17RD MAP2K1 NF1 PDPK1 PIK3C3 PRKAB1 PSMD1 PSMD2 SLC9A1 SPTBN1 SYNGAP1	AKT2 ARHGEF2 CAMK2D GNG3 GRIN2A GRIN2B MARK3 NF1 NUCKS1 PDPK1 PRKAB2 PRKAG2 PRKCB SRC SYNGAP1
GO:0022603	regulation of anatomical structure morphogenesis	10.0000		ABR AHI1 AMOT APC CAMSAP1 CTNNB1 DBN1 EPB42 FMNL1 GDI1 HSPB1 ITGA7 MAG MAP1B MAP2 MAP2K1 MARK2 MTDH NEDD4L NF1 PDCD10 RTN4 SIPA1L1 SRCIN1 STRIP1 SULF2 SYNGAP1 TBC1D15 TNIK	
GO:0051056	regulation of small GTPase mediated signal transduction	10.0000		ABR AMOT ARHGAP33 ARHGAP39 ARHGEF10 ARHGEF2 CAMK2D COL3A1 GARNL3 GDI1 MAP2K1 MYO9B NF1 PDCD10 PSD3 RALGAPA1 RALGAPB SIPA1L1 STARD8 SYNGAP1	
GO:0051494	negative regulation of cytoskeleton organization		10.0000		APC ARFGEF1 ARHGEF2 EML2 KANK3 MAP1A MAP1B NPM1 SCIN
GO:0006887	exocytosis		10.0000		ARFGEF1 CHGA CPLX1 KCNB1 NAPA PCLO PDPK1 PKDREJ RAB11FIP2 RIMS1 SCFD1 SCIN SYN1 SYNJ1 YWHAZ
GO:0010256	endomembrane system organization	10.0000		ARHGAP21 C2CD5 COL4A3BP CSNK1A1 GPR158 KCNB1 MAP2K1 MAP7 NDRG1 PACS1 PDCD10 PDPK1 PIK3C3 RTN4 SGIP1 SPTBN1 SYNJ1 TNIK VCPIP1 YWHAZ	
GO:0007269	neurotransmitter secretion	10.0000		APBA1 BRSK1 CACNA1B DNAJC5 HRH3 NF1 PCLO PPFIA4 RAB3A SLC6A11 SYN1 SYN2 SYN3 SYNJ1	
GO:0044087	regulation of cellular component biogenesis		10.0000		AMOT APC ARFGEF1 ATXN2L CHGA EML2 FHOD1 HTT KANK3 MAP1B NAPA NPM1 RIMS1 RPS3 SCFD1 SCIN SRC SYNPO TBC1D15 TBC1D5
GO:0000904	cell morphogenesis involved in differentiation	10.0000		ABLIM3 AGAP2 AP2A1 APC ARHGAP39 BRSK1 CAMK2D COL3A1 CRMP1 CTNNB1 CTNND2 DBN1 DPYSL5 GAP43 GDI1 IL17RD MAG MAP1B MAP2 MAP2K1 MARK2 MYCBP2 NF1 PSMD1 PSMD2 RAB3A RTN4 SIPA1L1 SLC9A6 SPTBN1 SRCIN1 SYNGAP1 TNIK TOP2B	
GO:0060627	regulation of vesicle-mediated transport		10.0000		AAK1 AKT2 ARHGAP21 CPLX1 KCNB1 NAPA PCLO PDPK1 PKDREJ PTPN23 RIMS1 SCFD1 SRC SYN1 SYNJ1 TBC1D5

Disease Models & Mechanisms • Supplementary information

GO:0006836	neurotransmitter transport	10.0000		APBA1 BRSK1 CACNA1B DNAJC5 HRH3 NF1 PCLO PPFIA4 RAB3A SLC6A11 SYN1 SYN2 SYN3 SYNJ1	
GO:0007265	Ras protein signal transduction	5.9502		ABR ARHGEF10 ARHGEF2 CAMK2D COL3A1 DBNL G3BP2 GDI1 IL17RD MAP2K1 MYO9B NF1 PDCD10 PSD3 PSMD1 PSMD2 RALGAPB SPTBN1 SYNGAP1	
GO:0007169	transmembrane receptor protein tyrosine kinase signaling pathway	5.9234		ABR ADCY5 ADCY9 AHI1 AP2A1 ARHGEF2 ATP6V1H C2CD5 CAMK2D CTNNB1 HSPB1 IL17RD MAG MAP2K1 NF1 PDE1B PDPK1 PIK3C3 PRKAB1 PSMD1 PSMD2 RTN4 SIPA1L1 SIRT2 SLC9A6 SPTBN1 SULF2 SYNGAP1	
GO:0010639	negative regulation of organelle organization		5.9090		APC ARFGEF1 ARHGEF2 EML2 ERCC4 HNRNPU KANK3 MAP1A MAP1B NPM1 SCFD1 SCIN SRC
GO:0051493	regulation of cytoskeleton organization	5.8864		AMOT APC ARHGEF10 ARHGEF2 CTNNB1 DBN1 DBNL MAP1A MAP1B MARK2 MYCBP2 NPM1 PYCARD SIRT2 SLC9A1 SPTBN1 SYNPO TAOK1	
GO:0008286	insulin receptor signaling pathway		5.8526		AKT2 CAMK2D GRIN2A GRIN2B MARK3 NF1 NUCKS1 PDPK1 PRKAB2 PRKAG2 PRKCB SRC SYNGAP1
GO:0023061	signal release	5.7546		ADCY5 APBA1 BRSK1 C2CD2L CACNA1B CACNA1E DNAJC5 HRH3 KCNB1 NF1 PCLO PPFIA4 RAB3A SLC6A11 SYN1 SYN2 SYN3 SYNJ1 YWHAZ	
GO:0055067	monovalent inorganic cation homeostasis	5.6749		ANPEP ATP1A1 ATP1A3 ATP4A ATP6V1H CAMK2D NEDD4L SLC4A4 SLC9A1 SLC9A6	
GO:0006883	cellular sodium ion homeostasis	5.5297		ATP1A1 ATP1A3 ATP4A NEDD4L SLC9A1	
GO:0016079	synaptic vesicle exocytosis		5.5016		CPLX1 NAPA PCLO RIMS1 SYN1 SYNJ1
GO:0022604	regulation of cell morphogenesis	5.4930		CAMSAP1 CTNNB1 DBN1 EPB42 FMNL1 GDI1 ITGA7 MAG MAP1B MAP2 MAP2K1 MARK2 NEDD4L RTN4 SIPA1L1 SRCIN1 STRIP1 SYNGAP1 TBC1D15 TNIK	
GO:0008306	associative learning		5.4854		GRIN2A GRIN2B HTT NF1 PDE1B PPP1R1B SYNGAP1
GO:0048167	regulation of synaptic plasticity		5.4831		BCAN GRIN2A GRIN2B HTT KCNB1 NF1 RIMS1 SIPA1L1 SYNGAP1
GO:0008542	visual learning		5.4110		GRIN2A HTT NF1 PDE1B PPP1R1B SYNGAP1
GO:0032880	regulation of protein localization	5.4068		AAK1 ABLIM3 ADCY5 AHI1 APC C2CD2L C2CD5 CACNA1E CTNNB1 DBN1 G3BP2 GAPVD1 GDI1 KCNB1 MYCBP2 NEDD4L NF1 PDPK1 PIK3C3 PYCARD RALGAPB	

				SIRT2 SLC9A1 SNX12 SPTBN1 SRCIN1 SULF2 YWHAZ	
GO:0048169	regulation of long-term neuronal synaptic plasticity		5.3573		GRIN2A GRIN2B NF1 RIMS1 SYNGAP1
GO:1901701	cellular response to oxygen-containing compound		5.3434		AKT2 ARHGEF2 CAMK2D GNG3 GRIN2A GRIN2B HCN1 HNRNPU KCNB1 MARK3 NF1 NUCKS1 PDE2A PDPK1 PRKAB2 PRKAG2 PRKCB RAB11FIP2 RPS3 SRC SYNGAP1 TIAM1 YAP1
GO:0090168	Golgi reassembly	5.3265		PDCD10 VCPIP1 YWHAZ	
GO:0046903	secretion		5.3185		ARFGEF1 CHGA COMT CPLX1 GRIN2B HTT KCNA2 KCNB1 NAPA NF1 PCLO PDPK1 PKDREJ PRKCB RAB11FIP2 RIMS1 RTN4 SCFD1 SCIN SRC SYN1 SYNJ1 TIAM1 YWHAZ
GO:0050804	modulation of synaptic transmission		5.2944		BCAN GRIN2A GRIN2B HTT KCNB1 NAPA NF1 RIMS1 SIPA1L1 SYN1 SYNGAP1 SYNJ1
GO:1901699	cellular response to nitrogen compound		5.2873		AKT2 ARHGEF2 CAMK2D GNG3 GRIN2A GRIN2B HCN1 MARK3 NF1 NPM1 NUCKS1 PDE2A PDPK1 PRKAB2 PRKAG2 PRKCB SRC SYNGAP1
GO:0030036	actin cytoskeleton organization		5.2766		ABLIM3 AGAP2 AMOT ARFGEF1 ARHGEF2 CDC42BPB FHOD1 FMNL1 IQSEC3 KANK3 NF1 PDPK1 SCIN SIPA1L1 SRC SYNPO
GO:0048168	regulation of neuronal synaptic plasticity		5.2398		BCAN GRIN2A GRIN2B NF1 RIMS1 SYNGAP1
GO:1902001	fatty acid transmembrane transport		5.2314		ABCC1 AKT2 PRKAB2 PRKAG2
GO:0007632	visual behavior		5.1989		GRIN2A HTT NF1 PDE1B PPP1R1B SYNGAP1
GO:0071417	cellular response to organonitrogen compound		5.1386		AKT2 ARHGEF2 CAMK2D GNG3 GRIN2A GRIN2B HCN1 MARK3 NF1 NUCKS1 PDE2A PDPK1 PRKAB2 PRKAG2 PRKCB SRC SYNGAP1

Disease Models & Mechanisms • Supplementary information

GO:0008543	fibroblast growth factor receptor signaling pathway	5.1121		ADCY5 ADCY9 CAMK2D CTNNB1 IL17RD MAP2K1 NF1 PDE1B PDPK1 PSMD1 PSMD2 SPTBN1 SULF2 SYNGAP1	
GO:0031111	negative regulation of microtubule polymerization or depolymerization		5.1112		APC ARHGEF2 EML2 MAP1A MAP1B
GO:0051650	establishment of vesicle localization		5.1039		AMPH CPLX1 HTT NAPA PCLO RIMS1 SCFD1 SEC22B SYN1 SYNJ1
GO:0050688	regulation of defense response to virus	5.0975		ALKBH5 AP2A1 ATP6V1H CSPG5 KIAA1549L MAP1A PACS1 PNPO PUM1 PYCARD TNIK	
GO:0030007	cellular potassium ion homeostasis	5.0830		ATP1A1 ATP1A3 ATP4A CAMK2D	
GO:0035725	sodium ion transmembrane transport	5.0562		ATP1A1 ATP1A3 ATP4A CAMK2D NEDD4L SLC4A4 SLC8A2 SLC9A1 SLC9A6	
GO:0032869	cellular response to insulin stimulus		5.0242		AKT2 CAMK2D GRIN2A GRIN2B MARK3 NF1 NUCKS1 PDPK1 PRKAB2 PRKAG2 PRKCB SRC SYNGAP1
GO:0031110	regulation of microtubule polymerization or depolymerization		4.9678		APC ARHGEF2 EML2 MAP1A MAP1B RPS3
GO:0071436	sodium ion export	4.9405		ATP1A1 ATP1A3 ATP4A SLC9A1	
GO:0048313	Golgi inheritance	4.9405		MAP2K1 PDCD10 VCPIP1 YWHAZ	
GO:0051046	regulation of secretion		4.9003		CHGA COMT CPLX1 GRIN2B KCNA2 KCNB1 NAPA NF1 PCLO PDPK1 PKDREJ PRKCB RIMS1 RTN4 SCIN SRC SYN1 TIAM1

Disease Models & Mechanisms • Supplementary information

GO:0051648	vesicle localization		4.8981		AMPH CPLX1 HTT NAPA PCLO RIMS1 SCFD1 SEC22B SYN1 SYNJ1
GO:0061024	membrane organization		4.8699		AKT2 AP4E1 EHD3 GRIN2B HTT KCNB1 NAPA NUP210 PACS1 PDPK1 PRKAB2 PRKAG2 PRKCB RIMS1 RPS3 SCFD1 SEC22B SRC SYNGAP1 SYNJ1 TIAM1 YWHAZ
GO:0048489	synaptic vesicle transport		4.8353		AMPH CPLX1 NAPA PCLO RIMS1 SYN1 SYNJ1
GO:0097480	establishment of synaptic vesicle localization		4.8353		AMPH CPLX1 NAPA PCLO RIMS1 SYN1 SYNJ1

Disease Models & Mechanisms • Supplementary information

Supplementary Table S13: Biological processes alterd by the common proteins up-and down-phosphorylated in the cerebellum of Tg(*Dyrk1a*) and Ts65Dn mice, and reverted by L41 treatment. ToppCluster analysis.

ID	GO: Biological Process	TgDyrk1a logP	Ts65Dn logP	TgDyrk1a	Ts65Dn
		pValues		Gene List	
GO:0048858	cell projection morphogenesis	10.0000	10.0000	ABLIM2 ARHGAP35 ARHGEF12 ASAP1 BRSK1 DCLK1 DOCK7 DPYSL3 FEZ1 GSK3B HTT KIF13B MAP1B MAP2 MAP4K4 MAPT PACSIN1 PARK2 PLXNB3 PSMD1 RIMS1 RIMS2 RUFY3 SPRED1 SPTBN1 SPTBN2 SPTBN4 SRCIN1 STMN1 TBC1D22B TNIK TOP2B USP9X	ABLIM2 ANK1 ARHGEF12 CAMK2B COL3A1 CSNK1D CTTN DCLK1 DNM1 DPYSL2 DPYSL5 EGFR EP300 GIT1 GOLGA4 HTT IMPACT KCNQ2 KIF13B MAP1B MAP2 MINK1 NEFH PAK1 PLEKHO1 PLXNB3 PSMD4 PTPRZ1 PVRL1 RAB3A RAPGEF2 ROBO2 RTN4 SHANK1 SHANK3 SPTBN2 SPTBN4 SRGAP2 TIAM1 TOP2B
GO:0051640	organelle localization	10.0000	10.0000	AP3D1 ARFGAP1 ARHGAP21 CHMP6 CLUH CTNNB1 DOCK7 FEZ1 GBF1 HTT MAP1B MAPT MYO5A NLGN2 PACSIN1 PARK2 PCLO PDPK1 RIMS1 RIMS2 SPIRE1 SPTBN2 SYN1	AMPH AP3B2 ARHGAP21 ARHGEF2 CSNK1D DNM1 DPYSL2 DYNC1I1 FMN2 HTT MAGI2 MAP1B MAP4 MYO5A PCLO PDPK1 RAB3A RAP1B SEC16A SPTBN2 STX1B SYN1
GO:0048666	neuron development	10.0000	10.0000	ABLIM2 ANKS1A ARHGAP35 ARHGEF12 ASAP1 BRSK1 CAMSAP2 DCLK1 DOCK7 DPYSL3 FEZ1 GBA2 GSK3B HTT KIF13B MAP1B MAP2 MAP4K4 MAPT MTOR NDRG4 NLGN2 PACSIN1 PARK2 PLXNB3 PSMD1 RIMS1 RIMS2 RUFY3 SPRED1 SPTBN1 SPTBN2 SPTBN4 SRCIN1 STMN1 TNIK TOP2B UBE4B USP9X	ABLIM2 ANK1 ARHGEF12 CAMK2B CAMSAP2 COL3A1 CTNNA1 CTTN DCLK1 DNM1 DPYSL2 DPYSL5 EGFR EP300 GIT1 GOLGA4 GPRIN1 GSK3A HTT IMPACT KCNQ2 KIF13B MAGI2 MAP1B MAP2 MAP4 MINK1 NEFH PAK1 PLXNB3 PREX1 PSMD4 PTPRZ1 PVRL1 RAB3A RAPGEF2 ROBO2 RTN4 SHANK1 SHANK3 SOD1 SPTBN2 SPTBN4 SRGAP2 STRN TIAM1 TOP2B UBE4B
GO:0031175	neuron projection development	10.0000	10.0000	ABLIM2 ARHGAP35 ARHGEF12 ASAP1 BRSK1 CAMSAP2 DCLK1 DOCK7 DPYSL3 FEZ1 GSK3B KIF13B MAP1B MAP2 MAP4K4 MAPT MTOR NDRG4 NLGN2 PACSIN1 PARK2 PLXNB3 PSMD1 RIMS1 RIMS2 RUFY3 SPRED1 SPTBN1 SPTBN2 SPTBN4 SRCIN1 STMN1 TNIK TOP2B UBE4B USP9X	ABLIM2 ANK1 ARHGEF12 CAMK2B CAMSAP2 COL3A1 CTNNA1 CTTN DCLK1 DNM1 DPYSL2 DPYSL5 EGFR EP300 GIT1 GOLGA4 GPRIN1 GSK3A IMPACT KCNQ2 KIF13B MAGI2 MAP1B MAP2 MAP4 MINK1 NEFH PAK1 PLXNB3 PREX1 PSMD4 PTPRZ1 PVRL1 RAB3A RAPGEF2 ROBO2 RTN4 SHANK1 SHANK3 SPTBN2 SPTBN4 SRGAP2 STRN TIAM1 TOP2B UBE4B
GO:0033043	regulation of organelle organization	10.0000	10.0000	ADD1 BAD CAMSAP2 CEP76 CORO1A CTNNB1 FEZ1 GSK3B HNRNPC HTT KMT2B LCP1 MAP1A MAP1B MAPT MTOR MTSS1 MYC MYO5A PARK2 PRKCE SIRT2 SPIRE1 SPTBN1 SPTBN2 SPTBN4 STMN1 TBC1D22B TOM1L2 UBQLN2 WBP11	ADD1 ADD2 AKT2 ANK1 ARFIP2 ARHGEF2 BCAS3 CAMK2B CAMSAP2 CDC26 CEP76 CORO1A CTTN DMTN EP300 FMN2 GSK3A HNRNPU HTT KMT2B MAP1A MAP1B MYC MYO5A PAK1 PHF8 PKIB PML PREX1 RAB3A RICTOR RPS3 SHANK1 SHANK3 SIRT2 SLC9A1 SPTBN2 SPTBN4 TOM1L2 TPR WIPF3 YWHAE
GO:0000904	cell morphogenesis involved in differentiation	10.0000	10.0000	ABLIM2 ARHGAP35 ARHGEF12 BRSK1 CTNNB1 DCLK1 DOCK7 DPYSL3 FEZ1 FRMD6 GSK3B KIF13B MAP1B MAP2 MAPT PLXNB3 PSMD1 PXN RUFY3 SPRED1 SPTBN1 SPTBN2 SPTBN4 SRCIN1 STMN1 TMEM100 TNIK TOP2B TRIM28 TTC3 USP9X	ABLIM2 ANK1 ARHGEF12 CAMK2B COL18A1 COL3A1 CTTN DCLK1 DMTN DNM1 DPYSL2 DPYSL5 EGFR EP300 FRMD6 GIT1 GOLGA4 KCNQ2 KIF13B MAP1B MAP2 MINK1 NEFH PAK1 PLXNB3 PREX1 PSMD4 PTPRZ1 PVRL1 RAB3A RAPGEF2 ROBO2 RTN4 SHANK1 SHANK3 SOD1 SPTBN2 SPTBN4 SRGAP2 TGFB1I1 TIAM1 TOP2B

Disease Models & Mechanisms • Supplementary information

GO:0048812	neuron projection morphogenesis	10.0000	10.0000	ABLIM2 ARHGAP35 ARHGEF12 BRSK1 DCLK1 DOCK7 DPYSL3 FEZ1 GSK3B KIF13B MAP1B MAP2 MAP4K4 MAPT PACSIN1 PARK2 PLXNB3 PSMD1 RIMS1 RIMS2 RUFY3 SPRED1 SPTBN1 SPTBN2 SPTBN4 SRCIN1 STMN1 TNIK TOP2B USP9X	ABLIM2 ANK1 ARHGEF12 CAMK2B COL3A1 CTTN DCLK1 DNM1 DPYSL2 DPYSL5 EGFR EP300 GIT1 GOLGA4 IMPACT KCNQ2 KIF13B MAP1B MAP2 MINK1 NEFH PAK1 PLXNB3 PSMD4 PTPRZ1 PVRL1 RAB3A RAPGEF2 ROBO2 RTN4 SHANK1 SHANK3 SPTBN2 SPTBN4 SRGAP2 TIAM1 TOP2B
GO:0051648	vesicle localization	10.0000	10.0000	AP3D1 ARFGAP1 CTNNB1 GBF1 HTT MYO5A NLGN2 PACSIN1 PARK2 PCLO RIMS1 RIMS2 SPTBN2 SYN1	AMPH AP3B2 CSNK1D DNM1 DPYSL2 DYNC1I1 HTT MAGI2 MYO5A PCLO RAB3A RAP1B SEC16A SPTBN2 STX1B SYN1
GO:0030029	actin filament-based process	10.0000	10.0000	ABLIM2 ACTA1 ADD1 ARHGEF18 ATP1A1 CORO1A DPYSL3 DSG2 FRMD6 LCP1 LIMCH1 MTOR MTSS1 MYO5A NOS1AP PACSIN1 PARK2 PDPK1 PRKCE RUFY3 RYR2 SPIRE1 SPTBN1 SPTBN2 SPTBN4 TNIK	ABLIM2 ADD1 ADD2 ARFIP2 ARHGEF2 BCAS3 CORO1A CTNNA1 CTTN DMTN DSG2 ELMO1 EP300 FMN2 FRMD6 MINK1 MYO5A PAK1 PDPK1 PREX1 RICTOR RYR2 SHANK1 SHANK3 SLC9A1 SPTBN2 SPTBN4 SRGAP2 STRIP1 WIPF3
GO:0007010	cytoskeleton organization	10.0000	10.0000	ABLIM2 ACTA1 ADD1 ARHGEF18 BRSK1 CAMSAP2 CEP76 CORO1A CSNK1A1 CTNNB1 DOCK7 DPYSL3 GSK3B HTT KIF2A LCP1 LIMCH1 MAP1A MAP1B MAP2 MAP7 MAP7D1 MAP7D2 MAPT MAST1 MTOR MTSS1 NLGN2 PACSIN1 PARK2 PCLO PDPK1 PRKCE PXN RUFY3 SIRT2 SPIRE1 SPTBN1 SPTBN2 SPTBN4 STMN1 TNIK TPPP	ABLIM2 ADD1 ADD2 ANK1 ARFIP2 ARHGEF2 BCAS3 BIRC7 CAMSAP2 CEP76 CORO1A CSNK1A1 CSNK1D CTNNA1 CTTN DMTN DPYSL2 ELMO1 EML1 EP300 FMN2 HTT KIF2A MAP1A MAP1B MAP2 MAP4 MINK1 NEFH PAK1 PCLO PDPK1 PREX1 RANBP10 RICTOR RPS3 SHANK1 SHANK3 SIRT2 SLC9A1 SOD1 SPTBN2 SPTBN4 SRGAP2 STRIP1 TLN2 TPR WIPF3
GO:0007017	microtubule-based process	10.0000	10.0000	AP3D1 ARHGAP21 BRSK1 CAMSAP2 CEP76 CTNNB1 DOCK7 GSK3B HTT KIF13B KIF2A MAP1A MAP1B MAP2 MAP7 MAP7D1 MAP7D2 MAPT SIRT2 SPIRE1 STMN1 TPPP	AP3B2 APBA1 ARHGAP21 ARHGEF2 BCAS3 BIRC7 CAMSAP2 CEP76 CSNK1D DYNC1I1 EML1 FMN2 HTT KIF13B KIF17 KIF2A MAP1A MAP1B MAP2 MAP4 NEFH RANBP10 RPS3 SIRT2 SOD1 SRGAP2 TPR
GO:0032271	regulation of protein polymerization	10.0000	10.0000	ADD1 CORO1A MAP1B MAPT MTOR PRKCE SPIRE1 SPTBN1 SPTBN2 SPTBN4 STMN1 TPPP	ADD1 ADD2 ARFIP2 CORO1A CTTN DMTN FMN2 MAP1B PREX1 RICTOR RPS3 SPTBN2 SPTBN4 WIPF3
GO:0032990	cell part morphogenesis	10.0000	10.0000	ABLIM2 ARHGAP35 ARHGEF12 ASAP1 BRSK1 DCLK1 DOCK7 DPYSL3 FEZ1 GSK3B HTT KIF13B MAP1B MAP2 MAP4K4 MAPT PACSIN1 PARK2 PLXNB3 PSMD1 RIMS1 RIMS2 RUFY3 SPRED1 SPTBN1 SPTBN2 SPTBN4 SRCIN1 STMN1 TBC1D22B TNIK TOP2B USP9X	ABLIM2 ANK1 ARHGEF12 CAMK2B COL3A1 CSNK1D CTTN DCLK1 DNM1 DPYSL2 DPYSL5 EGFR EP300 GIT1 GOLGA4 HTT IMPACT KCNQ2 KIF13B MAP1B MAP2 MINK1 NEFH PAK1 PLEKHO1 PLXNB3 PSMD4 PTPRZ1 PVRL1 RAB3A RAPGEF2 ROBO2 RTN4 SHANK1 SHANK3 SPTBN2 SPTBN4 SRGAP2 TIAM1 TOP2B
GO:0051656	establishment of organelle localization	10.0000	10.0000	AP3D1 ARFGAP1 ARHGAP21 CHMP6 CTNNB1 FEZ1 GBF1 HTT MAP1B MAPT MYO5A NLGN2 PACSIN1 PARK2 PCLO RIMS1 RIMS2 SPIRE1 SPTBN2 SYN1	AMPH AP3B2 ARHGAP21 ARHGEF2 CSNK1D DNM1 DPYSL2 DYNC1I1 FMN2 HTT MAP1B MAP4 MYO5A PCLO RAB3A RAP1B SEC16A SPTBN2 STX1B SYN1
GO:0044087	regulation of cellular component biogenesis	10.0000	10.0000	ADD1 CEP76 CORO1A CTNNB1 DPYSL3 FEZ1 GSK3B HTT LCP1 MAP1B MAP3K7 MAPT MTOR MTSS1 NLGN2 PARK2 PLXNB3 PRKCE RIMS1 SACS SIRT2 SPIRE1 SPTBN1 SPTBN2 SPTBN4 STMN1 TAF1 TBC1D22B TPPP UBQLN2	ACE ADD1 ADD2 ARFIP2 BCAS3 CEP76 CORO1A CTTN DMTN EP300 FMN2 HTT IMPACT MAGI2 MAP1B MAP3K7 PAK1 PLXNB3 PREX1 PVRL1 RAP1B RAPGEF2 RICTOR ROBO2 RPS3 SHANK1 SHANK3 SIRT2 SLC9A1 SPTBN2 SPTBN4 STX1B TPR WIPF3
GO:0051129	negative regulation of cellular component organization	10.0000	10.0000	ADD1 ASAP1 CORO1A CTNNB1 DPYSL3 FEZ1 GSK3B HNRNPC MAP1A MAP1B MAP3K7 MAP4K4 MTOR PACSIN1 PARK2 PLXNB3 RUFY3 SACS SIRT2 SPTBN1 SPTBN2 SPTBN4 STMN1 TOM1L2 TTC3 UBQLN2	ACE ADD1 ADD2 ARHGEF2 BCAS3 CDC26 CORO1A DMTN GSK3A HNRNPU IMPACT MAP1A MAP1B MAP3K7 PHF8 PLXNB3 PML PTPRZ1 RAPGEF2 ROBO2 RTN4 SHANK1 SHANK3 SIRT2 SPTBN2 SPTBN4 TMBIM1 TOM1L2 TPR

Disease Models & Mechanisms • Supplementary information

GO:0097479	synaptic vesicle localization	10.0000	10.0000	AP3D1 CTNNB1 NLGN2 PACSIN1 PARK2 PCLO RIMS1 RIMS2 SPTBN2 SYN1	AMPH AP3B2 DNM1 DPYSL2 MAGI2 PCLO RAB3A RAP1B SPTBN2 STX1B SYN1
GO:0051650	establishment of vesicle localization	10.0000	5.9775	AP3D1 ARFGAP1 CTNNB1 GBF1 HTT MYO5A NLGN2 PACSIN1 PARK2 PCLO RIMS1 RIMS2 SPTBN2 SYN1	AMPH AP3B2 CSNK1D DNM1 DPYSL2 DYNC1I1 HTT MYO5A PCLO RAB3A RAP1B SEC16A SPTBN2 STX1B SYN1
GO:0043254	regulation of protein complex assembly	10.0000	5.8788	ADD1 CORO1A CTNNB1 GSK3B LCP1 MAP1B MAP3K7 MAPT MTOR PRKCE RIMS1 SIRT2 SPIRE1 SPTBN1 SPTBN2 SPTBN4 STMN1 TAF1 TPPP	ADD1 ADD2 ARFIP2 CORO1A CTTN DMTN FMN2 IMPACT MAP1B MAP3K7 PREX1 RICTOR RPS3 SIRT2 SPTBN2 SPTBN4 STX1B TPR WIPF3
GO:0051258	protein polymerization	10.0000	5.8391	ADD1 CORO1A MAP1B MAPT MTOR MTSS1 PARK2 PRKCE SPIRE1 SPTBN1 SPTBN2 SPTBN4 STMN1 TPPP	ADD1 ADD2 ARFIP2 CORO1A CSNK1D CTTN DMTN FMN2 MAP1B PREX1 RICTOR RPS3 SPTBN2 SPTBN4 WIPF3
GO:0010256	endomembrane system organization	5.5650	10.0000	AP3D1 ARHGAP21 CSNK1A1 GBF1 HTT KCNIP4 KIAA1715 MAP7 MTSS1 MTSS1L MYO5A NDRG1 PACSIN1 PDPK1 SPTBN1 SPTBN4 TNIK TSC2 USP8	AKT2 ANK1 ARHGAP21 BCAS3 CSNK1A1 CSNK1D DNM1 GOLGA4 GPR158 HTT KCNIP4 MYO5A NUP85 PACS2 PDPK1 RANBP2 RAPGEF2 RTN3 RTN4 SEC16A SOD1 SPTBN4 TMBIM1 TPR TSC2
GO:0050767	regulation of neurogenesis	10.0000	5.5304	ARHGAP35 ASAP1 CTNNB1 DOCK7 DPYSL3 FEZ1 GSK3B KIF13B MAP1B MAP2 MAP4K4 MAPT MTOR MYC NDRG4 NLGN2 PACSIN1 PARK2 PLXNB3 RIMS1 RIMS2 RUFY3 SIRT2 SRCIN1 TNIK TTC3	ACE ARHGEF2 CAMK2B COL3A1 CTNNA1 CTTN CX3CR1 DPYSL2 EP300 GOLGA4 GSK3A IMPACT KIF13B MAGI2 MAP1B MAP2 MYC PLXNB3 PREX1 PTPRZ1 RAPGEF2 ROBO2 RTN4 SHANK1 SHANK3 SIRT2 SRGAP2 TIAM1
GO:0048489	synaptic vesicle transport	10.0000	5.5182	AP3D1 CTNNB1 NLGN2 PACSIN1 PARK2 PCLO RIMS1 RIMS2 SPTBN2 SYN1	AMPH AP3B2 DNM1 DPYSL2 PCLO RAB3A RAP1B SPTBN2 STX1B SYN1
GO:0097480	establishment of synaptic vesicle localization	10.0000	5.5182	AP3D1 CTNNB1 NLGN2 PACSIN1 PARK2 PCLO RIMS1 RIMS2 SPTBN2 SYN1	AMPH AP3B2 DNM1 DPYSL2 PCLO RAB3A RAP1B SPTBN2 STX1B SYN1
GO:0030036	actin cytoskeleton organization	5.4825	10.0000	ABLIM2 ACTA1 ADD1 ARHGEF18 CORO1A DPYSL3 LCP1 LIMCH1 MTOR MTSS1 PACSIN1 PARK2 PDPK1 PRKCE RUFY3 SPIRE1 SPTBN1 SPTBN2 SPTBN4 TNIK	ABLIM2 ADD1 ADD2 ARFIP2 ARHGEF2 BCAS3 CORO1A CTNNA1 CTTN DMTN ELMO1 EP300 FMN2 MINK1 PAK1 PDPK1 PREX1 RICTOR SHANK1 SHANK3 SLC9A1 SPTBN2 SPTBN4 STRIP1 WIPF3
GO:0070848	response to growth factor	10.0000	5.4783	ARHGEF12 ARHGEF18 BAD BYSL CORO1A CTNNB1 FEZ1 GSK3B HHIP HSPA5 MAP3K7 MCF2L MTOR MYC PDPK1 PRKAR1B PRKCE PSMD1 PXN RYR2 SIRT2 SPRED1 SPTBN1 SPTBN2 SPTBN4 TMEM100 TSC2 USP9X	ARHGEF12 ARHGEF2 CAMK2B CASK COL3A1 CORO1A CX3CR1 EGFR EP300 GSK3A HSPA5 KCNC1 MAGI2 MAP3K7 MYC PDPK1 PML PRKAR2A PSMD4 RAPGEF2 RICTOR RPS3 RTN4 RYR2 SIRT2 SPTBN2 SPTBN4 TGFB1I1 TIAM1 TPR TSC2 YWHAE
GO:0051960	regulation of nervous system development	10.0000	5.4561	ARHGAP35 ASAP1 CTNNB1 DOCK7 DPYSL3 FEZ1 GSK3B KIF13B MAP1B MAP2 MAP4K4 MAPT MTOR MYC NDRG4 NLGN2 PACSIN1 PARK2 PLXNB3 RIMS1 RIMS2 RUFY3 SIRT2 SRCIN1 TNIK TTC3	ACE ARHGEF2 CAMK2B CEND1 COL3A1 CTNNA1 CTTN CX3CR1 DPYSL2 EP300 GOLGA4 GSK3A IMPACT KIF13B MAGI2 MAP1B MAP2 MYC PLXNB3 PREX1 PTPRZ1 PVRL1 RAPGEF2 ROBO2 RTN4 SHANK1 SHANK3 SIRT2 SRGAP2 TIAM1
GO:0032970	regulation of actin filament-based process	5.3892	10.0000	ADD1 ATP1A1 CORO1A DSG2 FRMD6 MTOR MTSS1 NOS1AP PARK2 PRKCE RYR2 SPIRE1 SPTBN1 SPTBN2 SPTBN4	ADD1 ADD2 ARFIP2 BCAS3 CORO1A CTTN DMTN DSG2 EP300 FMN2 FRMD6 PAK1 PREX1 RICTOR RYR2 SHANK1 SHANK3 SLC9A1 SPTBN2 SPTBN4 WIPF3
GO:0051493	regulation of cytoskeleton organization	5.3753	10.0000	ADD1 CEP76 CORO1A CTNNB1 MAP1A MAP1B MAPT MTOR MTSS1 PARK2 PRKCE SIRT2 SPIRE1 SPTBN1 SPTBN2 SPTBN4 STMN1	ADD1 ADD2 ARFIP2 ARHGEF2 BCAS3 CEP76 CORO1A CTTN DMTN EP300 FMN2 MAP1A MAP1B PAK1 PREX1 RICTOR RPS3 SHANK1 SHANK3 SIRT2 SLC9A1 SPTBN2 SPTBN4 TPR WIPF3

GO:1902580	single-organism cellular localization	5.3144	10.0000	AP3D1 ARFGAP1 ARHGAP21 BAD CHMP6 CLUH CTNNB1 GBF1 GSK3B HTT KCNIP4 MAP1B MAP7 MAPT MTOR MYO5A NLGN2 PACSIN1 PARK2 PCLO PDPK1 RIMS1 RIMS2 SPIRE1 SPTBN1 SPTBN2 SPTBN4 SYN1 TNIK TRIM28 TSC2 WBP11	AKAP1 AKT2 AMPH ANK1 AP3B2 ARHGAP21 ARHGEF2 CSNK1D DMTN DNM1 DPYSL2 DYNC1I1 EGFR EP300 FMN2 GOLGA4 GPR158 GSK3A HTT KCNIP4 LIME1 MAGI2 MAP1B MAP4 MYO5A NUP85 PACS2 PAK1 PCLO PDPK1 PEX19 PML RAB3A RANBP2 RAP1B RAPGEF2 RPS28 RPS3 SEC16A SHANK3 SLC9A1 SPTBN2 SPTBN4 STX1B SYN1 TIAM1 TMBIM1 TPR TSC2 YWHAE
GO:0048667	cell morphogenesis involved in neuron differentiation	5.1604	10.0000	ABLIM2 ARHGAP35 ARHGEF12 BRSK1 DCLK1 DOCK7 DPYSL3 FEZ1 GSK3B KIF13B MAP1B MAP2 MAPT PLXNB3 PSMD1 RUFY3 SPRED1 SPTBN1 SPTBN2 SPTBN4 SRCIN1 STMN1 TNIK TOP2B USP9X	ABLIM2 ANK1 ARHGEF12 CAMK2B COL3A1 CTTN DCLK1 DNM1 DPYSL2 DPYSL5 EGFR EP300 GIT1 GOLGA4 KCNQ2 KIF13B MAP1B MAP2 MINK1 NEFH PAK1 PLXNB3 PSMD4 PTPRZ1 PVRL1 RAB3A RAPGEF2 ROBO2 RTN4 SHANK1 SHANK3 SOD1 SPTBN2 SPTBN4 SRGAP2 TIAM1 TOP2B
GO:0016192	vesicle-mediated transport	5.0765	10.0000	ADD1 AP3D1 ARFGAP1 ARHGAP21 ATP6V1H CHMP6 CORO1A CSNK1A1 CTAGE5 CTNNB1 EPN1 EPN2 GBF1 HSPA5 HTT MYO5A NDRG4 NLGN2 PACSIN1 PARK2 PCLO PDPK1 PRKCE RIMS1 RIMS2 SPIRE1 SPTBN1 SPTBN2 SPTBN4 SRCIN1 SYN1 TSC2 UBQLN2	AAK1 ADD1 AKT2 AMPH ANK1 AP3B2 ARHGAP21 COL3A1 CORO1A CSNK1A1 CSNK1D CTAGE5 CTTN DMTN DNAJC6 DNM1 DPYSL2 ELMO1 ELMO3 EPN1 EPN2 FMN2 GAPVD1 GOLGA4 HSPA5 HTT KIF17 MAGI2 MYO5A NBAS PAK1 PCLO PDPK1 RAB11FIP5 RAB3A RAP1B RTN3 SEC16A SOD1 SPTBN2 SPTBN4 STX1B SYN1 TSC2 WIPF3
GO:0007409	axonogenesis	5.0243	10.0000	ABLIM2 ARHGAP35 ARHGEF12 BRSK1 DCLK1 DOCK7 DPYSL3 FEZ1 GSK3B KIF13B MAP1B MAP2 MAPT PLXNB3 PSMD1 RUFY3 SPRED1 SPTBN1 SPTBN2 SPTBN4 STMN1 TOP2B USP9X	ABLIM2 ANK1 ARHGEF12 CAMK2B COL3A1 CTTN DCLK1 DNM1 DPYSL2 DPYSL5 EGFR EP300 GIT1 GOLGA4 KCNQ2 KIF13B MAP1B MAP2 NEFH PAK1 PLXNB3 PSMD4 PTPRZ1 PVRL1 RAB3A RAPGEF2 ROBO2 RTN4 SPTBN2 SPTBN4 SRGAP2 TIAM1 TOP2B
GO:0007015	actin filament organization	5.5587	5.8542	ACTA1 ADD1 CORO1A DPYSL3 LCP1 MTOR MTSS1 PACSIN1 PARK2 PRKCE RUFY3 SPIRE1 SPTBN1 SPTBN2 SPTBN4	ADD1 ADD2 ARFIP2 ARHGEF2 CORO1A CTNNA1 CTTN DMTN FMN2 PAK1 PREX1 RICTOR SHANK1 SHANK3 SLC9A1 SPTBN2 SPTBN4 WIPF3
GO:0022604	regulation of cell morphogenesis	5.6301	5.7622	ARHGAP35 ARHGEF18 CORO1A CTNNB1 GSK3B HTT KIF13B MAP1B MAP2 MAPT PARK2 PLXNB3 PXN RIMS1 RIMS2 RUFY3 SRCIN1 TBC1D22B TNIK TTC3	CAMK2B CORO1A CTTN DMTN DPYSL2 EP300 GOLGA4 HTT KIF13B MAP1B MAP2 PALMD PLEKHO1 PLXNB3 PREX1 PTPRZ1 RAPGEF2 ROBO2 RTN4 SHANK3 STRIP1 TGFB1I1 TIAM1 WIPF3
GO:0000226	microtubule cytoskeleton organization	5.9181	5.2405	BRSK1 CAMSAP2 CEP76 CTNNB1 DOCK7 HTT KIF2A MAP1A MAP1B MAP2 MAP7 MAP7D1 MAP7D2 MAPT SIRT2 SPIRE1 STMN1 TPPP	ARHGEF2 BCAS3 BIRC7 CAMSAP2 CEP76 CSNK1D EML1 FMN2 HTT KIF2A MAP1A MAP1B MAP2 MAP4 NEFH RANBP10 RPS3 SIRT2 SRGAP2 TPR
GO:0060284	regulation of cell development	5.6420	5.3831	ARHGAP35 ASAP1 BAD CTNNB1 DOCK7 DPYSL3 FEZ1 GSK3B KIF13B MAP1B MAP2 MAP4K4 MAPT MTOR MYC NDRG4 NLGN2 PACSIN1 PARK2 PLXNB3 RIMS1 RIMS2 RUFY3 SIRT2 SRCIN1 TNIK TTC3	ACE ARHGEF2 CAMK2B COL3A1 CTNNA1 CTTN CX3CR1 DMTN DPYSL2 EP300 GOLGA4 GSK3A IMPACT KIF13B MAGI2 MAP1B MAP2 MYC PLXNB3 PML PREX1 PTPRZ1 RAPGEF2 ROBO2 RTN4 SHANK1 SHANK3 SIRT2 SRGAP2 TGFB1I1 TIAM1 TJP2
GO:0051494	negative regulation of cytoskeleton organization	5.0452	5.6655	ADD1 CORO1A MAP1A MAP1B PARK2 SPTBN1 SPTBN2 SPTBN4 STMN1	ADD1 ADD2 ARHGEF2 CORO1A DMTN MAP1A MAP1B SHANK1 SHANK3 SPTBN2 SPTBN4
GO:0060341	regulation of cellular localization	5.0510	5.3711	BAD CACNA1E CHMP6 CORO1A CTNNB1 GBF1 GSK3B HTT LCP1 MAP1B MAP4K4 MAPT MTOR MYO5A NCBP2 NDRG4 NLGN2 PARK2 PCLO PDPK1 PRKAR1B PRKCE RIMS1 RIMS2 RUFY3 RYR2 SIRT2 SPTBN1 SRCIN1 SYN1 TRIM28 WBP11	AKAP1 AKT2 BCAS3 C2CD2L CORO1A DBI DMTN DNM1 DPYSL2 EGFR EP300 GSK3A HTT KCNC3 LIME1 MADD MAGI2 MAP1B MYO5A NEFH OXCT1 PCLO PDPK1 PML PRKAR2A RAB11FIP5 RAB3A RAP1B RTN4 RYR2 SHANK3 SIRT2 SLC9A1 SP100 STX1B SYN1 TIAM1 TMBIM1 TPR YWHAE

Disease Models & Mechanisms • Supplementary information

GO:0010720	positive regulation of cell development	5.3194	5.0207	ARHGAP35 BAD CTNNB1 DPYSL3 FEZ1 GSK3B MAP1B MAPT MTOR MYC NDRG4 NLGN2 PACSIN1 PARK2 PLXNB3 RIMS1 RIMS2 RUFY3 SIRT2	ACE CAMK2B CX3CR1 DMTN EP300 GOLGA4 IMPACT MAGI2 MAP1B MYC PLXNB3 PML PREX1 PTPRZ1 RAPGEF2 ROBO2 RTN4 SHANK1 SHANK3 SIRT2 TGFB1I1 TIAM1
GO:0071363	cellular response to growth factor stimulus	10.0000		ARHGEF12 ARHGEF18 BAD BYSL CORO1A CTNNB1 FEZ1 GSK3B HHIP HSPA5 MAP3K7 MCF2L MTOR MYC PDPK1 PRKAR1B PRKCE PSMD1 PXN RYR2 SIRT2 SPRED1 SPTBN1 SPTBN2 SPTBN4 TMEM100 TSC2 USP9X	
GO:0007268	synaptic transmission		10.0000		AMPH APBA1 BSN CAMK2B CASK CX3CR1 DBI DNM1 DTNA EGFR GABBR2 HCN2 HTT KCNC1 KCNC3 KCNQ2 MINK1 MYO5A PAK1 PCLO PDPK1 RAB3A RAP1B RAPGEF2 SHANK1 SHANK3 SHISA6 SLC1A4 SLC6A5 SPTBN2 STX1B SYN1
GO:0044802	single-organism membrane organization		10.0000		AKT2 ANK1 CORO1A CSNK1D DMTN EGFR GOLGA4 GPR158 GSK3A HTT KCNIP4 MAGI2 MYC MYO5A NUP85 PACS2 PAK1 PDPK1 PEX19 RAB3A RANBP2 RAPGEF2 RPS28 RPS3 SEC16A SHANK3 SLC25A46 SLC9A1 SOD1 SPTBN4 STX1B TIAM1 TMBIM1 TPR TSC2 YWHAE
GO:0031346	positive regulation of cell projection organization	10.0000		ARHGAP35 DPYSL3 FEZ1 GSK3B HTT MAP1B MAPT MTOR NDRG4 NLGN2 PACSIN1 PARK2 PLXNB3 RIMS1 RIMS2 RUFY3	
GO:0007264	small GTPase mediated signal transduction		10.0000		ARFGEF3 ARFIP2 ARHGAP5 ARHGEF12 ARHGEF2 ARHGEF33 CAMK2B COL3A1 CTNNA1 CTTN EGFR ELMO1 KIF2A MADD NUP85 PAK1 PDPK1 PPP1R14A PREX1 PSMD4 RAB12 RAB3A RALGPS1 RANBP2 RAP1B RAPGEF2 RGL3 SIPA1L2 SPTBN2 SPTBN4 SRGAP2 TIAM1 TSC2 WIPF3 YWHAE
GO:1901701	cellular response to oxygen-containing compound		10.0000		ADD1 AKAP1 AKT2 ARHGEF2 ATP6V0A2 C2CD2L CAMK2B COL3A1 CTNNA1 CX3CR1 DMTN EGFR EIF4B EP300 GSK3A HCN2 HNRNPU IMPACT MYO5A NEFH OXCT1 PAK1 PDPK1 PRKAR2A PSMD4 RAB11FIP5 RAP1B RAPGEF2 RPS3 RYR2 SASH1 SIRT2 SLC9A1 SOD1 SPTBN2 SPTBN4 TIAM1 TSC2 YBX1
GO:0032535	regulation of cellular component size		10.0000		ADD1 ADD2 ARFIP2 ARHGAP5 CLNS1A CORO1A CTTN DMTN DPYSL2 EP300 FMN2 GOLGA4 MAP1B PREX1 RICTOR RTN4 SHANK3 SPTBN2 SPTBN4 TSC2 WIPF3
GO:0061024	membrane organization		10.0000		AKT2 ANK1 CORO1A CSNK1D CTAGE5 DMTN DNAJC6 EGFR ELMO1 GOLGA4 GPR158 GSK3A HTT KCNIP4 MAGI2 MYC MYO5A NUP85 PACS2 PAK1 PDPK1 PEX19 RAB3A RANBP2 RAPGEF2 RPS28 RPS3 SEC16A SHANK3 SLC25A46 SLC9A1 SOD1 SPTBN4 STX1B TIAM1 TMBIM1 TPR TSC2 YWHAE
GO:0030833	regulation of actin filament polymerization		10.0000		ADD1 ADD2 ARFIP2 CORO1A CTTN DMTN FMN2 PREX1 RICTOR SPTBN2 SPTBN4 WIPF3

Disease Models & Mechanisms • Supplementary information

GO:0061564	axon development		10.0000		ABLIM2 ANK1 ARHGEF12 CAMK2B COL3A1 CTNNA1 CTTN DCLK1 DNM1 DPYSL2 DPYSL5 EGFR EP300 GIT1 GOLGA4 KCNQ2 KIF13B MAP1B MAP2 NEFH PAK1 PLXNB3 PSMD4 PTPRZ1 PVRL1 RAB3A RAPGEF2 ROBO2 RTN4 SPTBN2 SPTBN4 SRGAP2 TIAM1 TOP2B
GO:0051336	regulation of hydrolase activity		10.0000		AKT2 AMPH ARFGEF3 ARHGAP21 ARHGAP5 ARHGEF12 ARHGEF2 ARHGEF33 BCAS3 BIRC7 CAMK2B DAP EGFR EP300 GAPVD1 GIT1 HNRNPU HSPA5 HTT LMTK3 MADD MAGI2 MYC PCP2 PDPK1 PKP4 PLXNB3 PML PREX1 PRKAR2A RAB3A RALGPS1 RAPGEF2 RGL3 RICTOR RPS3 RYR2 SIPA1L2 SMAP2 SOD1 SRGAP2 TIAM1 TSC2 YWHAE
GO:0010976	positive regulation of neuron projection development	10.0000		ARHGAP35 DPYSL3 FEZ1 GSK3B MAP1B MAPT MTOR NDRG4 NLGN2 PACSIN1 PARK2 PLXNB3 RIMS1 RIMS2 RUFY3	
GO:0051345	positive regulation of hydrolase activity		10.0000		AKT2 AMPH ARFGEF3 ARHGAP21 ARHGAP5 ARHGEF12 ARHGEF2 ARHGEF33 BCAS3 CAMK2B DAP EGFR GAPVD1 GIT1 HNRNPU HSPA5 MADD MAGI2 MYC PCP2 PDPK1 PKP4 PML PREX1 PRKAR2A RAB3A RALGPS1 RAPGEF2 RGL3 RPS3 RYR2 SIPA1L2 SMAP2 SRGAP2 TIAM1 TSC2
GO:0090066	regulation of anatomical structure size		10.0000		ACE ADD1 ADD2 ARFIP2 ARHGAP5 CLNS1A CORO1A CTTN DMTN DPYSL2 EGFR EP300 FMN2 GOLGA4 MAP1B PREX1 RICTOR RTN4 SHANK3 SOD1 SPTBN2 SPTBN4 TSC2 WIPF3
GO:0060322	head development		10.0000		ARID1A CEND1 CKB COL3A1 CX3CR1 DBI DCLK1 DPYSL2 EGFR EML1 HSPA5 HTT KCNC1 NEFH NFIX OXCT1 PAK1 PHF8 RAPGEF2 ROBO2 RPH3A RTN4 SEC16A SHANK3 SIRT2 SPTBN2 SRGAP2 TOP2B YWHAE
GO:0045664	regulation of neuron differentiation	10.0000		ARHGAP35 ASAP1 DPYSL3 FEZ1 GSK3B KIF13B MAP1B MAP2 MAP4K4 MAPT MTOR NDRG4 NLGN2 PACSIN1 PARK2 PLXNB3 RIMS1 RIMS2 RUFY3 SRCIN1 TNIK TTC3	
GO:0071407	cellular response to organic cyclic compound		10.0000		AKAP1 AKT2 ATP1A3 BCAS3 CTNNA1 DMTN EGFR EP300 GSK3A HCN2 HNRNPU IMPACT NEFH PAK1 RAP1B RAPGEF2 RYR2 SFR1 SIRT2 SLC9A1 SOD1
GO:1901699	cellular response to nitrogen compound		10.0000		AKAP1 AKT2 ARHGEF2 ATP6V0A2 CAMK2B COL3A1 CTNNA1 DMTN EGFR EIF4B EP300 GSK3A HCN2 IMPACT MYO5A PAK1 PDPK1 PRKAR2A PSMD4 RAP1B RAPGEF2 RYR2 SIRT2 SLC9A1 SOD1 SPTBN2 SPTBN4 TSC2 YBX1
GO:0010243	response to organonitrogen compound		10.0000		AKAP1 AKT2 ARHGEF2 ATP6V0A2 CAMK2B COL3A1 CTNNA1 DMTN DPYSL2 EGFR EIF4B EP300 GSK3A HCN2 IMPACT KCNC1 MYC MYO5A NEFH PAK1 PDPK1 PRKAR2A PSMD4 RANBP2 RAP1B RAPGEF2 RYR2 SCAP SIRT2 SLC9A1 SOD1 SPTBN2 SPTBN4 TSC2 YBX1

Disease Models & Mechanisms • Supplementary information

GO:0043547	positive regulation of GTPase activity		10.0000		AKT2 AMPH ARFGEF3 ARHGAP21 ARHGAP5 ARHGEF12 ARHGEF2 ARHGEF33 BCAS3 GAPVD1 GIT1 MADD PCP2 PKP4 PREX1 RALGPS1 RAPGEF2 RGL3 SIPA1L2 SMAP2 SRGAP2 TIAM1 TSC2
GO:0016358	dendrite development		10.0000		CAMK2B DCLK1 GIT1 GSK3A MAP1B MAP2 MINK1 PAK1 PREX1 PTPRZ1 RAPGEF2 SHANK1 SHANK3 SRGAP2 STRN TIAM1
GO:0071417	cellular response to organonitrogen compound		10.0000		AKAP1 AKT2 ARHGEF2 ATP6V0A2 CAMK2B COL3A1 CTNNA1 DMTN EGFR EIF4B EP300 GSK3A HCN2 IMPACT MYO5A PAK1 PDPK1 PRKAR2A PSMD4 RAP1B RAPGEF2 RYR2 SIRT2 SLC9A1 SOD1 SPTBN2 SPTBN4 TSC2 YBX1
GO:0071495	cellular response to endogenous stimulus		10.0000		AKAP1 AKT2 ARHGEF2 ATP1A3 ATP6V0A2 BCAS3 CAMK2B COL3A1 CORO1A CTNNA1 CX3CR1 DMTN EGFR EIF4B EP300 GSK3A HCN2 HNRNPU HSPA5 IMPACT MAGI2 MAP3K7 MYC MYO5A NEFH PAK1 PDPK1 PML PRKAR2A PSMD4 RAP1B RAPGEF2 RICTOR ROBO2 RPS3 RYR2 SFR1 SIRT2 SLC9A1 SOD1 SPTBN2 SPTBN4 TGFB1I1 TSC2 YBX1
GO:0031344	regulation of cell projection organization	10.0000		ARHGAP35 ASAP1 DPYSL3 FEZ1 GSK3B HTT KIF13B MAP1B MAP2 MAP4K4 MAPT MTOR NDRG4 NLGN2 PACSIN1 PARK2 PLXNB3 RIMS1 RIMS2 RUFY3 SRCIN1 TBC1D22B TNIK	
GO:0010975	regulation of neuron projection development	10.0000		ARHGAP35 ASAP1 DPYSL3 FEZ1 GSK3B KIF13B MAP1B MAP2 MAP4K4 MAPT MTOR NDRG4 NLGN2 PACSIN1 PARK2 PLXNB3 RIMS1 RIMS2 RUFY3 SRCIN1 TNIK	
GO:0042391	regulation of membrane potential	10.0000		ATP1A1 ATP1A3 BAD CACNA1E DSG2 GCLM GSK3B HTT MYC NLGN2 NOS1AP PARK2 PRKCE RIMS1 RIMS2 RYR2 SCN11A SCN2A	
GO:0032956	regulation of actin cytoskeleton organization		10.0000		ADD1 ADD2 ARFIP2 BCAS3 CORO1A CTTN DMTN EP300 FMN2 PAK1 PREX1 RICTOR SHANK1 SHANK3 SLC9A1 SPTBN2 SPTBN4 WIPF3
GO:0007420	brain development		10.0000		ARID1A CEND1 CKB COL3A1 CX3CR1 DBI DCLK1 DPYSL2 EGFR EML1 HSPA5 HTT KCNC1 NEFH NFIX OXCT1 PAK1 PHF8 RAPGEF2 ROBO2 RPH3A RTN4 SEC16A SHANK3 SIRT2 SPTBN2 SRGAP2 TOP2B YWHAE
GO:0043087	regulation of GTPase activity		10.0000		AKT2 AMPH ARFGEF3 ARHGAP21 ARHGAP5 ARHGEF12 ARHGEF2 ARHGEF33 BCAS3 GAPVD1 GIT1 MADD PCP2 PKP4 PLXNB3 PREX1 RALGPS1 RAPGEF2 RGL3 RICTOR SIPA1L2 SMAP2 SOD1 SRGAP2 TIAM1 TSC2
GO:0016477	cell migration		5.8924		ACE AKT2 ARHGAP5 ASTN1 BCAS3 CEND1 COL18A1 COL3A1 CORO1A CTTN CX3CR1 DCLK1 DMTN EGFR ELMO1 ELMO3 GSK3A HSPA5 MAGI2 MINK1 MYC NUP85 PAK1 PDPK1 PLEKHO1 PLXNB3 PML PREX1 PTPRZ1 RAPGEF2 RTN4 SASH1 SLC7A5 SLC9A1 SP100 SRGAP2 TIAM1 TNS1 TOP2B YWHAE

GO:0031334	positive regulation of protein complex assembly	5.7721		CORO1A CTNNB1 GSK3B LCP1 MAP1B MAPT MTOR PRKCE RIMS1 SPIRE1 TAF1 TPPP	
GO:0048870	cell motility		5.7623		ACE AKT2 ARHGAP5 ASTN1 BCAS3 CEND1 COL18A1 COL3A1 CORO1A CTNNA1 CTTN CX3CR1 DCLK1 DMTN EGFR ELMO1 ELMO3 GSK3A HSPA5 LDHC MAGI2 MINK1 MYC NUP85 PAK1 PDPK1 PLEKHO1 PLXNB3 PML PREX1 PTPRZ1 RAPGEF2 RTN4 SASH1 SLC7A5 SLC9A1 SP100 SRGAP2 TIAM1 TNS1 TOP2B YWHAE
GO:0051674	localization of cell		5.7623		ACE AKT2 ARHGAP5 ASTN1 BCAS3 CEND1 COL18A1 COL3A1 CORO1A CTNNA1 CTTN CX3CR1 DCLK1 DMTN EGFR ELMO1 ELMO3 GSK3A HSPA5 LDHC MAGI2 MINK1 MYC NUP85 PAK1 PDPK1 PLEKHO1 PLXNB3 PML PREX1 PTPRZ1 RAPGEF2 RTN4 SASH1 SLC7A5 SLC9A1 SP100 SRGAP2 TIAM1 TNS1 TOP2B YWHAE
GO:0007267	cell-cell signaling		5.6590		AMPH APBA1 BSN C2CD2L CAMK2B CASK CX3CR1 DBI DNM1 DTNA EGFR GABBR2 HCN2 HTT KCNC1 KCNC3 KCNQ2 MADD MINK1 MYO5A OXCT1 PAK1 PCLO PDPK1 PKP4 PRKAR2A RAB11FIP5 RAB3A RAP1B RAPGEF2 RYR2 SHANK1 SHANK3 SHISA6 SLC1A4 SLC6A5 SPTBN2 STX1B SYN1 TIAM1
GO:0008542	visual learning	5.6364		ATP1A3 CACNA1E HTT MTOR NDRG4 PPP1R1B TANC1	
GO:0051881	regulation of mitochondrial membrane potential	5.5854		BAD GCLM GSK3B HTT MYC PARK2 PRKCE	
GO:0072657	protein localization to membrane		5.5726		AKT2 ANK1 DMTN EGFR GOLGA4 GPR158 KCNIP4 MAGI2 MYO5A PACS2 PAK1 PDPK1 PEX19 RAPGEF2 RPS28 RPS3 SHANK3 SPTBN4 TIAM1 TMBIM1 TSC2 YWHAE
GO:1901698	response to nitrogen compound		5.5451		AKAP1 AKT2 ARHGEF2 ATP6V0A2 CAMK2B COL3A1 CTNNA1 DMTN DPYSL2 EGFR EIF4B EP300 GSK3A HCN2 IMPACT KCNC1 MYC MYO5A NEFH PAK1 PDPK1 PRKAR2A PSMD4 RANBP2 RAP1B RAPGEF2 RYR2 SCAP SIRT2 SLC9A1 SOD1 SPTBN2 SPTBN4 TSC2 YBX1
GO:0030041	actin filament polymerization		5.5179		ADD1 ADD2 ARFIP2 CORO1A CTTN DMTN FMN2 PREX1 RICTOR SPTBN2 SPTBN4 WIPF3
GO:0060627	regulation of vesicle-mediated transport	5.5075		ADD1 ARFGAP1 ARHGAP21 CHMP6 CORO1A EPN2 MYO5A NDRG4 PACSIN1 PARK2 PCLO PDPK1 RIMS1 RIMS2 SYN1 TSC2 UBQLN2	
GO:0018105	peptidyl-serine phosphorylation	5.4637		BRSK1 CNKSR3 CSNK1A1 DOCK7 DYRK1A GSK3B MAST1 MTOR PDPK1 PLCL1 PRKCE SPTBN4 TAF1	
GO:0080135	regulation of cellular response to stress		5.4451		ARHGEF2 BIRC7 CAMK2B CTTN EGFR EP300 GSK3A HSPA5 IMPACT MAP3K7 MINK1 MYC NUP85 PAK1 PML PTGES3 RAB12 RANBP2 RPS3 RTN4 SASH1 SOD1 TIAM1 TPR TSC2 YWHAE

Disease Models & Mechanisms • Supplementary information

GO:0008064	regulation of actin polymerization or depolymerization		5.4349		ADD1 ADD2 ARFIP2 CORO1A CTTN DMTN FMN2 PREX1 RICTOR SPTBN2 SPTBN4 WIPF3
GO:0007167	enzyme linked receptor protein signaling pathway	5.4261		ANKS1A ARHGEF12 ARHGEF18 ATP6V1H BAD CTNNB1 DOK1 EIF4B EPN1 GSK3B HHIP HSPA5 MAP3K7 MCF2L MTOR MTSS1 MYC NDRG4 PDPK1 PRKAR1B PRKCE PSMD1 PXN RYR2 SIRT2 SPRED1 SPTBN1 SPTBN2 SPTBN4 TMEM100 TSC2 USP9X	
GO:0030832	regulation of actin filament length		5.4077		ADD1 ADD2 ARFIP2 CORO1A CTTN DMTN FMN2 PREX1 RICTOR SPTBN2 SPTBN4 WIPF3
GO:0007632	visual behavior	5.3906		ATP1A3 CACNA1E HTT MTOR NDRG4 PPP1R1B TANC1	
GO:0010769	regulation of cell morphogenesis involved in differentiation		5.3843		CAMK2B CTTN DMTN DPYSL2 EP300 GOLGA4 KIF13B MAP1B MAP2 PLXNB3 PREX1 PTPRZ1 RAPGEF2 ROBO2 RTN4 SHANK3 TGFB1I1 TIAM1
GO:0050769	positive regulation of neurogenesis	5.3753		ARHGAP35 CTNNB1 DPYSL3 FEZ1 GSK3B MAP1B MAPT MTOR MYC NDRG4 NLGN2 PACSIN1 PARK2 PLXNB3 RIMS1 RIMS2 RUFY3	
GO:0007417	central nervous system development		5.3643		ARID1A CEND1 CKB COL3A1 CX3CR1 DBI DCLK1 DPYSL2 EGFR EML1 HSPA5 HTT KCNC1 MAP2 NEFH NFIX OXCT1 PAK1 PHF8 PTPRZ1 RAPGEF2 ROBO2 RPH3A RTN4 SEC16A SHANK3 SIRT2 SPTBN2 SPTBN4 SRGAP2 TOP2B YWHAE
GO:1990089	response to nerve growth factor		5.3277		CORO1A EP300 KCNC1 MAGI2 RAPGEF2 RPS3
GO:0034330	cell junction organization		5.3070		ACE BCAS3 CTNNA1 CTTN DMTN DSG2 PDPK1 PKP4 PVRL1 RAP1B RAPGEF2 SLC9A1 STRN TLN2 TNS1
GO:0043269	regulation of ion transport		5.2864		ACE AKT2 CAMK2B CASK CORO1A DBI DPYSL2 HCN2 HTT KCNC1 KCNC3 KCNIP4 KCNQ2 LIME1 MINK1 MYO5A PDPK1 PML RTN4 RYR2 SHANK1 SHANK3 SLC9A1 SPTBN4 YWHAE
GO:0045666	positive regulation of neuron differentiation	5.2848		ARHGAP35 DPYSL3 FEZ1 GSK3B MAP1B MAPT MTOR NDRG4 NLGN2 PACSIN1 PARK2 PLXNB3 RIMS1 RIMS2 RUFY3	
GO:0051130	positive regulation of cellular component organization	5.2510		ADD1 ARHGAP35 BAD CORO1A CTNNB1 DPYSL3 FEZ1 GSK3B HTT LCP1 MAP1B MAPT MTOR MTSS1 NDRG4 NLGN2 PACSIN1 PARK2 PDPK1 PLXNB3 PRKCE RIMS1 RIMS2 RUFY3 SIRT2 SPIRE1 SPTBN1 TAF1 TPPP WBP11	
GO:0034329	cell junction assembly		5.2460		ACE BCAS3 CTNNA1 CTTN DMTN PDPK1 PKP4 PVRL1 RAP1B RAPGEF2 SLC9A1 STRN TLN2 TNS1
GO:1901880	negative regulation of protein depolymerization		5.2333		ADD1 ADD2 ARHGEF2 DMTN MAP1A MAP1B SPTBN2 SPTBN4

Disease Models & Mechanisms • Supplementary information

GO:0038179	neurotrophin signaling pathway	5.2198		ARHGEF12 ARHGEF18 BAD CORO1A GSK3B MCF2L MTOR PDPK1 PRKAR1B PRKCE PSMD1 SPRED1 SPTBN1 SPTBN2 SPTBN4 TSC2	
GO:0008543	fibroblast growth factor receptor signaling pathway	5.2141		BAD CTNNB1 GSK3B HHIP MTOR PDPK1 PRKAR1B PRKCE PSMD1 SPRED1 SPTBN1 SPTBN2 SPTBN4 TSC2	
GO:0018209	peptidyl-serine modification	5.1976		BRSK1 CNKSR3 CSNK1A1 DOCK7 DYRK1A GSK3B MAST1 MTOR PDPK1 PLCL1 PRKCE SPTBN4 TAF1	
GO:0010639	negative regulation of organelle organization		5.1770		ADD1 ADD2 ARHGEF2 CDC26 CORO1A DMTN HNRNPU MAP1A MAP1B PHF8 PML SHANK1 SHANK3 SPTBN2 SPTBN4 TOM1L2 TPR
GO:0007169	transmembrane receptor protein tyrosine kinase signaling pathway	5.1123		ANKS1A ARHGEF12 ARHGEF18 ATP6V1H BAD CTNNB1 DOK1 EIF4B EPN1 GSK3B HHIP MCF2L MTOR MTSS1 NDRG4 PDPK1 PRKAR1B PRKCE PSMD1 PXN SIRT2 SPRED1 SPTBN1 SPTBN2 SPTBN4 TSC2	
GO:0043242	negative regulation of protein complex disassembly		5.0955		ADD1 ADD2 ARHGEF2 DMTN MAP1A MAP1B SPTBN2 SPTBN4
GO:0044089	positive regulation of cellular component biogenesis	5.0875		CORO1A CTNNB1 DPYSL3 GSK3B HTT LCP1 MAP1B MAPT MTOR MTSS1 NLGN2 PRKCE RIMS1 SPIRE1 TAF1 TPPP	
GO:0030705	cytoskeleton-dependent intracellular transport		5.0461		AP3B2 APBA1 ARHGAP21 DYNC1I1 HTT KIF13B KIF17 MAP1B NEFH SOD1
GO:0030900	forebrain development		4.9895		ARID1A COL3A1 CX3CR1 DBI DCLK1 DPYSL2 EGFR HTT KCNC1 NEFH PAK1 RAPGEF2 ROBO2 RTN4 SHANK3 SRGAP2 TOP2B YWHAE
GO:0010918	positive regulation of mitochondrial membrane potential	4.9586		BAD GSK3B MYC	
GO:0043244	regulation of protein complex disassembly		4.9531		ADD1 ADD2 ARHGEF2 DMTN MAP1A MAP1B SPTBN2 SPTBN4 UPF1
GO:0022603	regulation of anatomical structure morphogenesis		4.9481		ADD1 CAMK2B CORO1A CTTN CX3CR1 DMTN DPYSL2 EP300 GOLGA4 HTT KIF13B MAGI2 MAP1B MAP2 MYC PALMD PLEKHO1 PLXNB3 PML PREX1 PTPRZ1 RAPGEF2 ROBO2 RTN4 SASH1 SHANK3 SP100 STRIP1 TGFB1I1 TIAM1 TMBIM1 WIPF3
GO:0044344	cellular response to fibroblast growth factor stimulus	4.9122		BAD CTNNB1 GSK3B HHIP MTOR PDPK1 PRKAR1B PRKCE PSMD1 SPRED1 SPTBN1 SPTBN2 SPTBN4 TSC2	

Disease Models & Mechanisms • Supplementary information
